# Photochemically-Enabled Umpolung Conversion of 2-Acyloxybenzaldehydes into 2-Hydroxybenzofuranones

**DOI:** 10.3390/molecules30153080

**Published:** 2025-07-23

**Authors:** Victoria E. Opryshko, Svetlana A. Krasnova, Andrey A. Mikhaylov, Yulia A. Bogdanova, Alexander Yu. Smirnov, Mikhail S. Baranov, Dmitrii S. Ivanov

**Affiliations:** 1 Institute of Bioorganic Chemistry, Russian Academy of Sciences, Miklukho-Maklaya 16/10, 117997 Moscow, Russia; 2 Laboratory of Medicinal Substances Chemistry, Institute of Translational Medicine, Pirogov Russian National Research Medical University, Ostrovitianov 1, 117997 Moscow, Russia

**Keywords:** cyclization, photochemical reactions, benzofuranones, 2-acyloxybenzaldehydes, carbonyl umpolung, acyl radical, ketyl radical

## Abstract

2-Acyloxybenzaldehydes are converted into 2-hydroxybenzofuranones in good to excellent yields (60–99%). The reaction proceeds at room temperature in DMSO upon 365 nm LED irradiation under photocatalyst-free conditions. The present atom-economical synthetic approach represents the aldehyde group umpolung reactivity.

## 1. Introduction

**Scheme 1:**
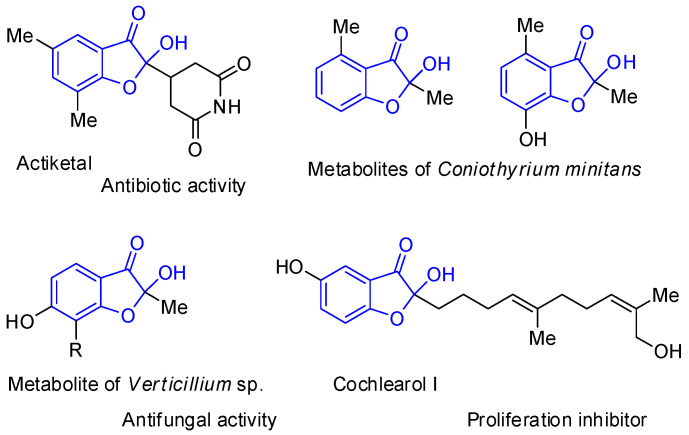
Natural and biologically active compounds containing 2-hydroxybenzofuranone core.

Benzofuranones with a hemiacetal moiety (i.e., 2-hydroxybenzofuranones) are a common structural motif in natural compounds (Scheme 1). These compounds and the corresponding acetals were identified in fungi [1,2,3,4], plants [5,6], and bacteria [7,8]. These compounds exhibit various biological activities, including cytotoxic [9] and antifungal effects [5], cyclooxygenase-2 inhibition [10,11], and antioxidant properties [6]. 2-Hydroxybenzofuranones attract significant interest in the search for novel antibiotics [7,12,13,14,15,16].

**Scheme 2:**
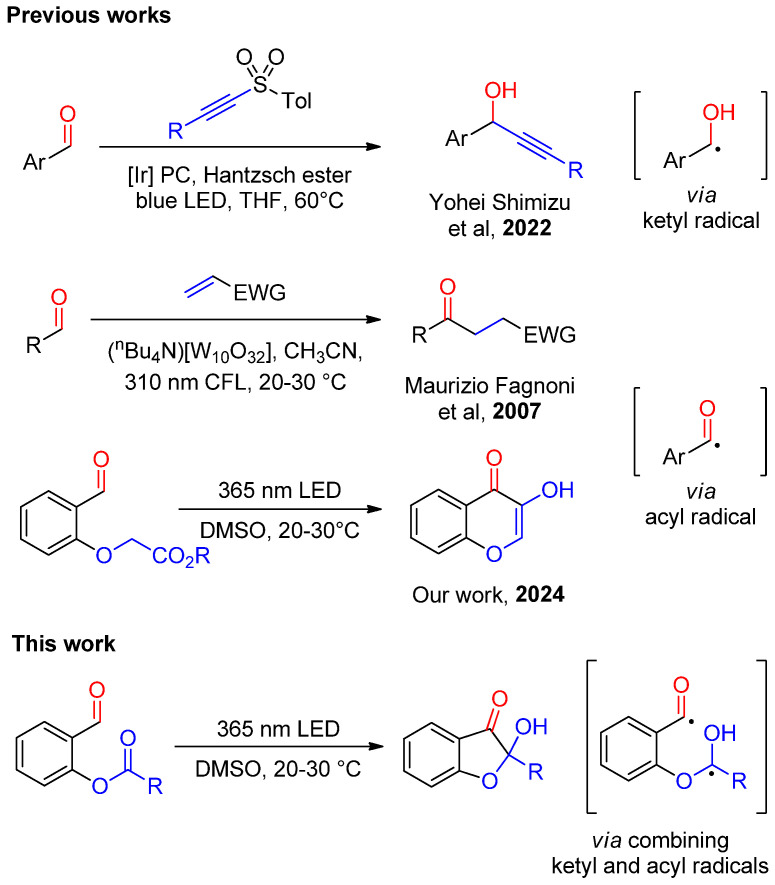
Umpolung approaches to aldehyde group phototransformations [35,38,39].

Organic photochemistry is an important part of modern organic chemistry. The excitation of molecules opens a way to realize unique reactivity that is difficult to access by other methods [17]. Photochemical transformations are successfully used in the synthesis of natural and biologically active compounds [18,19], as well as in industrial [20] and pharmaceutical [21] processes. Application of powerful monochromatic light-emitting diodes (LEDs), flow chemistry, and photoelectrochemical reactions [22,23] expand the palette of modern photochemical methods. The use of natural sunlight [24,25] and atmospheric oxygen in photoreactions [26,27] aligns with the principles of “green chemistry” [28,29]. The unusual photoinduced reactivity can achieve umpolung reactions, in which the polarity of the functional group is inverted [30,31]. The strategy of carbonyl polarity reversal through radical processes gives rise to a wide range of products [32,33,34]. Thus, recent works present photoinduced umpolung aldehyde transformations achieved via either ketyl radical [35,36] or acyl radical reactions (Scheme 2) [37,38]. In our recent work, we demonstrated photochemical acyl radical generation from benzaldehyde derivatives that was applied for intramolecular cyclization into chromanones [39]. In this work, we continue the systematic investigation of photoinduced transformations of ortho-substituted benzaldehydes. We propose an approach that may combine both ketyl and acyl radical moieties for photoinduced umpolung transformation of 2-acyloxybenzaldehydes into 2-hydroxybenzofuranones.

## 2. Results and Discussion

During the performed systematic photochemical screening of salicylaldehyde derivatives reactivity, their acyl derivatives **1** were studied. We found that 2-acetoxybenzaldehyde **1a** is converted into 2-hydroxybenzofuranone **2a** under 365 nm irradiation in DMSO. While such cyclic hemiacetals have been previously reported, existing synthetic methods are typically complex and low-yielding [40,41], which prompted us to study the found phototransformation in more detail. We screened various solvents (see Supplementary Materials, Part 1) and demonstrated that DMSO is uniquely effective for this transformation (Table 1), similar to all those we have identified previously [39,42]. Aside from DMSO, the target product was formed only in DMF and DMAC, albeit in significantly lower yields. In all other solvents, complex mixtures were observed, along with the oxidation product of the starting aldehyde **1a**—2-acetoxybenzoic acid.

The addition of small amounts of water or conducting the reaction in open air had negligible effects on the process (Supplementary Materials, Part 2). However, the observed oxidation to 2-acetoxybenzoic acid in several solvents (e.g., Table 1, Entry 1, 6, 9, 12) suggests that an inert atmosphere is preferable. Although the substrate absorbs weakly at 365 nm (see Supplementary Materials, Part 3), this irradiation wavelength successfully induced phototransformation, whereas longer wavelengths were ineffective. Optimizing the light source to match the absorption maximum (e.g., using shorter wavelengths) may further enhance reaction efficiency in future work. Heating slightly increased the reaction rate (Supplementary Materials, Part 4). However, since precise temperature control during phototransformation is challenging, we performed the reaction at room temperature.

Thus, we propose carrying out the reaction in dry DMSO under an inert atmosphere with 365 nm irradiation at room temperature. However, the reaction was found rather tolerant to set-up conditions (such as the presence of wet DMSO, open-air atmosphere, heating, or shorter-wavelength diodes), which could potentially be employed.

Using the optimized conditions, we investigated the reactivity of a series of derivatives **1** and **3**. The structures of all corresponding reaction products **2** and **4** were confirmed by ^1^H and ^13^C NMR spectroscopy (Supplementary Materials, Part 8), HRMS analysis, and comparison with previously reported spectral data for analogous derivatives.

**Scheme 3:**
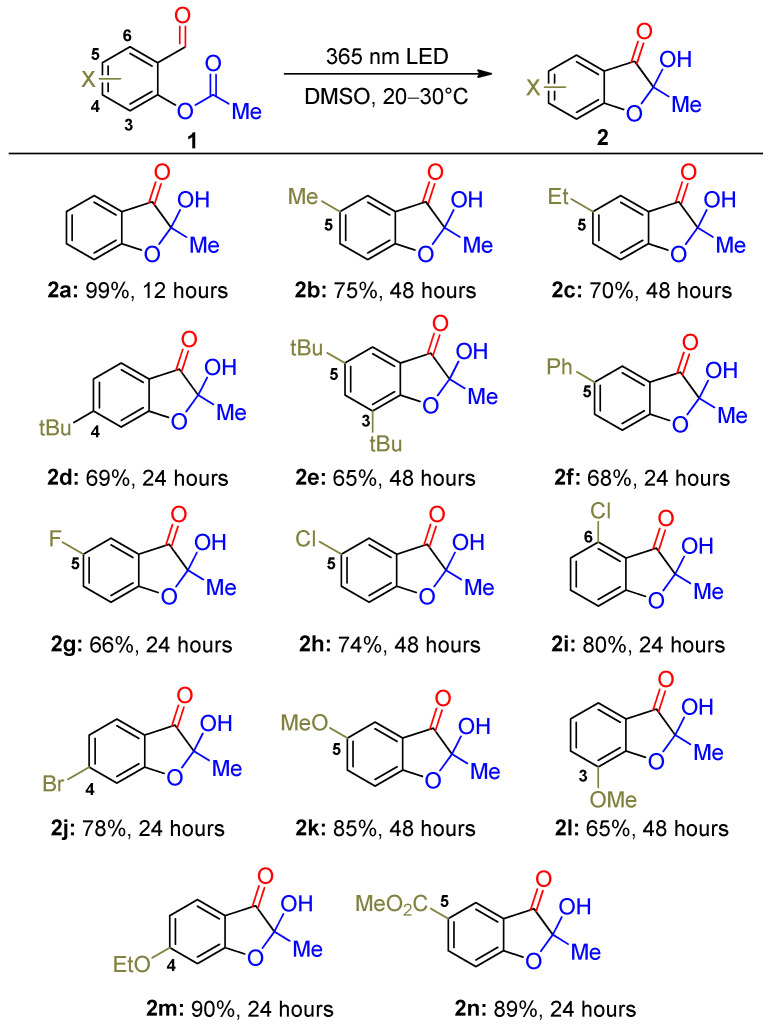
Scope of 2-acetyloxybenzaldehydes **1** photoreaction. The times for complete conversion of the starting compounds **1** are indicated.

First, we studied the acetoxy derivatives **1** with different substitution patterns in the benzene ring (Scheme 3).

Based on the obtained results, it can be concluded that the yields of substituted compounds **2b**–**n** are lower than the yield of compound **2a**. Moreover, substrates **1a**–**n** require a longer irradiation time for transformation. A noticeable decrease in the yield was observed for all derivatives containing substituents in position 5 (para to the oxygen atom). The only exception was derivative **2n** with an electron-withdrawing substituent in this position, which was formed in high yield. A similar effect was observed for 3-substituted derivatives **2e** and **2l**. However, in all cases the reaction yields ranged from good to high, which indicates the sufficient versatility of the reaction for acetoxy derivatives.

**Scheme 4:**
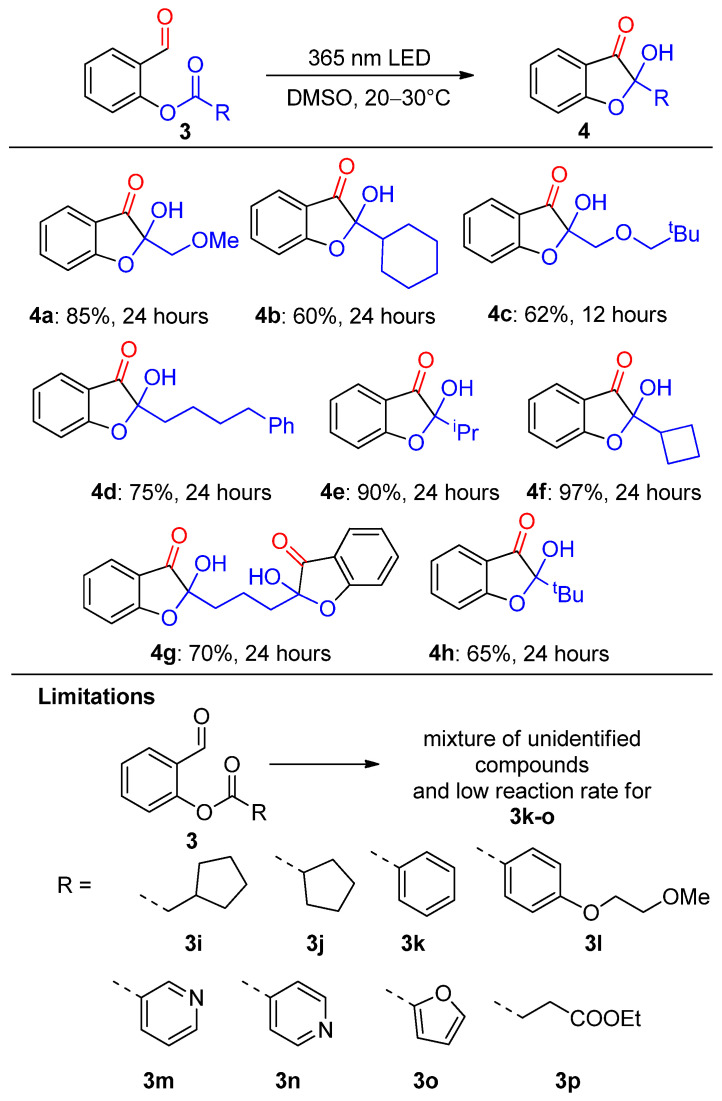
Scope of the 2-acyloxybenzaldehydes **3** photoreaction. The times for complete conversion of the starting compounds **3** are indicated.

Next, we briefly examined the scope of the reaction for other carboxylate derivatives **3** (Scheme 4). The yields for all these substances **4** are also lower than that of derivative **2a**, and the transformation also requires a longer irradiation time. At the same time, it was impossible to reveal any strict dependence between the structure of the substrate and the yield of the reaction. The lowest yield was observed for the cyclohexyl derivative **4b**, while the very similar isopropyl and cyclobutyl derivatives **4e** and **4f** showed the best yields. We also succeeded in obtaining derivatives with cyclohexyl and cyclobutyl (**4b** and **4f**) but not with those belonging to a cyclopentyl group. The only reliable exception was the phototransformation of aryl derivatives (**3l**–**o**), for which the rate was very low and which led to the formation of complex mixtures.

A detailed analysis of substituent effects on products’ yields, including potential mechanistic explanations, is presented below, following the mechanistic discussion.

**Scheme 5:**
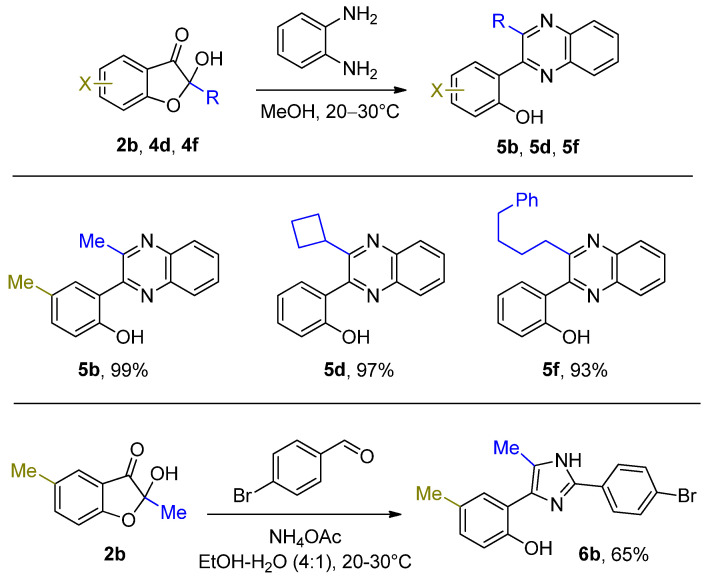
Synthesis of heterocyclic derivatives **5** and **6** from furanones **1** and **3**.

Hypothetically, hemiacetals **2** and **4** may exist in equilibrium with the open form [43]. However, we did not observe such forms for any of the products by NMR spectroscopy. The high stability of the hemiacetal can be explained by the increased electrophilicity of the 1,2-dicabonyl **moiety** and the cyclic structure of the product. However, product **4g**, though capable of forming two diastereomers, was obtained exclusively as a single isomer, likely the thermodynamically stable one stabilized by intramolecular hydrogen bonding. The absence of the second diastereomer supports possible transition between the ketone and hemiacetal tautomers. Next, we further confirmed the tautomeric equilibrium showing that these substances can be used as 1,2-dicarbonyl compounds in the synthesis of heterocyclic derivatives. Thus, we synthesized three quinoxaline derivatives—**5b**, **5d**, and **5f**—in nearly quantitative yields and one imidazole **6b** with 65% yield (Scheme 5).

**Scheme 6:**
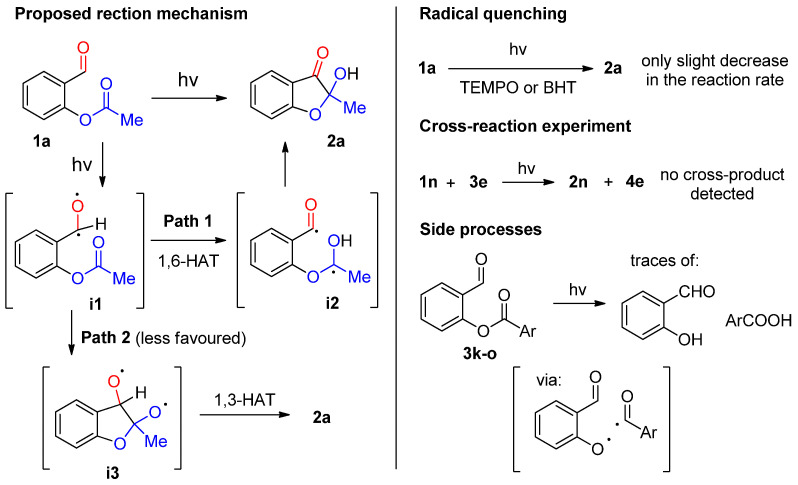
Proposed reaction mechanism and related studies.

Finally, we briefly studied the possible reaction mechanism. First, a detailed study of the transformation in DMSO-*d*_6_ (Supplementary Materials, Part 5) revealed that this reaction proceeds directly without detectable intermediates, forming a single product via the first-order reaction. Second, we demonstrated that the presence of TEMPO ((2,2,6,6-tetramethylpiperidin-1-yl)oxidanyl) and BHT (butylated hydroxytoluene, 2,6-di-tert-butyl-4-methylphenol) as radical scavengers led to only a slight decrease in the reaction rate (Supplementary Materials, Part 6). Furthermore, no adducts with TEMPO were detected by NMR analysis. These results indicate the absence of long-lived radical intermediates in the reaction course (and no formation of mono-radical species). Third, the cross-reaction experiment (Supplementary Materials, Part 7) between substrates **1n** and **3e** confirmed the intramolecular nature of the transformation (Scheme 6).

Based on the above observations, we propose two reaction pathways, as shown in Scheme 6. Both mechanisms involve excitation of the substrate to a biradical-like excited state (intermediate **i1**, [44] Scheme 6), which then undergoes transformation through alternative routes. The first preferred pathway (Path 1) involves 1,6-hydrogen atom transfer (1,6-HAT [34]) from the aldehyde to the ester oxygen, yielding intermediate **i2** containing acyl and ketyl radical fragments. Radical recombination leads to the formation of the target benzofuranone **2a**. The less favorable second pathway (Path 2) proceeds through initial C-C bond formation followed by 1,3-HAT. The first pathway is favored, as evidenced by the well-documented high reactivity of aldehydes as hydrogen atom donors in HAT processes [34]. Furthermore, 1,6-HAT is a common and well-established process, whereas 1,3-HAT is very rare [45]. Thus, the generation of the acyl radical by 1,6-HAT in this photoprocess is probably the key to the umpolung reactivity of the aldehyde group. Another possible reaction mechanism could involve a Paternò–Büchi-type [2+2] cycloaddition between the C=O bond of the aldehyde and the C=C bond of the enol, formed via acyl group isomerization. However, the successful phototransformation of **3h** aldehyde with a pivaloyl moiety to the corresponding product **4h** excludes this pathway.

NMR analysis of the crude reaction mixtures suggested the occurrence of side processes for unsuccessful substrates **3i**–**p**. Typically, complex mixtures of unidentifiable compounds were obtained instead of the target products. For aryl-substituted substrates **3k**–**o**, traces of salicylaldehyde and the corresponding carboxylic acids were detected in these crude mixtures (Scheme 6). We propose that these substrates’ degradation occurs via photoinduced C-O bond cleavage, as described for similar compounds [46]. Analogically, the photodegradation of **3j** and **3i** may be related to the increased stability of the cyclopentyl radical [47]. A similar hypothetical pathway for photodegradation of substrates **2b**, **2c**, **2e**, and **2h** with a substituent at the 5-position may involve elimination of the acyl fragment to form quinone methides [48]. The decrease in the yields of products **2f**, **2g**, and **2l** was also accompanied by the formation of complex mixtures. The corresponding substituents (Ph, F, OMe) are likely to contribute to more effective electron delocalization and reactivity in side processes. Apparently, the photochemical “meta-effect” [49,50] plays a role in the HAT efficiency. Thus, the introduction of an electron-withdrawing group at the meta position to the aldehyde group (position 5, Scheme 3) leads to more efficient formation of product **2n** compared to products **2k** and **2l** containing electron-donating groups at similar meta positions (3 and 5, Scheme 3). Similarly, product **2m** is formed more efficiently than **2k** and **2l**, since the alkoxy electron-donating group is presented in the para position [51].

## 3. Materials and Methods

### 3.1. Materials

Commercially available reagents were used without additional purification. E. Merck Kieselgel 60 (Darmstadt, Germany) was used for column chromatography.

Thin-layer chromatography (TLC) was performed on silica gel 60 F254 glass-backed plates (MERCK, Darmstadt, Germany). Visualization was performed using UV light (254 or 312 nm) or staining with KMnO_4_.

NMR spectra were recorded on a Bruker Avance III 800 (with a 5 mm CPTXI cryoprobe) and Bruker Fourier 300 (Billerica, MA, USA). Chemical shifts were reported relative to residue peaks of DMSO-*d*_6_ (2.51 ppm: for ^1^H and 39.5 ppm: for ^13^C).

Melting points were measured on an SMP 30 apparatus without correction.

High-resolution mass spectra (HRMS) were recorded on AB Sciex TripleTOF^®^ 5600+ system (Framingham, MA, USA) using electrospray ionization (ESI). The measurements were performed in positive ion mode (interface capillary voltage—5500 V); mass range was from *m*/*z* 50 to *m*/*z* 3000; external or internal calibration was carried out with ESI Tuning Mix, Agilent (Santa Clara, CA, USA). A syringe injection was used for solutions in acetonitrile, methanol, or water (flow rate 20 μL/min). Nitrogen was applied as a dry gas; interface temperature was set at 180 °C. IUPAC compound names were generated using ChemDraw 12.0 Software.

Photoinduced processes were performed on Evoluchem™ PhotoRedOx box (HepatoChem, Beverly, MA, USA). A 365 nm (LG, HCK1012-01-006, 25 mW/cm^2^), 405 nm (LG, HCK1012-01-010, 28 mW/cm^2^), and 450 nm (CREE-XPE, HCK1012-01-002, 20 mW/cm^2^) light-emitting diode (LED) lamp from Evoluchem™ was used. This device is equipped with a fan to maintain room temperature during the irradiation process.

Optical properties of compound **1a** were investigated using a 10 µM solution in dry DMSO on a Cary 100 Bio spectrophotometer (Varian, Palo Alto, CA, USA).

Data processing was performed using OriginPro 8.6 software (https://www.originlab.com/).

### 3.2. Synthesis of the Starting Compounds ***1*** and ***3***

#### 3.2.1. Synthesis of the Starting Compound **1**

A mixture of the corresponding 2-hydroxybenzaldehyde (10 mmol), acetyl chloride (13 mmol), and K_2_CO_3_ (2.76 g, 20 mmol), in freshly distilled CH_3_CN (50 mL) was heated at 65 °C for 10 h. Next, the reaction mixtures were dissolved in 200 mL of EtOAc, washed with saturated KCl solution (3 × 50 mL), and dried over Na_2_SO_4_. All volatiles were removed in vacuo, and the residue was purified with flash chromatography (eluent: mixture of hexane and EtOAc, *v*/*v* 50:1).

*2-Formylphenyl acetate* (**1a**). Yield, 1.36 g (83%). ^1^H NMR (800 MHz, DMSO-*d*_6_) δ, ppm: 10.08 (1H, s), 7.91 (1H, dd, *J* = 7.6, 1.6 Hz), 7.76 (1H, td, *J* = 7.7, 1.6 Hz), 7.50 (1H, t, *J* = 7.5 Hz), 7.31 (1H, d, *J* = 8.1 Hz), 2.35 (3H, s). The spectral properties corresponded to the literature data [52].
*2-Formyl-4-methylphenyl acetate* (**1b**). Yield, 1.60 g (90%). ^1^H NMR (800 MHz, DMSO-*d*_6_) δ, ppm: 10.04 (1H, s), 7.70 (1H, d, *J* = 1.7 Hz), 7.55 (1H, dd, *J* = 8.2, 1.7 Hz), 7.18 (1H, d, *J* = 8.2 Hz), 2.38 (3H, s), 2.33 (3H, s). The spectral properties corresponded to the literature data [53].
*4-Ethyl-2-formylphenyl acetate* (**1c**). Yield, 1.84 g (96%); yellowish viscous oil. ^1^H NMR (800 MHz, DMSO-*d*_6_) δ, ppm: 10.05 (1H, s), 7.73 (1H, d, *J* = 2.0 Hz), 7.59 (1H, dd, *J* = 8.2, 2.0 Hz), 7.21 (1H, d, *J* = 8.2 Hz), 2.69 (2H, q, *J* = 7.6 Hz), 2.33 (3H, s), 1.21 (3H, t, *J* = 7.6 Hz). ^13^C NMR (75 MHz, DMSO-*d*_6_) δ, ppm: 190.0, 169.4, 149.2, 142.2, 135.0, 129.7, 127.6, 123.6, 27.3, 20.6, 15.3. HRMS (ESI) *m*/*z*: 193.0854 found (calcd for C_11_H_13_O_3_^+^, [M + H]^+^ 193.0859).
*5-(Tert-butyl)-2-formylphenyl acetate* (**1d**). Yield, 2.14 g (97%); yellowish viscous oil. ^1^H NMR (800 MHz, DMSO-*d*_6_) δ, ppm: 10.00 (1H, s), 7.84 (1H, d, *J* = 8.1 Hz), 7.52–7.53 (1H, m), 7.28 (1H, d, *J* = 1.8 Hz), 2.34 (3H, s), 1.30 (9H, s). ^13^C NMR (75 MHz, DMSO-*d*_6_) δ, ppm: 189.5, 169.3, 159.6, 151.0, 130.9, 125.6, 123.5, 120.5, 35.2, 30.5, 20.7. HRMS (ESI) *m*/*z*: 221.1171 found (calcd for C_13_H_17_O_3_^+^, [M + H]^+^ 221.1172).
*2,4-Di-tert-butyl-6-formylphenyl acetate* (**1e**). Yield, 2.24 g (81%). ^1^H NMR (800 MHz, DMSO-*d*_6_) δ, ppm: 9.92 (1H, s), 7.82 (1H, d, *J* = 2.4 Hz), 7.71 (1H, d, *J* = 2.4 Hz), 2.38 (3H, s), 1.34 (18H, m). The spectral properties corresponded to the literature data [54].
*3-Formyl-[1,1′-biphenyl]-4-yl acetate* (**1f**). Yield, 2.28 g (95%); white solid; m.p., 118–120 °C. ^1^H NMR (800 MHz, DMSO-*d*_6_) δ, ppm: 10.14 (1H, s), 8.18 (1H, d, *J* = 2.0 Hz), 8.04 (1H, dd, *J* = 8.3, 2.0 Hz), 7.74 (2H, d, *J* = 7.5 Hz), 7.51 (2H, t, *J* = 7.5 Hz), 7.43 (1H, t, *J* = 7.3 Hz), 7.40 (1H, d, *J* = 8.3 Hz), 2.37 (3H, s). ^13^C NMR (75 MHz, DMSO-*d*_6_) δ, ppm: 190.2, 169.2, 150.2, 138.5, 138.2, 133.5, 129.4, 129.1, 128.2, 128.0, 126.7, 124.4, 20.7. HRMS (ESI) *m*/*z*: 241.0860 found (calcd for C_15_H_13_O_3_^+^, [M + H]^+^ 241.0859).
*4-Fluoro-2-formylphenyl acetate* (**1g**). Yield, 1.70 g (93%). ^1^H NMR (800 MHz, DMSO-*d*_6_) δ, ppm: 10.05 (1H, d, *J* = 1.9 Hz), 7.68 (1H, dd, *J* = 8.4, 3.2 Hz), 7.63 (1H, td, *J* = 8.4, 3.2 Hz), 7.39 (1H, dd, *J* = 8.9, 4.5 Hz), 2.35 (3H, s). The spectral properties corresponded to the literature data [55].
*4-Chloro-2-formylphenyl acetate* (**1h**). Yield, 1.95 g (97%). ^1^H NMR (800 MHz, DMSO-*d*_6_) δ, ppm: 10.04 (1H, s), 7.92 (1H, d, *J* = 2.6 Hz), 7.82 (1H, dd, *J* = 8.6, 2.6 Hz), 7.38 (1H, d, *J* = 8.6 Hz), 2.35 (3H, s). The spectral properties corresponded to the literature data [56].
*3-Chloro-2-formylphenyl acetate* (**1i**). Yield, 1.57g (79%), yellowish viscous oil. ^1^H NMR (800 MHz, DMSO-*d*_6_) δ, ppm: 10.30 (1H, s), 7.72 (1H, t, *J* = 8.1 Hz), 7.57 (1H, dd, *J* = 8.0, 0.8 Hz), 7.28 (1H, d, *J* = 8.0 Hz), 2.31 (3H, s). ^13^C NMR (75 MHz, DMSO-*d*_6_) δ, ppm: 188.6, 168.9, 150.8, 136.6, 135.6, 128.6, 124.8, 123.4, 20.6. HRMS (ESI) *m*/*z*: 199.0156 found (calcd for C_9_H_8_ClO_3_^+^, [M + H]^+^ 199.0156).
*5-Bromo-2-formylphenyl acetate* (**1j**). Yield, 2.16 g (89%). ^1^H NMR (800 MHz, DMSO-*d*_6_) δ, ppm: 10.06 (1H, s), 7.84 (1H, d, *J* = 8.3 Hz), 7.72 (1H, d, *J* = 8.3 Hz), 7.67 (1H, d, *J* = 1.1 Hz), 2.35 (3H, s). The spectral properties corresponded to the literature data [57].
*2-Formyl-4-methoxyphenyl acetate* (**1k**). Yield, 1.57 g (81%). ^1^H NMR (800 MHz, DMSO-*d*_6_) δ, ppm: 10.04 (1H, s), 7.38 (1H, d, *J* = 3.2 Hz), 7.30–7.31 (1H, m), 7.23 (1H, d, *J* = 8.8 Hz), 3.83 (3H, s), 2.33 (3H, s). The spectral properties corresponded to the literature data [58].
*2-Formyl-6-methoxyphenyl acetate* (**1l**). Yield, 1.55 g (80%). ^1^H NMR (800 MHz, DMSO-*d*_6_) δ, ppm: 10.10 (1H, s), 7.47–7.50 (1H, m), 7.44 (2H, d, *J* = 4.8 Hz), 3.84 (3H, s), 2.35 (3H, s). The spectral properties corresponded to the literature data [59].
*5-Ethoxy-2-formylphenyl acetate* (**1m**). Yield, 1.92 g (92%); yellow solid; m.p., 58–60 °C. ^1^H NMR (800 MHz, DMSO-*d*_6_) δ, ppm: 9.90 (1H, s), 7.84 (1H, d, *J* = 8.6 Hz), 7.02 (1H, dd, *J* = 8.6, 2.2 Hz), 6.85 (1H, d, *J* = 2.2 Hz), 4.15 (2H, q, *J* = 6.9 Hz), 2.33 (3H, s), 1.35 (3H, t, *J* = 6.9 Hz). ^13^C NMR (201 MHz, DMSO-*d*_6_) δ, ppm: 188.3, 168.9, 164.1, 152.9, 132.9, 121.3, 112.7, 109.4, 64.2, 20.6, 14.3. HRMS (ESI) *m*/*z*: 209.0808 found (calcd for C_11_H_13_O_4_^+^, [M + H]^+^ 209.0808).
*Methyl 4-acetoxy-3-formylbenzoate* (**1n**). Yield, 1.89 g (85%); yellow solid; m.p., 88–90 °C. ^1^H NMR (800 MHz, DMSO-*d*_6_) δ, ppm: 10.14 (1H, s), 8.45 (1H, d, *J* = 1.2 Hz), 8.28 (1H, dd, *J* = 8.4, 1.2 Hz), 7.49 (1H, d, *J* = 8.4 Hz), 3.90 (3H, s), 2.38 (3H, s). ^13^C NMR (75 MHz, DMSO-*d*_6_) δ, ppm: 189.6, 168.8, 164.8, 154.3, 135.8, 131.7, 128.0, 127.9, 124.6, 52.5, 20.7. HRMS (ESI) *m*/*z*: 223.0602 found (calcd for C_11_H_11_O_5_^+^, [M + H]^+^ 223.0601).

#### 3.2.2. Synthesis of the Starting Compounds **3a**–**3d**, **3h**–**3l**, and **3p**

Corresponding 2-hydroxybenzaldehyde (10 mmol) and Et_3_N (15 mmol) were dissolved in freshly distilled CH_2_Cl_2_ (50 mL) and cooled to 0 °C. The corresponding acyl chloride (15 mmol) was added dropwise, and the resulted mixture was stirred at room temperature overnight. Next, the reaction mixtures were dissolved in 200 mL of CH_2_Cl_2_, washed with saturated KCl solution (3 × 50 mL), and dried over Na_2_SO_4_. All volatiles were removed in vacuo, and the residue was purified with flash chromatography (eluent: mixture of hexane and EtOAc, *v*/*v* 20:1).

*2-Formylphenyl 2-methoxyacetate* (**3a**). Yield, 1.16 g (60%); white oil. ^1^H NMR (800 MHz, DMSO-*d*_6_) δ, ppm: 10.08 (1H, s), 7.94 (1H, dd, *J* = 7.6, 1.7 Hz), 7.76–7.79 (1H, m), 7.50–7.55 (1H, m), 7.35 (1H, d, *J* = 8.1 Hz), 4.45 (2H, s), 3.42 (3H, s). ^13^C NMR (75 MHz, DMSO-*d*_6_) δ, ppm: 190.1, 169.0, 150.3, 135.8, 131.5, 127.8, 126.9, 123.7, 68.8, 58.7. HRMS (ESI) *m*/*z*: 195.0652 found (calcd for C_10_H_11_O_4_^+^, [M + H]^+^ 195.0652).
*2-Formylphenyl cyclohexanecarboxylate* (**3b**). Yield, 1.59 g (69%). ^1^H NMR (800 MHz, DMSO-*d*_6_) δ, ppm: 10.04 (1H, s), 7.91 (1H, dd, *J* = 7.7, 1.6 Hz), 7.74–7.77 (1H, m), 7.49 (1H, t, *J* = 7.5 Hz), 7.27 (1H, d, *J* = 8.1 Hz), 2.71 (1H, tt, *J* = 11.1, 3.7 Hz), 1.75 (2H, ddd, *J* = 13.2, 3.8, 3.7 Hz), 1.60–1.67 (2H, m), 1.51–1.59 (2H, m), 1.24–1.37 (4H, m). The spectral properties corresponded to the literature data [60].
*2-Formylphenyl 2-(neopentyloxy)acetate* (**3c**). Yield, 1.63 g (65%); white oil. ^1^H NMR (800 MHz, DMSO-*d*_6_) δ, ppm: 10.08 (1H, s), 7.93 (1H, dd, *J* = 7.6, 1.5 Hz), 7.77 (1H, td, *J* = 7.7, 1.4 Hz), 7.52 (1H, t, *J* = 7.5 Hz), 7.35 (1H, d, *J* = 8.1 Hz), 4.51 (2H, s), 3.28 (2H, s), 0.91 (9H, s). ^13^C NMR (75 MHz, DMSO-*d*_6_) δ, ppm: 190.1, 169.3, 150.5, 135.7, 131.2, 127.9, 126.8, 123.7, 81.2, 67.9, 31.8, 26.5. HRMS (ESI) *m*/*z*: 251.1280 found (calcd for C_14_H_19_O_4_^+^, [M + H]^+^ 251.1278).
*2-Formylphenyl 5-phenylpentanoate* (**3d**). Yield, 1.97 g (70%); white oil. ^1^H NMR (800 MHz, DMSO-*d*_6_) δ, ppm: 10.05 (1H, s), 7.91 (1H, dd, *J* = 7.6, 1.6 Hz), 7.75 (1H, td, *J* = 7.7, 1.7 Hz), 7.49 (1H, t, *J* = 7.5 Hz), 7.26–7.31 (3H, m), 7.22 (2H, d, *J* = 7.1 Hz), 7.18 (1H, t, *J* = 7.3 Hz), 2.72 (2H, t, *J* = 6.9 Hz), 2.64 (2H, t, *J* = 6.9 Hz), 1.66–1.73 (4H, m). ^13^C NMR (75 MHz, DMSO-*d*_6_) δ, ppm: 189.9, 171.7, 151.0, 141.9, 135.7, 131.0, 128.3, 128.2, 127.9, 126.6, 125.7, 123.8, 34.8, 33.0, 30.3, 23.7. HRMS (ESI) *m*/*z*: 283.1330 found (calcd for C_18_H_19_O_3_^+^, [M + H]^+^ 283.1329).
*2-Formylphenyl pivalate* (**3h**). Yield, 1.96 g (95%); white oil. ^1^H NMR (300 MHz, DMSO-*d*_6_) δ, ppm: 10.03 (1H, s) 7.92 (1H, dd, *J* = 7.7, 1.5 Hz) 7.76 (1H, td, *J* = 7.7, 1.7 Hz) 7.50 (1H, t, *J* = 7.6 Hz) 7.27 (1H, d, *J* = 8.2 Hz) 1.35 (9H, s). ^13^C NMR (75 MHz, DMSO-*d*_6_) δ, ppm: 189.4, 176.2, 151.3, 135.7, 130.9, 128.1, 126.6, 123.6, 38.8, 26.7. HRMS (ESI) *m*/*z*: 207.1018 found (calcd for C_12_H_15_O_3_^+^, [M + H]^+^ 207.1016).
*2-Formylphenyl cyclopentanecarboxylate* (**3i**). Yield, 2.04 g (88%); white oil. ^1^H NMR (800 MHz, DMSO-*d*_6_) δ, ppm 10.06 (1H, s), 7.91 (1H, dd, *J* = 7.6, 1.4 Hz), 7.74–7.77 (1H, m), 7.49 (1H, t, *J* = 7.5 Hz), 7.28 (1H, d, *J* = 8.0 Hz), 2.69 (2H, d, *J* = 7.4 Hz), 2.26–2.32 (1H, m), 1.83–1.88 (2H, m), 1.61–1.66 (2H, m), 1.52–1.56 (2H, m), 1.24–1.28 (2H, m). ^13^C NMR (75 MHz, DMSO-*d*_6_) δ, ppm: 189.7, 171.3, 151.1, 135.7, 130.8, 128.0, 126.6, 123.7, 35.6, 31.9, 24.6. HRMS (ESI) *m*/*z*: 233.1169 found (calcd for C_14_H_17_O_3_^+^, [M + H]^+^ 233.1172).
*2-Formylphenyl cyclopentanecarboxylate* (**3j**). Yield, 1.42 g (65%). ^1^H NMR (800 MHz, DMSO-*d*_6_) δ, ppm: 10.05 (1H, s), 7.91 (1H, dd, *J* = 7.6, 1.6 Hz), 7.74–7.76 (1H, m), 7.49 (1H, t, *J* = 7.5 Hz), 7.30 (1H, d, *J* = 8.1 Hz), 3.14 (1H, quin, *J* = 7.9 Hz), 1.92–2.02 (4H, m), 1.61–1.70 (4H, m). The spectral properties corresponded to the literature data [61].
*2-Formylphenyl benzoate* (**3k**). Yield, 1.24 g (55%). ^1^H NMR (800 MHz, DMSO-*d*_6_) δ, ppm: 10.10 (1H, s), 8.17 (2H, dd, *J* = 8.3, 1.2 Hz), 7.99 (1H, dd, *J* = 7.7, 1.6 Hz), 7.80–7.83 (1H, m), 7.76–7.79 (1H, m), 7.62–7.65 (2H, m), 7.57 (1H, t, *J* = 7.5 Hz), 7.48 (1H, d, *J* = 8.0 Hz). The spectral properties corresponded to the literature data [62].
*2-Formylphenyl 4-(2-methoxyethoxy)benzoate* (**3l**). Yield, 1.38 g (46%); white oil. ^1^H NMR (800 MHz, DMSO-*d*_6_) δ, ppm: 10.09 (1H, s), 8.11 (2H, m), 7.97 (1H, dd, *J* = 7.7, 1.6 Hz), 7.80 (1H, td, *J* = 7.7, 1.7 Hz), 7.54 (1H, t, *J* = 7.5 Hz), 7.45 (1H, d, *J* = 8.1 Hz), 7.15 (2H, m), 4.23–4.26 (2H, m), 3.70–3.72 (2H, m), 3.33 (3H, s). ^13^C NMR (201 MHz, DMSO-*d*_6_) δ, ppm: 189.5, 164.1, 163.1, 151.2, 135.6, 132.2, 131.0, 128.1, 126.6, 123.9, 120.6, 114.7, 70.1, 67.4, 58.2. HRMS (ESI) *m*/*z*: 301.1071 found (calcd for C_17_H_17_O_5_^+^, [M + H]^+^ 301.1071).
*Ethyl (2-formylphenyl) succinate* (**3p**). Yield, 2.25 g (90%). ^1^H NMR (800 MHz, DMSO-*d*_6_) δ, ppm: 10.09 (1H, s), 7.90 (1H, dd, *J* = 7.7, 1.5 Hz), 7.77 (1H, td, *J* = 7.7, 1.6 Hz), 7.49 (1H, t, *J* = 7.5 Hz), 7.28 (1H, d, *J* = 8.1 Hz), 4.10 (2H, q, *J* = 7.1 Hz), 2.95 (2H, t, *J* = 6.6 Hz), 2.71 (2H, t, *J* = 6.6 Hz), 1.19 (3H, t, *J* = 7.1 Hz). The spectral properties corresponded to the literature data [63].

#### 3.2.3. Synthesis of the Starting Compounds **3e**–**3g** and **3m**–**3o**

A mixture of the corresponding 2-hydroxybenzaldehyde (10 mmol), corresponding acid (10 mmol), 1-ethyl-3-(3-dimethylaminopropyl)carbodiimide (3.10 g, 20 mmol), and 4-dimethylaminopyridine (0.24 g, 2 mmol) in freshly distilled CH_2_Cl_2_ (50 mL) was stirred at room temperature overnight. All volatiles were removed in vacuo, and the residue was purified with flash chromatography (eluent: mixture of hexane and EtOAc, *v*/*v* 20:1).

*2-Formylphenyl isobutyrate* (**3e**). Yield, 1.23 g (64%). ^1^H NMR (800 MHz, DMSO-*d*_6_) δ, ppm: 10.05 (1H, s), 7.92 (1H, dd, *J* = 7.6, 1.6 Hz), 7.74–7.77 (1H, m), 7.50 (1H, t, *J* = 7.5 Hz), 7.29 (1H, d, *J* = 8.1 Hz), 2.92 (1H, spt, *J* = 7.0 Hz), 1.28 (6H, d, *J* = 7.0 Hz). The spectral properties corresponded to the literature data [61].
*Tert-butyl (2-formylphenyl) carbonate* (**3f**). Yield, 1.18 g (58%); white oil. ^1^H NMR (800 MHz, DMSO-*d*_6_) δ, ppm: 10.04 (1H, s), 7.92 (1H, dd, *J* = 7.7, 1.6 Hz), 7.73–7.78 (1H, m), 7.47–7.53 (1H, m), 7.30 (1H, d, *J* = 8.1 Hz), 3.48–3.61 (1H, m), 2.37–2.43 (2H, m), 2.28–2.33 (2H, m), 1.99–2.04 (1H, m), 1.88–1.94 (1H, m). ^13^C NMR (75 MHz, DMSO-*d*_6_) δ, ppm: 189.8, 173.1, 151.0, 135.6, 131.2, 128.0, 126.6, 123.7, 37.1, 24.6, 17.8. HRMS (ESI) *m*/*z*: 205.0854 found (calcd for C_12_H_13_O_3_^+^, [M + H]^+^ 205.0859).
*Bis(2-formylphenyl) glutarate* (**3g**). Yield, 1.36 g (40%). ^1^H NMR (800 MHz, DMSO-*d*_6_) δ, ppm: 10.09 (2H, s), 7.93 (2H, dd, *J* = 7.7, 1.7 Hz), 7.76 (2H, td, *J* = 7.7, 1.7 Hz), 7.51 (2H, td, *J* = 7.5, 0.5 Hz), 7.30–7.36 (2H, m), 2.86 (4H, t, *J* = 7.4 Hz), 2.07 (2H, quin, *J* = 7.4 Hz). The spectral properties corresponded to the literature data [64].
*2-Formylphenyl nicotinate* (**3m**). Yield, 1.10 g (48%). ^1^H NMR (800 MHz, DMSO-*d*_6_) δ, ppm: 10.12 (1H, s), 9.27–9.31 (1H, m), 8.93 (1H, dd, *J* = 4.8, 1.6 Hz), 8.50 (1H, dt, *J* = 7.9, 1.9 Hz), 8.01 (1H, dd, *J* = 7.7, 1.6 Hz), 7.83 (1H, td, *J* = 7.7, 1.7 Hz), 7.67–7.69 (1H, m), 7.59 (1H, t, *J* = 7.5 Hz), 7.52 (1H, d, *J* = 8.1 Hz). The spectral properties corresponded to the literature data [65].
*2-Formylphenyl isonicotinate* (**3n**). Yield, 1.25 g (55%). ^1^H NMR (800 MHz, DMSO-*d*_6_) δ, ppm: 10.10 (1H, s), 8.91 (2H, dd, *J* = 4.6, 1.3 Hz), 8.04 (2H, dd, *J* = 4.4, 1.6 Hz), 8.02 (1H, dd, *J* = 7.7, 1.6 Hz), 7.83 (1H, td, *J* = 7.8, 1.7 Hz), 7.60 (1H, t, *J* = 7.5 Hz), 7.52 (1H, d, *J* = 8.0 Hz). The spectral properties corresponded to the literature data [66].
*2-Formylphenyl furan-2-carboxylate* (**3o**). Yield, 1.12 g (52%). ^1^H NMR (800 MHz, DMSO-*d*_6_) δ, ppm: 10.10 (1H, s), 8.06–8.18 (1H, m), 7.98 (1H, dd, *J* = 7.6, 1.6 Hz), 7.80 (1H, td, *J* = 7.8, 1.7 Hz), 7.63 (1H, d, *J* = 3.5 Hz), 7.56 (1H, t, *J* = 7.5 Hz), 7.47 (1H, d, *J* = 8.1 Hz), 6.83 (1H, dd, *J* = 3.5, 1.7 Hz). The spectral properties corresponded to the literature data [65].

### 3.3. Photoinduced Synthesis of the 2-Hydroxy-Benzofuran-3(2H)-Ones ***2*** and ***4***

The corresponding starting compound **1** or **3** (1.0 mmol) was dissolved in freshly distilled DMSO (20 mL) in a Schlenk vessel. The mixtures were degassed under vacuum and filled with argon three times. The solvent screening demonstrated that wet DMSO can be used, and the reaction can be performed in open air. However, given the potential for oxidation of the starting aldehydes to corresponding 2-acyloxybenzoic acids and the fact that screening was performed only on aldehyde **1a**, carrying out the reaction under inert conditions seems preferable. Obtained solutions were irradiated with 365 nm LED lamp in Evoluchem™ PhotoRedOx box with stirring. The process was carried out strictly with two samples at a time. This approach allowed us to claim approximately identical irradiation conditions for all samples, since the Schlenk vessels were installed symmetrically into the reactor each time. It is very important that during the entire irradiation process, the internal ventilation cooling system of the reactor must be turned on to maintain the room temperature of the reaction mixture. The progress of the reaction was monitored by TLC and ^1^H NMR. After the completion of the reaction, reaction mixtures were dissolved in 200 mL of EtOAc, washed with saturated KCl solution (10 × 30 mL), and dried over Na_2_SO_4_. All volatiles were removed in vacuo, and the residue was purified with flash chromatography (eluent: mixture of hexane and EtOAc, *v*/*v* 5:1).

*2-Hydroxy-2-methylbenzofuran-3(2H)-one* (**2a**). Yield, 162 mg (99%). Reaction time, ~12 h. ^1^H NMR (800 MHz, DMSO-*d*_6_) δ, ppm: 7.72–7.74 (1H, m), 7.69 (1H, s), 7.62 (1H, dd, *J* = 7.6, 0.8 Hz), 7.14 (1H, d, *J* = 8.3 Hz), 7.12 (1H, t, *J* = 7.4 Hz), 1.45 (3H, s). The spectral properties corresponded to the literature data [39].
*2-Hydroxy-2,5-dimethylbenzofuran-3(2H)-one* (**2b**). Yield, 134 mg (75%); yellow solid; m.p., 100–102 °C. Reaction time, ~48 h. ^1^H NMR (800 MHz, DMSO-*d*_6_) δ, ppm m: 7.63 (1H, s), 7.55 (1H, dd, *J* = 8.4, 1.6 Hz), 7.41 (1H, s), 7.04 (1H, d, *J* = 8.4 Hz), 2.30 (3H, s), 1.43 (3H, s). ^13^C NMR (201 MHz, DMSO-*d*_6_) δ, ppm: 199.2, 167.8, 140.0, 131.0, 123.8, 118.3, 112.9, 104.3, 21.8, 20.0. HRMS (ESI) *m*/*z*: 179.0704 found (calcd for C_10_H_11_O_3_^+^, [M + H]^+^ 179.0703).
*5-Ethyl-2-hydroxy-2-methylbenzofuran-3(2H)-one* (**2c**). Yield, 134 mg (70%). Reaction time, ~48 h. ^1^H NMR (800 MHz, DMSO-*d*_6_) δ, ppm: 7.62 (1H, s), 7.60 (1H, dd, *J* = 8.5, 2.0 Hz), 7.43 (1H, d, *J* = 1.4 Hz), 7.06 (1H, d, *J* = 8.4 Hz), 2.61 (2H, q, *J* = 7.6 Hz), 1.44 (3H, s), 1.17 (3H, t, *J* = 7.6 Hz). The spectral properties corresponded to the literature data [39].
*6-(Tert-butyl)-2-hydroxy-2-methylbenzofuran-3(2H)-one* (**2d**). Yield, 152 mg (69%); white solid; m.p., 48–50 °C. Reaction time, ~24 h. ^1^H NMR (800 MHz, DMSO-*d*_6_) δ, ppm: 7.60 (1H, s), 7.54 (1H, d, *J* = 8.1 Hz), 7.19 (1H, d, *J* = 8.1 Hz), 7.11 (1H, s), 1.44 (3H, s), 1.30 (10H, s). ^13^C NMR (75 MHz, DMSO-*d*_6_) δ, ppm: 198.5, 169.9, 163.5, 124.1, 119.6, 116.0, 109.7, 104.4, 35.7, 30.6, 21.7. HRMS (ESI) *m*/*z*: 221.1172 found (calcd for C_13_H_17_O_3_^+^, [M + H]^+^ 221.1172).
*5,7-Di-tert-butyl-2-hydroxy-2-methylbenzofuran-3(2H)-one* (**2e**). Yield, 180 mg (65%); yellow solid; m.p., 138–140 °C. Reaction time, ~48 h. ^1^H NMR (800 MHz, DMSO-*d*_6_) δ, ppm: 7.61 (1H, d, *J* = 2.1 Hz), 7.60 (1H, s), 7.37 (1H, d, *J* = 2.1 Hz), 1.45 (3H, s), 1.38 (9H, s), 1.29 (9H, s). ^13^C NMR (75 MHz, DMSO-*d*_6_) δ, ppm: 199.9, 166.4, 144.0, 134.7, 132.3, 118.3, 117.5, 103.8, 34.3, 34.0, 31.1, 28.9, 21.8. HRMS (ESI) *m*/*z*: 277.1803 found (calcd for C_17_H_25_O_3_^+^, [M + H]^+^ 277.1798).
*2-Hydroxy-2-methyl-5-phenylbenzofuran-3(2H)-one* (**2f**). Yield, 163 mg (68%); yellow solid; m.p., 121–123 °C. Reaction time, ~24 h. ^1^H NMR (800 MHz, DMSO-*d*_6_) δ, ppm: 8.06 (1H, dd, *J* = 8.6, 2.0 Hz), 7.85 (1H, d, *J* = 2.0 Hz), 7.78 (1H, s), 7.66–7.70 (2H, m), 7.46 (2H, t, *J* = 7.7 Hz), 7.35–7.39 (1H, m), 7.25 (1H, d, *J* = 8.6 Hz), 1.50 (3H, s). ^13^C NMR (201 MHz, DMSO-*d*_6_) δ, ppm: 199.1, 168.9, 138.8, 137.8, 134.2, 129.0, 127.4, 126.5, 121.9, 119.1, 113.8, 104.9, 21.7. HRMS (ESI) *m*/*z*: 241.0859 found (calcd for C_15_H_13_O_3_^+^, [M + H]^+^ 241.0859).
*5-Fluoro-2-hydroxy-2-methylbenzofuran-3(2H)-one* (**2g**). Yield, 120 mg (66%). Reaction time, ~24 h. ^1^H NMR (800 MHz, DMSO-*d*_6_) δ, ppm: 7.78 (1H, s), 7.63 (1H, td, *J* = 9.0, 2.9 Hz), 7.47 (1H, dd, *J* = 7.0, 2.9 Hz), 7.20 (1H, dd, *J* = 9.0, 3.7 Hz), 1.46 (3H, s). The spectral properties corresponded to the literature data [39].
*5-Chloro-2-hydroxy-2-methylbenzofuran-3(2H)-one* (**2h**). Yield, 147 mg (74%); yellow solid; m.p., 90–92 °C. Reaction time, ~48 h. ^1^H NMR (800 MHz, DMSO-*d*_6_) δ, ppm: 7.84 (1H, s), 7.77 (1H, dd, *J* = 8.8, 2.4 Hz), 7.69 (1H, d, *J* = 2.4 Hz), 7.21 (1H, d, *J* = 8.8 Hz), 1.47 (3H, s). ^13^C NMR (201 MHz, DMSO-*d*_6_) δ, ppm: 198.3, 167.9, 138.6, 125.8, 123.8, 119.9, 115.3, 105.3, 21.6. HRMS (ESI) *m*/*z*: 199.0154 found (calcd for C_9_H_8_ClO_3_^+^, [M + H]^+^ 199.0156).
*4-Chloro-2-hydroxy-2-methylbenzofuran-3(2H)-one* (**2i**). Yield, 159 mg (80%); yellow solid; m.p., 80–82 °C. Reaction time, ~24 h. ^1^H NMR (800 MHz, DMSO-*d*_6_) δ, ppm: 7.82 (1H, s), 7.70 (1H, t, *J* = 8.1 Hz), 7.14 (1H, d, *J* = 7.8 Hz), 7.12 (1H, d, *J* = 8.3 Hz), 1.47 (3H, s). ^13^C NMR (201 MHz, DMSO-*d*_6_) δ, ppm: 196.4, 170.2, 139.6, 130.5, 122.5, 115.5, 112.1, 104.7, 21.6. HRMS (ESI) *m*/*z*: 199.0157 found (calcd for C_9_H_8_ClO_3_^+^, [M + H]^+^ 199.0156).
*6-Bromo-2-hydroxy-2-methylbenzofuran-3(2H)-one* (**2j**). Yield, 190 mg (78%); white solid; m.p., 96–98 °C. Reaction time, ~24 h. ^1^H NMR (700 MHz, DMSO-*d*_6_) δ, ppm: 7.85 (1H, s), 7.57 (1H, dd, *J* = 8.1, 0.3 Hz), 7.50 (1H, d, *J* = 1.3 Hz), 7.30 (1H, dd, *J* = 8.1, 1.3 Hz), 1.47 (3H, s). ^13^C NMR (75 MHz, DMSO-*d*_6_) δ, ppm: 198.1, 169.6, 132.6, 126.1, 125.2, 117.9, 116.5, 105.3, 21.6. HRMS (ESI) *m*/*z*: 242.9648 found (calcd for C_9_H_8_BrO_3_^+^, [M + H]^+^ 242.9651).
*2-Hydroxy-5-methoxy-2-methylbenzofuran-3(2H)-one* (**2k**). Yield, 165 mg (85%); light green solid; m.p., 100–102 °C. Reaction time, ~48 h. ^1^H NMR (800 MHz, DMSO-*d*_6_) δ, ppm: 7.63 (1H, s), 7.35 (1H, dd, *J* = 8.9, 2.8 Hz), 7.09 (1H, d, *J* = 8.9 Hz), 7.08 (1H, d, *J* = 2.8 Hz), 3.77 (3H, s), 1.43 (3H, s). ^13^C NMR (75 MHz, DMSO-*d*_6_) δ, ppm: 199.5, 164.6, 154.3, 128.1, 118.4, 114.3, 105.2, 104.7, 55.8, 21.8. HRMS (ESI) *m*/*z*: 195.0652 found (calcd for C_10_H_11_O_4_^+^, [M + H]^+^ 195.0652).
*2-Hydroxy-7-methoxy-2-methylbenzofuran-3(2H)-one* (**2l**). Yield, 126 mg (65%); yellow solid; m.p., 100–102 °C. Reaction time, ~48 h. ^1^H NMR (800 MHz, DMSO-*d*_6_) δ, ppm: 7.72 (1H, s), 7.35 (1H, d, *J* = 7.8 Hz), 7.16 (1H, d, *J* = 7.7 Hz), 7.05 (1H, t, *J* = 7.8 Hz), 3.87 (3H, s), 1.45 (3H, s). ^13^C NMR (75 MHz, DMSO-*d*_6_) δ, ppm: 199.4, 159.6, 146.0, 122.2, 119.9, 119.4, 115.3, 104.6, 55.9, 21.7. HRMS (ESI) *m*/*z*: 195.0652 found (calcd for C_10_H_11_O_4_^+^, [M + H]^+^ 195.0652).
*6-Ethoxy-2-hydroxy-2-methylbenzofuran-3(2H)-one* (**2m**). Yield, 194 mg (90%); yellow solid; m.p., 78–80 °C. Reaction time, ~24 h. ^1^H NMR (800 MHz, DMSO-*d*_6_) δ, ppm: 7.63 (1H, s), 7.50 (1H, d, *J* = 8.4 Hz), 6.65 (1H, d, *J* = 1.9 Hz), 6.62–6.65 (1H, m), 4.14 (2H, q, *J* = 7.0 Hz), 1.43 (3H, s), 1.35 (3H, t, *J* = 7.0 Hz). ^13^C NMR (75 MHz, DMSO-*d*_6_) δ, ppm: 196.5, 171.9, 167.8, 125.8, 111.3, 111.2, 105.1, 96.8, 64.3, 21.9, 14.3. HRMS (ESI) *m*/*z*: 209.0813 found (calcd for C_11_H_13_O_4_^+^, [M + H]^+^ 209.0808).
*Methyl 2-hydroxy-2-methyl-3-oxo-2,3-dihydrobenzofuran-5-carboxylate* (**2n**). Yield, 198 mg (89%); yellow solid; m.p., 88–90 °C. Reaction time, ~24 h. ^1^H NMR (800 MHz, DMSO-*d*_6_) δ, ppm: 8.29 (1H, dd, *J* = 8.7, 1.8 Hz), 8.13 (1H, d, *J* = 1.8 Hz), 7.98 (1H, s), 7.28 (1H, d, *J* = 8.7 Hz), 3.86 (3H, s), 1.50 (3H, s). ^13^C NMR (201 MHz, DMSO-*d*_6_) δ, ppm: 198.3, 172.0, 165.1, 139.5, 126.1, 123.3, 118.8, 113.8, 106.0, 52.3, 21.5. HRMS (ESI) *m*/*z*: 223.0597 found (calcd for C_11_H_11_O_5_^+^, [M + H]^+^ 223.0601).
*2-Hydroxy-2-(methoxymethyl)benzofuran-3(2H)-one* (**4a**). Yield, 165 mg (85%); yellow solid; m.p., 98–100 °C. Reaction time, ~24 h. ^1^H NMR (800 MHz, DMSO-*d*_6_) δ, ppm: 7.92 (1H, s), 7.72 (1H, ddd, *J* = 8.4, 7.2, 1.4 Hz), 7.59 (1H, dd, *J* = 7.6, 0.9 Hz), 7.17 (1H, d, *J* = 8.3 Hz), 7.08–7.12 (1H, m), 3.66–3.70 (1H, m), 3.61–3.65 (1H, m), 3.16 (3H, s). ^13^C NMR (201 MHz, DMSO-*d*_6_) δ, ppm: 198.6, 170.5, 138.9, 124.1, 121.7, 119.7, 113.0, 103.6, 73.4, 59.0. HRMS (ESI) *m*/*z*: 195.0650 found (calcd for C_10_H_11_O_4_^+^, [M + H]^+^ 195.0652).
*2-Cyclohexyl-2-hydroxybenzofuran-3(2H)-one* (**4b**). Yield, 139 mg (60%); yellow solid; m.p., 73–75 °C. Reaction time, ~24 h. ^1^H NMR (800 MHz, DMSO-*d*_6_) δ, ppm: 7.70–7.72 (1H, m), 7.58 (1H, dd, *J* = 7.6, 0.8 Hz), 7.56 (1H, s), 7.15 (1H, d, *J* = 8.3 Hz), 7.09 (1H, t, *J* = 7.4 Hz), 1.91 (1H, d, *J* = 12.8 Hz), 1.78–1.81 (1H, m), 1.69–1.72 (1H, m), 1.57–1.63 (2H, m), 1.43 (1H, d, *J* = 13.0 Hz), 1.03–1.17 (5H, m). ^13^C NMR (201 MHz, DMSO-*d*_6_) δ, ppm: 200.1, 170.3, 138.9, 123.9, 121.6, 120.1, 112.9, 106.7, 43.2, 25.8, 25.6, 25.4, 25.2, 25.1. HRMS (ESI) *m*/*z*: 233.1173 found (calcd for C_14_H_17_O_3_^+^, [M + H]^+^ 233.1172).
*2-Hydroxy-2-((neopentyloxy)methyl)benzofuran-3(2H)-one* (**4c**). Yield, 155 mg (62%); white solid; m.p., 63–65 °C. Reaction time, ~12 h. ^1^H NMR (800 MHz, DMSO-*d*_6_) δ, ppm: 7.88 (1H, s), 7.70 (1H, ddd, *J* = 8.3, 7.2, 1.4 Hz), 7.59 (1H, dd, *J* = 7.6, 0.8 Hz), 7.16 (1H, d, *J* = 8.3 Hz), 7.08 (1H, t, *J* = 7.4 Hz), 3.72–3.75 (1H, m), 3.67–3.70 (1H, m), 3.04 (1H, d, *J* = 8.6 Hz), 2.93 (1H, d, *J* = 8.6 Hz), 0.56 (9H, s). ^13^C NMR (201 MHz, DMSO-*d*_6_) δ, ppm: 199.0, 170.5, 138.7, 123.7, 121.5, 120.1, 112.7, 104.1, 81.3, 72.4, 31.6, 26.0. HRMS (ESI) *m*/*z*: 251.1279 found (calcd for C_14_H_19_O_4_^+^, [M + H]^+^ 251.1278).
*2-Hydroxy-2-(4-phenylbutyl)benzofuran-3(2H)-one* (**4d**). Yield, 212 mg (75%); yellow solid; m.p., 78–80 °C. Reaction time, ~24 h. ^1^H NMR (800 MHz, DMSO-*d*_6_) δ, ppm: 7.72 (1H, ddd, *J* = 8.3, 7.2, 1.4 Hz), 7.64 (1H, s), 7.60 (1H, dd, *J* = 7.6, 0.8 Hz), 7.24 (2H, t, *J* = 7.6 Hz), 7.12–7.16 (4H, m), 7.11 (1H, t, *J* = 7.4 Hz), 1.82–1.87 (1H, m), 1.79 (1H, ddd, *J* = 14.0, 11.1, 5.2 Hz), 1.50–1.55 (2H, m), 1.36–1.40 (1H, m), 1.15–1.24 (2H, m), 0.73–0.88 (1H, m). ^13^C NMR (201 MHz, DMSO-*d*_6_) δ, ppm: 199.5, 169.9, 142.0, 139.0, 128.2, 128.1, 125.6, 124.3, 121.7, 119.3, 113.1, 105.6, 35.0, 34.9, 30.9, 21.7. HRMS (ESI) *m*/*z*: 283.1322 found (calcd for C_18_H_19_O_3_^+^, [M + H]^+^ 283.1329).
*2-Hydroxy-2-isopropylbenzofuran-3(2H)-one* (**4e**). Yield, 173 mg (90%); yellow solid; m.p., 58–60 °C. Reaction time, ~24 h. ^1^H NMR (800 MHz, DMSO-*d*_6_) δ, ppm: 7.68–7.74 (1H, m), 7.53–7.64 (2H, m), 7.17 (1H, d, *J* = 8.3 Hz), 7.10 (1H, t, *J* = 7.4 Hz), 2.05–2.12 (1H, m), 0.99 (3H, d, *J* = 6.8 Hz), 0.78 (3H, d, *J* = 6.8 Hz). ^13^C NMR (75 MHz, DMSO-*d*_6_) δ, ppm: 200.1, 170.4, 139.0, 124.1, 121.6, 120.1, 112.9, 107.0, 33.4, 15.9, 15.5. HRMS (ESI) *m*/*z*: 193.0859 found (calcd for C_11_H_13_O_3_^+^, [M + H]^+^ 193.0859).
*2-Cyclobutyl-2-hydroxybenzofuran-3(2H)-one* (**4f**). Yield, 198 mg (97%); yellow solid; m.p., 62–64 °C. Reaction time, ~24 h. ^1^H NMR (800 MHz, DMSO-*d*_6_) δ, ppm: 7.73 (1H, ddd, *J* = 8.4, 7.2, 1.5 Hz), 7.62 (1H, s), 7.59 (1H, dd, *J* = 7.6, 0.9 Hz), 7.19 (1H, d, *J* = 8.3 Hz), 7.05–7.13 (1H, m), 2.67–2.76 (1H, m), 2.09–2.15 (1H, m), 1.94–1.99 (1H, m), 1.80–1.87 (2H, m), 1.70–1.76 (2H, m). ^13^C NMR (75 MHz, DMSO-*d*_6_) δ, ppm: 199.4, 170.1, 139.1, 124.2, 121.7, 119.9, 113.1, 105.7, 22.0, 21.0, 17.5. HRMS (ESI) *m*/*z*: 205.0858 found (calcd for C_12_H_13_O_3_^+^, [M + H]^+^ 205.0859).
*2,2′-(Propane-1,3-diyl)bis(2-hydroxybenzofuran-3(2H)-one)* (**4g**). Yield, 238 mg (70%); white solid; m.p., 128–130 °C. Reaction time, ~24 h. ^1^H NMR (800 MHz, DMSO-*d*_6_) δ, ppm: 7.69–7.73 (2H, m), 7.65 (2H, d, *J* = 2.2 Hz), 7.59 (2H, d, *J* = 7.6 Hz), 7.13 (2H, dd, *J* = 8.3, 3.4 Hz), 7.10 (2H, t, *J* = 7.4 Hz), 1.76–1.81 (2H, m), 1.70–1.75 (2H, m), 1.36–1.41 (1H, m), 1.14–1.26 (1H, m). ^13^C NMR (75 MHz, DMSO-*d*_6_) δ, ppm: 199.4, 169.8, 139.1, 124.4, 121.8, 119.2, 113.2, 105.4, 34.9, 15.5. HRMS (ESI) *m*/*z*: 341.1018 found (calcd for C_19_H_17_O_6_^+^, [M + H]^+^ 341.1020).
*2-(tert-butyl)-2-hydroxybenzofuran-3(2H)-one* (**4h**). Yield, 134 mg (65%); yellow oil. Reaction time, ~24 h. ^1^H NMR (300 MHz, DMSO-*d*_6_) δ, ppm: 7.66–7.75 (1H, m) 7.61–7.56 (2H, m) 7.15 (1H, d, *J* = 8.3 Hz) 7.08 (1H, t, *J* = 7.4 Hz) 0.97 (9H, s). ^13^C NMR (75 MHz, DMSO-*d*_6_) δ, ppm: 200.1, 170.3, 138.9, 124.0, 121.5, 120.5, 112.8, 108.1, 37.3, 23.8. HRMS (ESI) *m*/*z*: 207.1019 found (calcd for C_12_H_15_O_3_^+^, [M + H]^+^ 207.1016).

### 3.4. Synthesis of the Heterocyclic Derivatives ***5*** and ***6***

#### 3.4.1. Synthesis of the Heterocyclic Derivatives **5**

A mixture of the corresponding 2-hydroxybenzofuran-3(2*H*)-one **2b**, **2d**, or **4f** (0.07 mmol) and *o*-phenylenediamine (8 mg, 0.07 mmol) in freshly distilled MeOH (0.5 mL) was stirred at room temperature for 10 min. All volatiles were removed in vacuo, and the residue was purified with flash chromatography (eluent: mixture of hexane and EtOAc, *v*/*v* 10:1).

*4-Methyl-2-(3-methylquinoxalin-2-yl)phenol* (**5b**). Yield, 17 mg (99%); pale yellow solid; m.p., 190–192 °C. ^1^H NMR (300 MHz, DMSO-*d*_6_) δ, ppm: 9.63 (1H, s), 7.96–8.10 (2H, m), 7.71–7.87 (2H, m), 7.07–7.18 (2H, m), 6.88 (1H, d, *J* = 8.1 Hz), 2.57 (3H, s), 2.27 (3H, s). ^13^C NMR (75 MHz, DMSO-*d*_6_) δ, ppm: 154.1, 154.0, 152.4, 140.6, 140.3, 130.7, 130.7, 129.6, 129.1, 128.7, 128.1, 127.8, 126.1, 115.6, 22.7, 20.0. HRMS (ESI) *m*/*z*: 251.1180 found (calcd for C_16_H_15_N_2_O^+^, [M + H]^+^ 251.1179).
*2-(3-Cyclobutylquinoxalin-2-yl)phenol* (**5d**). Yield, 19 mg (97%); yellow oil. ^1^H NMR (300 MHz, DMSO-*d*_6_) δ, ppm: 9.73 (1H, s), 8.11 (1H, dd, *J* = 7.7, 1.3 Hz), 8.04 (1H, dd, *J* = 7.7, 1.4 Hz), 7.74–7.88 (2H, m), 7.29–7.38 (1H, m), 7.20–7.28 (1H, m), 6.88–7.03 (2H, m), 3.87 (1H, quin, *J* = 8.5 Hz), 2.29–2.45 (2H, m), 1.68–2.06 (4H, m). ^13^C NMR (75 MHz, DMSO-*d*_6_) δ, ppm: 158.7, 154.8, 153.3, 140.7, 140.3, 130.3, 130.1, 129.6, 129.1, 128.7, 128.4, 126.2, 119.0, 115.5, 39.0, 27.2, 17.4. HRMS (ESI) *m*/*z*: 277.1333 found (calcd for C_18_H_17_N_2_O^+^, [M + H]^+^ 277.1335).
*2-(3-(4-Phenylbutyl)quinoxalin-2-yl)phenol* (**5f**). Yield, 23 mg (93%); yellow oil. ^1^H NMR (300 MHz, DMSO-*d*_6_) δ, ppm: 9.81 (1H, s), 7.96–8.13 (2H, m), 7.72–7.88 (2H, m), 7.30–7.40 (1H, m), 7.17–7.30 (3H, m), 7.03–7.17 (3H, m), 6.88–7.03 (2H, m), 2.91 (2H, t, *J* = 7.5 Hz), 2.43–2.49 (2H, m), 1.59–1.74 (2H, m), 1.43–1.57 (2H, m). ^13^C NMR (75 MHz, DMSO-*d*_6_) δ, ppm: 157.1, 154.6, 153.9, 142.0, 140.7, 140.3, 130.5, 130.2, 129.7, 129.1, 128.7, 128.2 (4C), 128.2, 126.3, 125.6, 119.2, 115.6, 34.8, 34.5, 30.6, 27.1. HRMS (ESI) *m*/*z*: 355.1804 found (calcd for C_24_H_23_N_2_O^+^, [M + H]^+^ 355.1805).

#### 3.4.2. Synthesis of the Heterocyclic Derivative 6

A mixture of the 2-hydroxy-2,5-dimethylbenzofuran-3(*2H*)-one (**2b**) (24 mg, 0.14 mmol), 4-bromobenzaldehyde (25 mg, 0.14 mmol), ammonium acetate (21 mg, 0.35 mmol), and H_2_O (0.1 mL) in EtOH (0.4 mL) was stirred at 70 °C for 8 h. All volatiles were removed in vacuo, and the residue was purified with flash chromatography (eluent: mixture of toluene and EtOAc, *v*/*v* 50:1).

*2-(2-(4-Bromophenyl)-5-methyl-1H-imidazol-4-yl)-4-methylphenol* (**6b**). Yield, 30 mg (65%); pale yellow solid; m.p., 230–232 °C. ^1^H NMR (300 MHz, DMSO-*d*_6_) δ, ppm: 12.89 (1H, br. s.), 12.20 (1H, s), 7.85 (2H, m, *J* = 8.5 Hz), 7.71 (2H, m, *J* = 8.5 Hz), 7.30 (1H, s), 6.92 (1H, d, *J* = 7.9 Hz), 6.76 (1H, d, *J* = 8.2 Hz), 2.54 (3H, s), 2.26 (3H, s). ^13^C NMR (75 MHz, DMSO-*d*_6_) δ, ppm: 153.5, 140.5, 134.9, 132, 128.3, 128.0, 127.0, 126.5, 126.0, 124.7, 121.5, 118.1, 116.3, 20.4, 12.0. HRMS (ESI) *m*/*z*: 329.0280 found (calcd for C_16_H_14_BrN_2_O^+^, [M + H]^+^ 329.0284).

## 4. Conclusions

In this paper, we present a non-catalytic and atom-economical photochemical transformation of 2-acyloxybenzaldehydes to 2-hydroxybenzofuranones under 365 nm irradiation. This approach represents the carbonyl umpolung, which likely proceeds through generation and recombination of acyl and ketyl radicals. The resulting structural motif is widespread in natural compounds with various bioactivities. The proposed method utilizes readily available substrates and mild reaction conditions for the synthesis of masked 1,2-dicarbonyl compounds. A representative group of 2-acyloxybenzaldehydes with various substituents in both the aromatic ring and the acyl moiety was converted to 2-hydroxybenzofuranones with yields of 60–99%. A notable yield decrease is shown for 2-acyloxybenzaldehydes with substituents in the fifth position of the cycle. The approach cannot be utilized for derivatives containing benzoyl or aromatic heterocyclic groups. The synthetic utility of the 2-hydroxybenzofuranones in subsequent chemical transformations was briefly studied. These compounds were converted into quinoxaline and imidazole derivatives in good yields.

## Figures and Tables

**Table 1:** Solvents’ screening results for **1a.** For more details, see Supplementary Materials, Part 1.

No.	Solvent	Remaining 1a, %	2a, %	2-Acetoxybenzoic Acid, %
1	CCl_4_	0	0	90
2	DMSO	<1	99	0
3	DMF	<1	8 *	0
4	DMAC	<1	11 *	0
5	PhMe	9	0 *	0
6	DCM	9	0	67
7	MeOH	2	0 *	0
8	THF	0	0 *	0
9	acetone	0	0	53
10	TEA	0	0 *	0
11	(CF_3_)_2_CHOH	92	0	0
12	CH_3_CN	0	0	76

* Complex reaction by-products.

## Data Availability

All data are contained within the article and Supplementary Materials.

## Supplementary Materials

The following supporting information can be downloaded at: https://www.mdpi.com/article/10.3390/molecules30153080/s1, Table S1: Solvent’s screening results for compound **1a**; Table S2: Wet and open air conditions screening results for compound **1a**; Table S3: Yield of 2a at different wavelengths; Figure S1: The absorption spectrum of the compound **1a**. Irradiation wavelengths is marked with a dashed line; Table S4: Study of photoreactions with different temperature; Figure S2: Photochemical set-up with heating; Figure S3: Photochemical set-up in NMR tubes; Figure S4: Kinetic study of compound **1a**; Figure S5: 1H NMR of kinetic experiment; Figure S6: Kinetic study of compound **1a** phototranformation with BHT and TEMPO. Reference [67] is cited in the Supplementary Materials.
