# Supplementary material for: Photochemically-Enabled Umpolung Conversion of 2-Acyloxybenzaldehydes into 2-Hydroxybenzofuranones
Source: Molecules. 2025 Jul 23;30(15):3080. doi: 10.3390/molecules30153080 (PMC12348330; doi:10.3390/molecules30153080)
Supplement: Supplementary file 1 [file molecules-30-03080-s001.zip › molecules-3773751-supplementary.pdf]

Contents

1. Optimization of solvent S2
2. Study of wet and open air conditions S4
3. Optimization of wavelength S5
4. Temperature study S6
5. Kinetic study S7
6. Experiments with radical quenching reagents S9
7. Cross-reaction experiment S10
8. Copies of ^{1}H and ^{13}C NMR spectra S11

1. Optimization of solvent

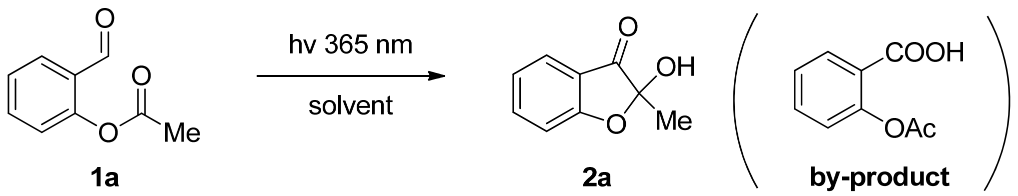

1a (8 mg) was dissolved in freshly distilled solvent (1.0 mL). Vials with obtained solutions were irradiated with 365 nm LED lamp in Evoluchem™ PhotoRedOx box with stirring. After 18 hours of irradiation, solvents were removed in vacuum and the residue were weighted. For solutions in DMSO, DMF, DMAC and NMP reaction mixtures were dissolved in 10 mL of EtOAc and washed with saturated KCl solution (20×3 mL). Next, organic solutions were dried over Na_2SO_4, evaporated and weighted. Finally, all residues were analyzed by ^1H NMR in DMSO-d_6. The overall aromatic signal from 7.00 ppm to 8.00 ppm (4H) was used as a standard (400%). The quantity of components was assessed using the following signals:

- singlet from 10.07 ppm to 10.12 ppm (1H) for substrate **1a**;
- singlet from 1.40 ppm to 1.53 ppm (3H) for product **2a**;
- doublet from 7.17 ppm to 7.21 ppm (1H)[1] for by-product 2-acetoxybenzoic acid.

Results are presented in Table S1.

[1] According to the data from Wu, X.A.; Ying, P .; Liu, J.Y.; Shen, H.S.; Chen, Y.; He, L. Lithium Chloride–Assisted Selective Hydrolysis of Methyl Esters Under Microwave Irradiation. Synth. Commun. 2009, 39, 3459–3470. https://doi.org/10.1080/00397910902778001.

Table S1. Solvent's screening results for **1a**.

№	Solvent	Remained **1a**, %	**2a**, %	Comments
1	NMP	<1	0	c.m.*
**2**	**DMSO**	**<1**	**99**	
3	DMF	<1	8	c.m.*
4	DMAC	<1	11	c.m.*
5	PhMe	9	0	c.m.*
6	PhH	7	0	b.-p.** 71%
7	DCM	9	0	b.-p.** 67%
8	$CHCl_3$	0	0	b.-p.** 49%
9	CCl_4	0	0	b.-p.** 90%
10	DCE	0	0	b.-p.** 70%
11	MeOH	2	0	c.m.*
12	EtOH	0	0	c.m.*
13	Et_2O	0	0	c.m.*
14	Dioxane	0	0	c.m.*
15	THF	0	0	c.m.*
16	EtOAc	0	0	b.-p.** 66%
17	acetone	0	0	b.-p.** 53%
18	PhCl	5	0	b.-p.** 56%
19	TEA	0	0	c.m.*
20	DIPEA	0	0	c.m.*
21	Py	0	0	c.m.*
22	$(CF_3)_2CHOH$	92	0	
23	CF_3CH_2OH	10	0	b.-p.** 72%
24	CH_3CN	0	0	b.-p.** 76%

*a complex mixture of a large number of difficult to identify compounds;

**by-product = 2-acetoxybenzoic acid.

2. Study of wet and open air conditions

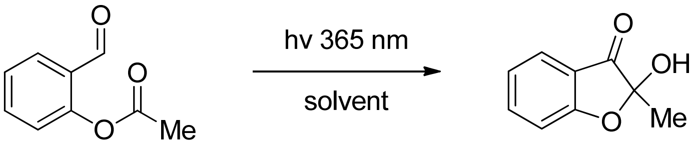

1a **2a**

1a (8 mg) was dissolved in corresponding solvent (1.0 mL). Vials with obtained solutions were irradiated with 365 nm LED lamp in Evoluchem™ PhotoRedOx box with stirring. After 4 or 6 hours of irradiation reaction mixtures were dissolved in 10 mL of EtOAc and washed with saturated KCl solution (20×3 mL). Next, organic solutions were dried over Na_2SO_4, evaporated and weighted. Finally, all residues were analyzed by ^{1}H NMR in DMSO-d_6 (see above in Part 1).

Results are presented in Table S2.

Table S2. Wet and open air conditions screening results for **1a**.

№	Time, h	Solvent	Remained **1a**, %	**2a**, %
1	6	DMSO	28	62
2	6	DMSO wet*	48	45
3	4	DMSO argon**	38	55
4	4	DMSO open air***	39	54

*33 mg H_2O (3% by weight) was added to 1 ml of DMSO;
**DMSO was pre-degassed and saturated with argon three times, the reaction mixture was prepared under argon atmosphere;
***vial without a cap was used.

3. Optimization of wavelength

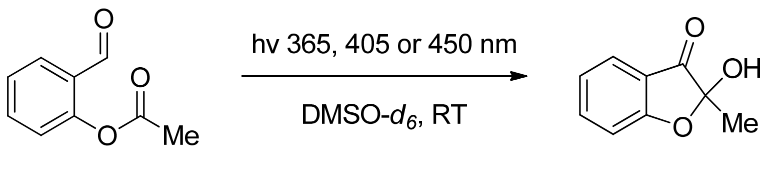

1a **2a**

Compound **1a** (15 mg, 0.09 mmol) was dissolved in 3 mL of DMSO-*d6* in a Schlenk vessel. The mixture was degassed under vacuum and filled with argon three times. Next, 0.65 mL portions of the solution were transferred to three argon fused NMR tube and sealed. The mixtures were analyzed by ^{1}H NMR (initial point). NMR tubes with this solution were irradiated with different LED lamps (365, 405, and 425 nm separately) in Evoluchem™ PhotoRedOx box for an hour. The mixtures were analyzed by ^{1}H NMR (see above in Part 1).

Results are presented in Table S3.

Table S3. Yield of **2a** at different wavelengths.

Wavelength, nm	**2a**, %	Remained **1a**, %
365	67	28
405	6	92
450	0	100

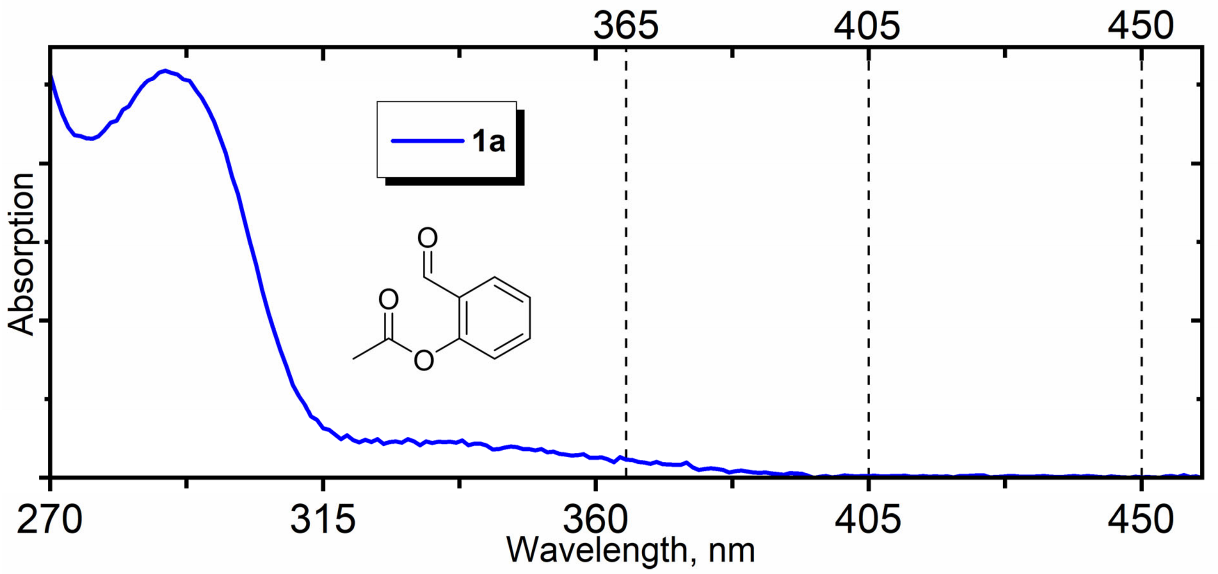

Figure S1. The absorption spectrum of the compound **1a**. Irradiation wavelengths are marked with a dashed line.

4. Temperature study

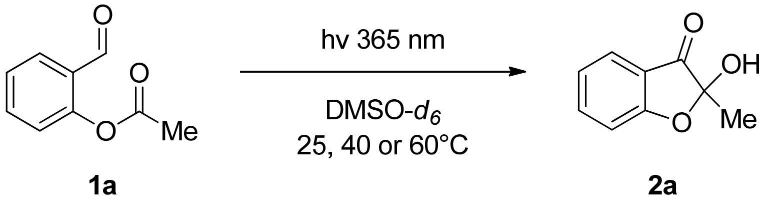

Compound **1a** (15 mg, 0.09 mmol) was dissolved in 3 mL of DMSO-d_6 in a Schlenk vessel. The mixture was degassed under vacuum and filled with argon three times. Next, parts of the solution (0.65 mL) was transferred to three argon fused NMR tubes and sealed. The mixture was analyzed by ^{1}H NMR (initial point). NMR tube with these solutions was irradiated for 30 min with 365 nm LED lamp in a glass with distilled water, with the temperature inside controlled using a magnetic stirrer with a thermocouple. The mixtures were analyzed by ^{1}H NMR (see above in Part 1).

Table S4. Study of photoreactions with different temperature.

Temperature	**2a**, %	Remained **1a**, %
25 °C	71%	27%
40 °C	77%	22%
60 °C	81%	18%

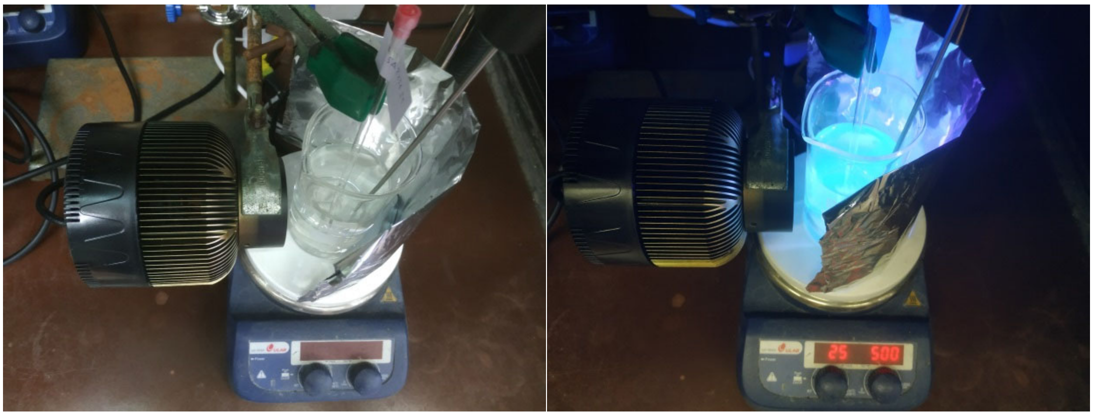

Figure S2. Photochemical set-up with heating.

5. Kinetic study

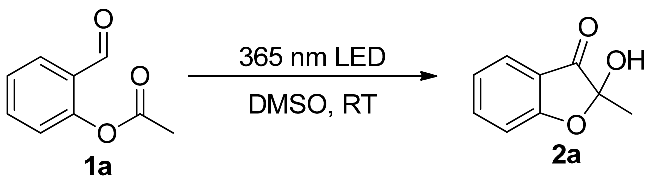

Compound **1a** (10 mg, 0.06 mmol) was dissolved in 2 mL of DMSO-d_6 in a Schlenk vessel. The mixture was degassed under vacuum and filled with argon three times. Next, 0.65 mL portions of the solution were transferred to two argon fused NMR tube and sealed. The process was carried out strictly with two samples at a time. One of two samples was wrapped in a foil and served as a blank standard confirming that the reaction does not proceed without irradiation at the similar conditions (solvent, time, temperature). NMR tubes with these solutions were irradiated with 365 nm LED lamp in Evoluchem™ PhotoRedOx box for 4 h. The mixtures were analyzed by ^{1}H NMR. TMS signal was used as an internal standard - for all spectra its integral area was set on equal value. Initial integral area of aldehyde singlet signal from 10.03 ppm to 10.15 ppm was determined as 100%. The yields of component were determined using the following integral areas:

- singlet from 10.03 ppm to 10.15 ppm (1H) for the starting aldehyde **1a**.
- singlet from 1.40 ppm to 1.52 ppm (3H) for product **2a**

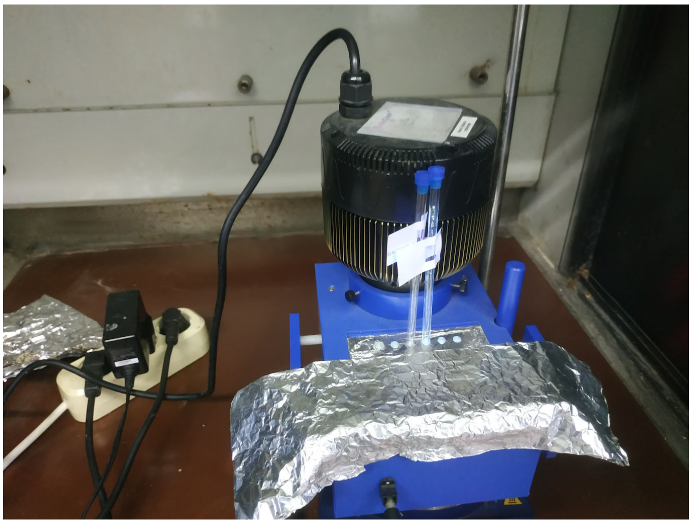

Figure S3. Photochemical set-up in NMR tubes.

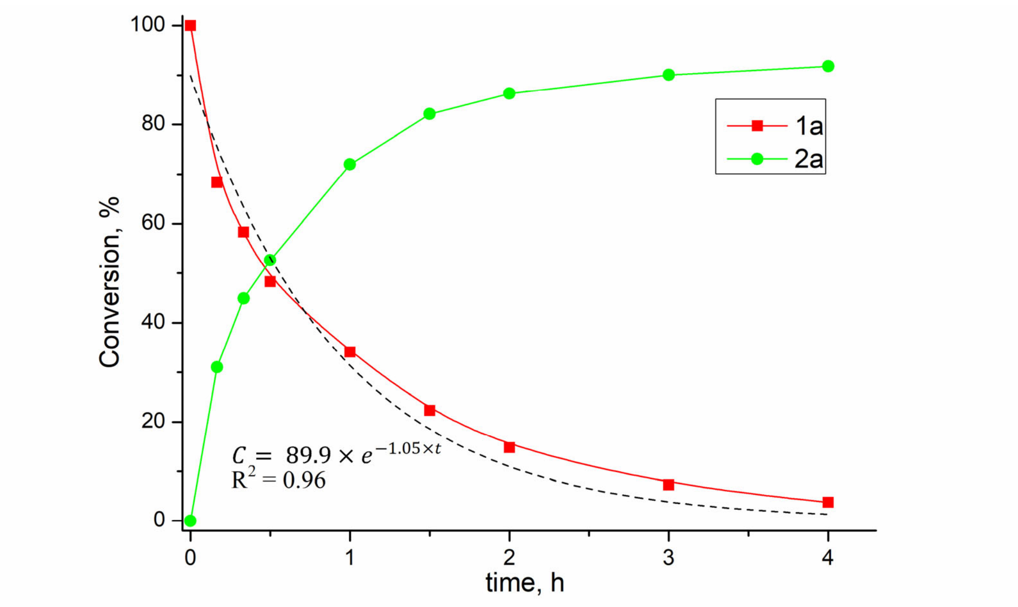

Figure S4. Kinetic study of compound **1a**.

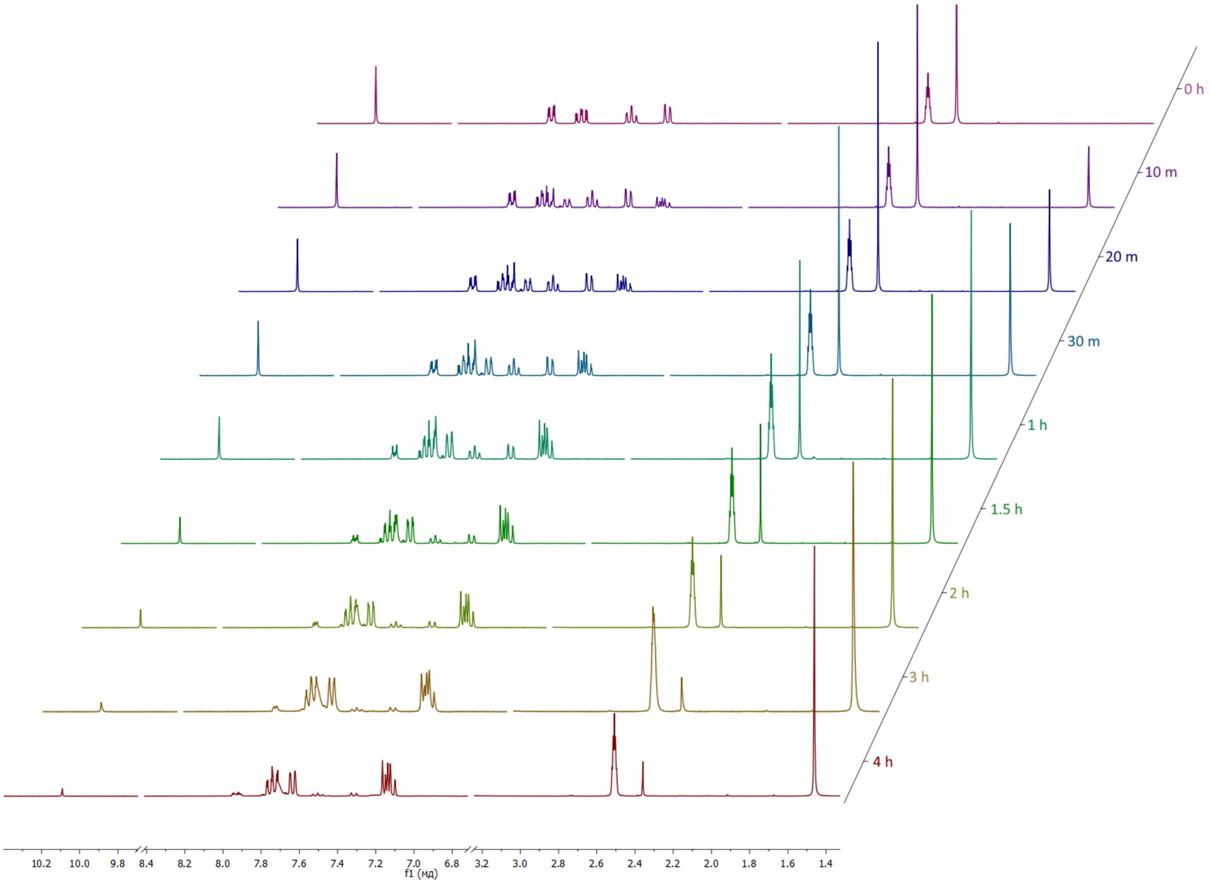

Figure S5. ^{1}H NMR of kinetic experiment.

6. Experiments with radical quenching reagents

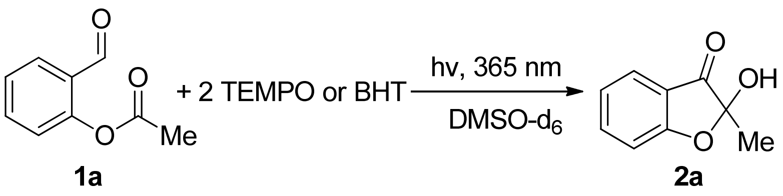

Compound **1a** (15 mg, 0.09 mmol) was dissolved in 3 mL of DMSO-*d₆* in a Schlenk vessel. The mixture was degassed under vacuum and filled with argon three times. Next, parts of the solution (0.65 mL) were transferred to three argon-filled NMR tubes and sealed. (2,2,6,6-tetramethylpiperidin-1-yl)oxidanyl (TEMPO, 6 mg, 0.04 mmol) was added to one tube, butylated hydroxytoluene (BHT, 8 mg, 0.04 mmol) was added to the second, and the last tube was used without additives. NMR tubes with these solutions were sealed and irradiated with 365 nm LED lamp in Evoluchem™ PhotoRedOx box. The mixtures were analyzed by ^{1}H NMR (see above in Part 1).

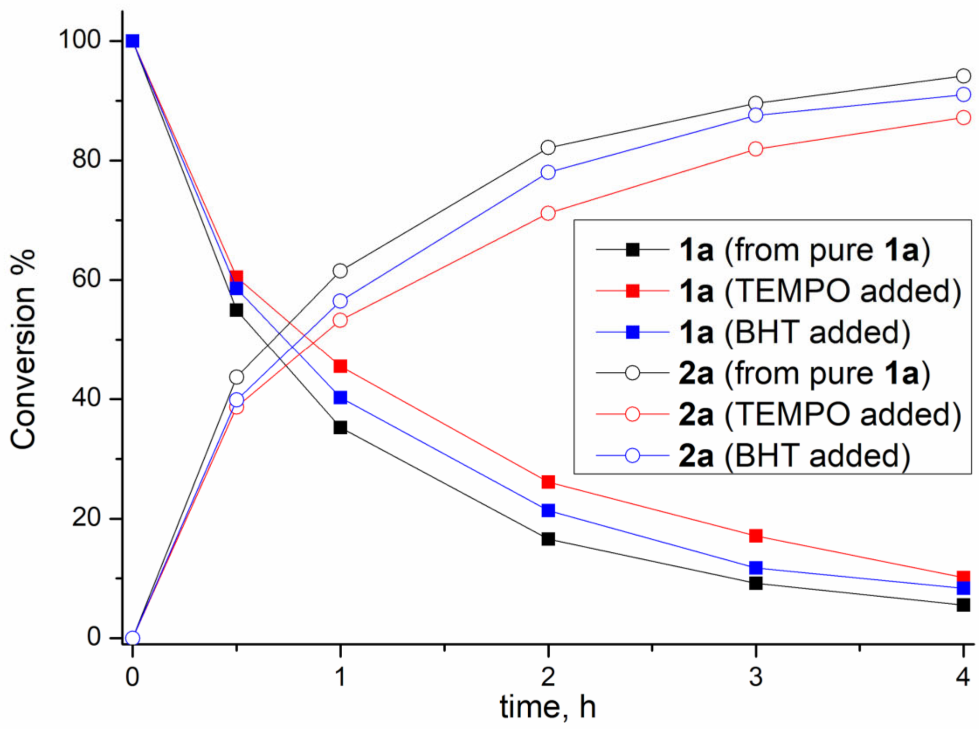

Figure S6. Kinetic study of **1a** phototranformation with BHT and TEMPO.

7. Cross-reaction experiment

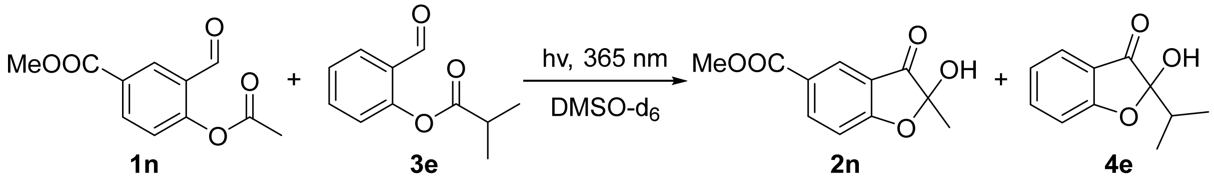

Compounds **1n** (7 mg, 0.03 mmol) and **3e** (6 mg, 0.03 mmol) were dissolved in 1 mL of DMSO-d_6 in a Schlenk vessel. The mixture was degassed under vacuum and filled with argon three times. Next, 0.65 mL portion of the solution were transferred to argon fused NMR tube and sealed. The mixture was analyzed by ^{1}H NMR (initial point). NMR tube with this solution was irradiated with 365 nm LED lamp in Evoluchem™ PhotoRedOx box for 4 hours. The mixture was analyzed by ^{1}H NMR. Only products **2n** and **4e** were detected.

8. Copies of ^{1}H and ^{13}C NMR spectra

1a

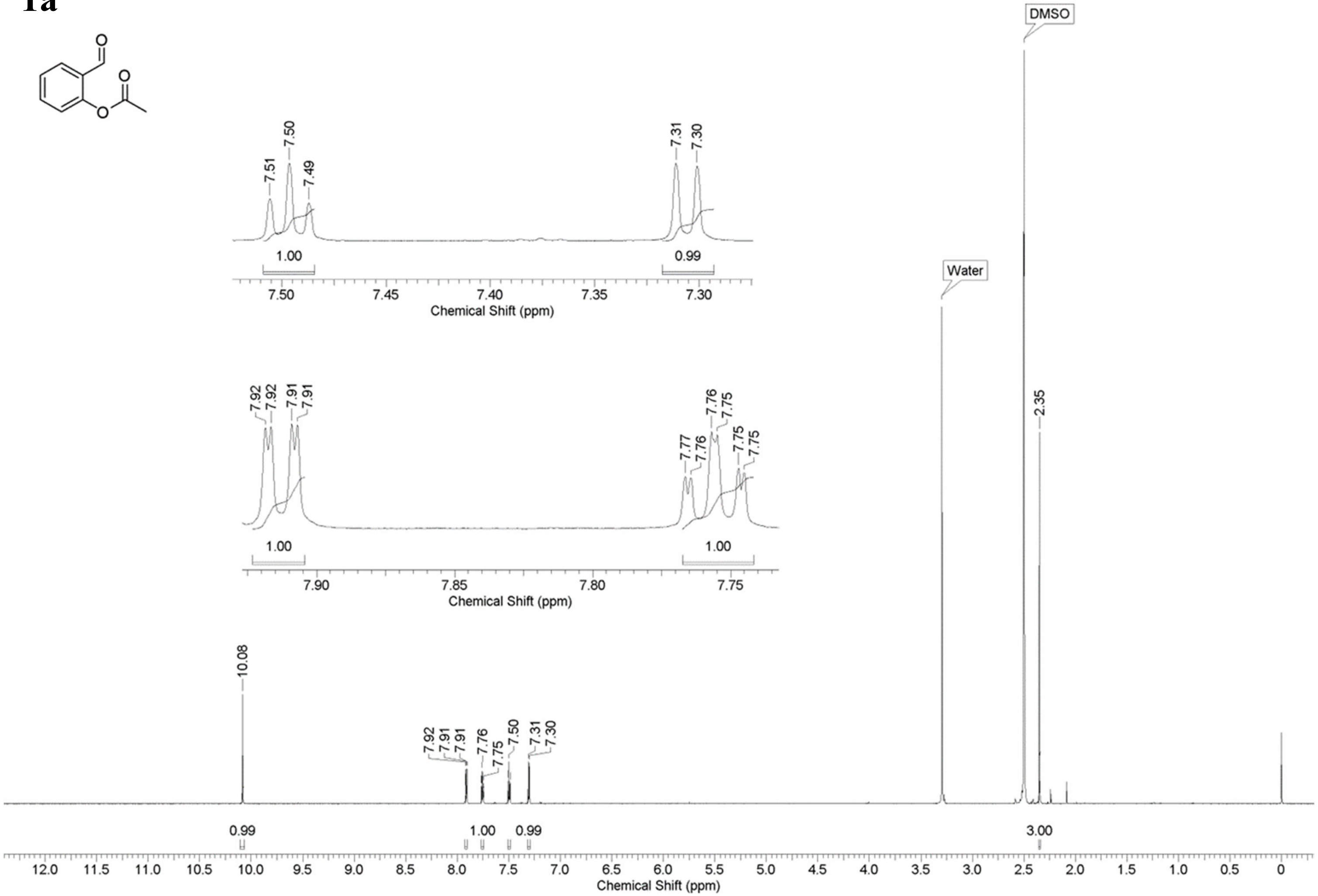

1b

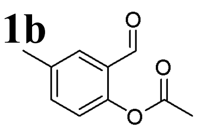

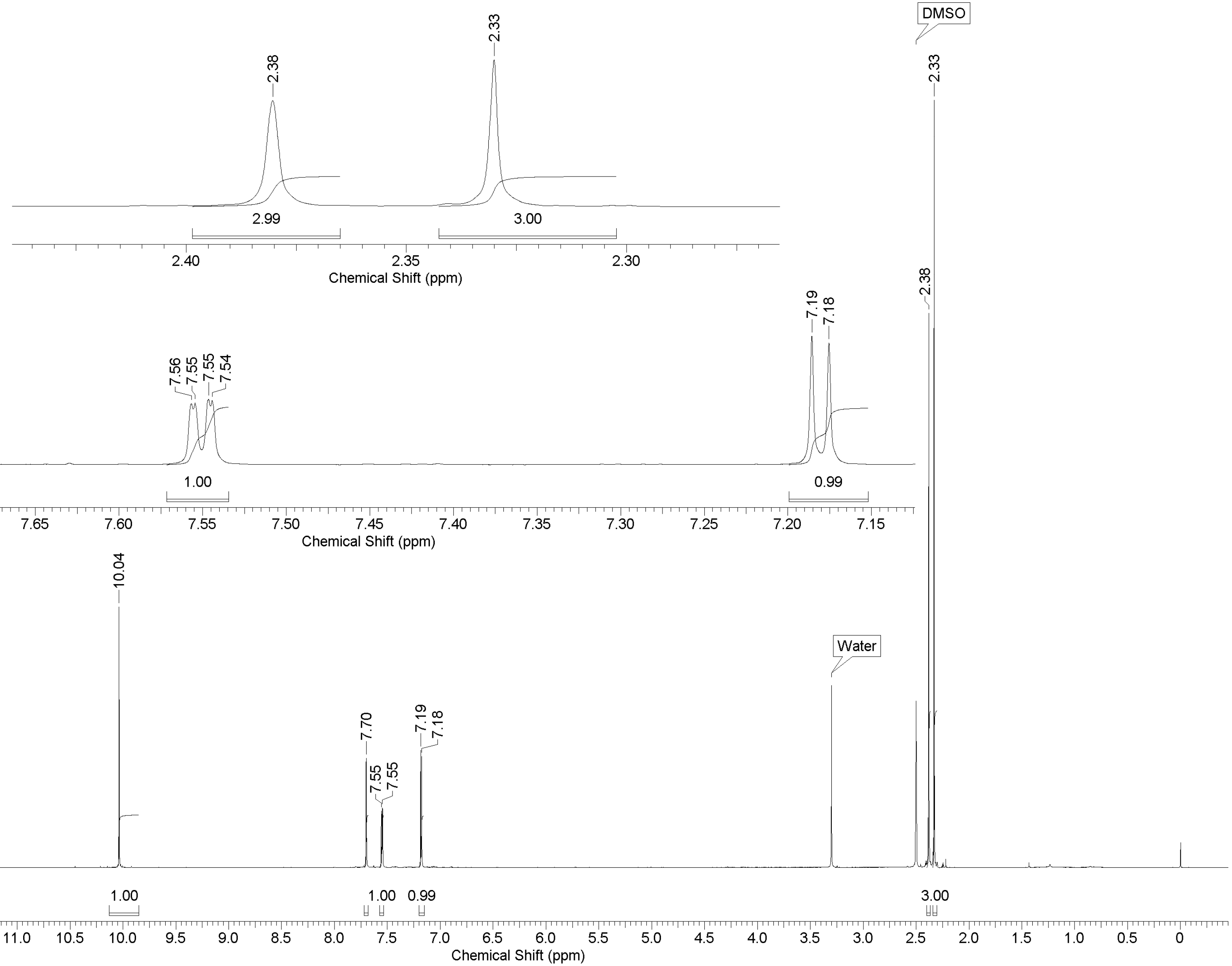

DMSO
Water
2.38
2.33
2.99
3.00
2.40
2.35
2.30
Chemical Shift (ppm)
7.70
7.70
7.56
7.55
7.55
7.54
7.19
7.18
1.00
1.00
0.99
7.75 7.70 7.65 7.60 7.55 7.50 7.45 7.40 7.35 7.30 7.25 7.20 7.15
Chemical Shift (ppm)
10.04
7.70
7.55
7.55
7.19
7.18
1.00
1.00 0.99
3.00
12.5 12.0 11.5 11.0 10.5 10.0 9.5 9.0 8.5 8.0 7.5 7.0 6.5 6.0 5.5 5.0 4.5 4.0 3.5 3.0 2.5 2.0 1.5 1.0 0.5 0
Chemical Shift (ppm)

1c

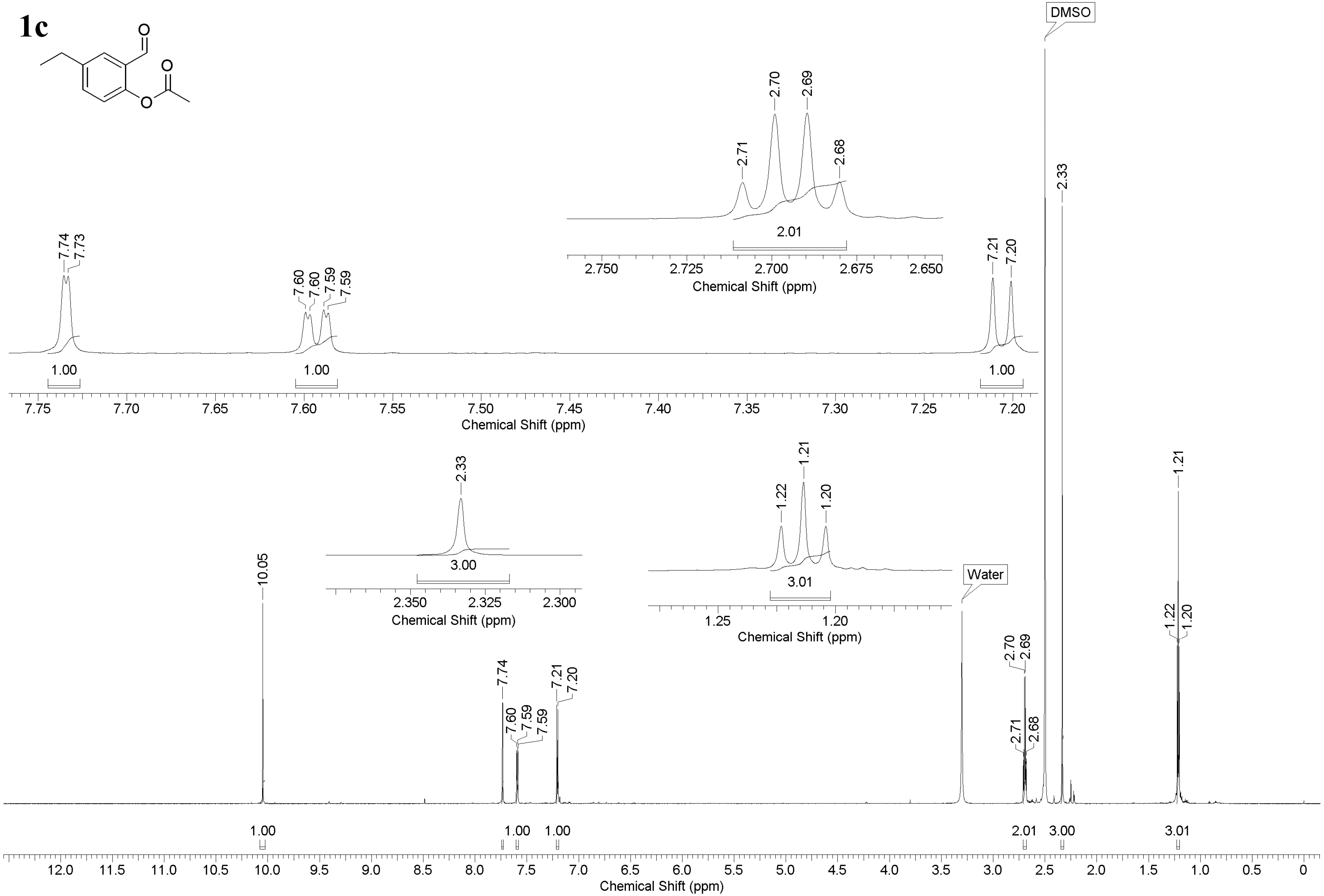
DMSO
Water
10.05
7.74
7.73
7.60
7.59
7.21
7.20
2.71
2.70
2.69
2.68
2.33
1.22
1.21
1.20
1.00
2.01
3.00
3.01
Chemical Shift (ppm)

1c

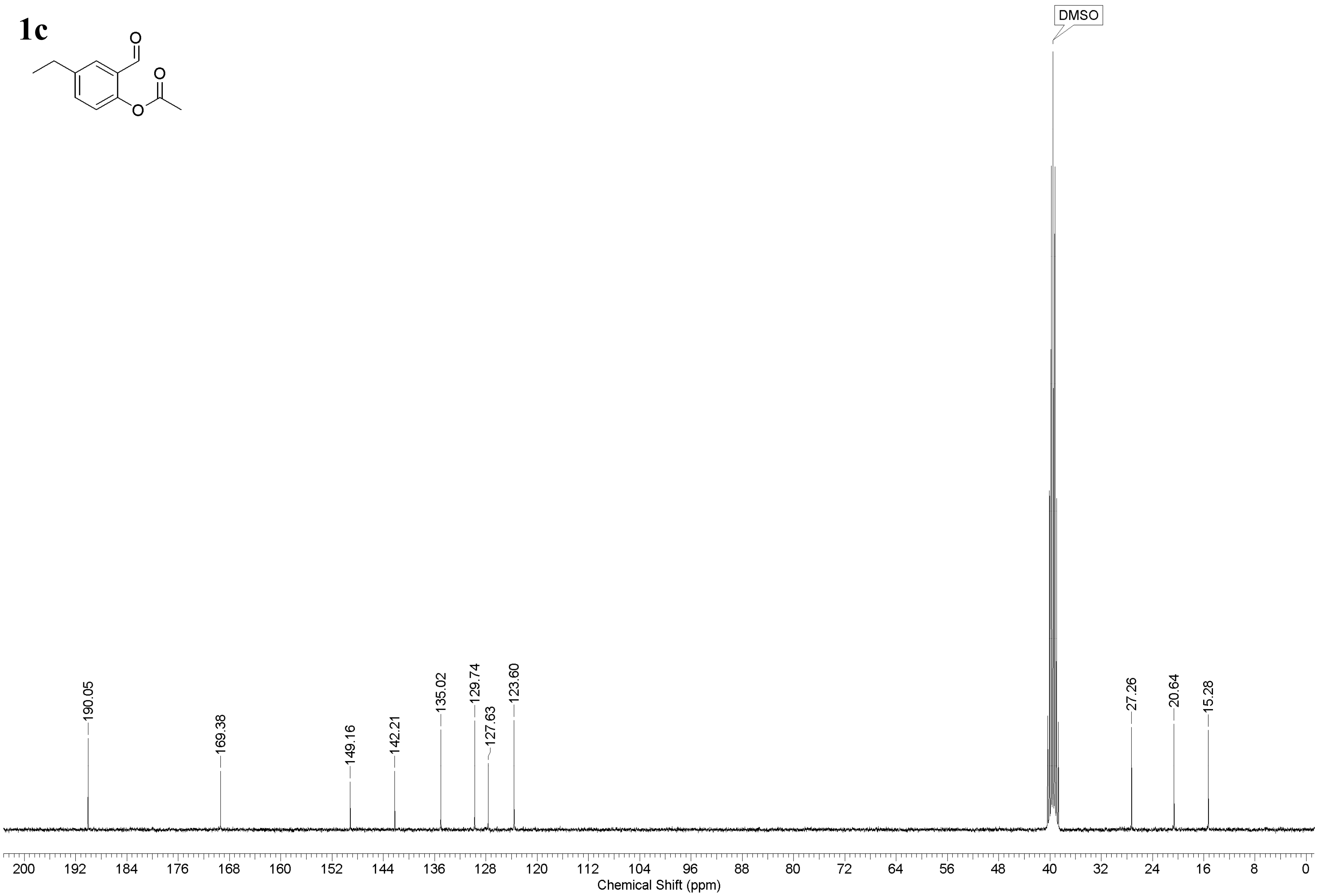
DMSO
190.05
169.38
149.16
142.21
135.02
129.74
127.63
123.60
27.26
20.64
15.28
200
192
184
176
168
160
152
144
136
128
120
112
104
96
88
80
72
64
56
48
40
32
24
16
8
0
Chemical Shift (ppm)

1d

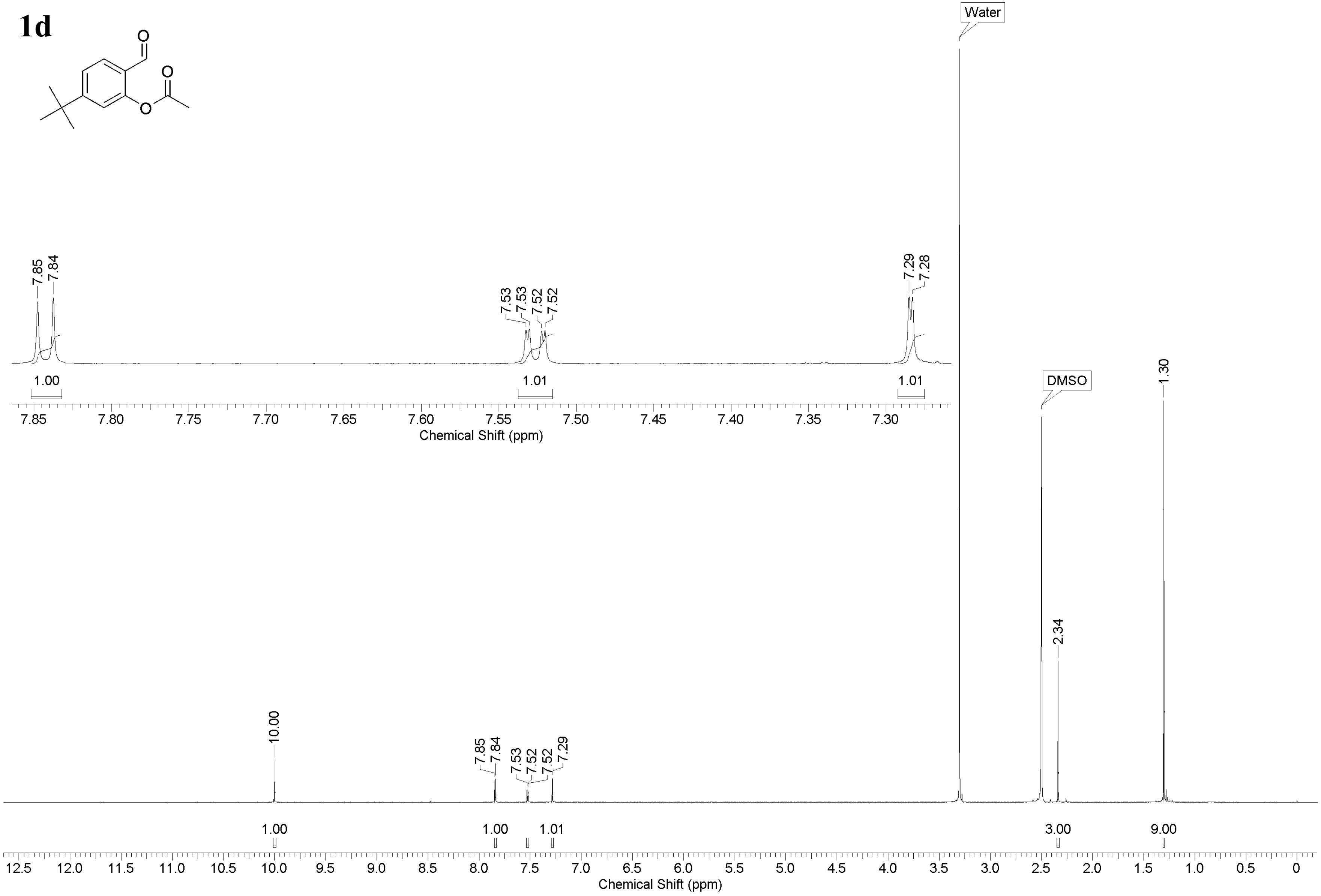
Water
DMSO
7.85
7.84
7.53
7.53
7.52
7.52
7.29
7.28
1.00
1.01
1.01
7.85 7.80 7.75 7.70 7.65 7.60 7.55 7.50 7.45 7.40 7.35 7.30
Chemical Shift (ppm)
10.00
7.85
7.84
7.53
7.52
7.52
7.29
2.34
1.30
1.00
1.00
1.01
3.00
9.00
12.5 12.0 11.5 11.0 10.5 10.0 9.5 9.0 8.5 8.0 7.5 7.0 6.5 6.0 5.5 5.0 4.5 4.0 3.5 3.0 2.5 2.0 1.5 1.0 0.5 0
Chemical Shift (ppm)

1d

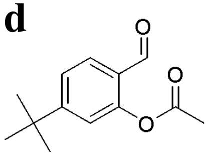

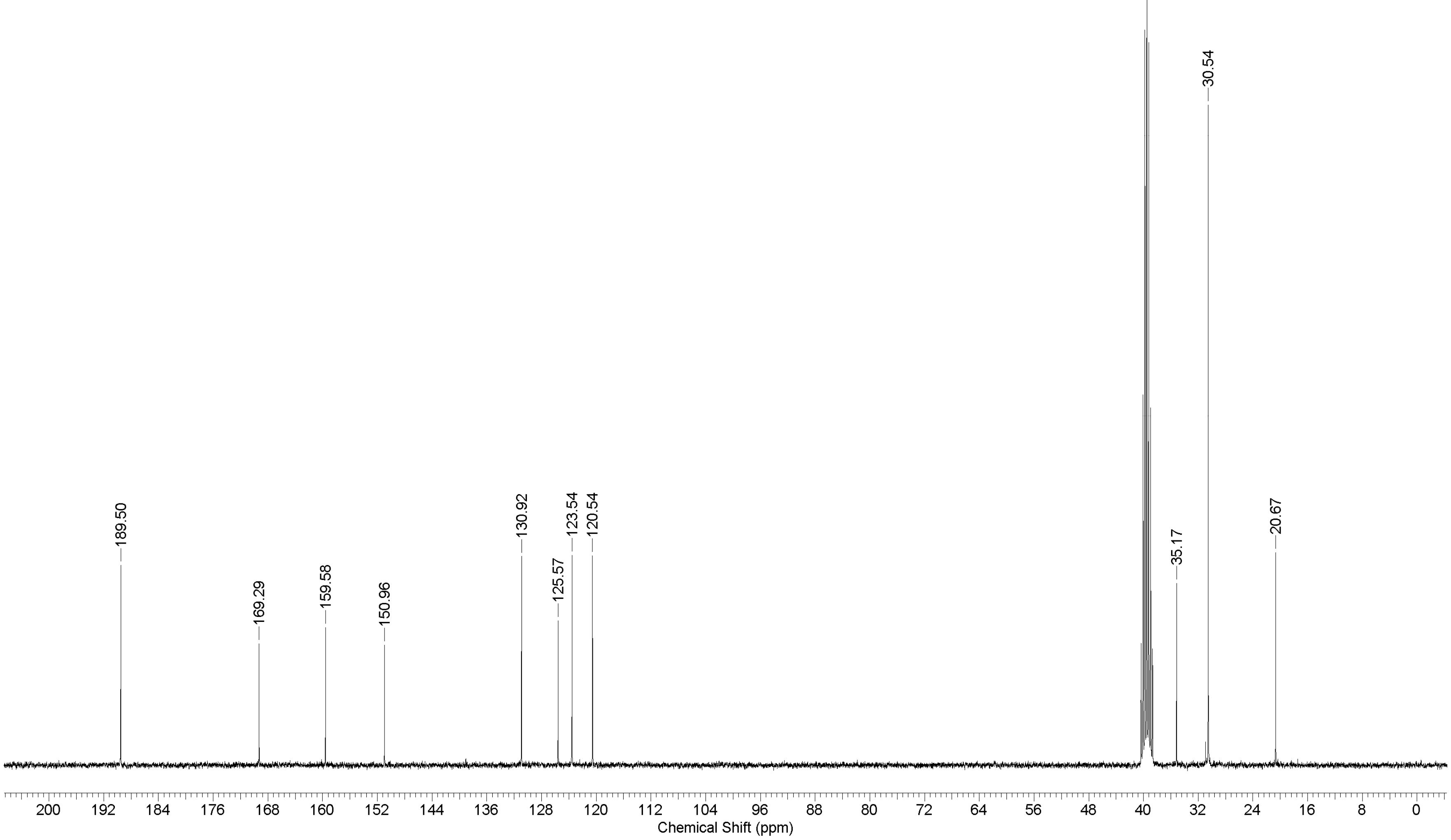

DMSO
189.50
169.29
159.58
150.96
130.92
125.57
123.54
120.54
35.17
30.54
20.67
200
192
184
176
168
160
152
144
136
128
120
112
104
96
88
80
72
64
56
48
40
32
24
16
8
0
Chemical Shift (ppm)

1e

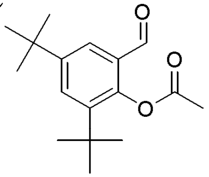

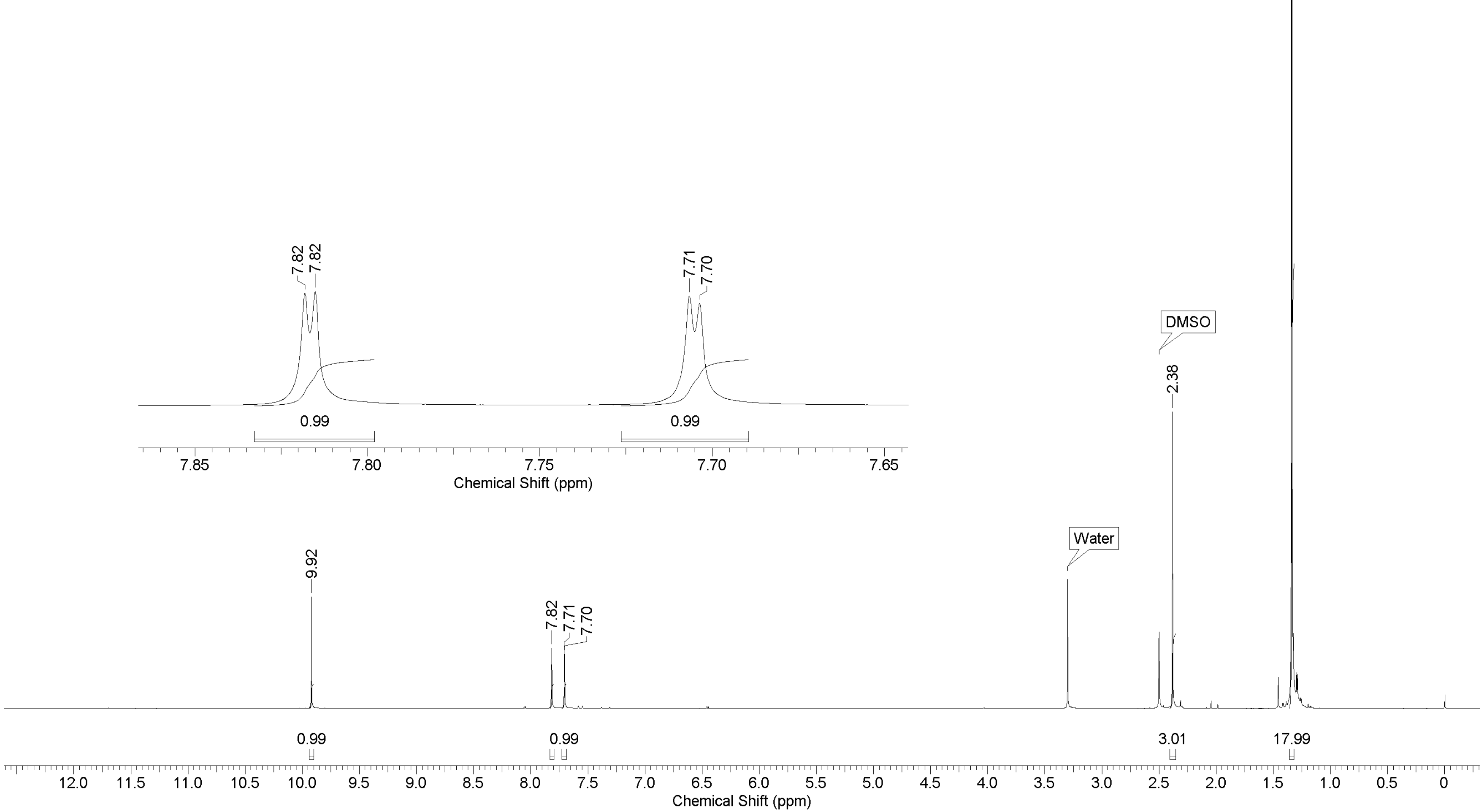

1.34
1.34
7.82
7.82
7.71
7.70
DMSO
2.38
0.99
0.99
7.85
7.80
7.75
7.70
7.65
Chemical Shift (ppm)
Water
9.92
7.82
7.71
7.70
0.99
0.99
3.01
17.99
12.0
11.5
11.0
10.5
10.0
9.5
9.0
8.5
8.0
7.5
7.0
6.5
6.0
5.5
5.0
4.5
4.0
3.5
3.0
2.5
2.0
1.5
1.0
0.5
0
Chemical Shift (ppm)

1f

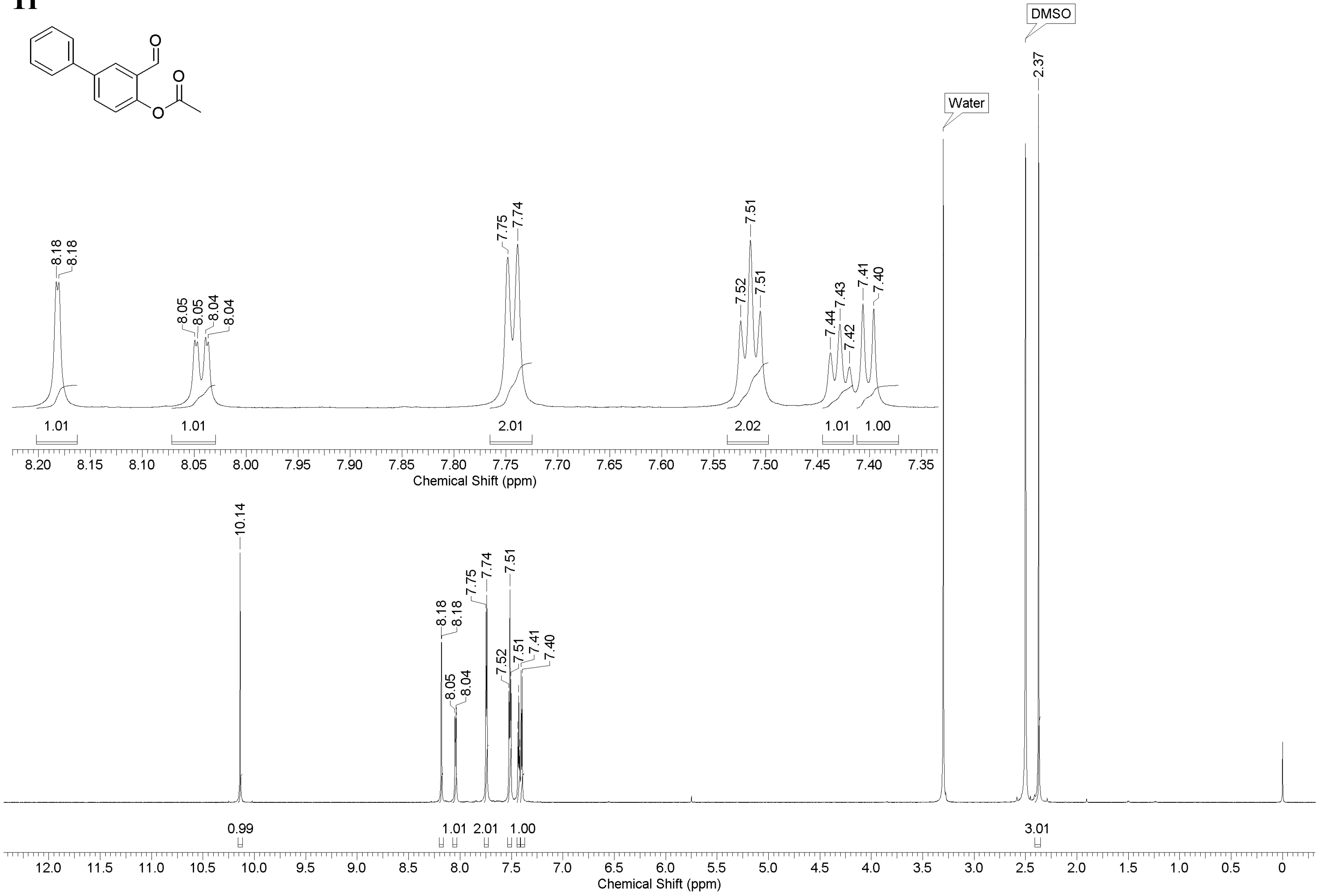
O
O
O
DMSO
2.37
Water
8.18
8.18
8.05
8.05
8.04
8.04
7.75
7.74
7.52
7.51
7.51
7.44
7.43
7.42
7.41
7.40
1.01
1.01
2.01
2.02
1.01
1.00
8.20 8.15 8.10 8.05 8.00 7.95 7.90 7.85 7.80 7.75 7.70 7.65 7.60 7.55 7.50 7.45 7.40 7.35
Chemical Shift (ppm)
10.14
0.99
1.01 2.01 1.00
3.01
12.0 11.5 11.0 10.5 10.0 9.5 9.0 8.5 8.0 7.5 7.0 6.5 6.0 5.5 5.0 4.5 4.0 3.5 3.0 2.5 2.0 1.5 1.0 0.5 0
Chemical Shift (ppm)

1f

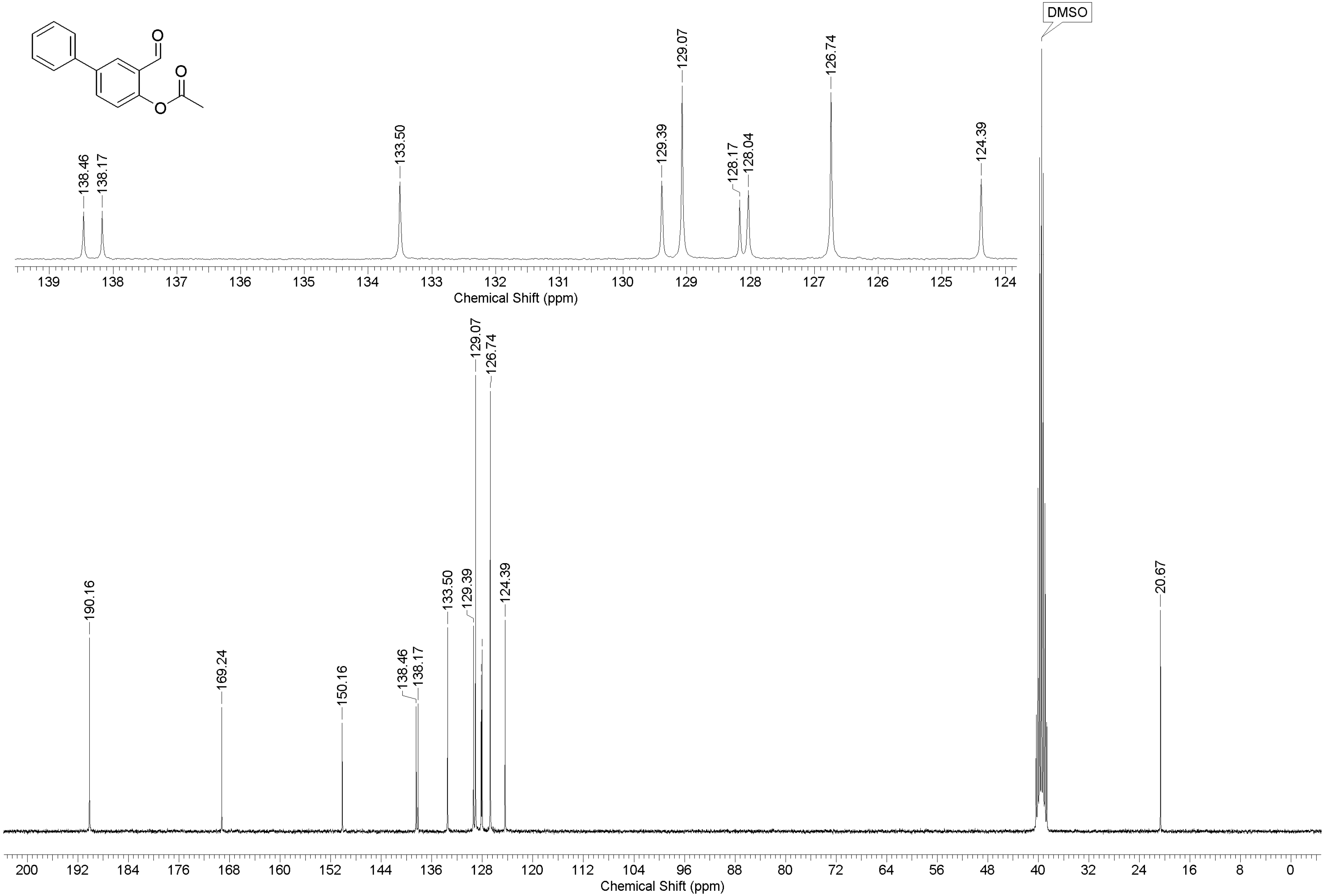

O
O
O
DMSO
138.46
138.17
133.50
129.39
129.07
128.17
128.04
126.74
124.39
139
138
137
136
135
134
133
132
131
130
129
128
127
126
125
124
Chemical Shift (ppm)
190.16
169.24
150.16
138.46
138.17
133.50
129.39
129.07
126.74
124.39
20.67
200
192
184
176
168
160
152
144
136
128
120
112
104
96
88
80
72
64
56
48
40
32
24
16
8
0
Chemical Shift (ppm)

1g

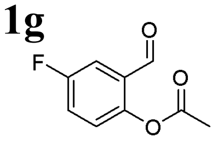
F
O
O
O

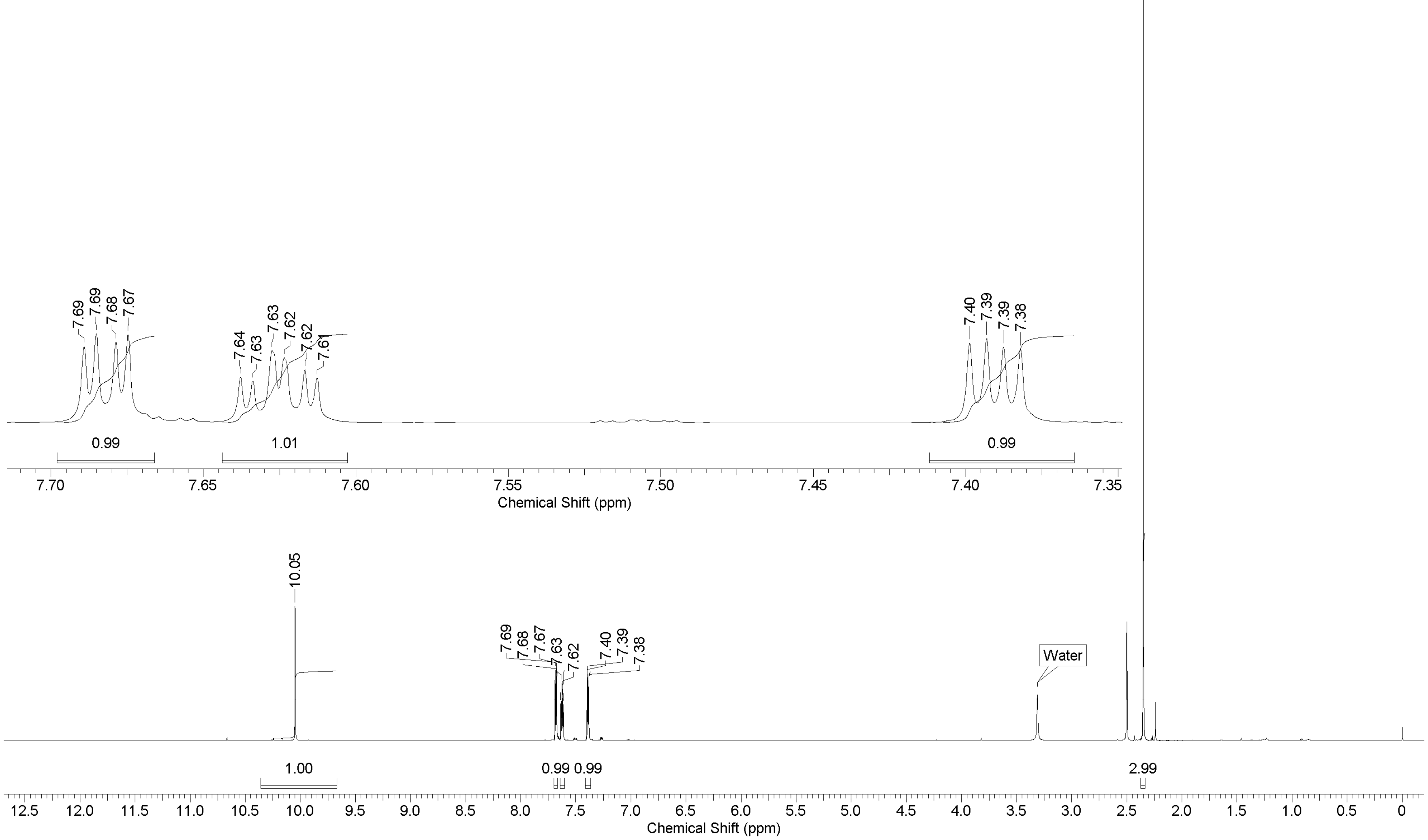
DMSO
2.35
7.69
7.69
7.68
7.67
7.64
7.63
7.63
7.62
7.62
7.61
7.40
7.39
7.39
7.38
0.99
1.01
0.99
7.70
7.65
7.60
7.55
7.50
7.45
7.40
7.35
Chemical Shift (ppm)
10.05
7.69
7.68
7.67
7.63
7.62
7.40
7.39
7.38
Water
1.00
0.99
0.99
2.99
12.5
12.0
11.5
11.0
10.5
10.0
9.5
9.0
8.5
8.0
7.5
7.0
6.5
6.0
5.5
5.0
4.5
4.0
3.5
3.0
2.5
2.0
1.5
1.0
0.5
0
Chemical Shift (ppm)

1h

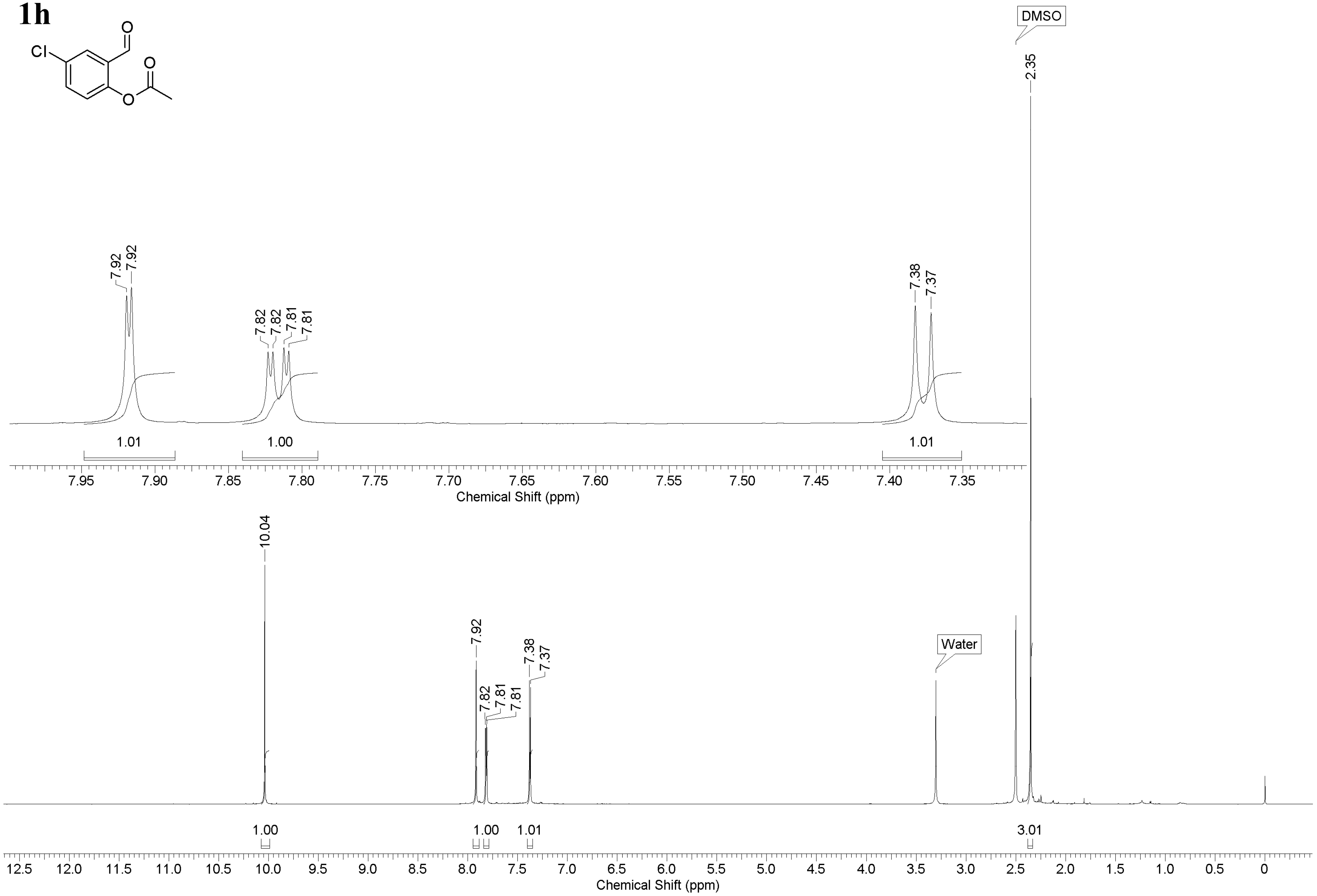
Cl
O
O
O
DMSO
2.35
7.92
7.92
7.82
7.82
7.81
7.81
7.38
7.37
1.01
1.00
1.01
7.95 7.90 7.85 7.80 7.75 7.70 7.65 7.60 7.55 7.50 7.45 7.40 7.35
Chemical Shift (ppm)
10.04
7.92
7.82
7.81
7.81
7.38
7.37
Water
1.00
1.00
1.01
3.01
12.5 12.0 11.5 11.0 10.5 10.0 9.5 9.0 8.5 8.0 7.5 7.0 6.5 6.0 5.5 5.0 4.5 4.0 3.5 3.0 2.5 2.0 1.5 1.0 0.5 0
Chemical Shift (ppm)

1i

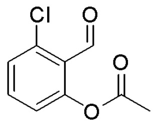

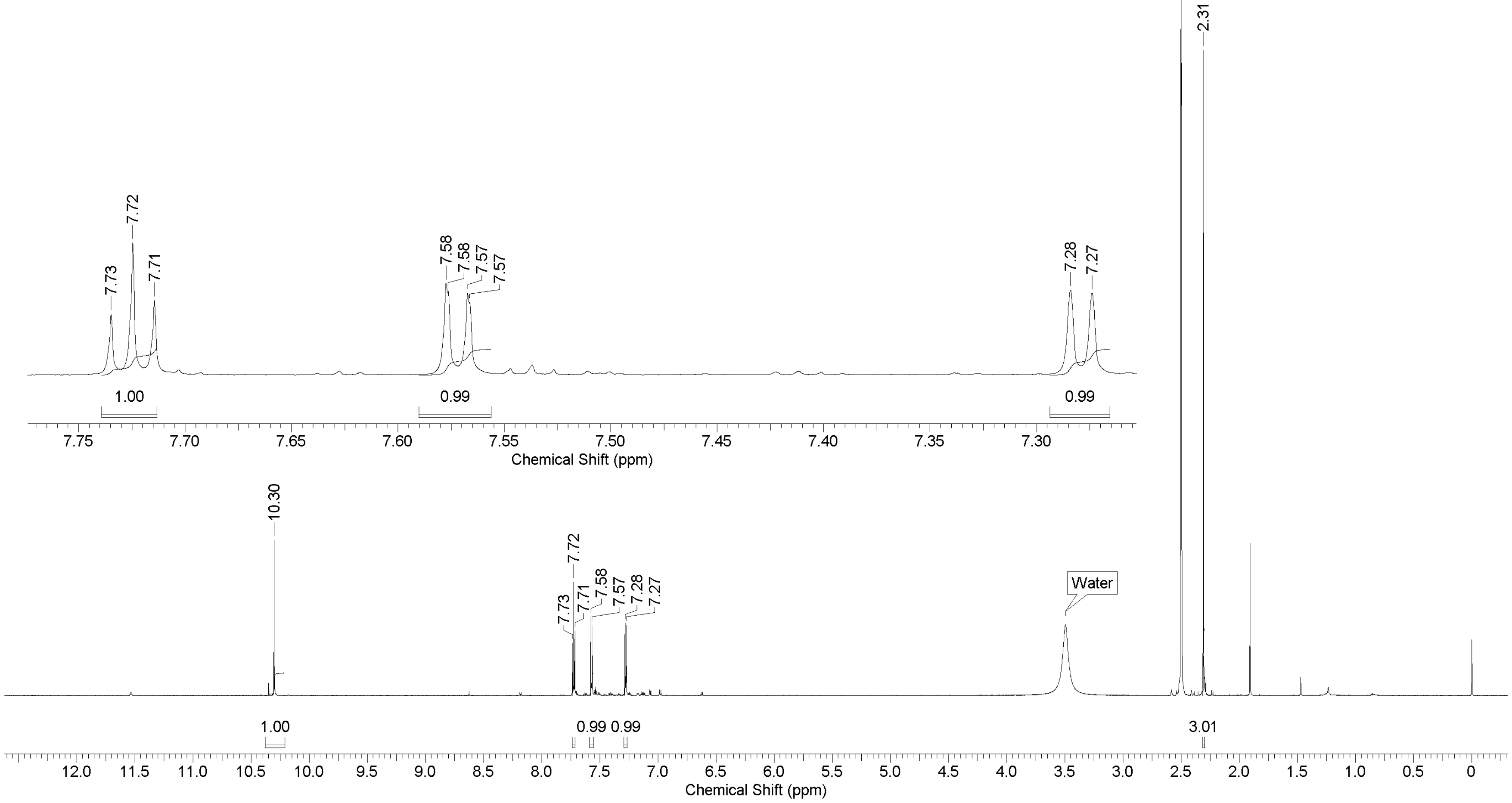
DMSO
2.31
7.73
7.72
7.71
7.58
7.58
7.57
7.57
7.28
7.27
1.00
0.99
0.99
7.75
7.70
7.65
7.60
7.55
7.50
7.45
7.40
7.35
7.30
Chemical Shift (ppm)
10.30
7.73
7.72
7.71
7.58
7.57
7.28
7.27
Water
1.00
0.99 0.99
3.01
12.0
11.5
11.0
10.5
10.0
9.5
9.0
8.5
8.0
7.5
7.0
6.5
6.0
5.5
5.0
4.5
4.0
3.5
3.0
2.5
2.0
1.5
1.0
0.5
0
Chemical Shift (ppm)

1i

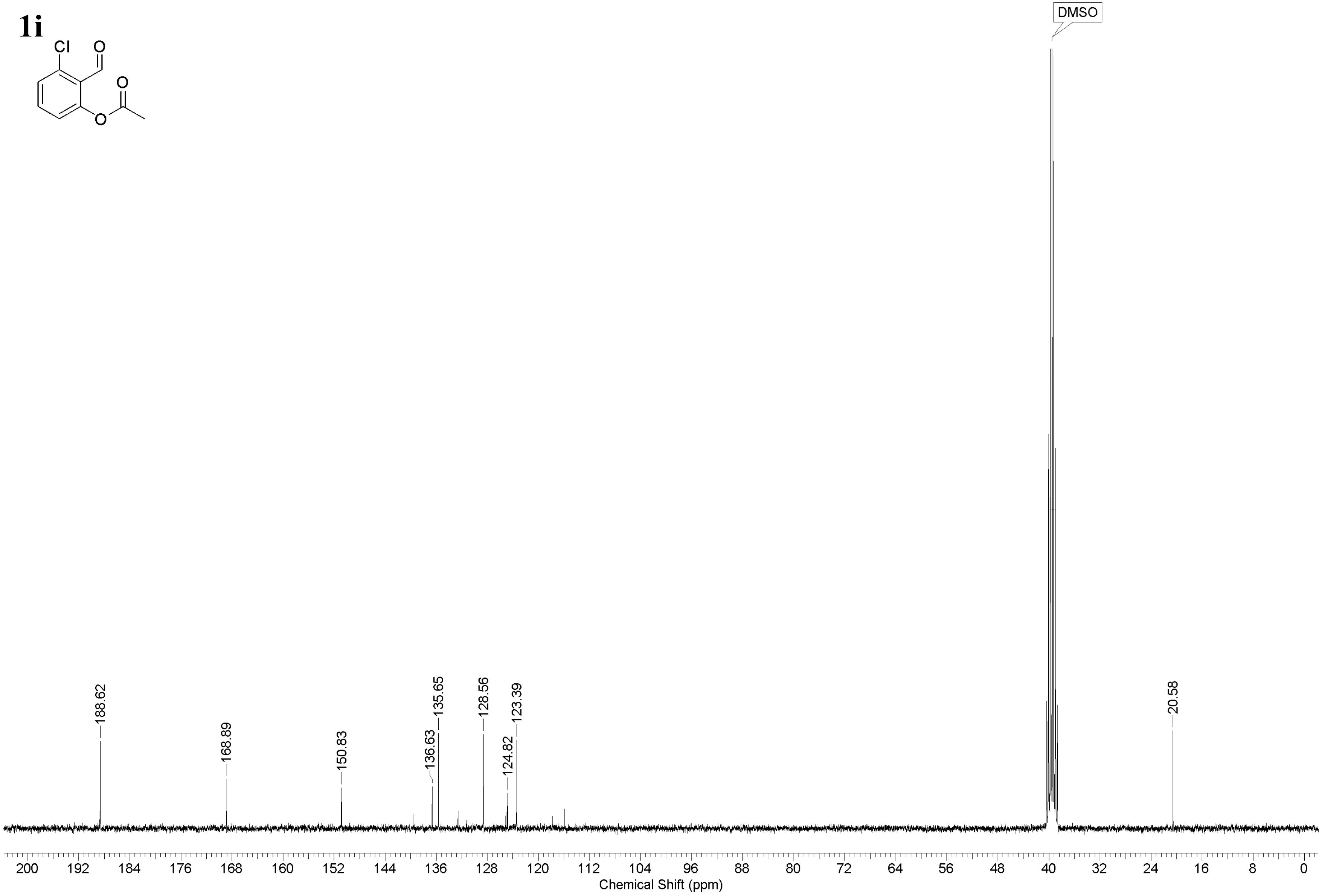

Cl
O
O
O
DMSO
188.62
168.89
150.83
136.63
135.65
128.56
124.82
123.39
20.58
200 192 184 176 168 160 152 144 136 128 120 112 104 96 88 80 72 64 56 48 40 32 24 16 8 0
Chemical Shift (ppm)

1j

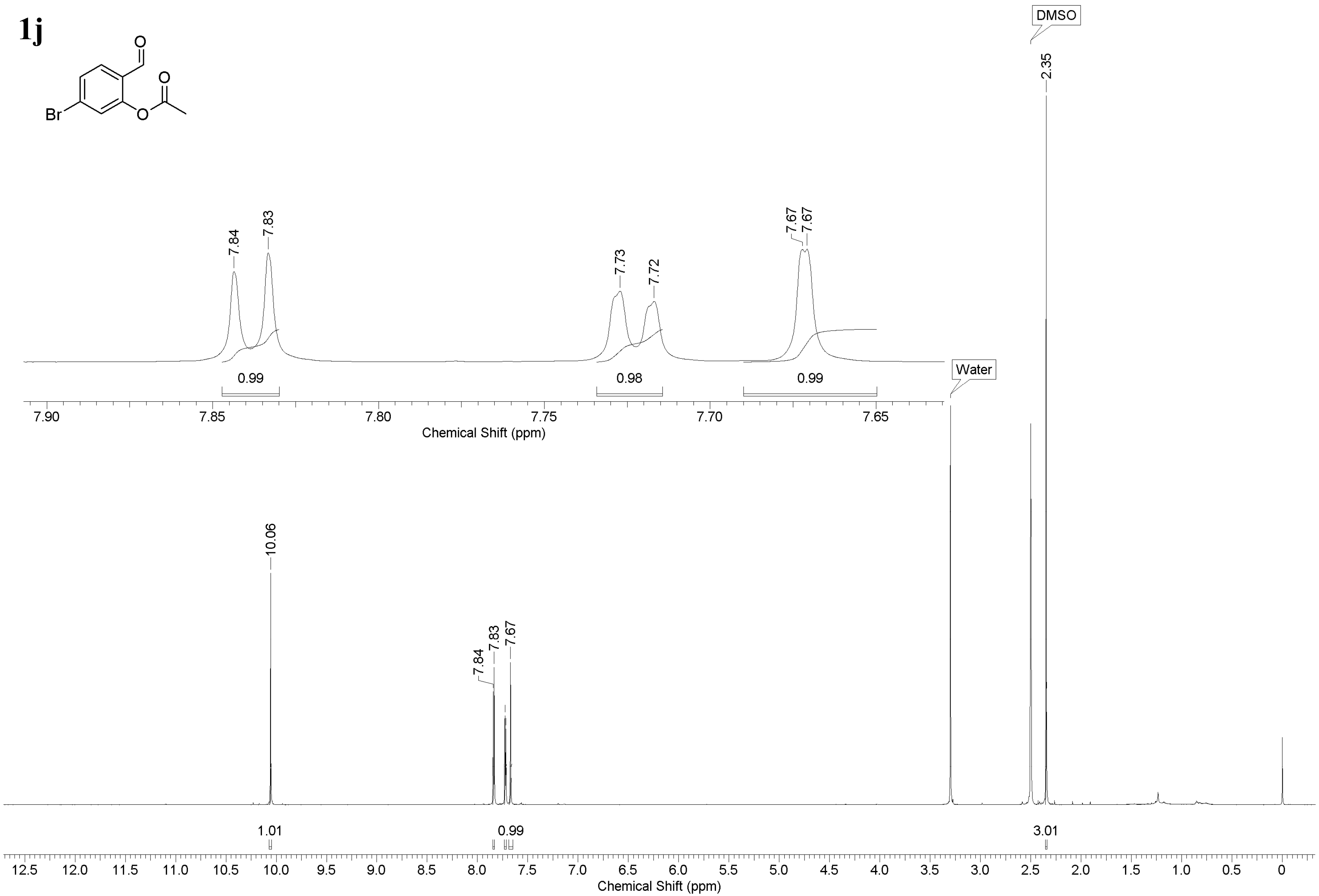
Br
O
O
O
DMSO
2.35
7.84
7.83
7.73
7.72
7.67
7.67
0.99
0.98
0.99
7.90
7.85
7.80
7.75
7.70
7.65
Chemical Shift (ppm)
Water
10.06
7.84
7.83
7.67
1.01
0.99
3.01
12.5
12.0
11.5
11.0
10.5
10.0
9.5
9.0
8.5
8.0
7.5
7.0
6.5
6.0
5.5
5.0
4.5
4.0
3.5
3.0
2.5
2.0
1.5
1.0
0.5
0
Chemical Shift (ppm)

1k

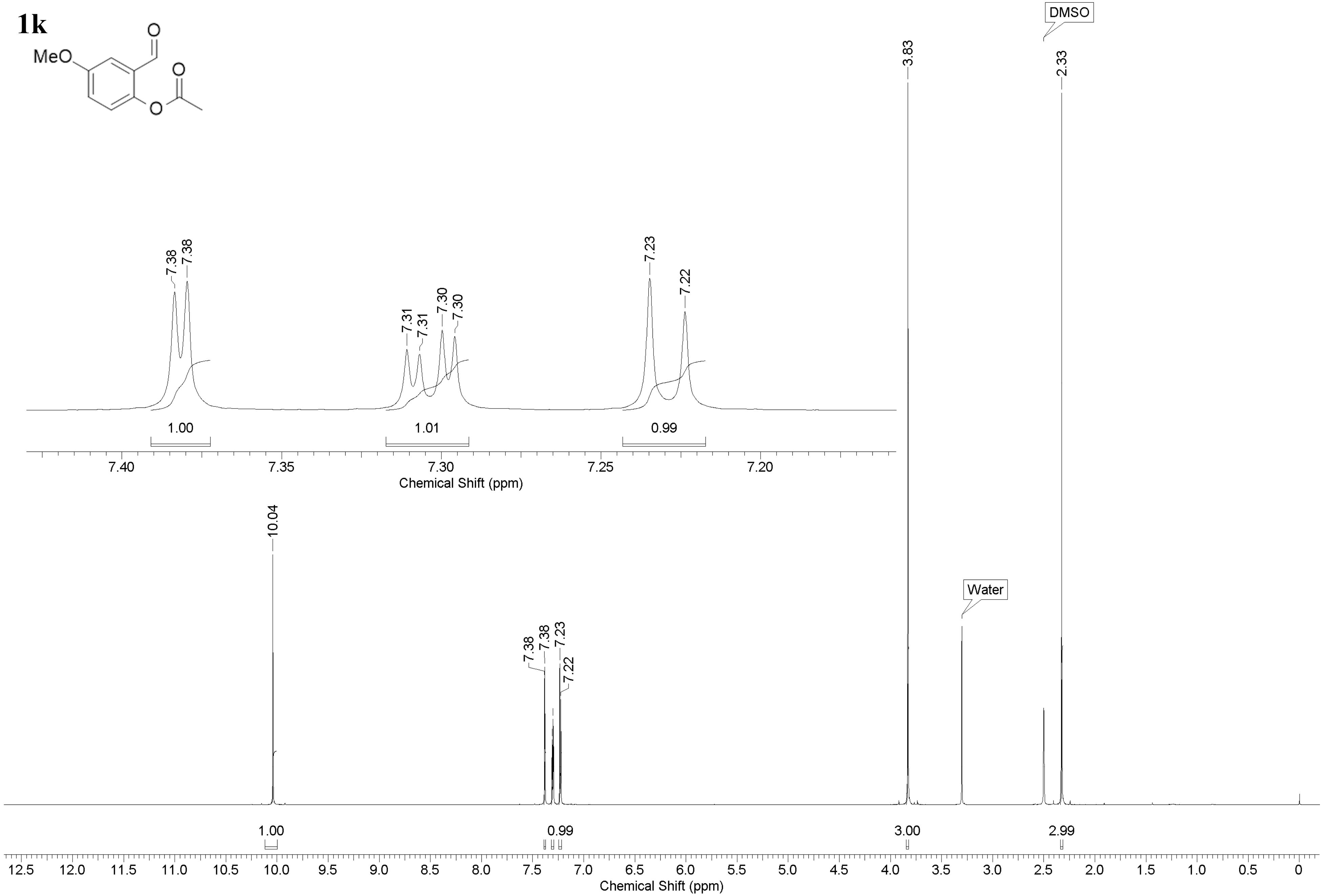

MeO
DMSO
Water
3.83
2.33
10.04
7.38
7.38
7.31
7.31
7.30
7.30
7.23
7.22
1.00
1.01
0.99
3.00
2.99
7.40
7.35
7.30
7.25
7.20
Chemical Shift (ppm)
12.5
12.0
11.5
11.0
10.5
10.0
9.5
9.0
8.5
8.0
7.5
7.0
6.5
6.0
5.5
5.0
4.5
4.0
3.5
3.0
2.5
2.0
1.5
1.0
0.5
0
Chemical Shift (ppm)

1l

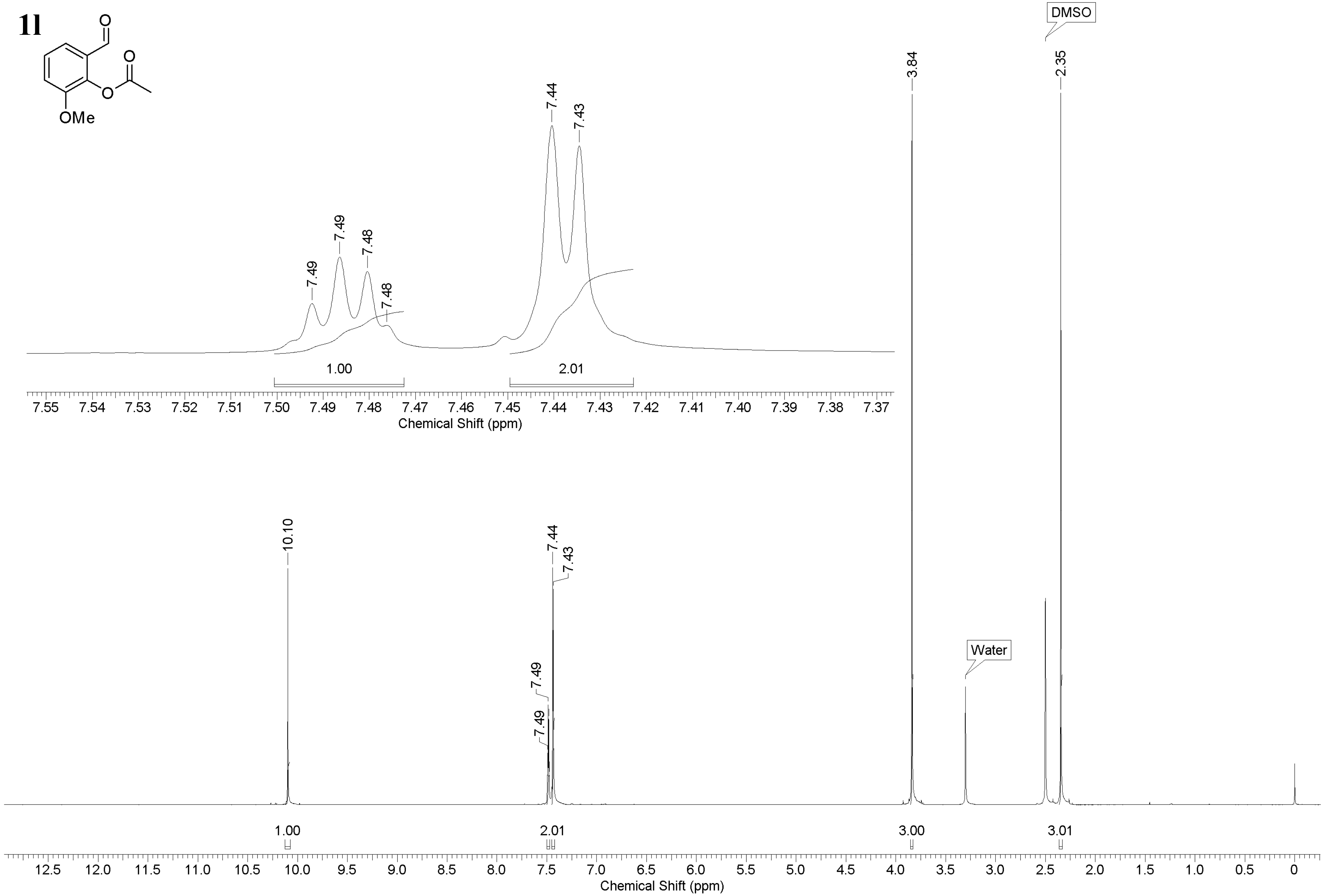
OMe
DMSO
Water
3.84
2.35
7.49
7.48
7.44
7.43
1.00
2.01
10.10
3.00
3.01
Chemical Shift (ppm)

1m

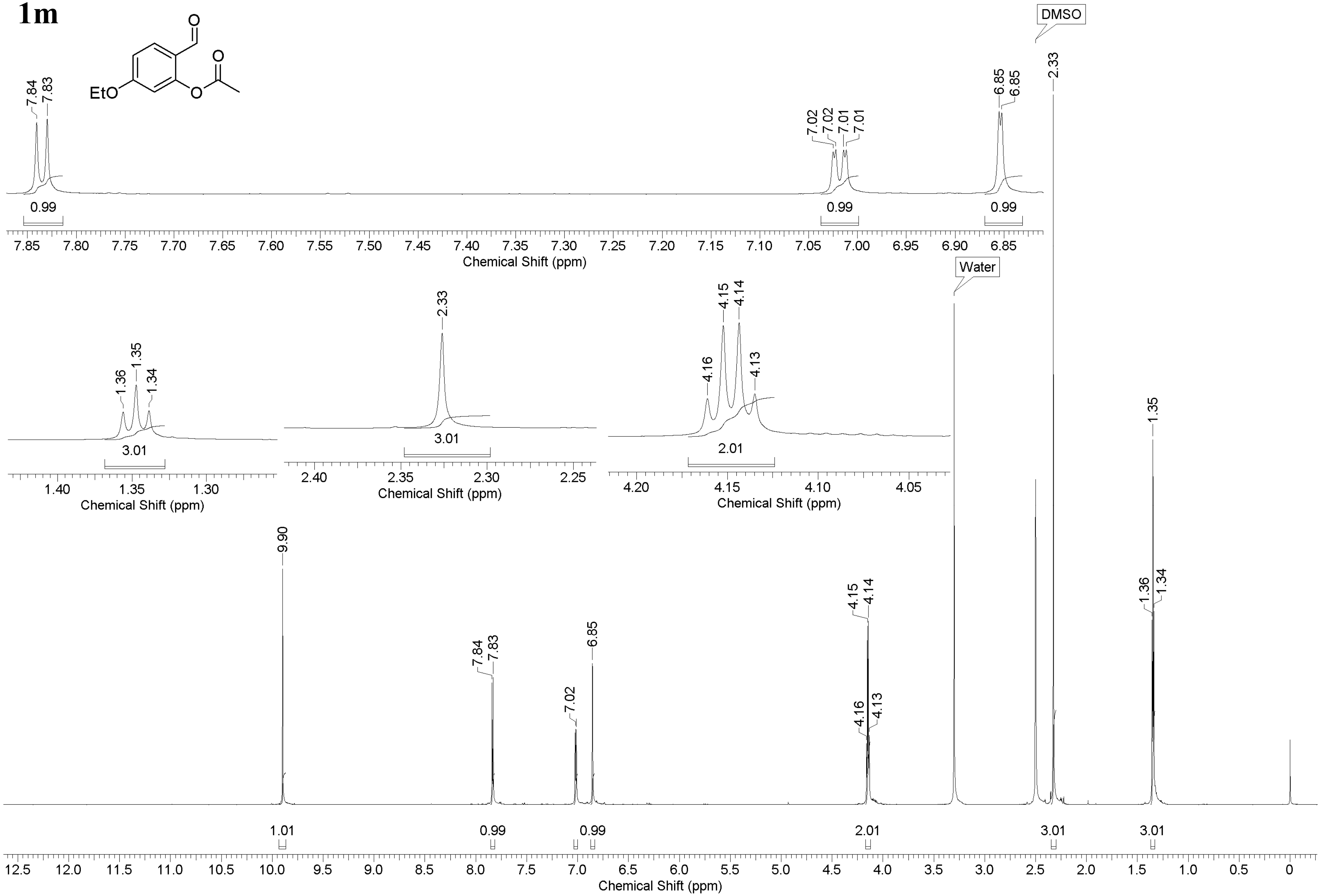
EtO
O
O
DMSO
Water
7.84
7.83
7.02
7.02
7.01
7.01
6.85
6.85
2.33
0.99
0.99
0.99
Chemical Shift (ppm)
1.36
1.35
1.34
3.01
2.33
3.01
4.16
4.15
4.14
4.13
2.01
9.90
4.15
4.14
4.16
4.13
1.36
1.34
1.35
7.84
7.83
7.02
6.85
1.01
0.99
0.99
2.01
3.01
3.01

1m

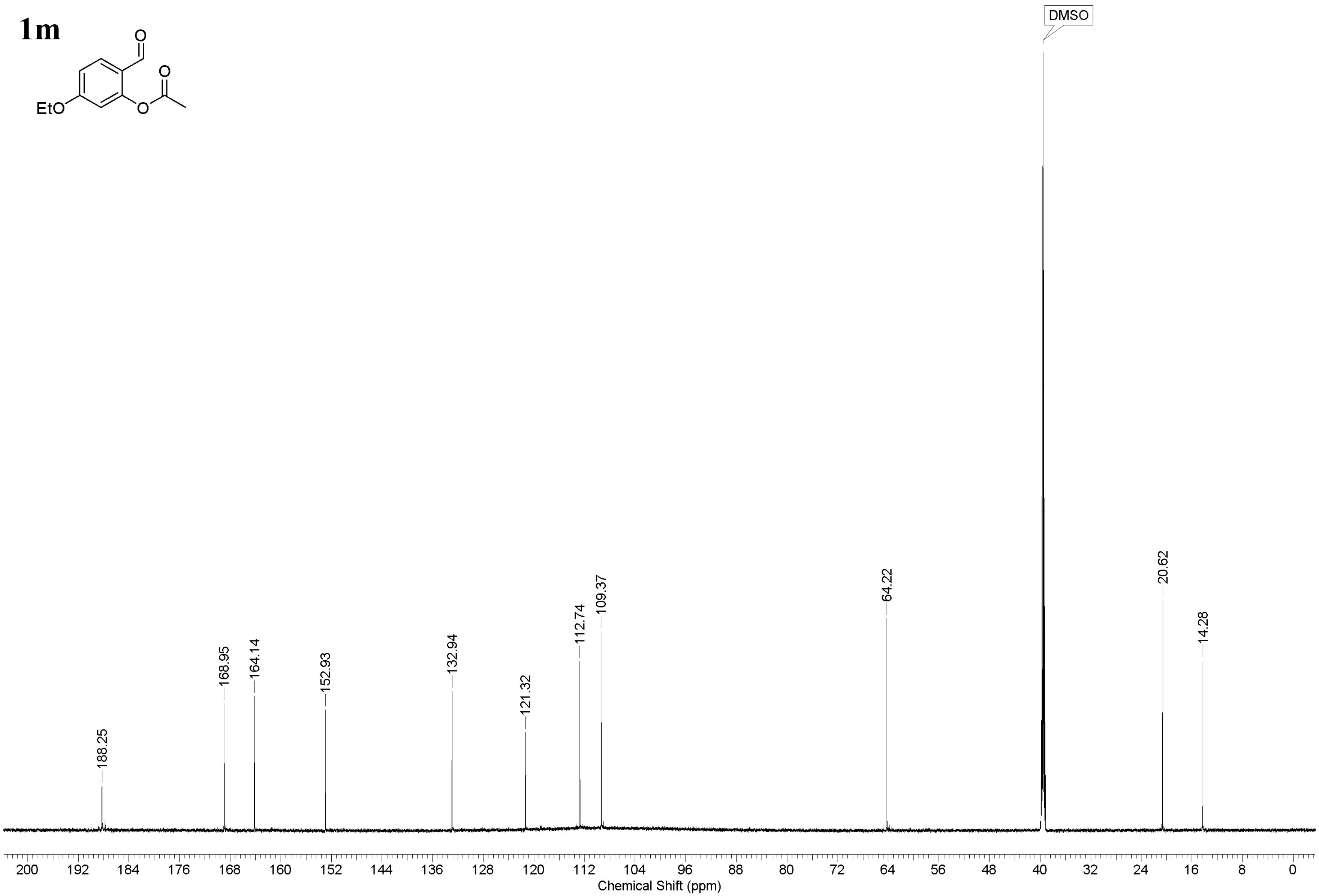

EtO
DMSO
188.25
168.95
164.14
152.93
132.94
121.32
112.74
109.37
64.22
20.62
14.28
200 192 184 176 168 160 152 144 136 128 120 112 104 96 88 80 72 64 56 48 40 32 24 16 8 0
Chemical Shift (ppm)

1n

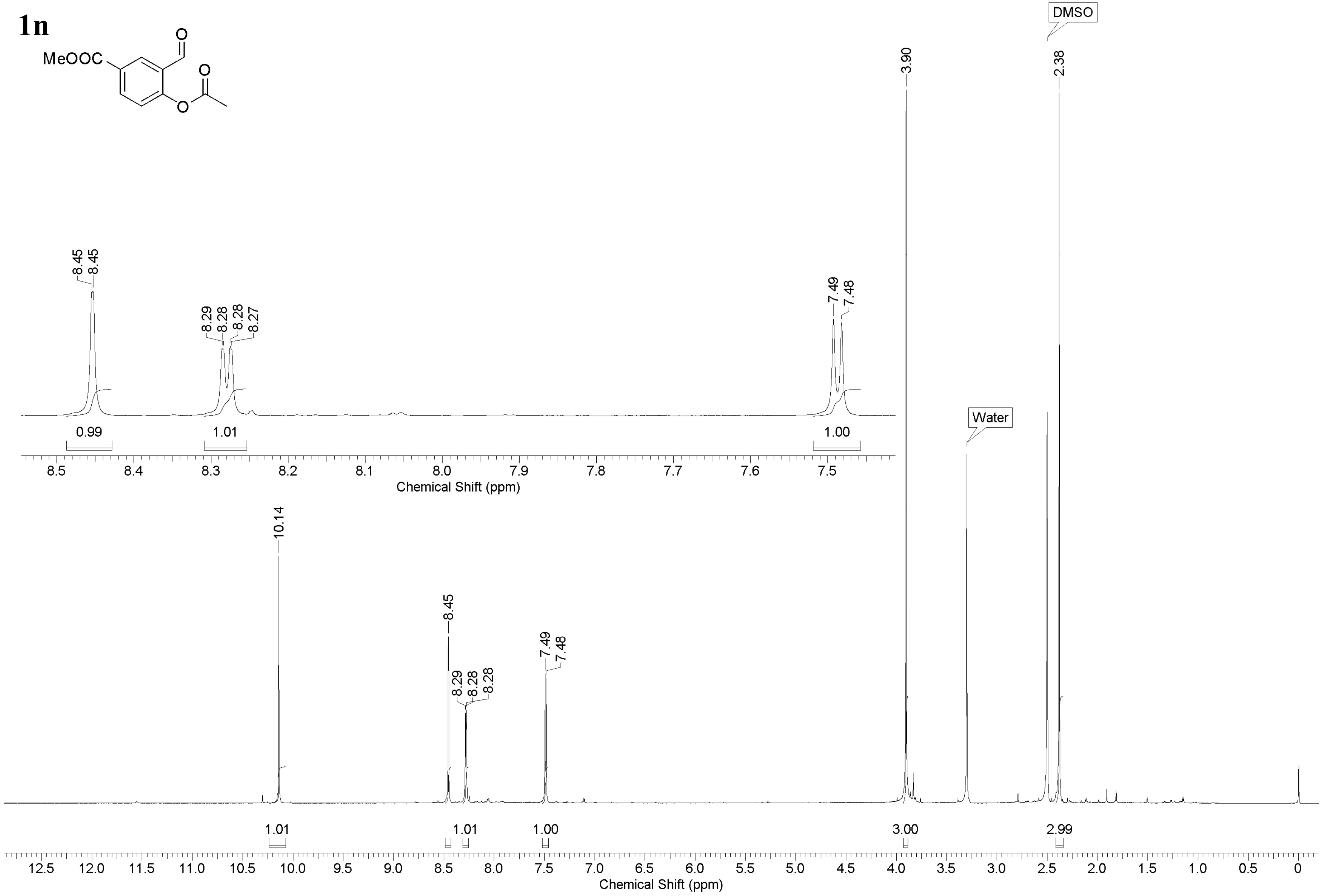

MeOOC
O
O
O
DMSO
Water
3.90
2.38
8.45
8.45
8.29
8.28
8.28
8.27
7.49
7.48
0.99
1.01
1.00
Chemical Shift (ppm)
10.14
8.45
8.29
8.28
8.28
7.49
7.48
1.01
1.01
1.00
3.00
2.99
Chemical Shift (ppm)

1n

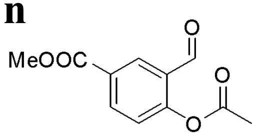
MeOOC
O
O
O

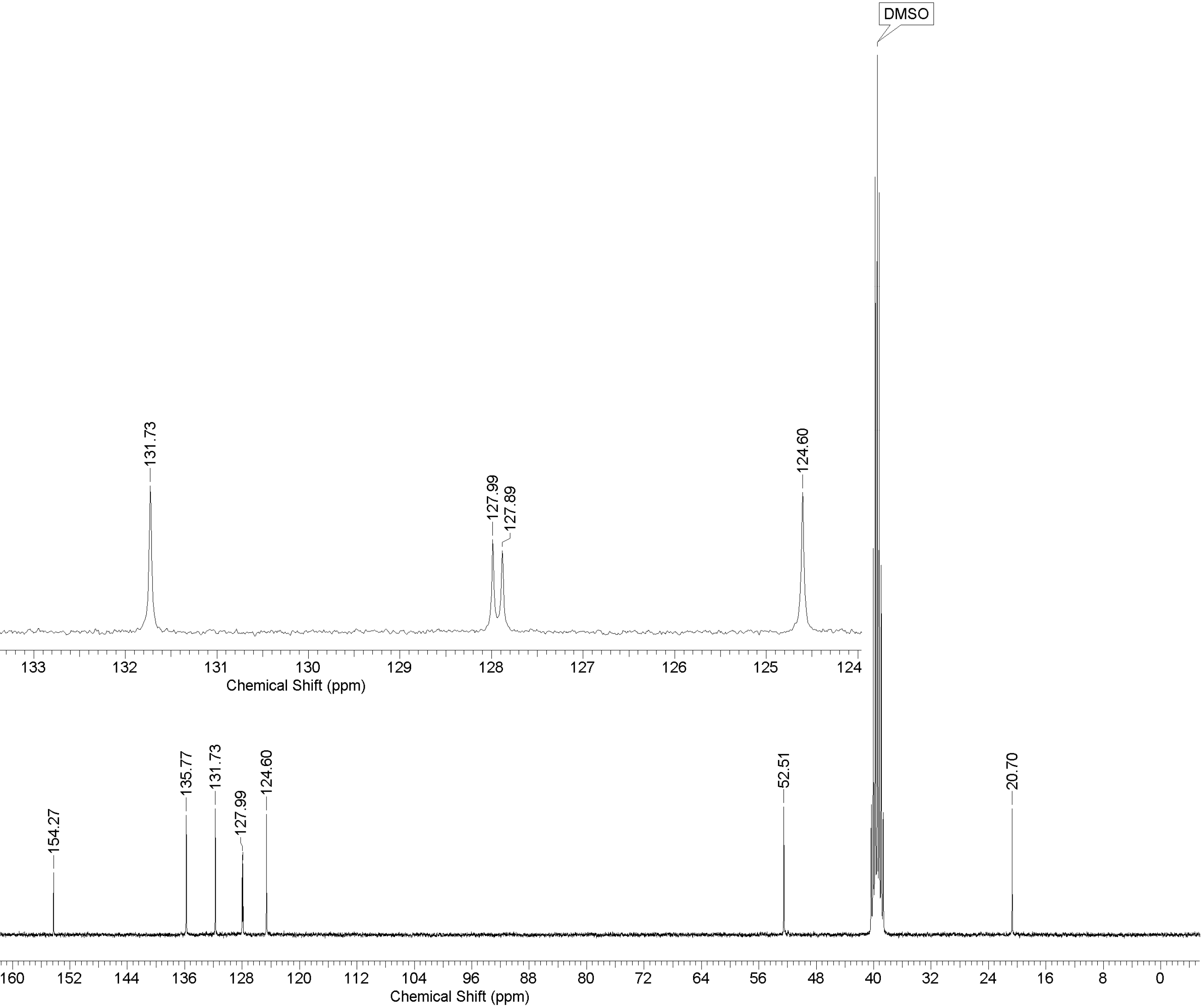
DMSO
135.77
131.73
127.99
127.89
124.60
136
135
134
133
132
131
130
129
128
127
126
125
124
Chemical Shift (ppm)
189.56
168.79
164.78
154.27
135.77
131.73
127.99
124.60
52.51
20.70
192
184
176
168
160
152
144
136
128
120
112
104
96
88
80
72
64
56
48
40
32
24
16
8
0
Chemical Shift (ppm)

2a

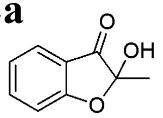
O
OH
O

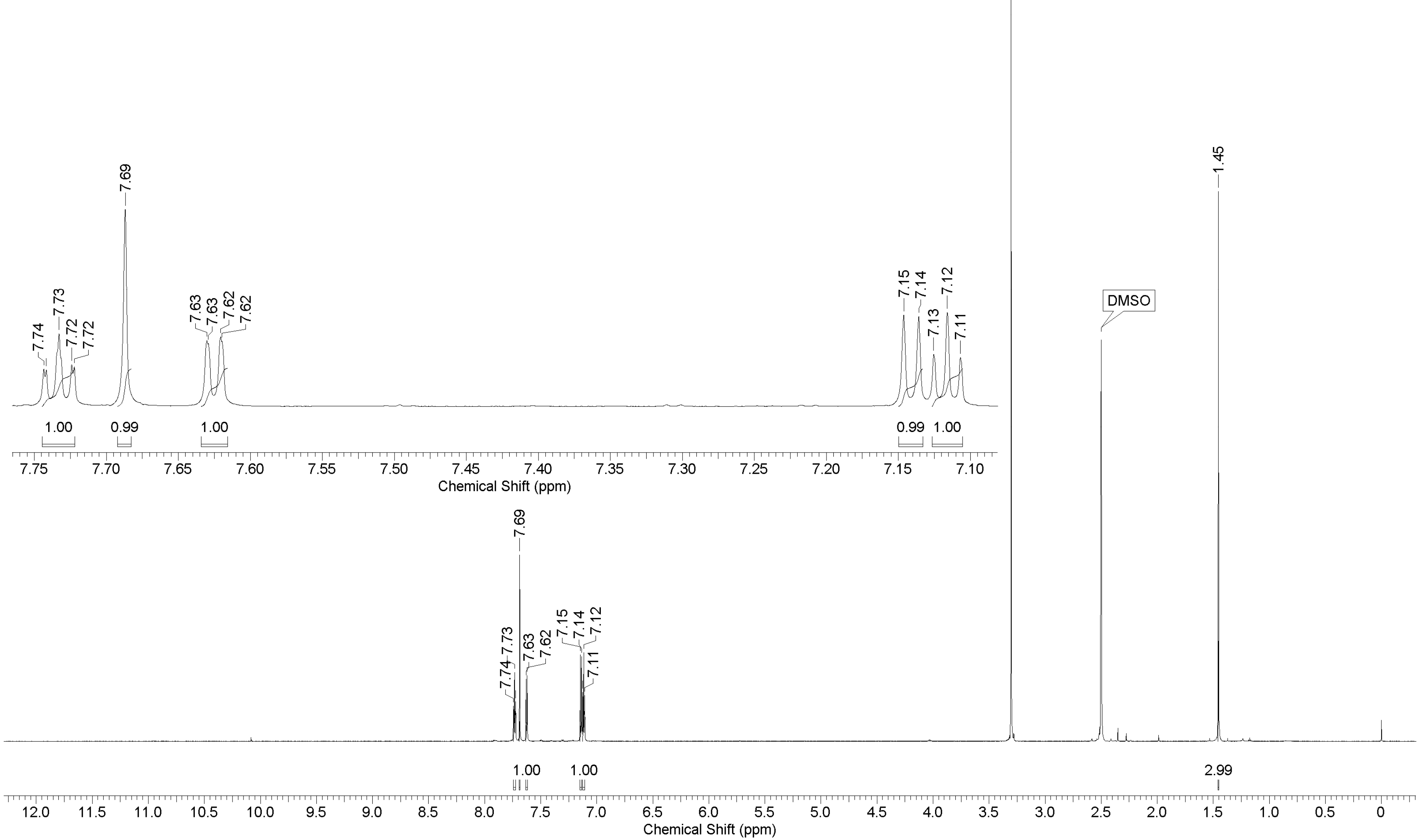
Water
DMSO
7.74
7.73
7.72
7.72
7.69
7.63
7.63
7.62
7.62
7.15
7.14
7.13
7.12
7.11
1.45
1.00
0.99
1.00
0.99
1.00
1.00
1.00
2.99
Chemical Shift (ppm)
Chemical Shift (ppm)

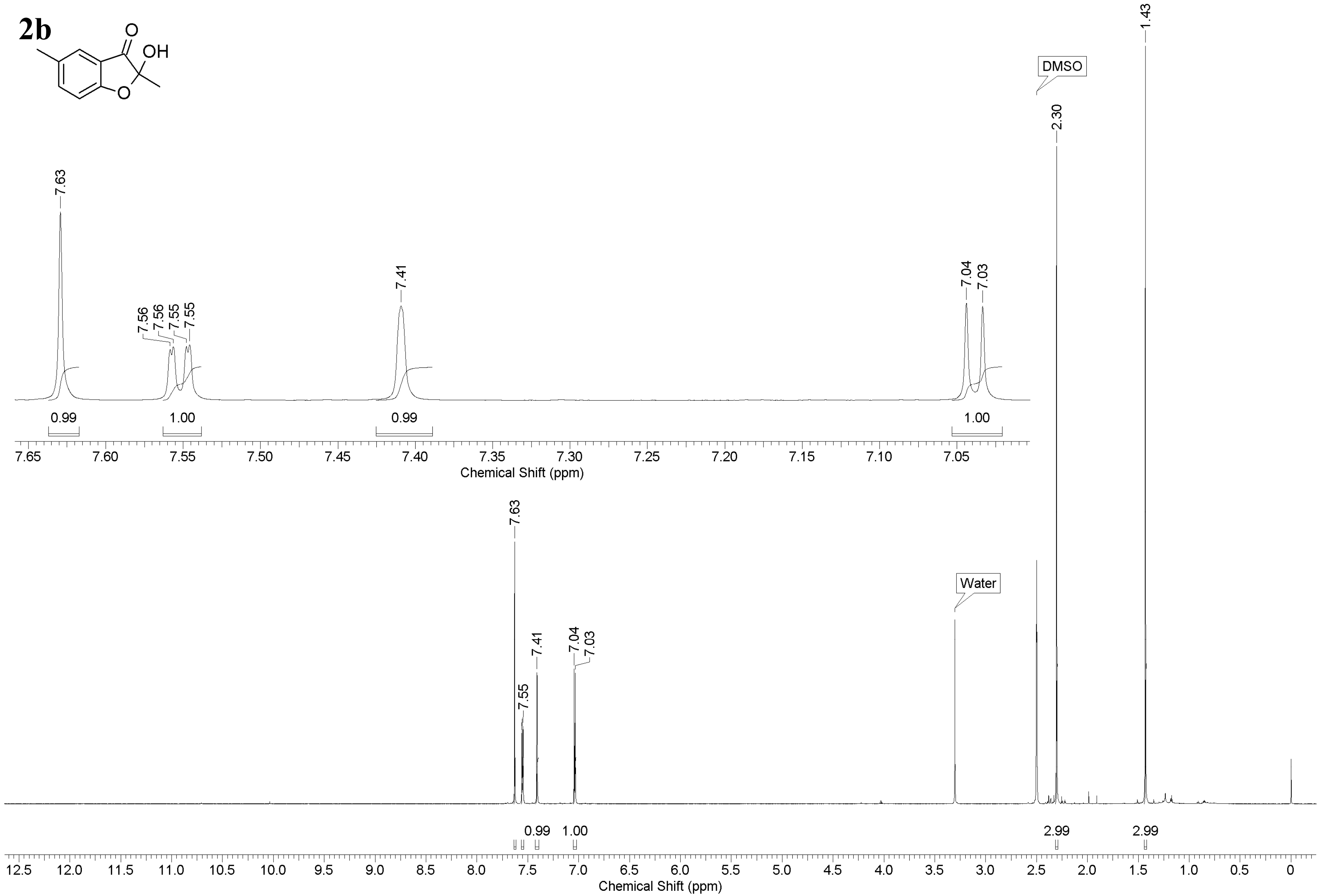
2b
DMSO
Water
7.63
7.56
7.56
7.55
7.55
7.41
7.04
7.03
2.30
1.43
0.99
1.00
0.99
1.00
2.99
2.99
Chemical Shift (ppm)
Chemical Shift (ppm)

2b

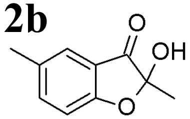
O
OH
O

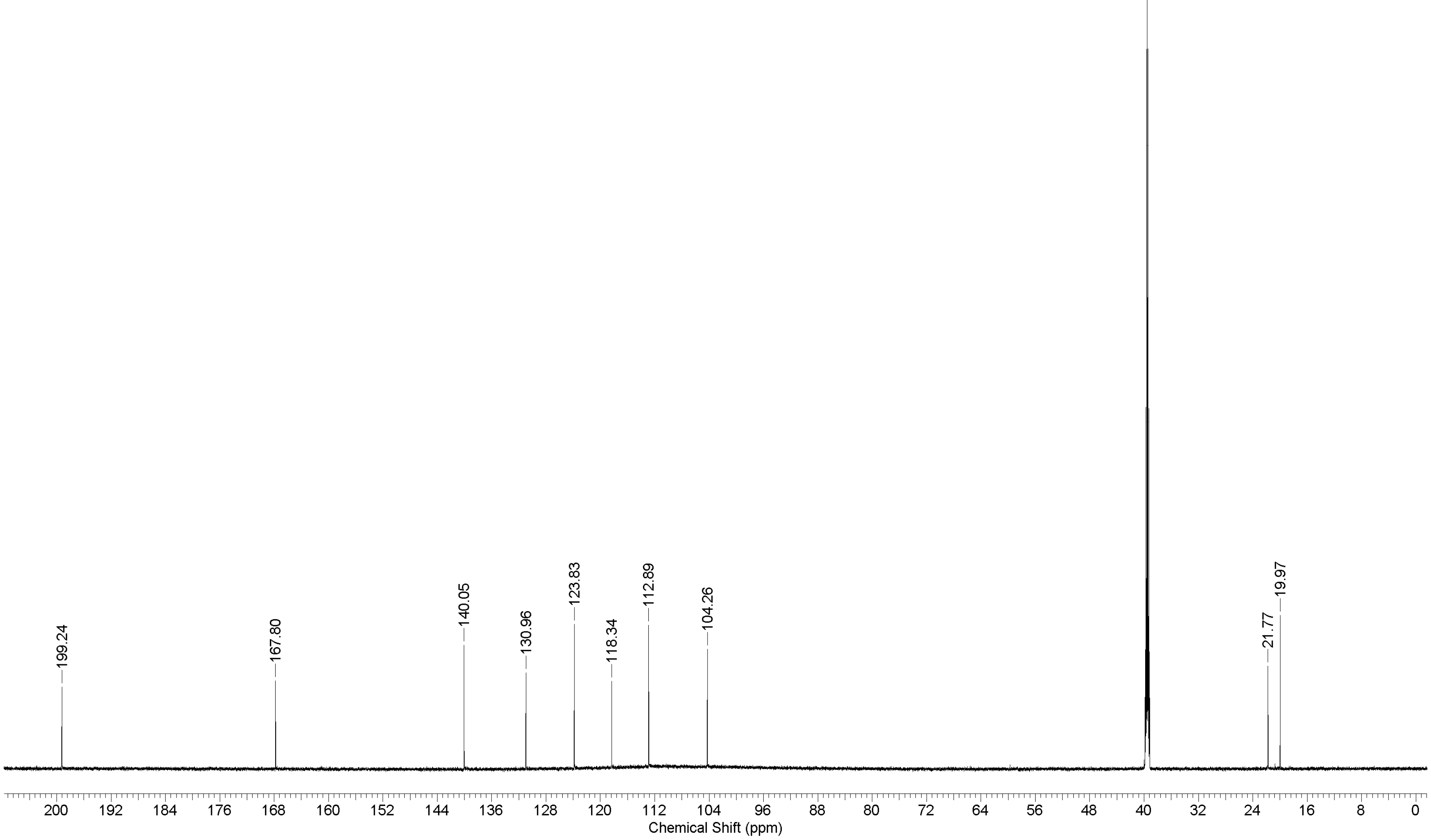
DMSO
199.24
167.80
140.05
130.96
123.83
118.34
112.89
104.26
21.77
19.97
200 192 184 176 168 160 152 144 136 128 120 112 104 96 88 80 72 64 56 48 40 32 24 16 8 0
Chemical Shift (ppm)

2c

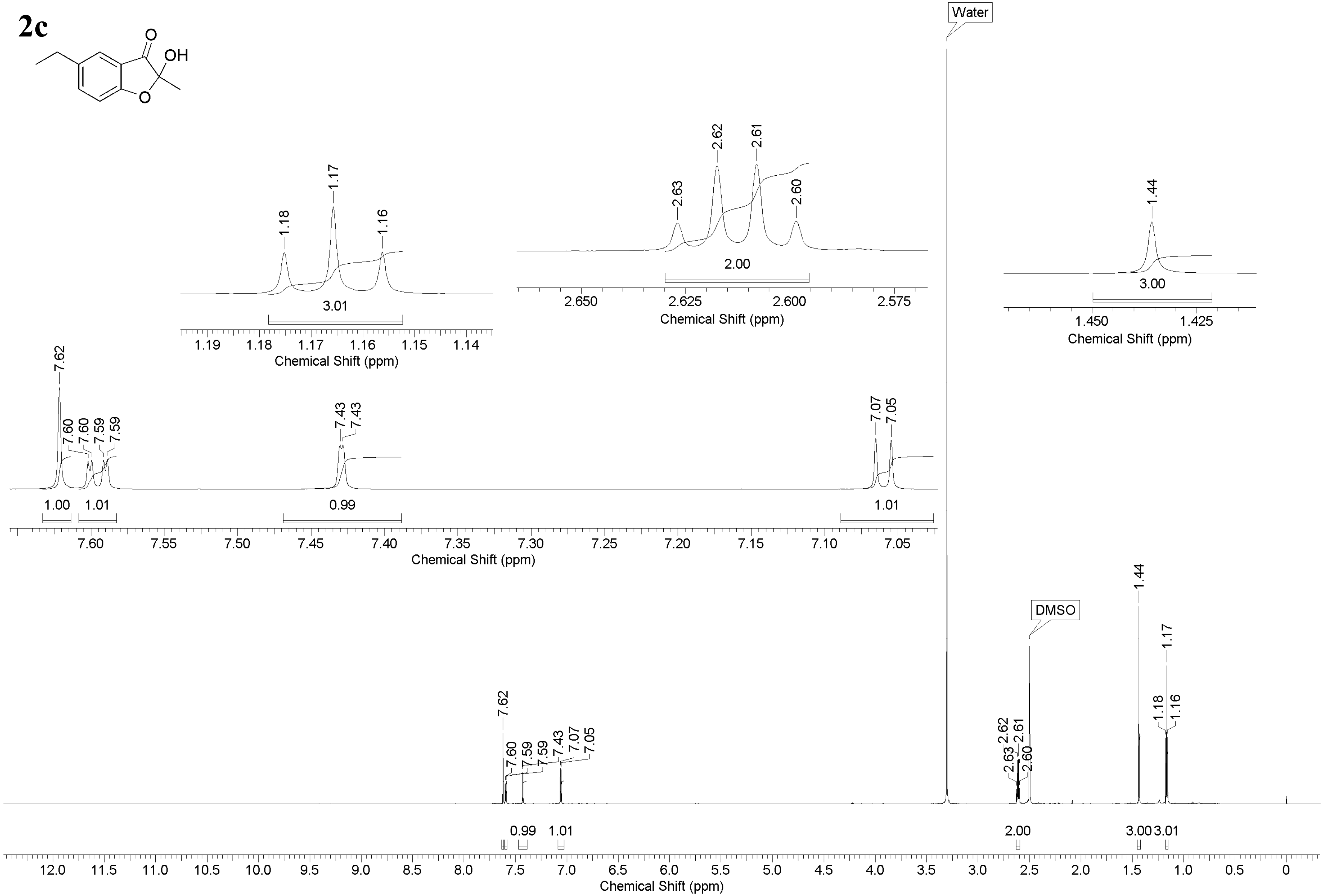
OH
O
O
Water
DMSO
Chemical Shift (ppm)
7.62
7.60
7.60
7.59
7.59
7.43
7.43
7.07
7.05
2.63
2.62
2.61
2.60
1.44
1.18
1.17
1.16
1.00
1.01
0.99
1.01
2.00
3.00
3.01

2d

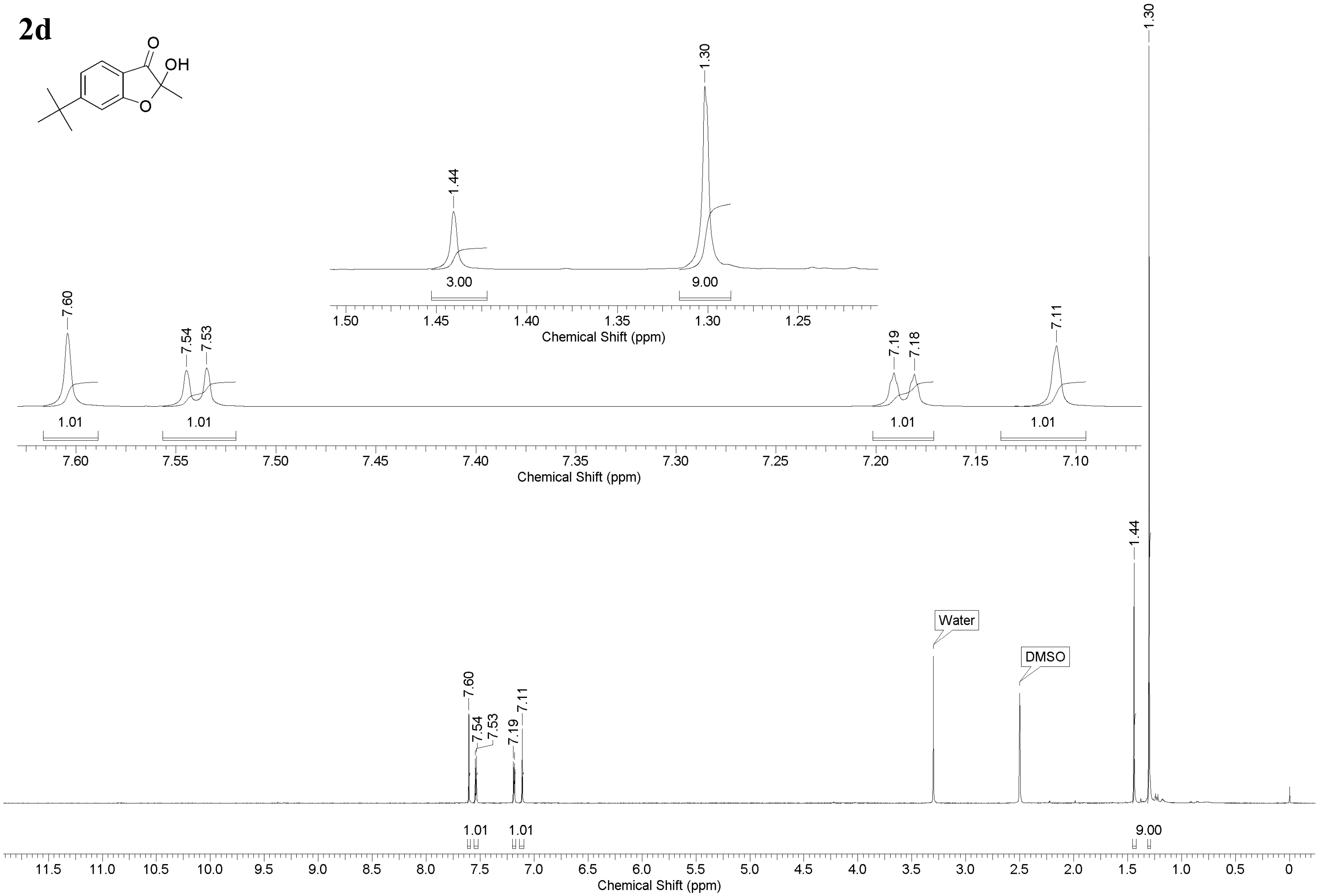
OH
O
O
1.30
1.44
3.00
9.00
1.50 1.45 1.40 1.35 1.30 1.25
Chemical Shift (ppm)
7.60
7.54
7.53
7.19
7.18
7.11
1.01 1.01 1.01 1.01
7.60 7.55 7.50 7.45 7.40 7.35 7.30 7.25 7.20 7.15 7.10
Chemical Shift (ppm)
Water
DMSO
11.5 11.0 10.5 10.0 9.5 9.0 8.5 8.0 7.5 7.0 6.5 6.0 5.5 5.0 4.5 4.0 3.5 3.0 2.5 2.0 1.5 1.0 0.5 0
Chemical Shift (ppm)

2d

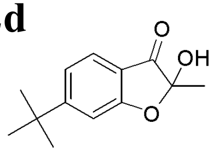

O
OH
O

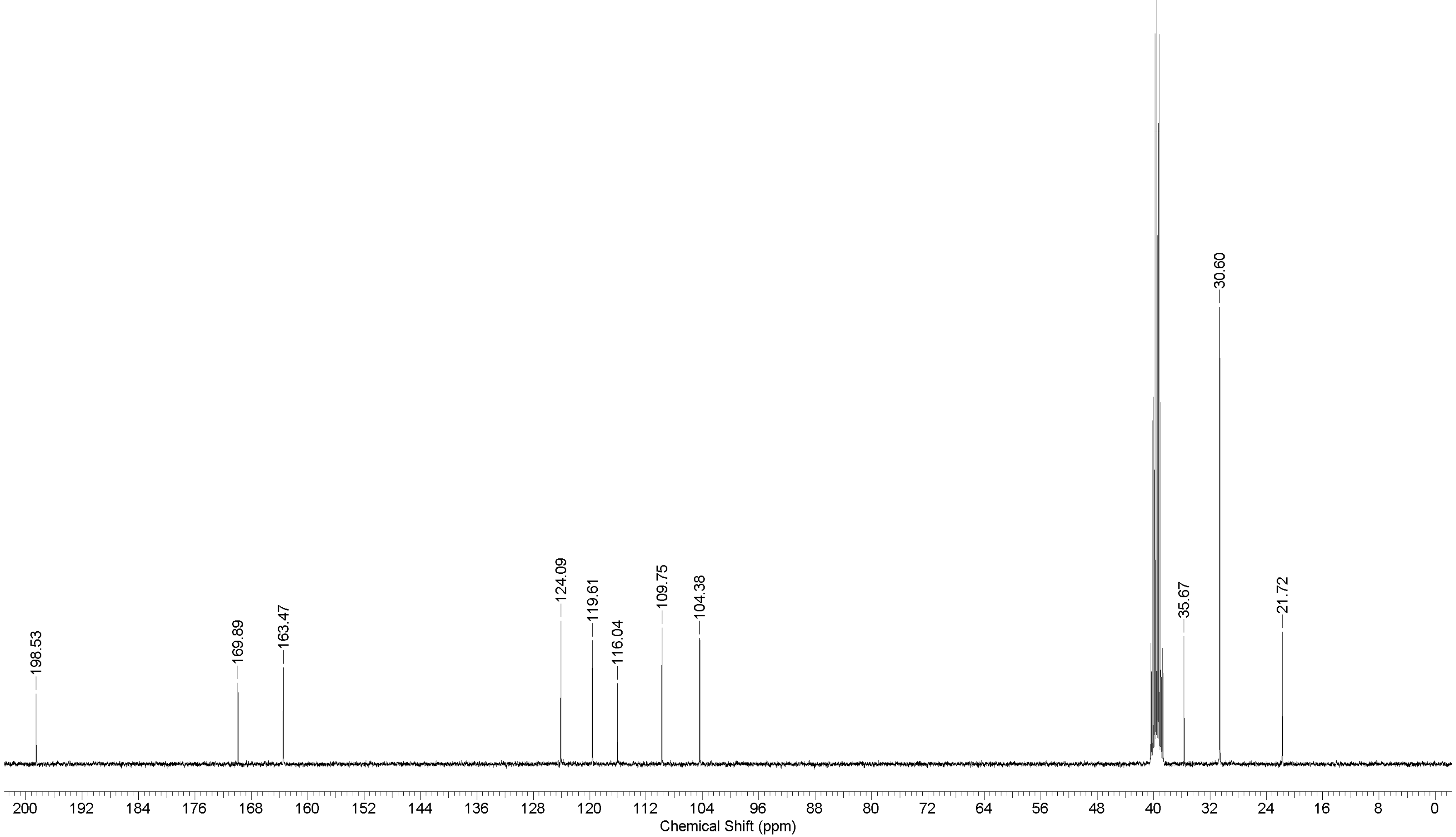

DMSO
198.53
169.89
163.47
124.09
119.61
116.04
109.75
104.38
35.67
30.60
21.72
200 192 184 176 168 160 152 144 136 128 120 112 104 96 88 80 72 64 56 48 40 32 24 16 8 0
Chemical Shift (ppm)

2e

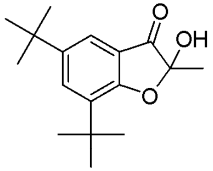

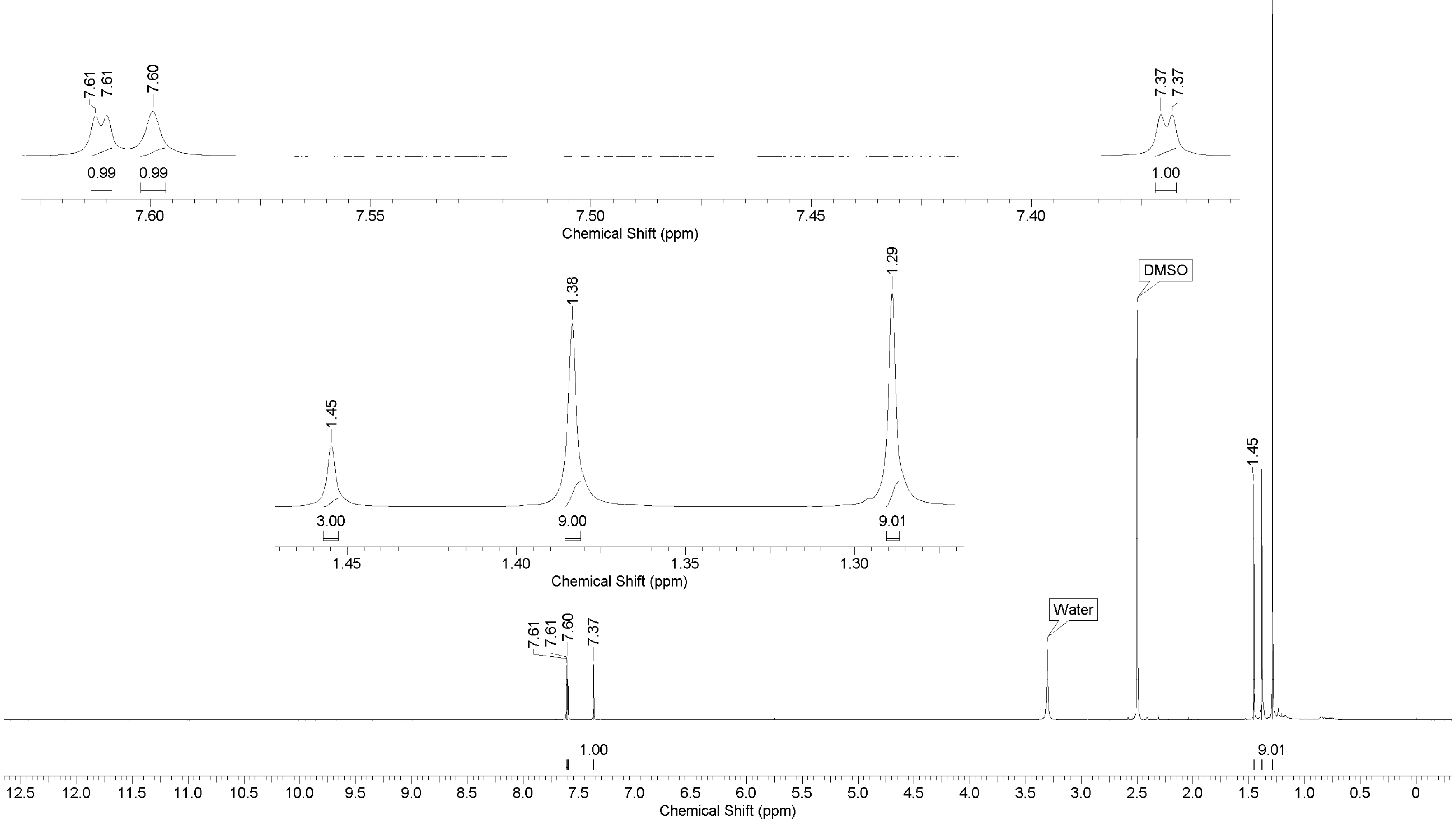
7.61
7.61
7.60
7.37
7.37
0.99
0.99
1.00
7.60
7.55
7.50
7.45
7.40
Chemical Shift (ppm)
1.45
1.38
1.29
3.00
9.00
9.01
1.45
1.40
1.35
1.30
Chemical Shift (ppm)
DMSO
Water
7.61
7.61
7.60
7.37
1.00
9.01
12.5
12.0
11.5
11.0
10.5
10.0
9.5
9.0
8.5
8.0
7.5
7.0
6.5
6.0
5.5
5.0
4.5
4.0
3.5
3.0
2.5
2.0
1.5
1.0
0.5
0
Chemical Shift (ppm)

2e

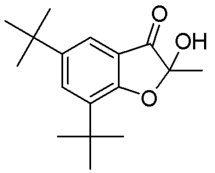

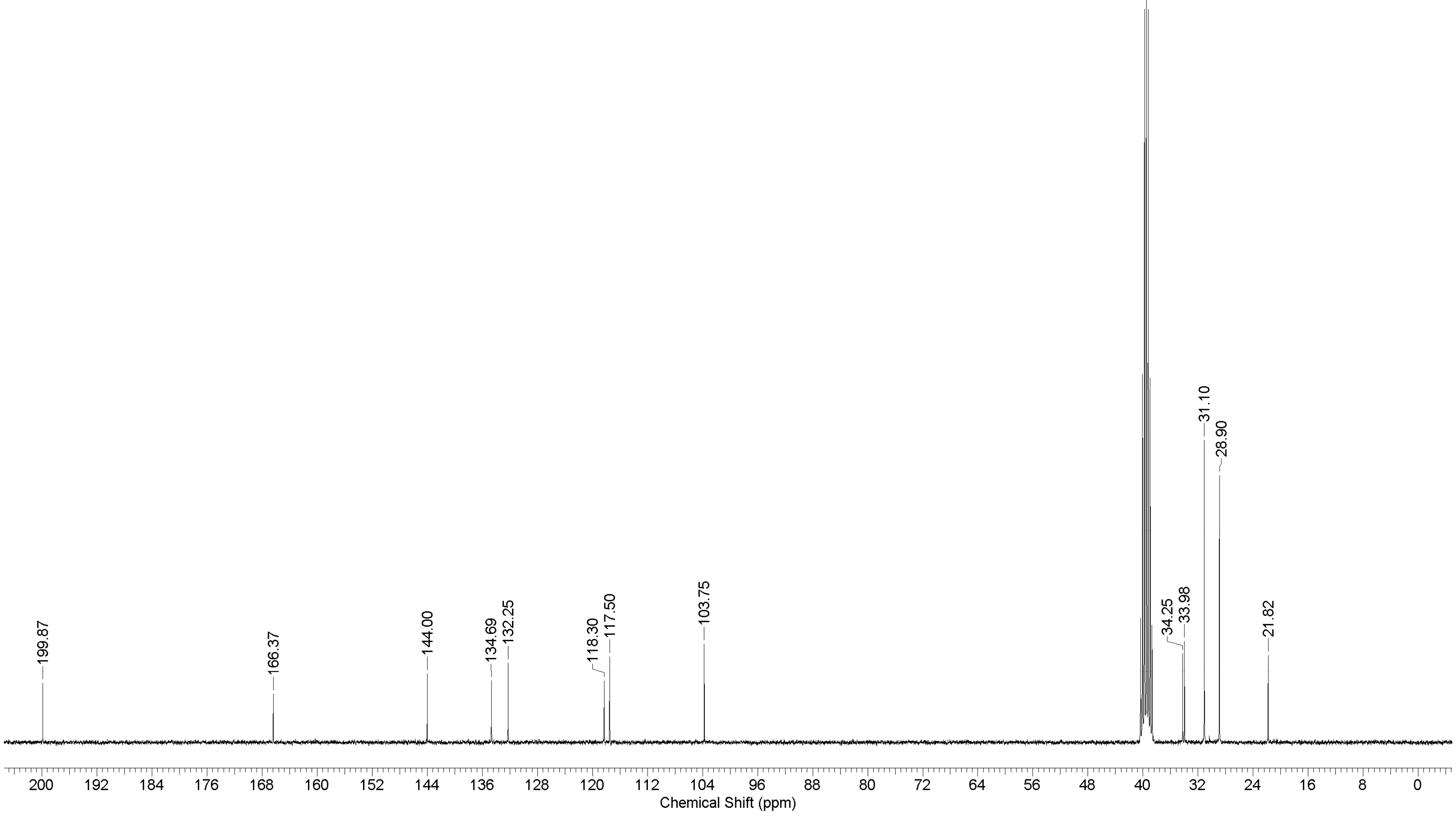

DMSO
199.87
166.37
144.00
134.69
132.25
118.30
117.50
103.75
34.25
33.98
31.10
28.90
21.82
200
192
184
176
168
160
152
144
136
128
120
112
104
96
88
80
72
64
56
48
40
32
24
16
8
0
Chemical Shift (ppm)

2f

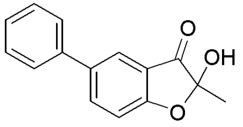

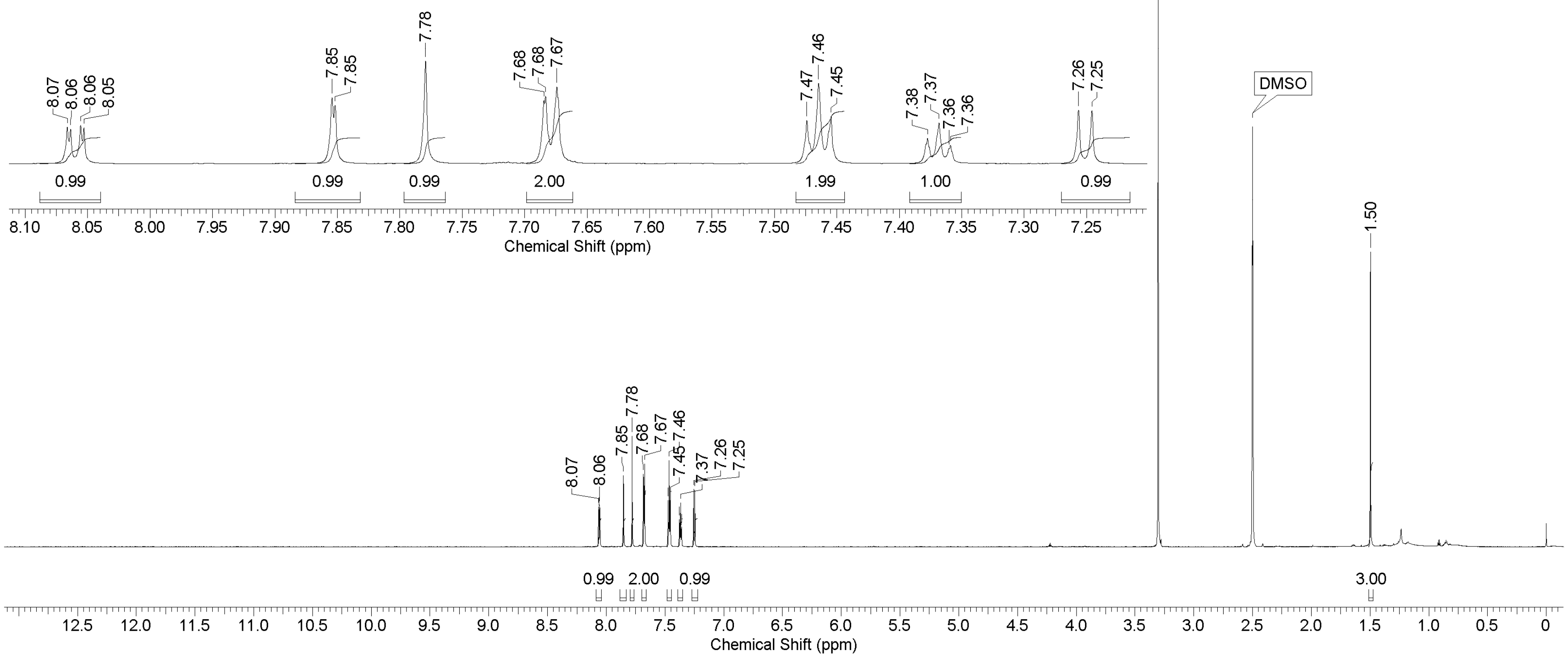

Water
DMSO
8.07
8.06
8.06
8.05
7.85
7.85
7.78
7.68
7.68
7.67
7.47
7.46
7.45
7.38
7.37
7.36
7.36
7.26
7.25
0.99
0.99
0.99
2.00
1.99
1.00
0.99
Chemical Shift (ppm)
1.50
3.00
Chemical Shift (ppm)

2f

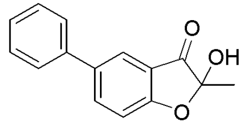

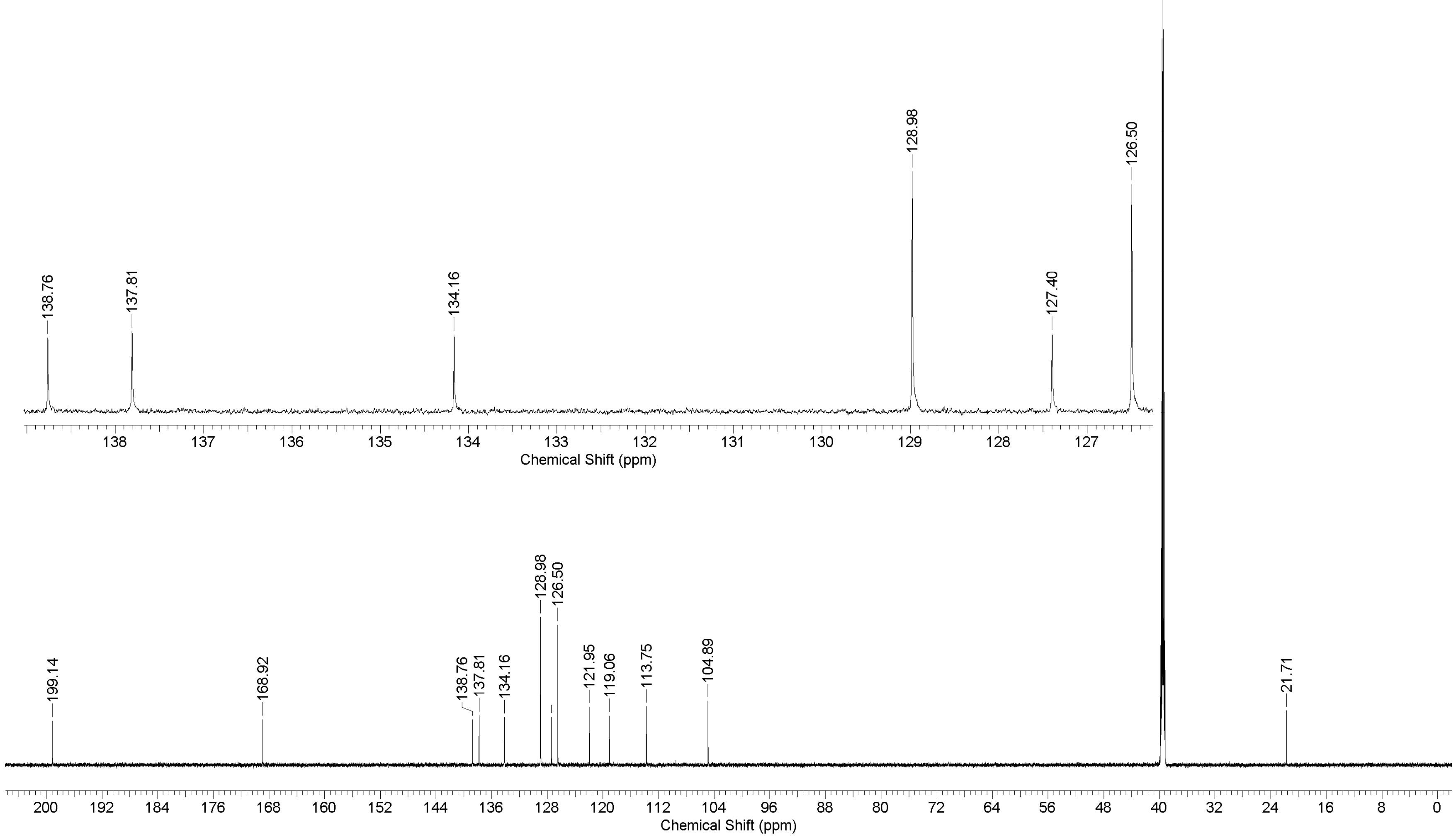
DMSO
138.76
137.81
134.16
128.98
127.40
126.50
138
137
136
135
134
133
132
131
130
129
128
127
Chemical Shift (ppm)
199.14
168.92
138.76
137.81
134.16
128.98
126.50
121.95
119.06
113.75
104.89
21.71
200
192
184
176
168
160
152
144
136
128
120
112
104
96
88
80
72
64
56
48
40
32
24
16
8
0
Chemical Shift (ppm)

2g

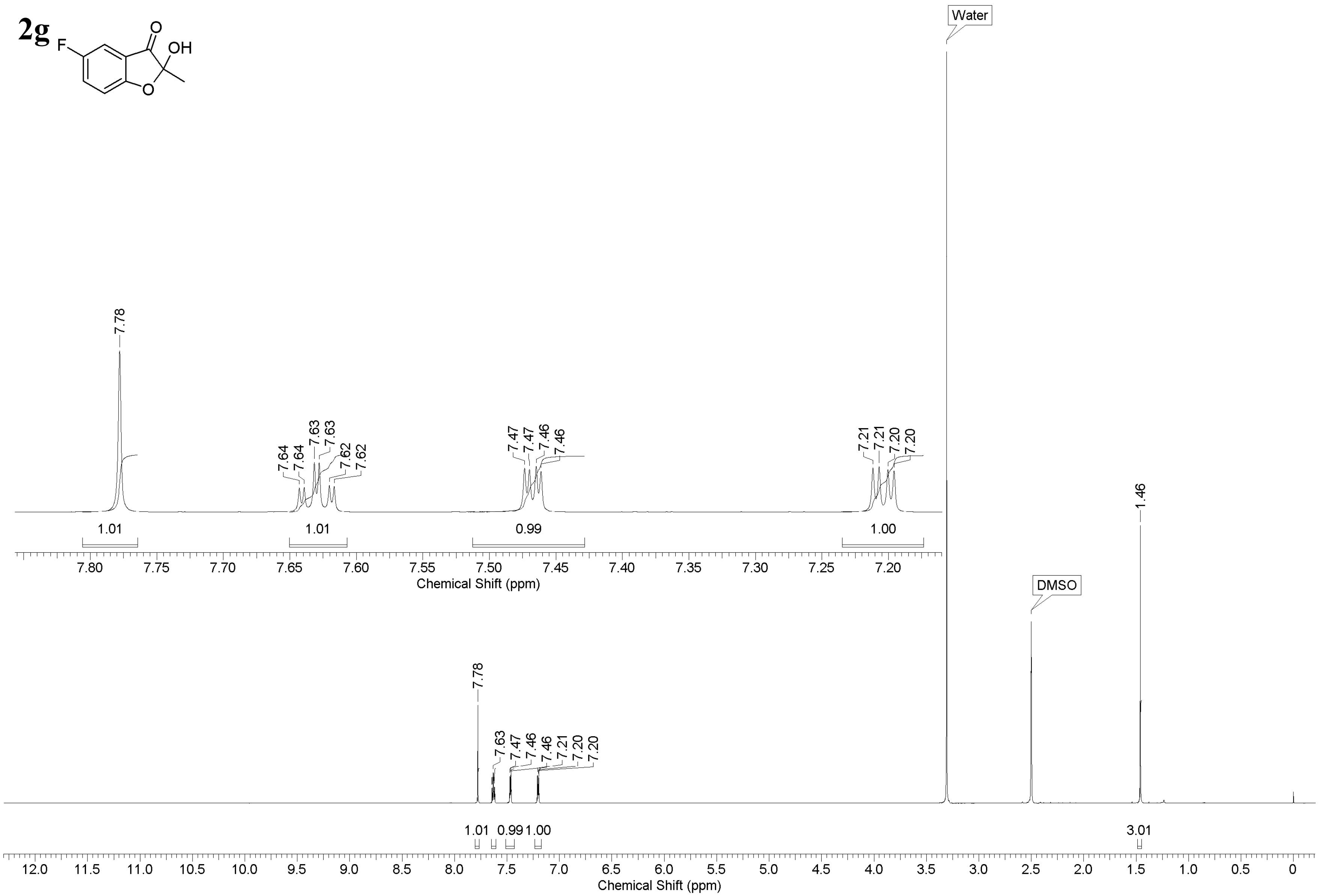
F
O
OH
O
Water
DMSO
7.78
7.64
7.64
7.63
7.63
7.62
7.62
7.47
7.47
7.46
7.46
7.21
7.21
7.20
7.20
1.46
1.01
1.01
0.99
1.00
3.01
Chemical Shift (ppm)
Chemical Shift (ppm)

2h

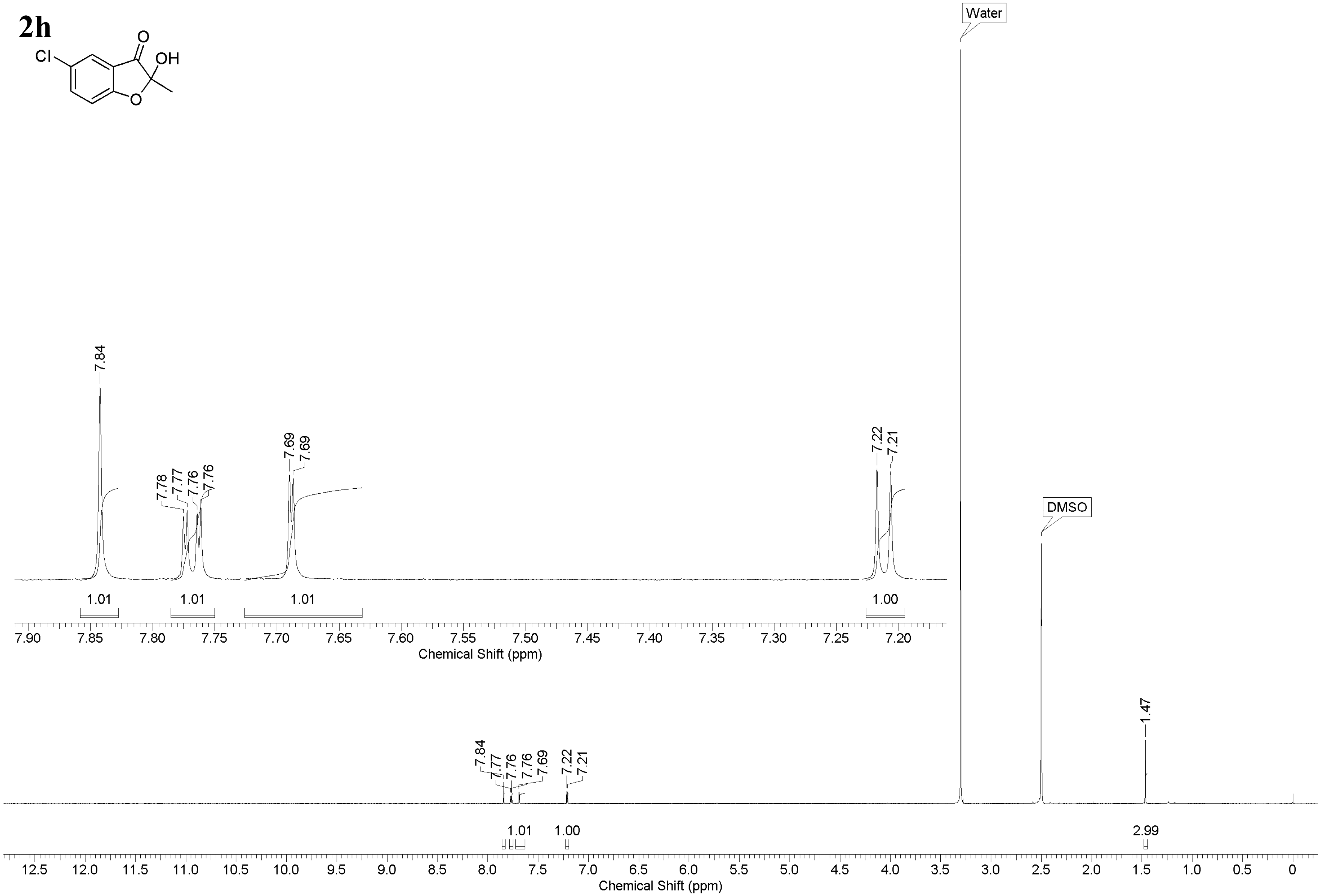
Cl
O
OH
O
Water
DMSO
7.84
7.78
7.77
7.76
7.76
7.69
7.69
7.22
7.21
1.01
1.01
1.01
1.00
Chemical Shift (ppm)
1.47
1.01
1.00
2.99
Chemical Shift (ppm)

2h

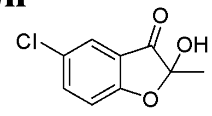

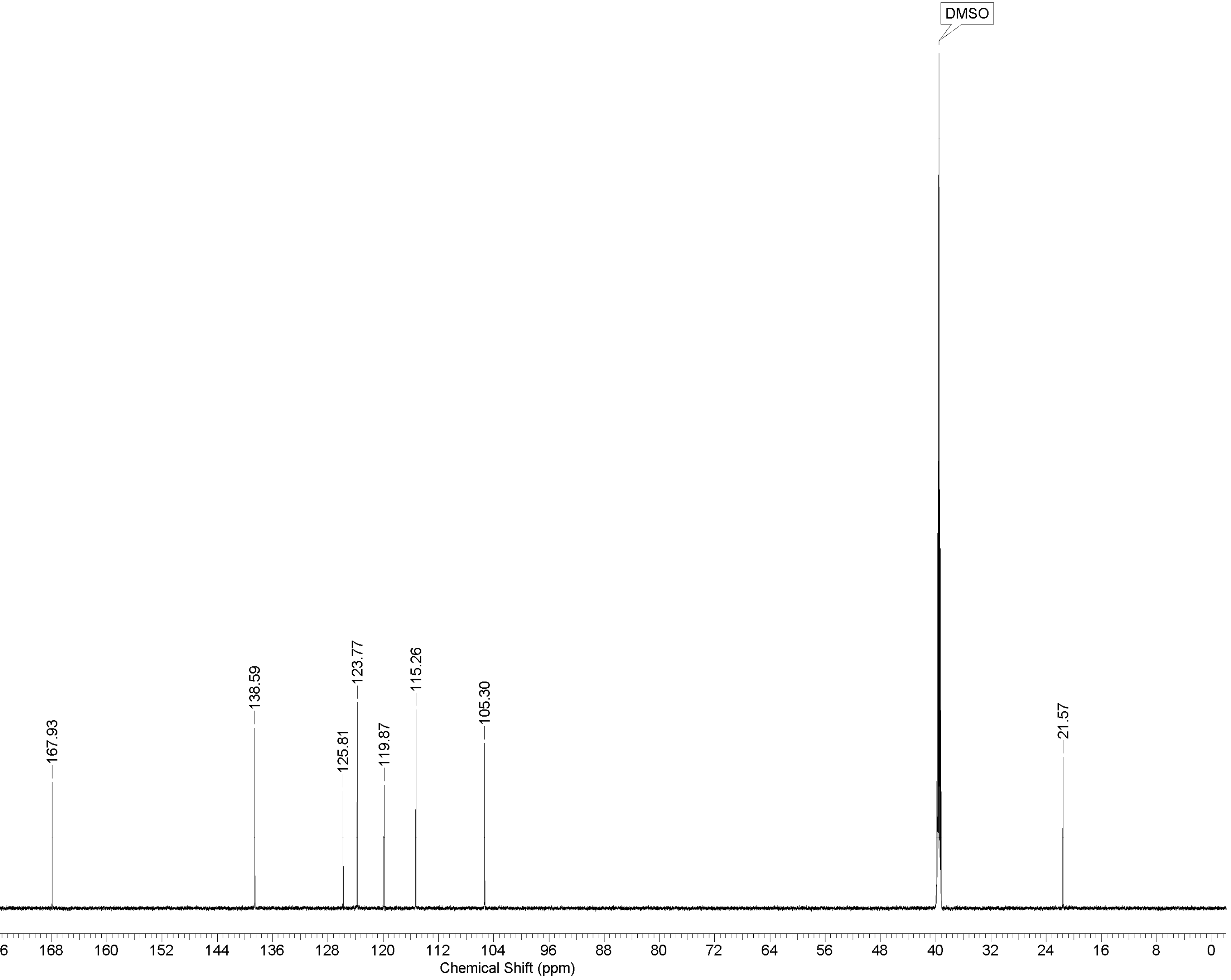

DMSO
198.27
167.93
138.59
125.81
123.77
119.87
115.26
105.30
21.57
200 192 184 176 168 160 152 144 136 128 120 112 104 96 88 80 72 64 56 48 40 32 24 16 8 0
Chemical Shift (ppm)

2i

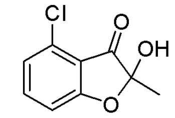
Cl
O
OH
O

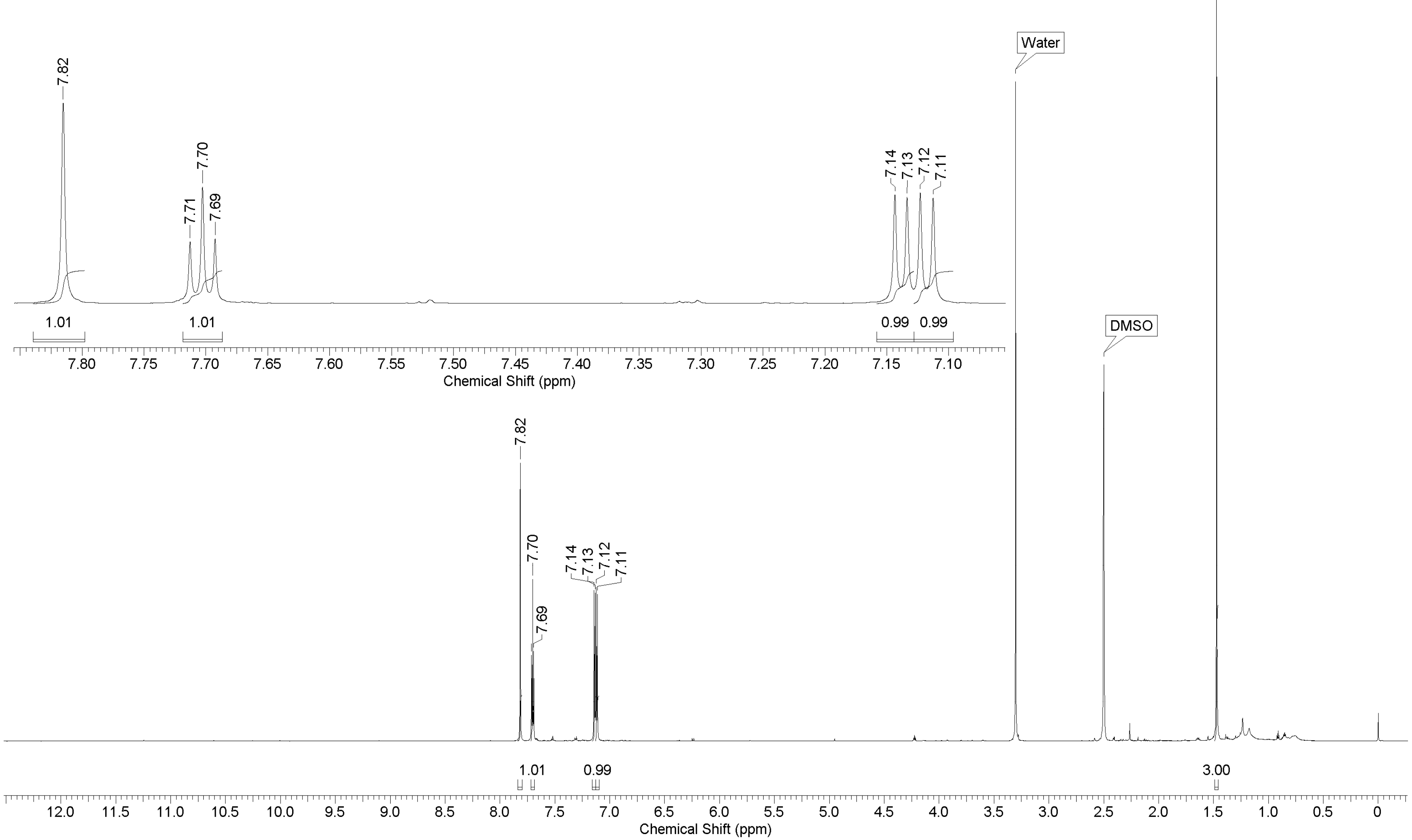
7.82
7.70
7.71
7.69
7.14
7.13
7.12
7.11
1.01
1.01
0.99
0.99
7.80
7.75
7.70
7.65
7.60
7.55
7.50
7.45
7.40
7.35
7.30
7.25
7.20
7.15
7.10
Chemical Shift (ppm)
Water
DMSO
1.47
3.00
12.0
11.5
11.0
10.5
10.0
9.5
9.0
8.5
8.0
7.5
7.0
6.5
6.0
5.5
5.0
4.5
4.0
3.5
3.0
2.5
2.0
1.5
1.0
0.5
0
Chemical Shift (ppm)

2i

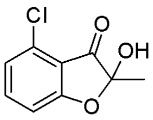
Cl
O
OH
O

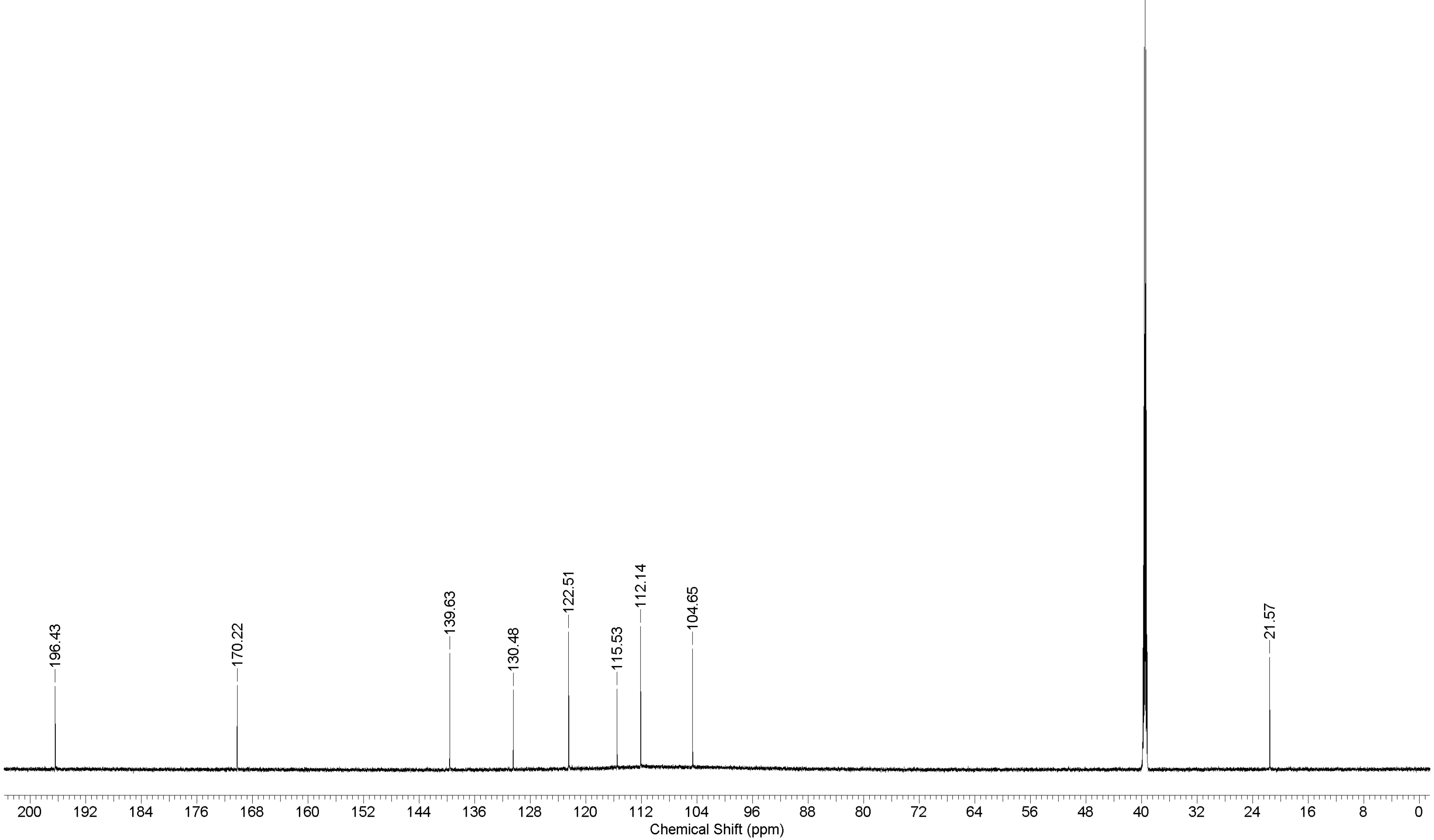
DMSO
196.43
170.22
139.63
130.48
122.51
115.53
112.14
104.65
21.57
200 192 184 176 168 160 152 144 136 128 120 112 104 96 88 80 72 64 56 48 40 32 24 16 8 0
Chemical Shift (ppm)

2j

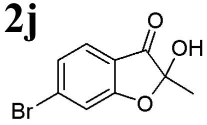

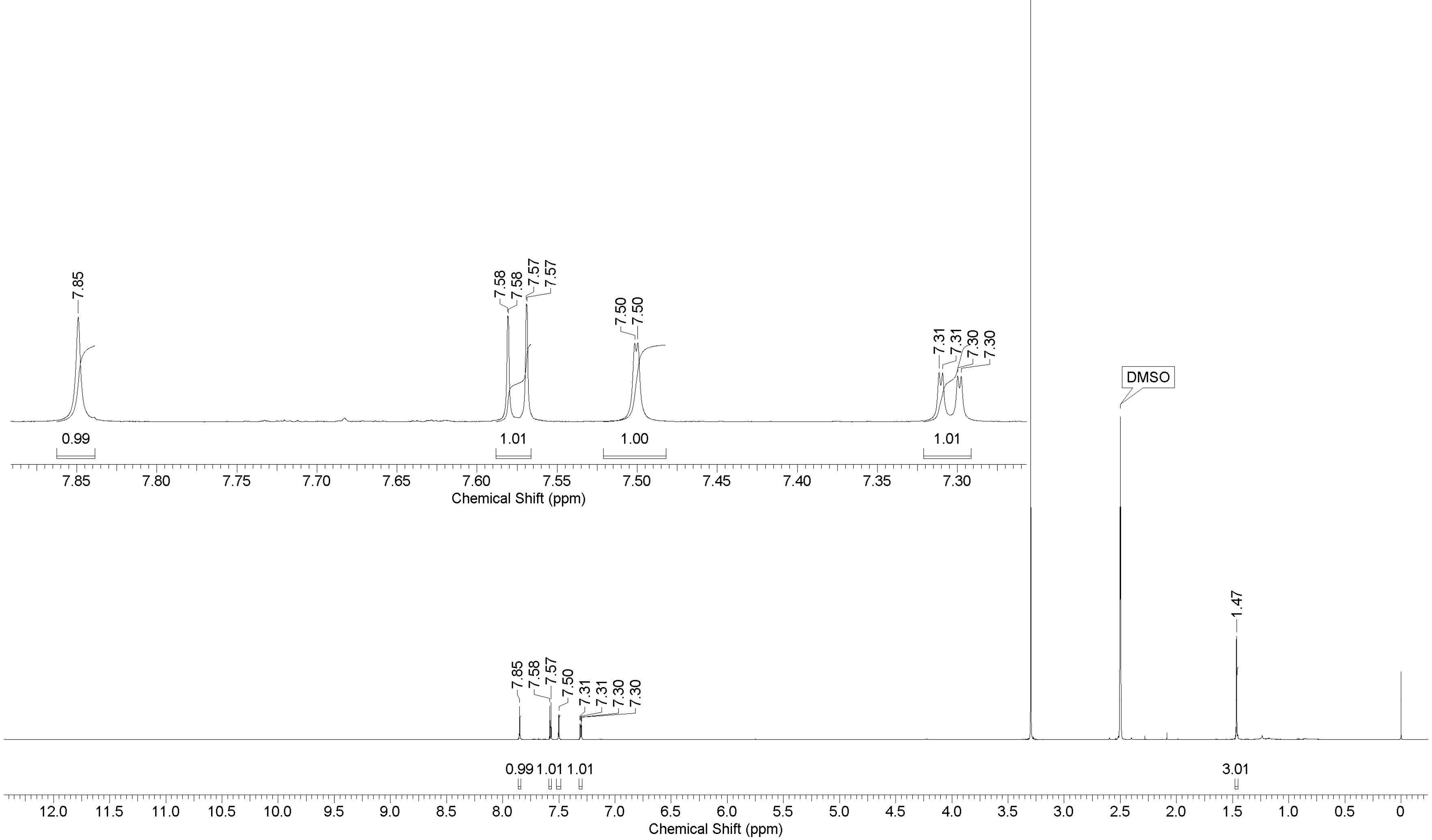

Water
DMSO
7.85
7.58
7.58
7.57
7.57
7.50
7.50
7.31
7.31
7.30
7.30
0.99
1.01
1.00
1.01
7.85
7.80
7.75
7.70
7.65
7.60
7.55
7.50
7.45
7.40
7.35
7.30
Chemical Shift (ppm)
1.47
0.99 1.01 1.01
3.01
12.0
11.5
11.0
10.5
10.0
9.5
9.0
8.5
8.0
7.5
7.0
6.5
6.0
5.5
5.0
4.5
4.0
3.5
3.0
2.5
2.0
1.5
1.0
0.5
0
Chemical Shift (ppm)

2j

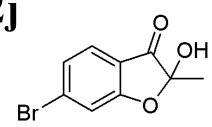
O
OH
Br
O

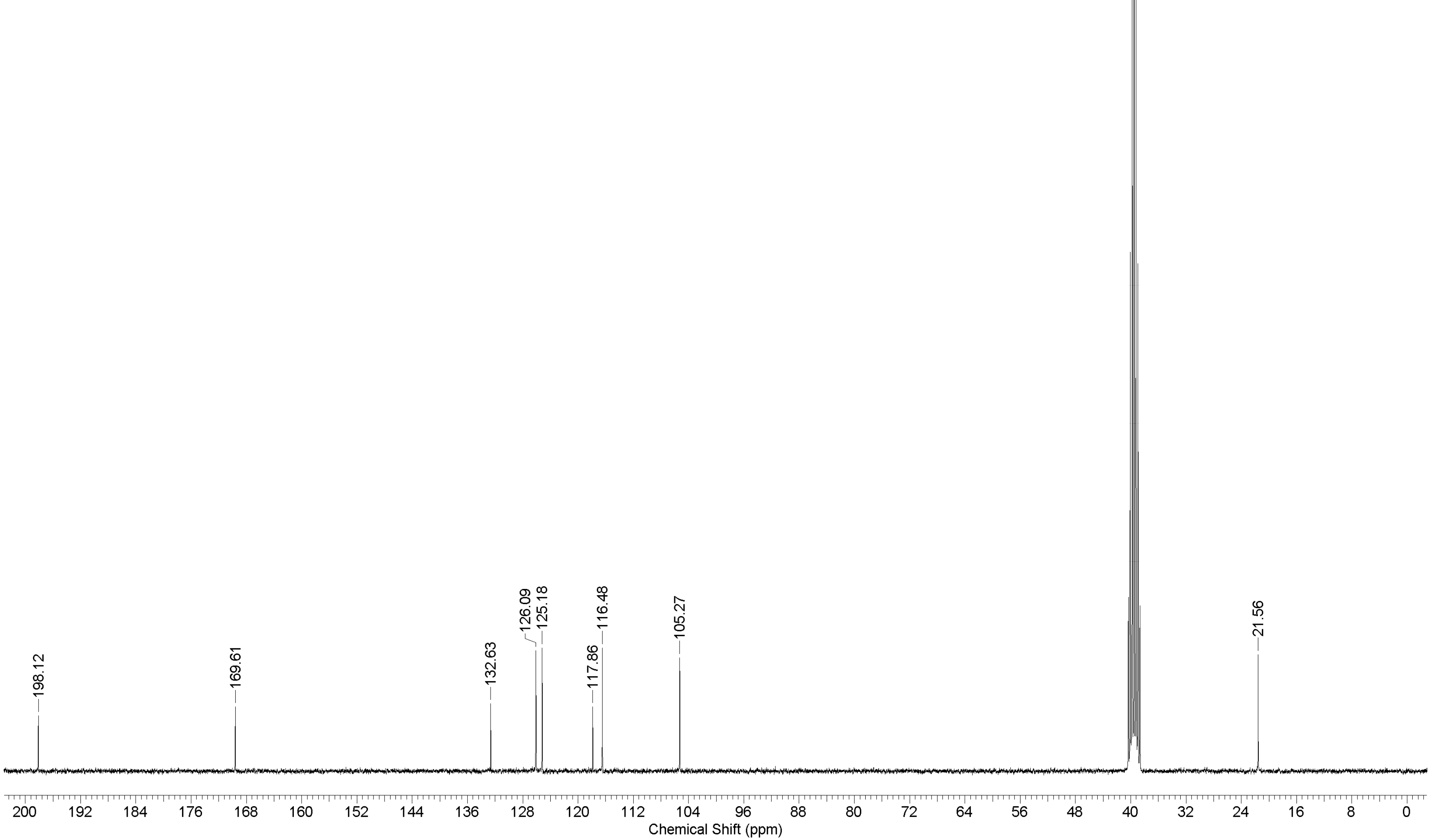
DMSO
198.12
169.61
132.63
126.09
125.18
117.86
116.48
105.27
21.56
200 192 184 176 168 160 152 144 136 128 120 112 104 96 88 80 72 64 56 48 40 32 24 16 8 0
Chemical Shift (ppm)

2k

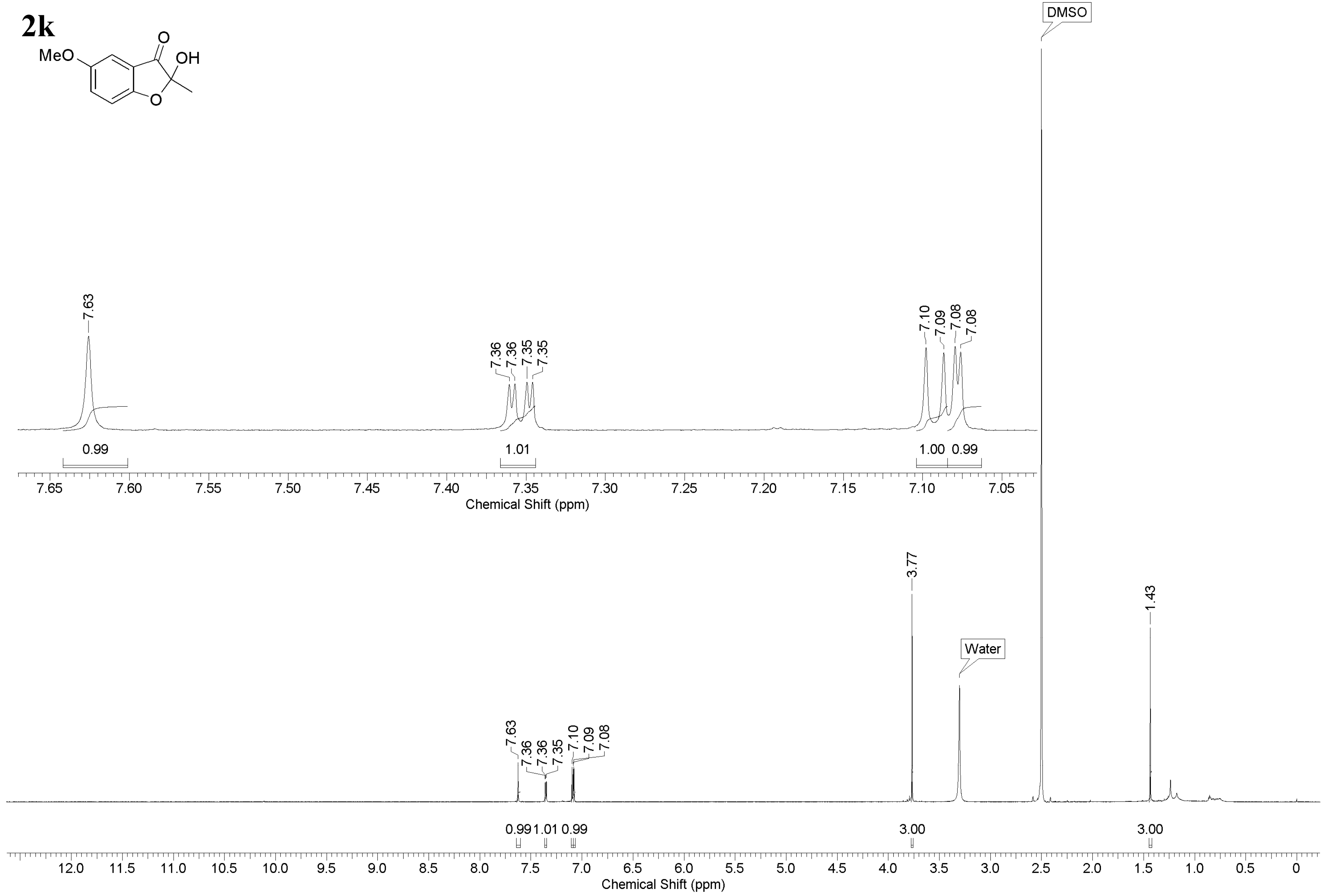
MeO
O
OH
O
DMSO
7.63
7.36
7.36
7.35
7.35
7.10
7.09
7.08
7.08
0.99
1.01
1.00
0.99
Chemical Shift (ppm)
3.77
Water
1.43
0.99
1.01
0.99
3.00
3.00
Chemical Shift (ppm)

2k

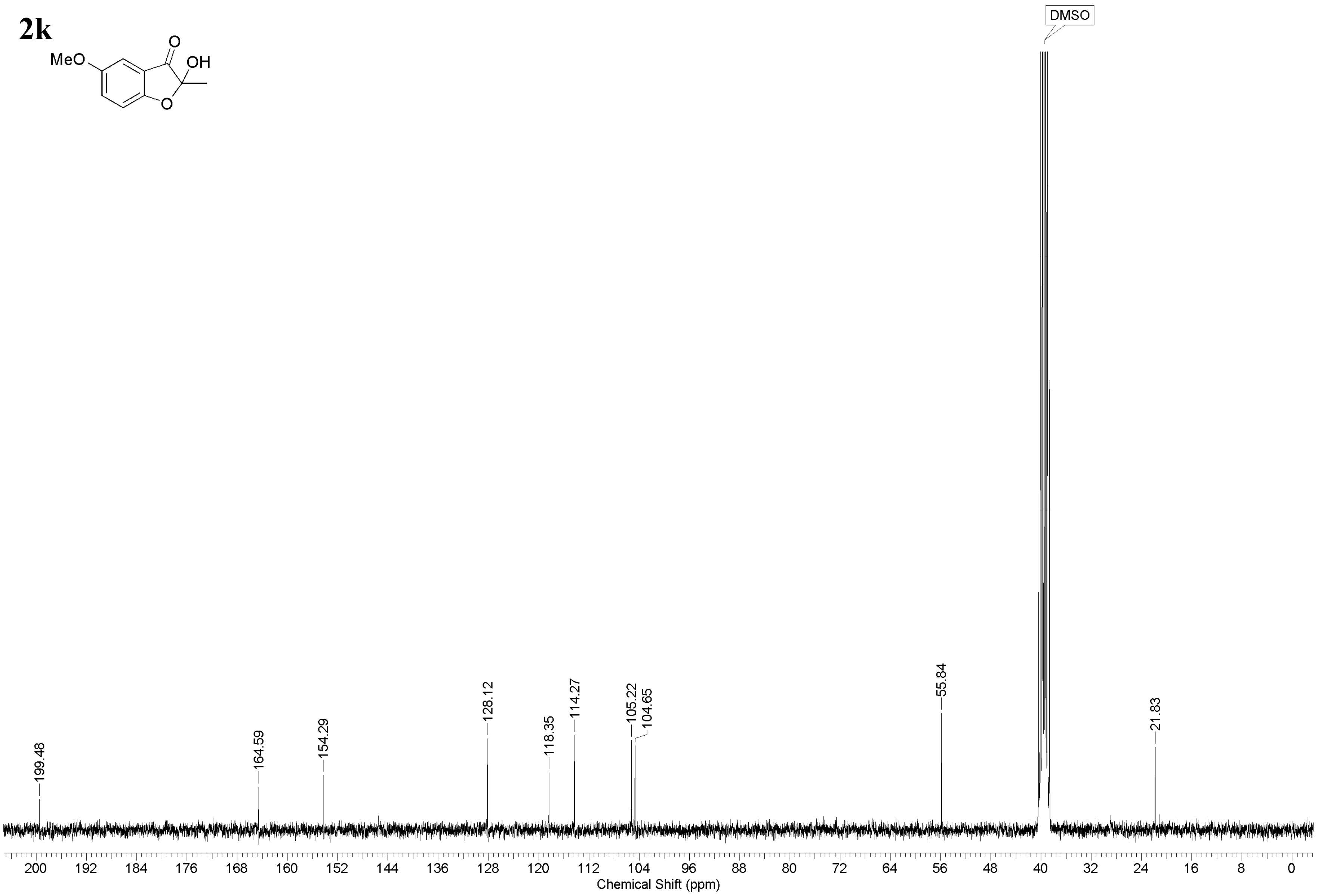
MeO
O
OH
O
DMSO
199.48
164.59
154.29
128.12
118.35
114.27
105.22
104.65
55.84
21.83
200
192
184
176
168
160
152
144
136
128
120
112
104
96
88
80
72
64
56
48
40
32
24
16
8
0
Chemical Shift (ppm)

2l

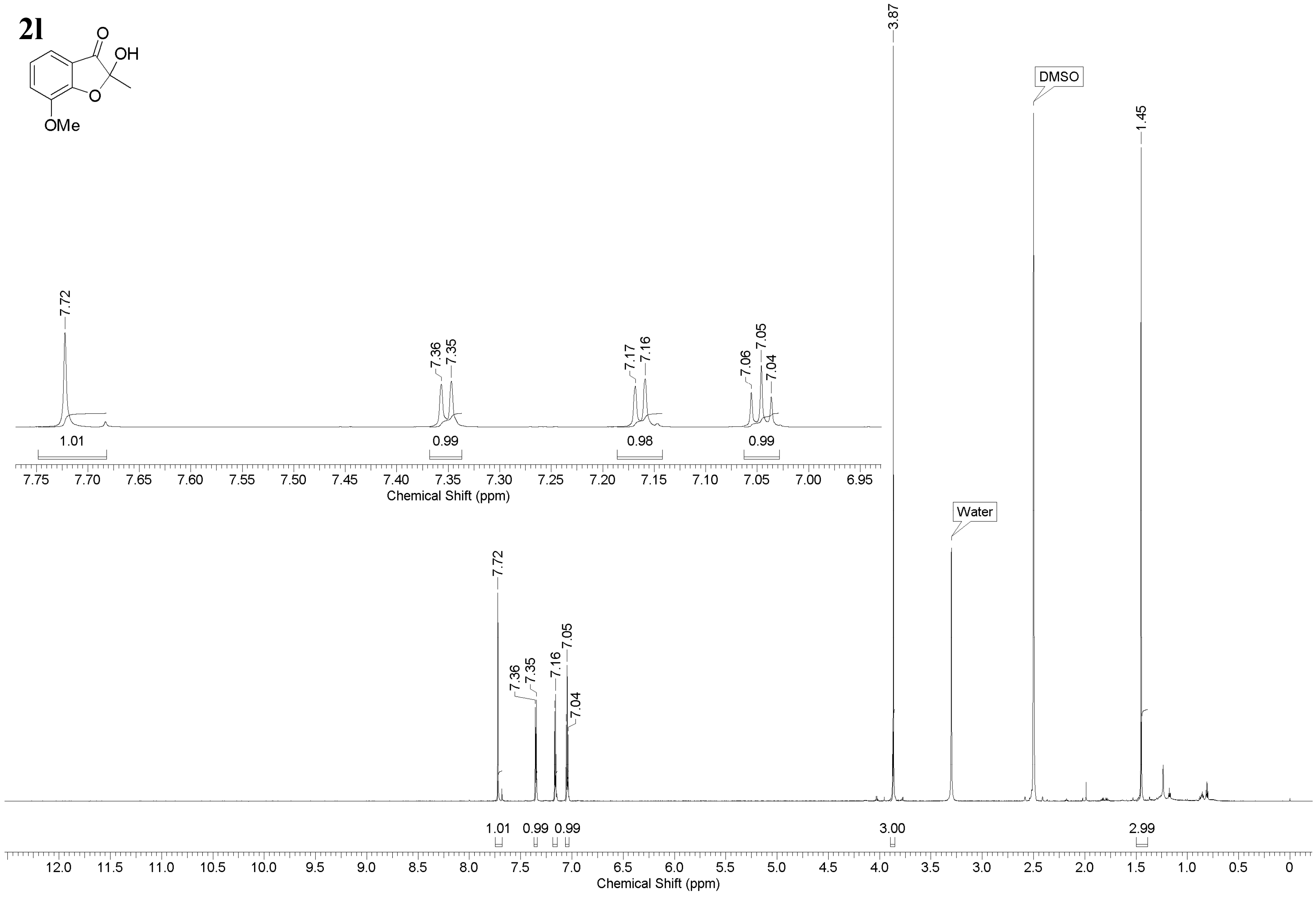

O
OH
O
OMe
7.72
7.36
7.35
7.17
7.16
7.06
7.05
7.04
1.01
0.99
0.98
0.99
Chemical Shift (ppm)
3.87
DMSO
1.45
Water
7.72
7.36
7.35
7.16
7.05
7.04
1.01
0.99
0.99
0.99
3.00
2.99
Chemical Shift (ppm)

2l

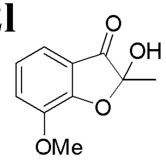
O
OH
O
OMe

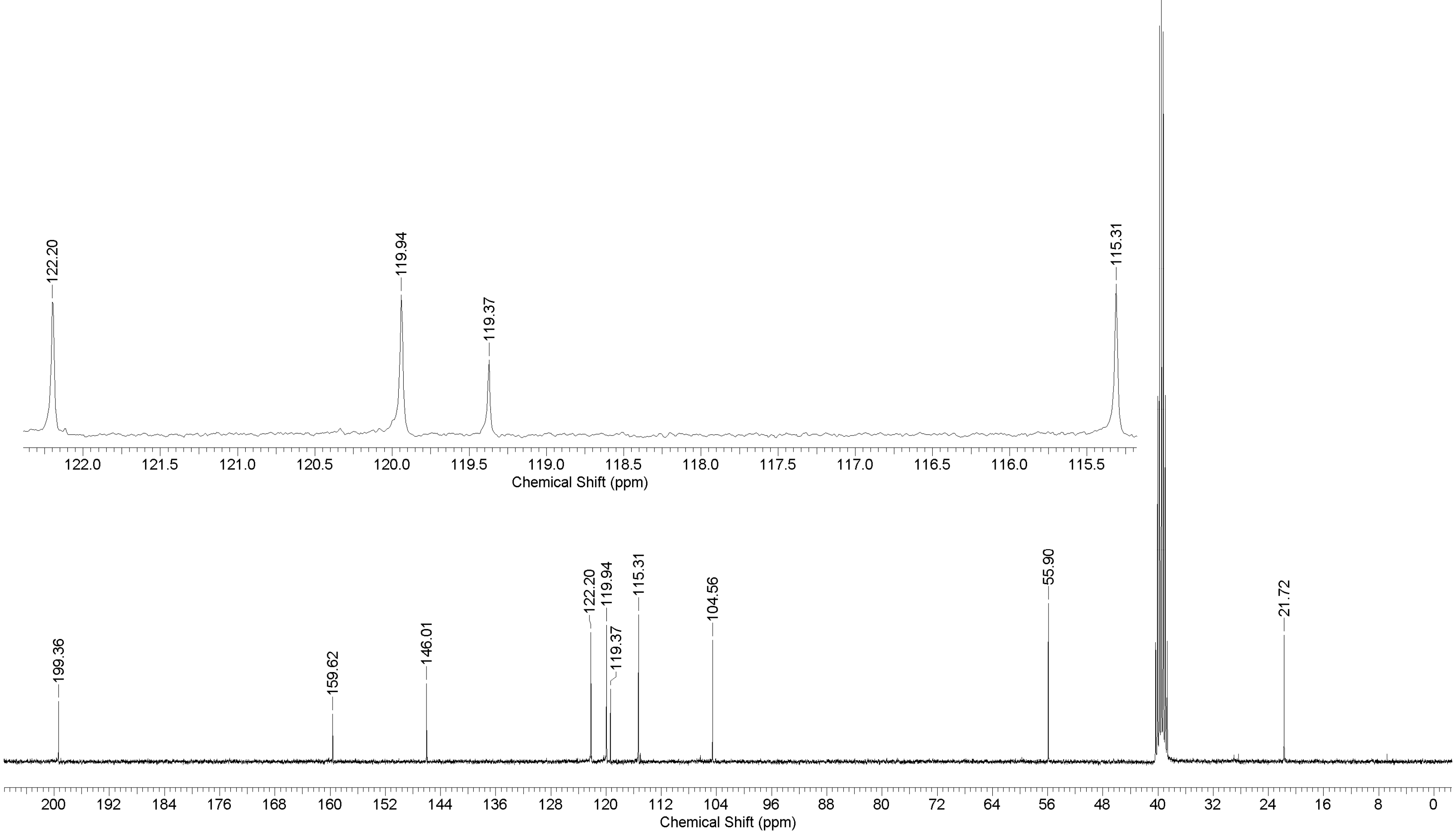
DMSO
122.20
119.94
119.37
115.31
122.0
121.5
121.0
120.5
120.0
119.5
119.0
118.5
118.0
117.5
117.0
116.5
116.0
115.5
Chemical Shift (ppm)
199.36
159.62
146.01
122.20
119.94
119.37
115.31
104.56
55.90
21.72
200
192
184
176
168
160
152
144
136
128
120
112
104
96
88
80
72
64
56
48
40
32
24
16
8
0
Chemical Shift (ppm)

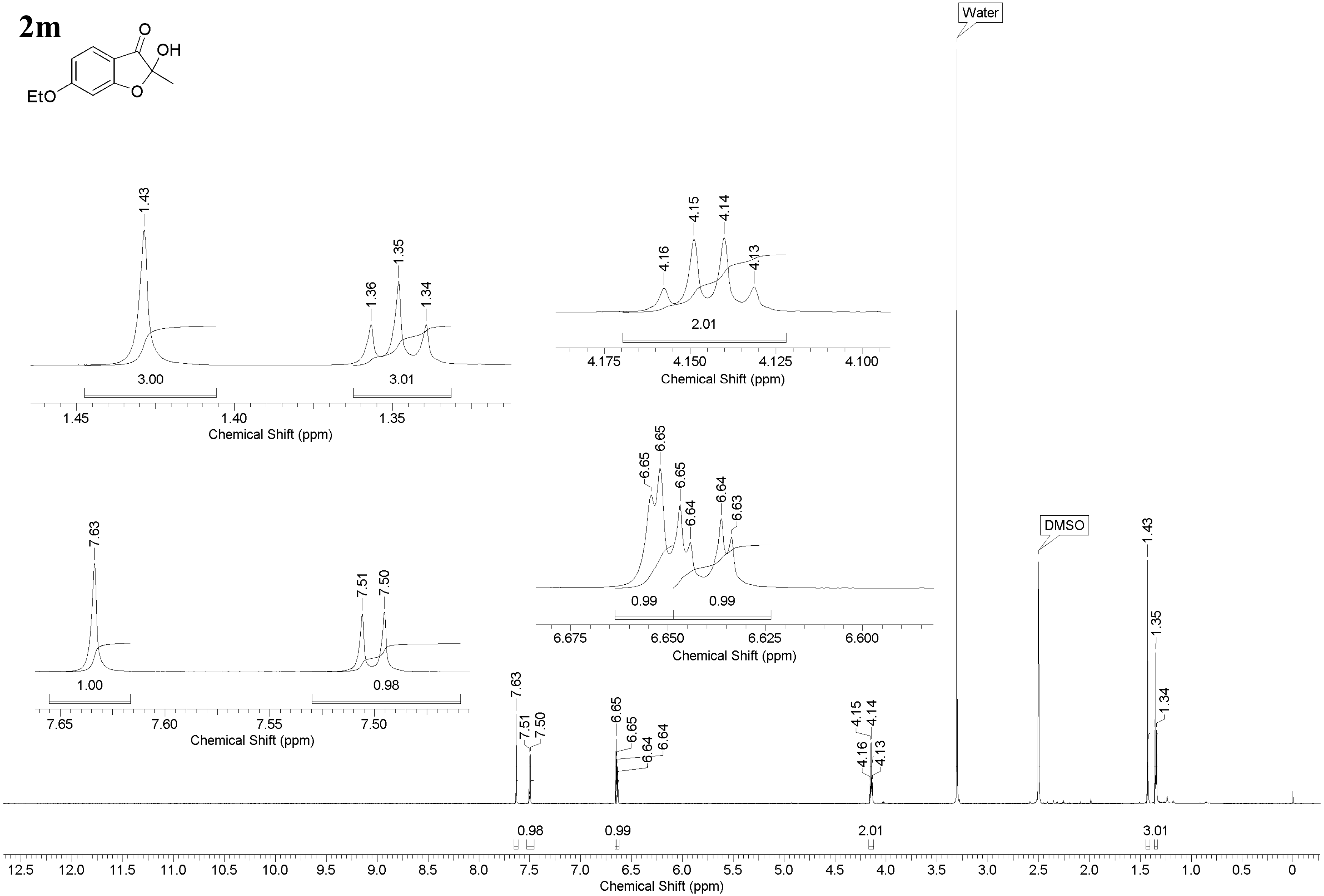
2m
O
OH
EtO
O
Water
DMSO
Chemical Shift (ppm)

2m

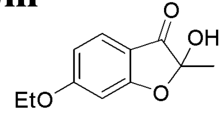

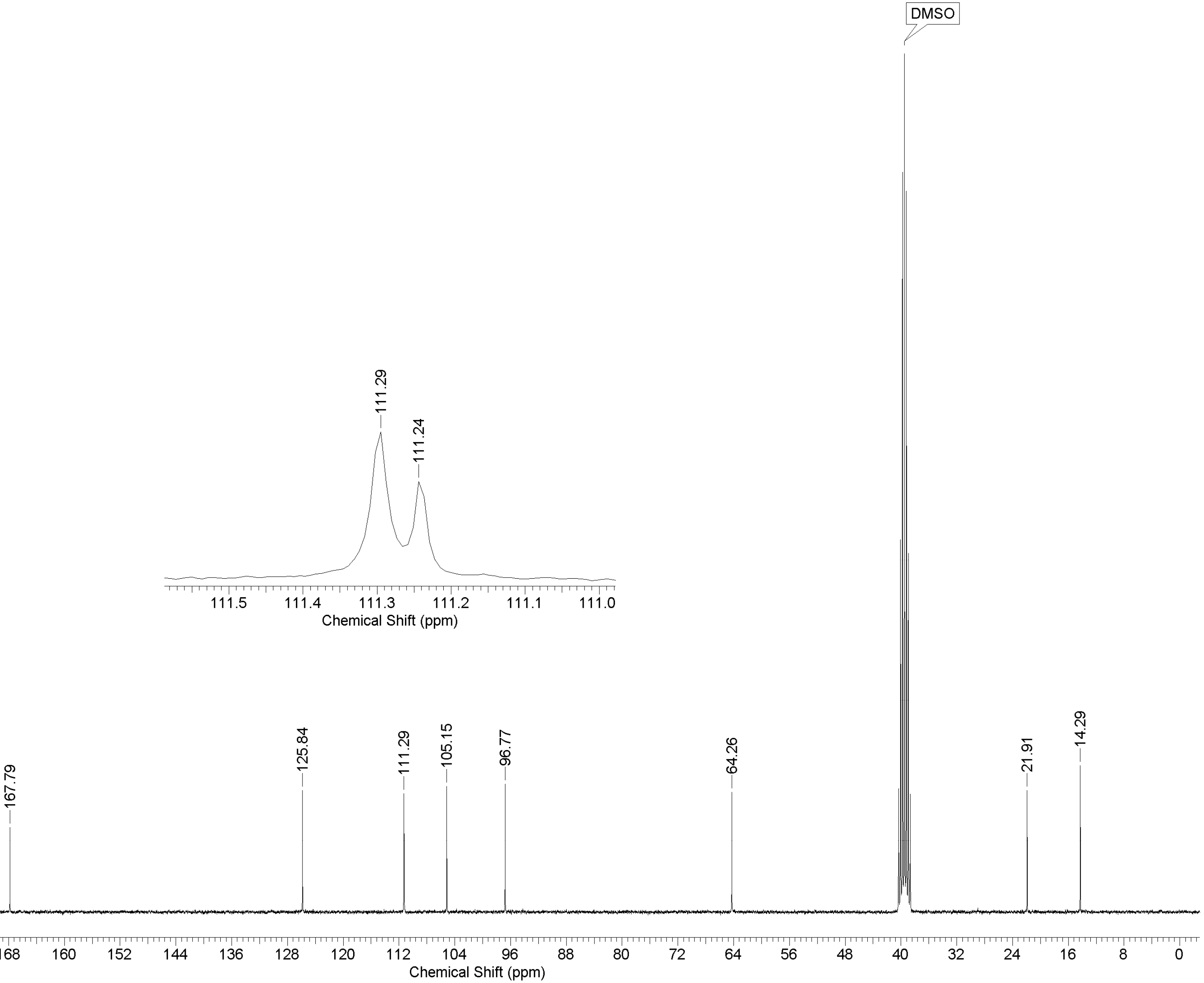
DMSO
196.48
171.88
167.79
125.84
111.29
105.15
96.77
64.26
21.91
14.29
111.29
111.24
111.5
111.4
111.3
111.2
111.1
111.0
Chemical Shift (ppm)
200
192
184
176
168
160
152
144
136
128
120
112
104
96
88
80
72
64
56
48
40
32
24
16
8
0
Chemical Shift (ppm)

2n

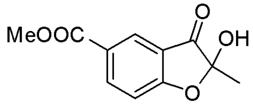

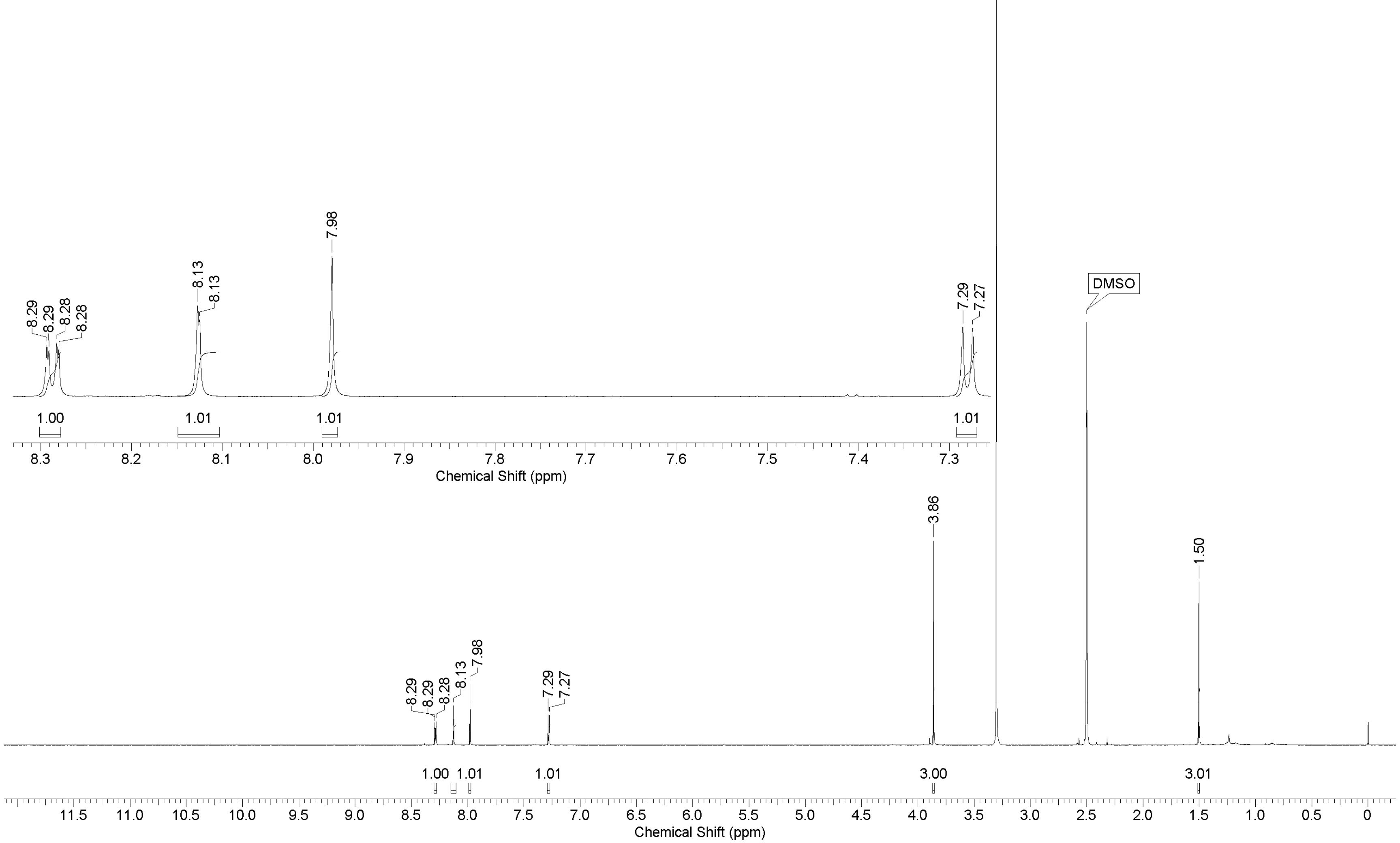
Water
DMSO
8.29
8.29
8.28
8.28
8.13
8.13
7.98
7.29
7.27
1.00
1.01
1.01
1.01
8.3
8.2
8.1
8.0
7.9
7.8
7.7
7.6
7.5
7.4
7.3
Chemical Shift (ppm)
3.86
1.50
3.00
3.01
11.5
11.0
10.5
10.0
9.5
9.0
8.5
8.0
7.5
7.0
6.5
6.0
5.5
5.0
4.5
4.0
3.5
3.0
2.5
2.0
1.5
1.0
0.5
0
Chemical Shift (ppm)

2n

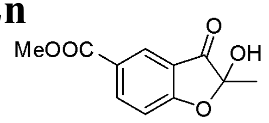
MeOOC
O
OH
O

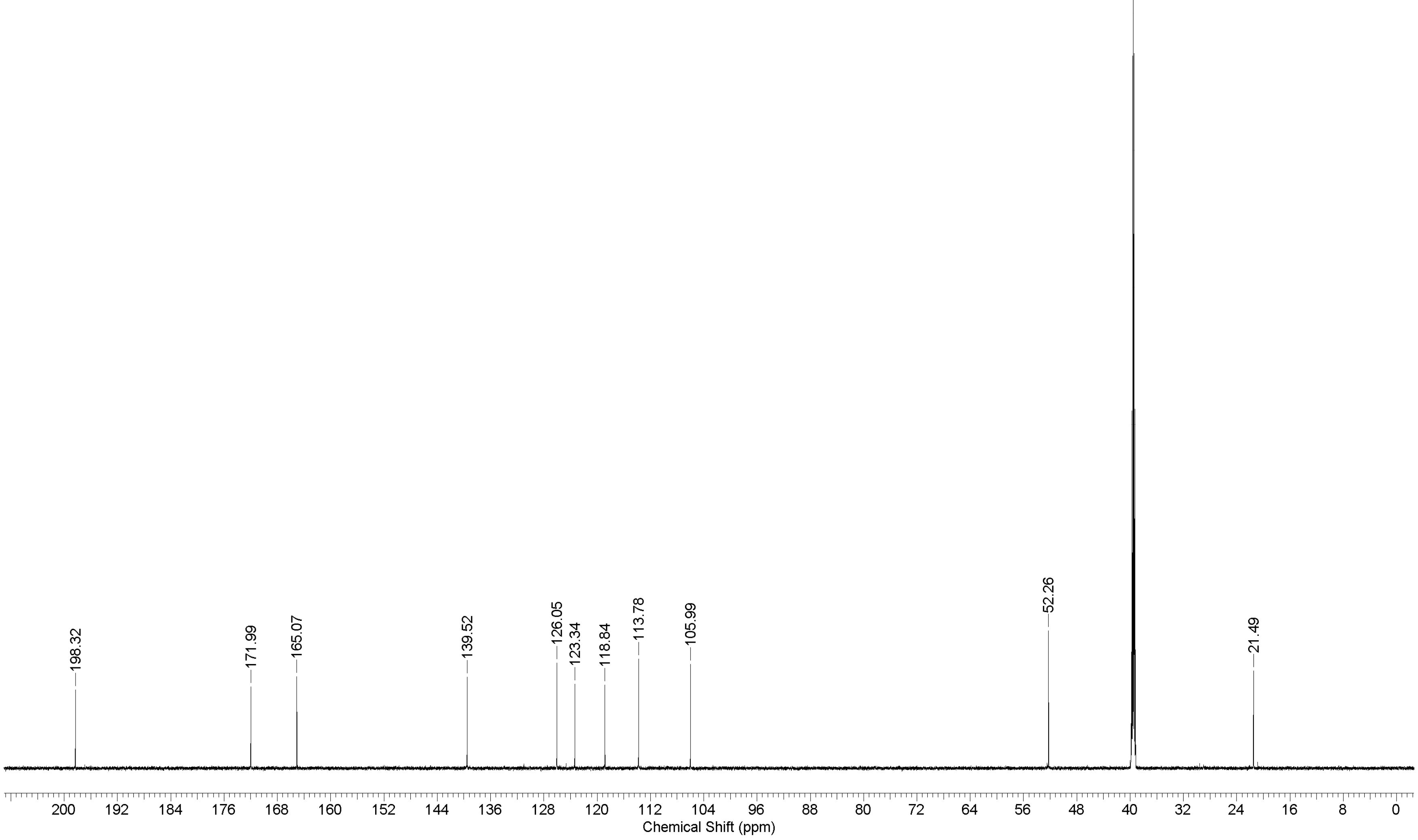
DMSO
198.32
171.99
165.07
139.52
126.05
123.34
118.84
113.78
105.99
52.26
21.49
200 192 184 176 168 160 152 144 136 128 120 112 104 96 88 80 72 64 56 48 40 32 24 16 8 0
Chemical Shift (ppm)

3a

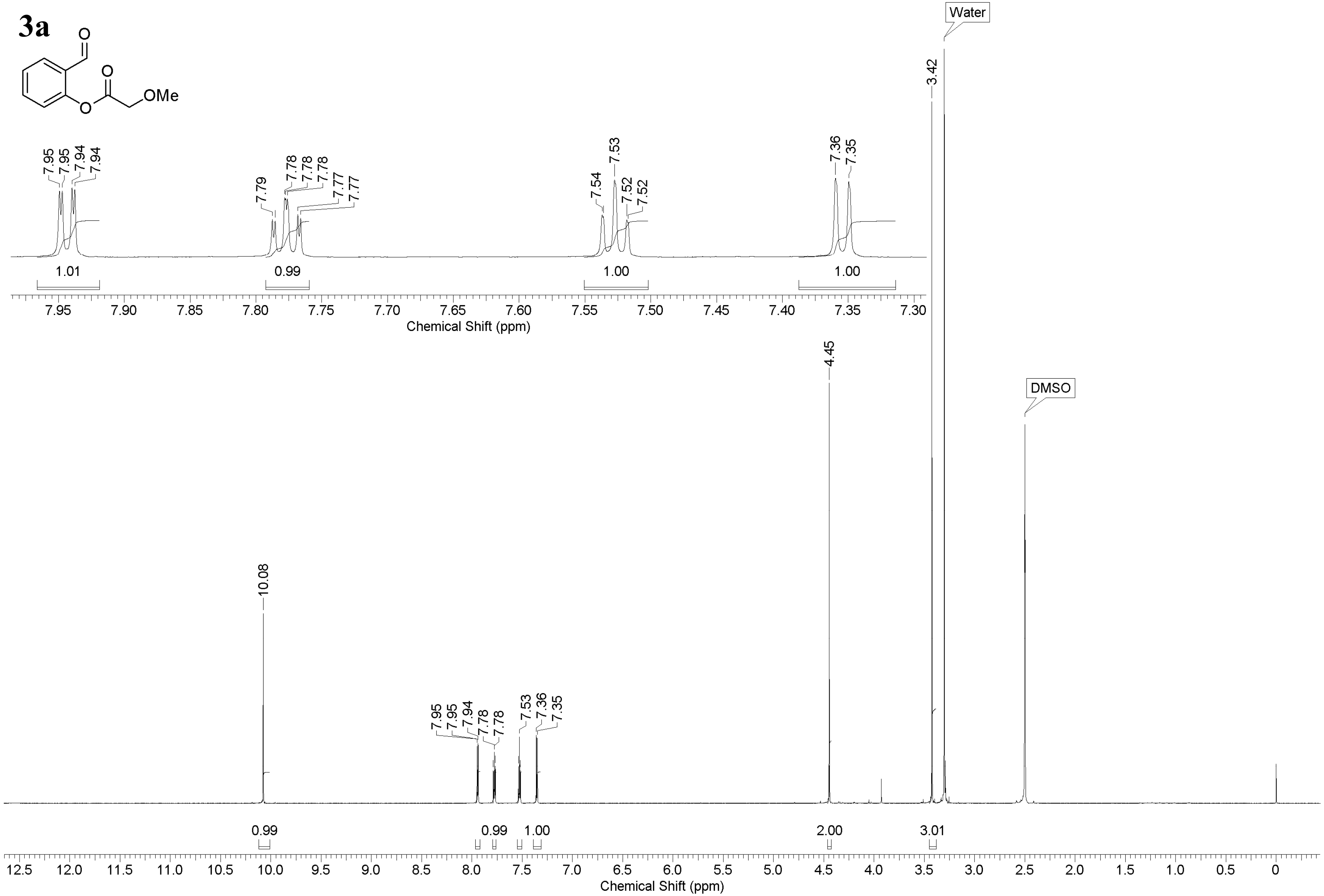

3a

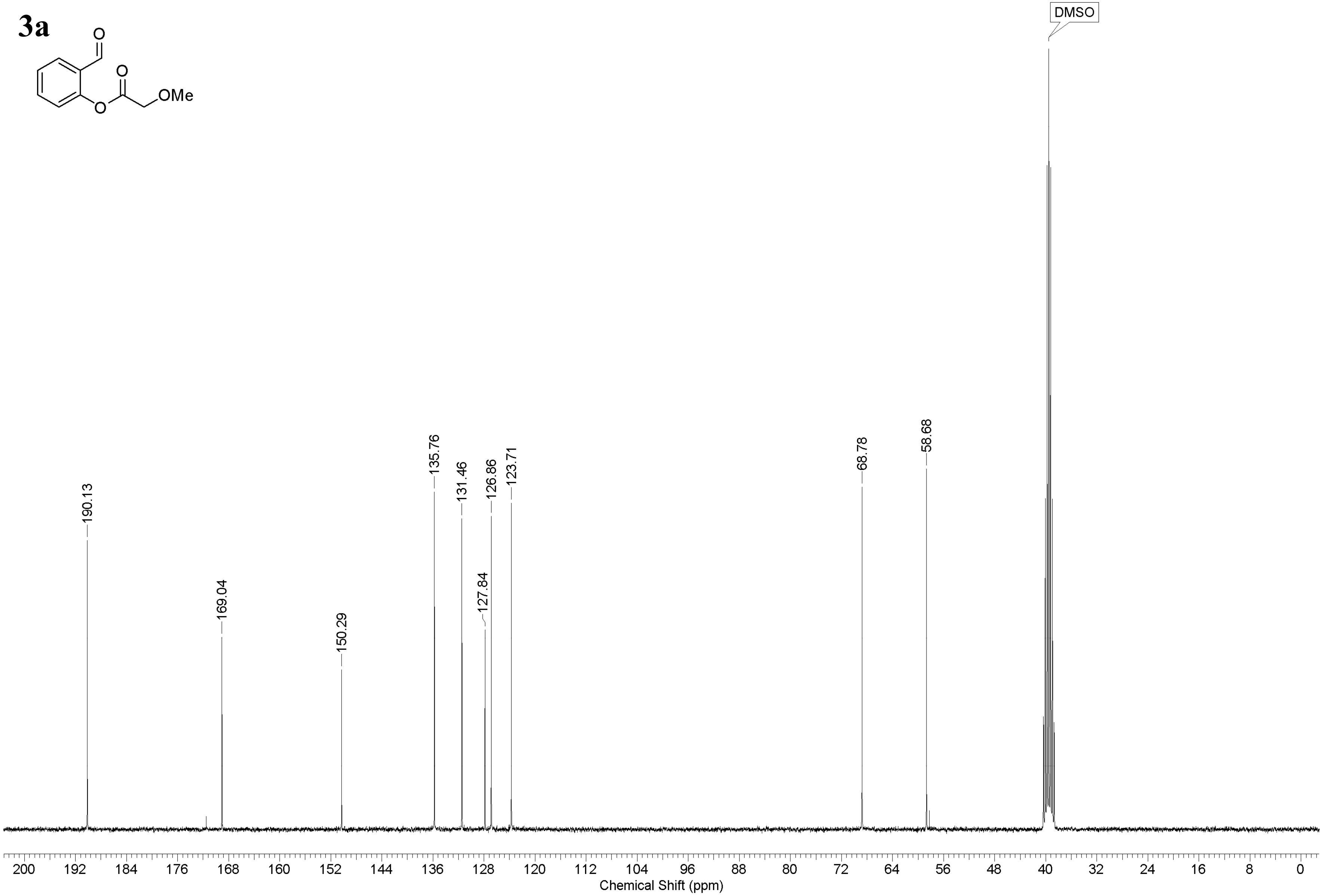
OMe
DMSO
190.13
169.04
150.29
135.76
131.46
127.84
126.86
123.71
68.78
58.68
200 192 184 176 168 160 152 144 136 128 120 112 104 96 88 80 72 64 56 48 40 32 24 16 8 0
Chemical Shift (ppm)

3b

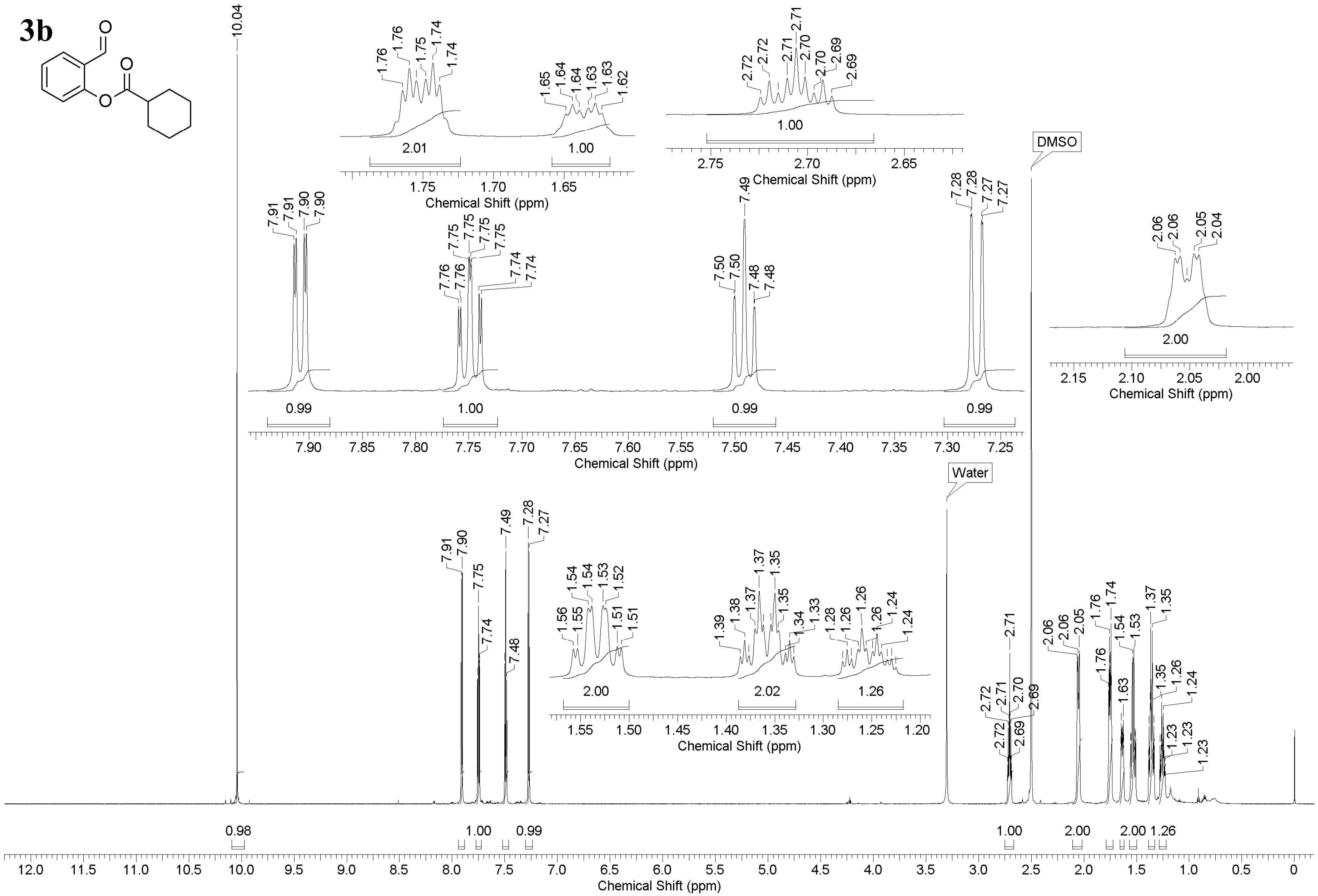

3c

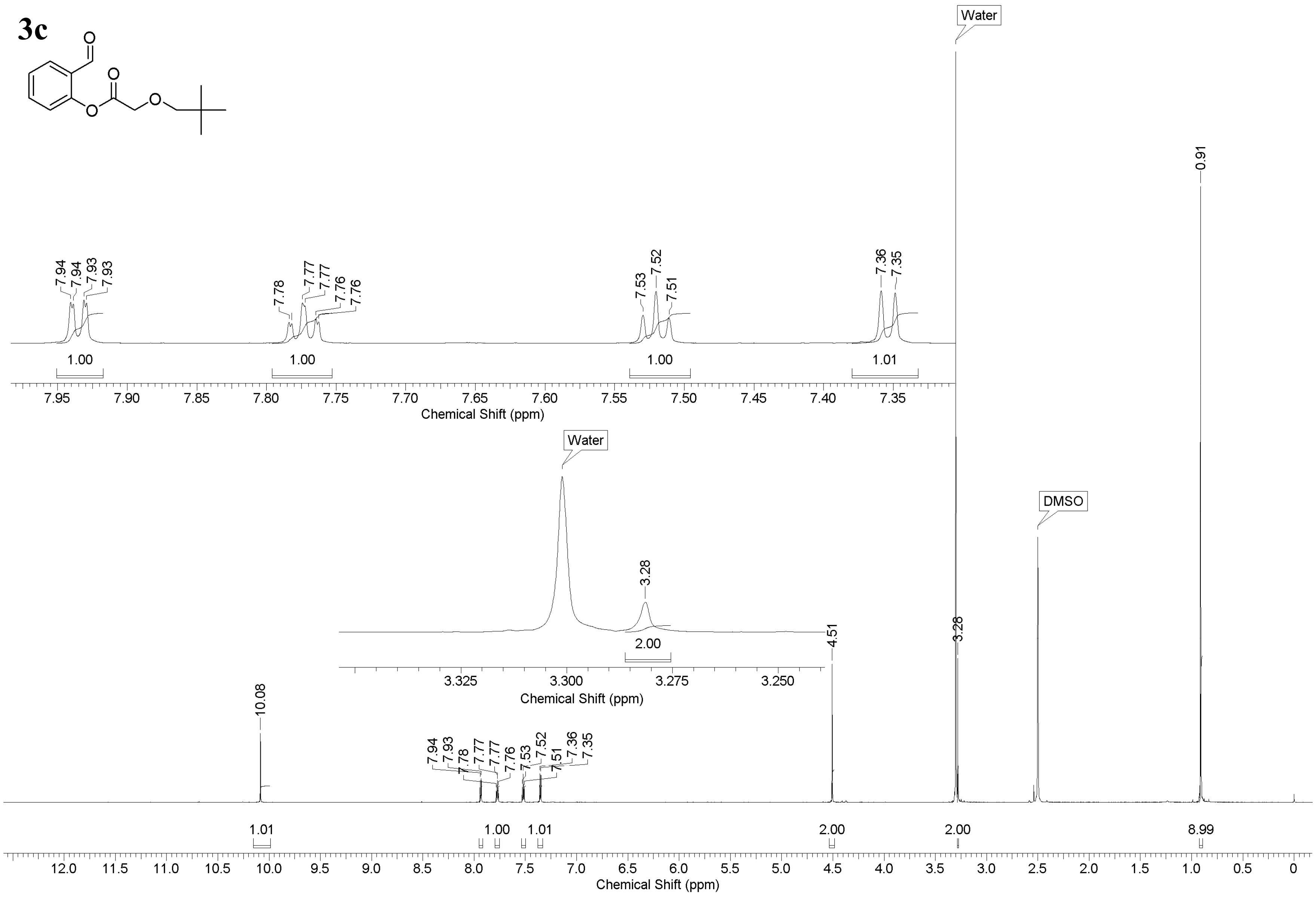

Water
0.91
7.94
7.94
7.93
7.93
7.78
7.77
7.77
7.76
7.76
7.53
7.52
7.51
7.36
7.35
1.00
1.00
1.00
1.01
Chemical Shift (ppm)
Water
DMSO
3.28
2.00
4.51
3.28
10.08
1.01
1.00
1.01
2.00
2.00
8.99
Chemical Shift (ppm)

3c

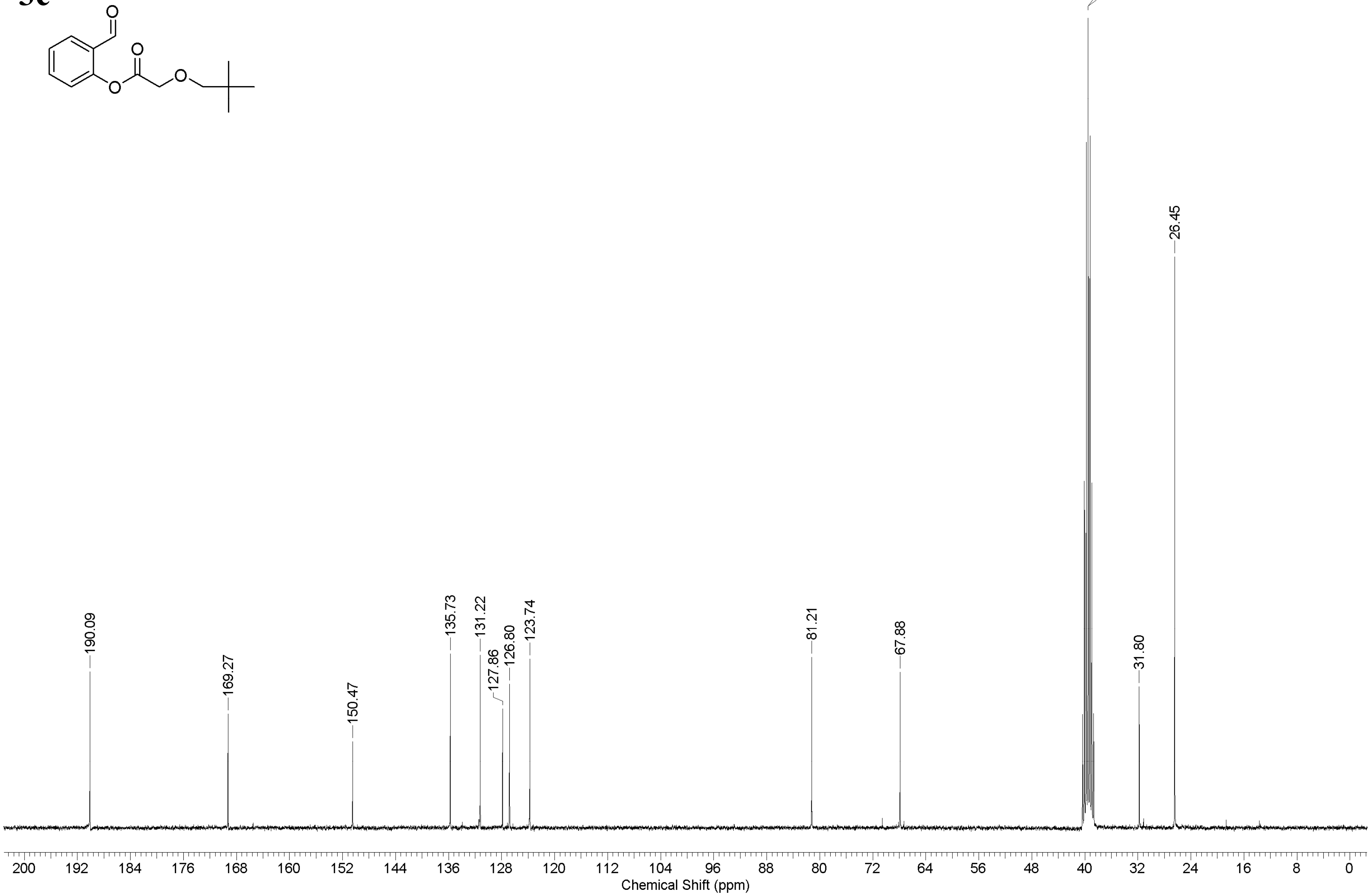
DMSO
190.09
169.27
150.47
135.73
131.22
127.86
126.80
123.74
81.21
67.88
31.80
26.45
200 192 184 176 168 160 152 144 136 128 120 112 104 96 88 80 72 64 56 48 40 32 24 16 8 0
Chemical Shift (ppm)

3d

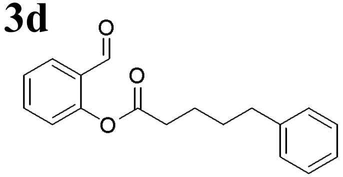

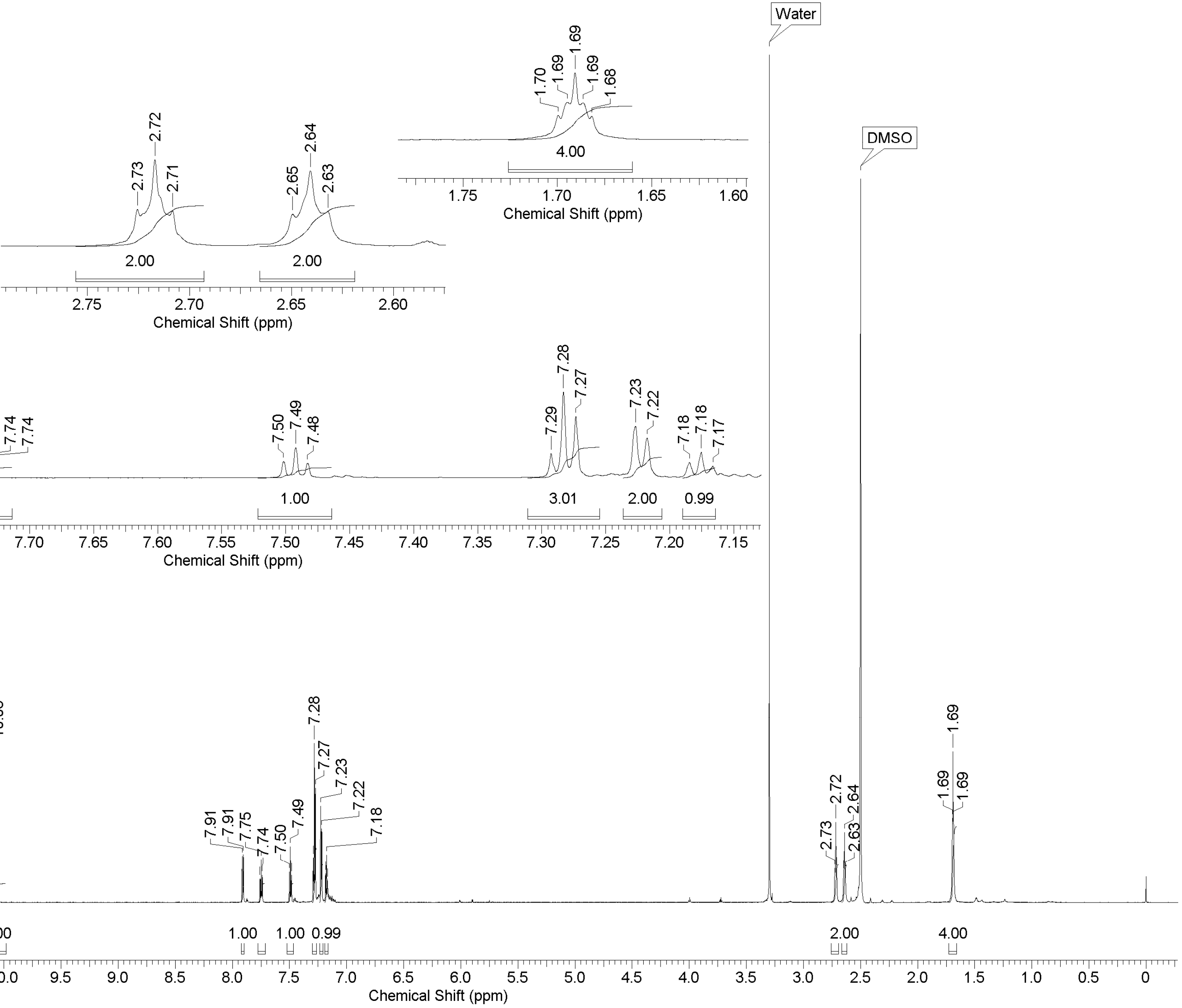
Water
DMSO
10.05
7.91 7.91 7.75 7.74 7.50 7.49 7.28 7.27 7.23 7.22 7.18
2.73 2.72 2.64 2.63
1.69 1.69 1.69
1.00
1.00 1.00 0.99
2.00
4.00
Chemical Shift (ppm)
7.91 7.91 7.91 7.90
7.76 7.76 7.75 7.75 7.74 7.74
7.50 7.49 7.48
7.29 7.28 7.27
7.23 7.22
7.18 7.18 7.17
1.00 1.00 1.00 3.01 2.00 0.99
2.73 2.72 2.71
2.65 2.64 2.63
2.00 2.00
1.70 1.69 1.69 1.69 1.68
4.00

3d

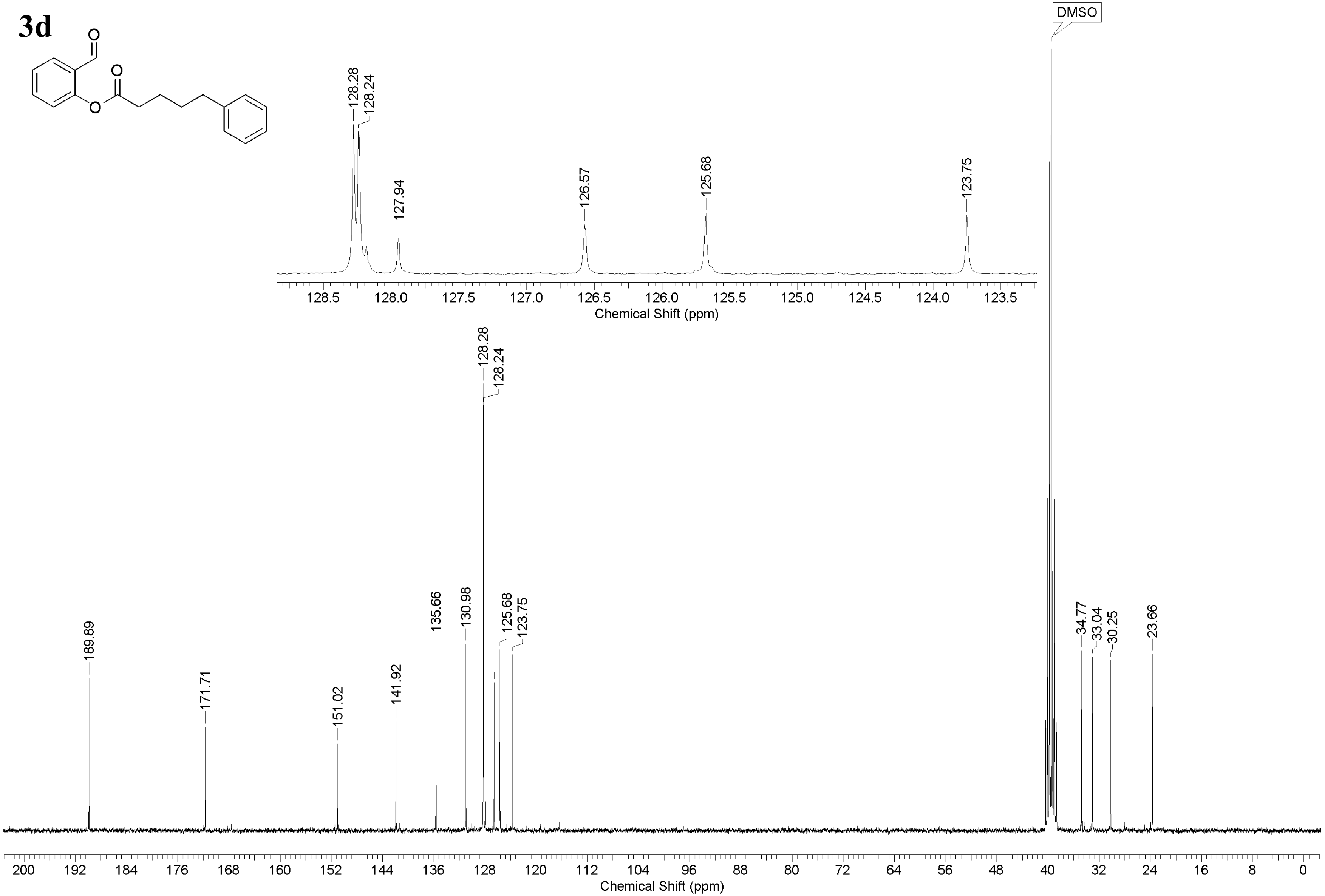

DMSO
128.28
128.24
127.94
126.57
125.68
123.75
Chemical Shift (ppm)
189.89
171.71
151.02
141.92
135.66
130.98
34.77
33.04
30.25
23.66

3h

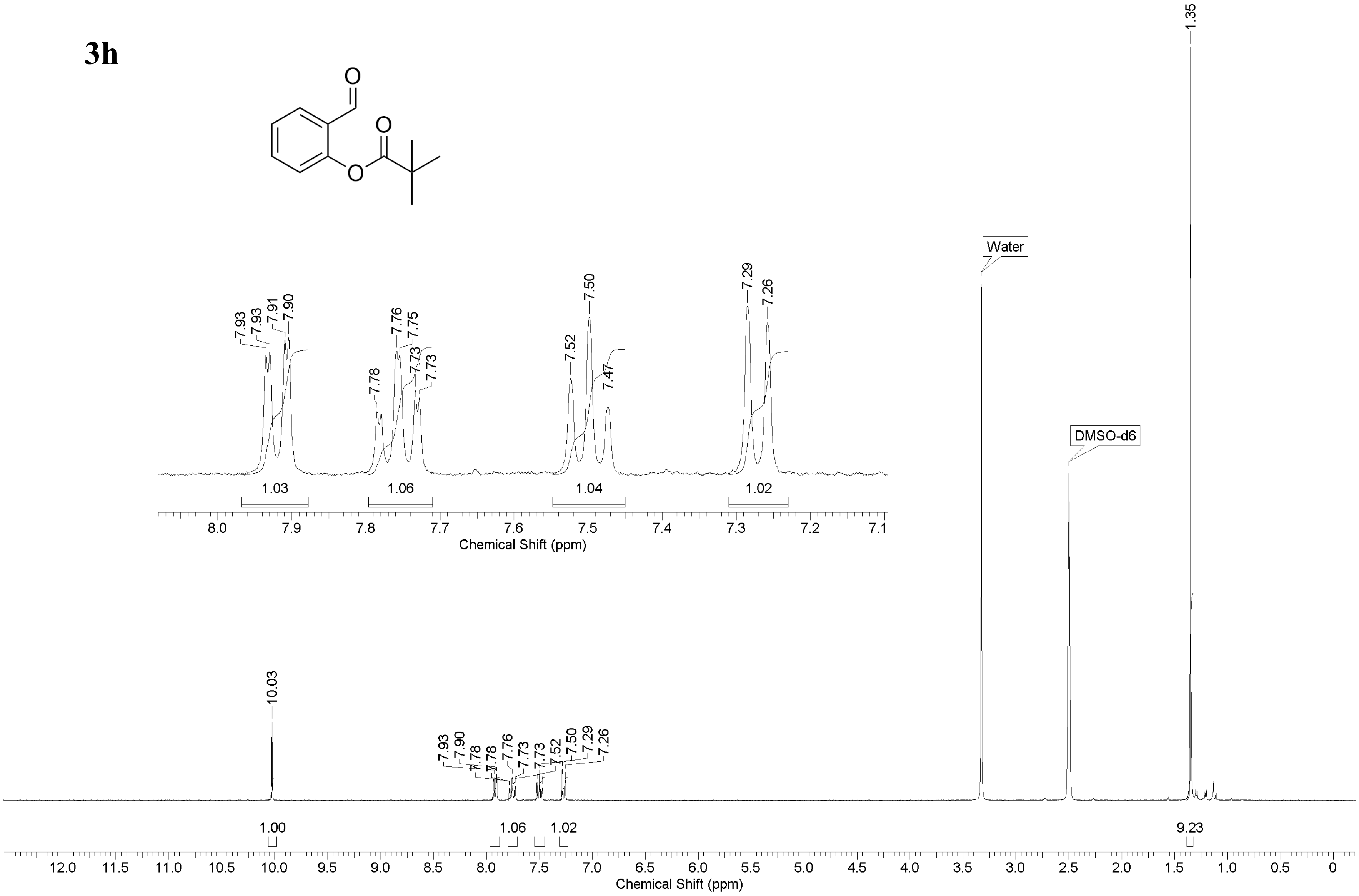

3h

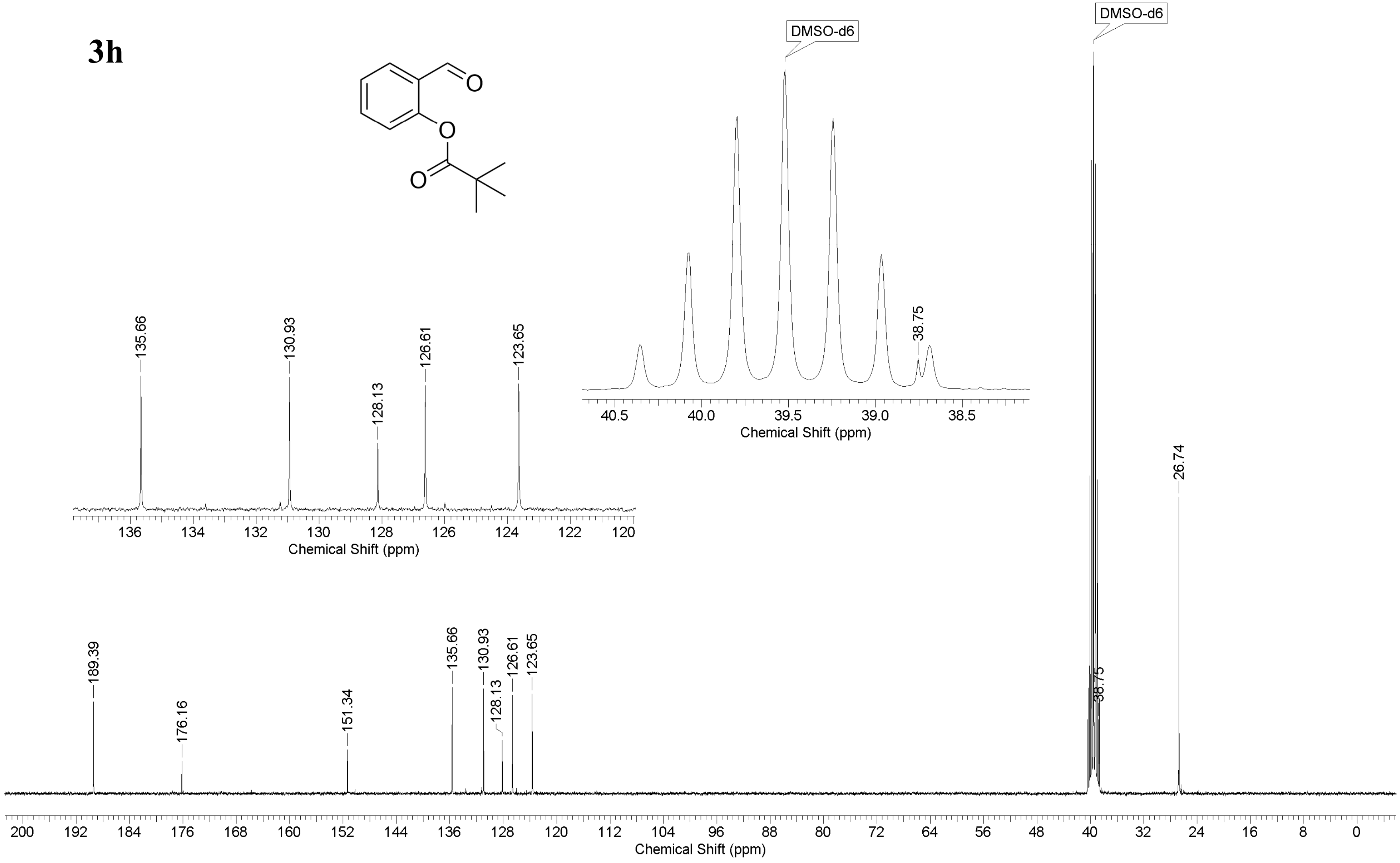

O
O
O
DMSO-d6
DMSO-d6
135.66
130.93
128.13
126.61
123.65
136 134 132 130 128 126 124 122 120
Chemical Shift (ppm)
38.75
40.5 40.0 39.5 39.0 38.5
Chemical Shift (ppm)
189.39
176.16
151.34
135.66
130.93
128.13
126.61
123.65
38.75
26.74
200 192 184 176 168 160 152 144 136 128 120 112 104 96 88 80 72 64 56 48 40 32 24 16 8 0
Chemical Shift (ppm)

3i

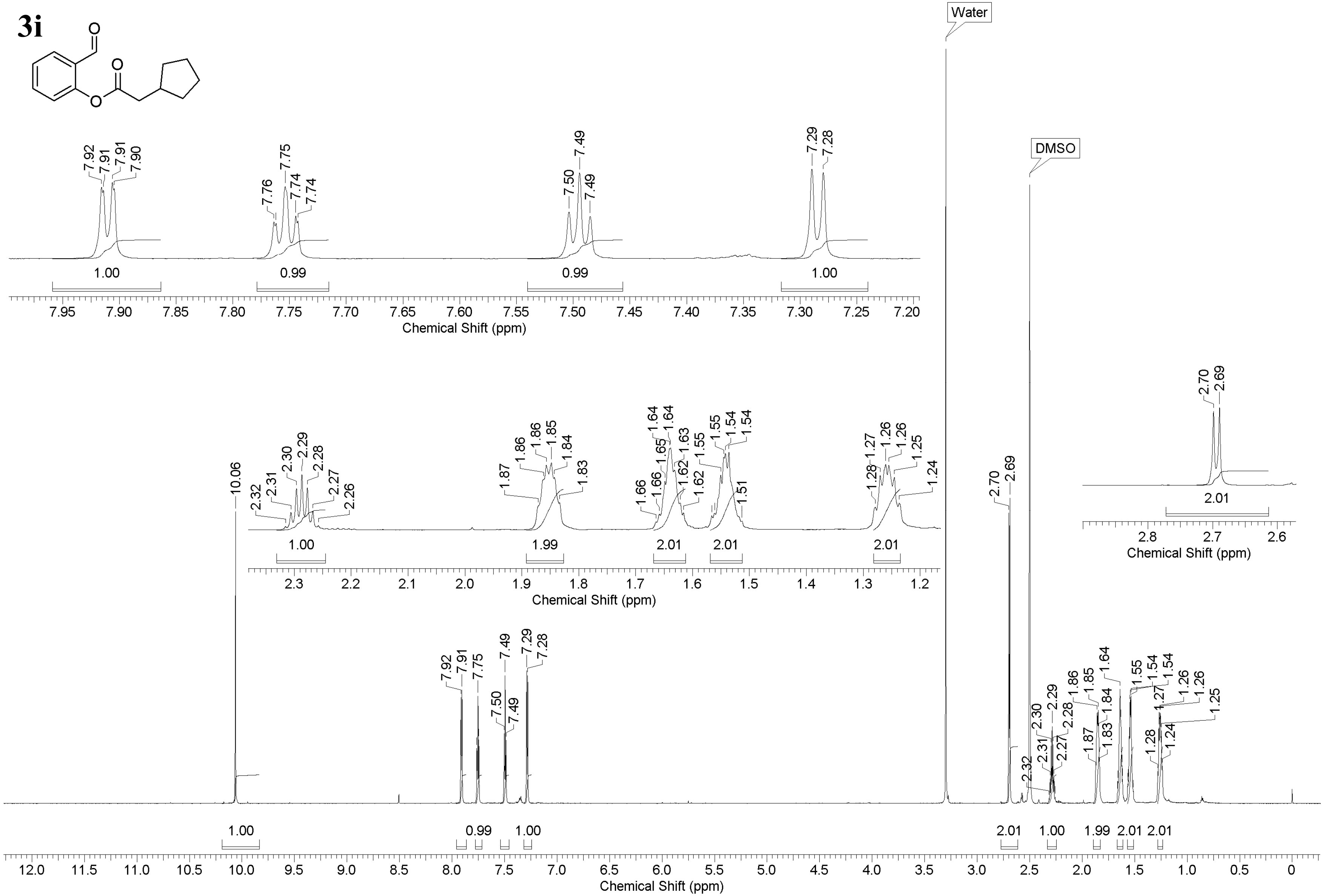
Water
DMSO
10.06
7.92
7.91
7.90
7.76
7.75
7.74
7.50
7.49
7.29
7.28
2.70
2.69
2.32
2.31
2.30
2.29
2.28
2.27
2.26
1.87
1.86
1.85
1.84
1.83
1.66
1.65
1.64
1.63
1.62
1.55
1.54
1.51
1.28
1.27
1.26
1.25
1.24
1.00
0.99
2.01
1.99
Chemical Shift (ppm)

3i

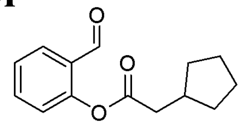

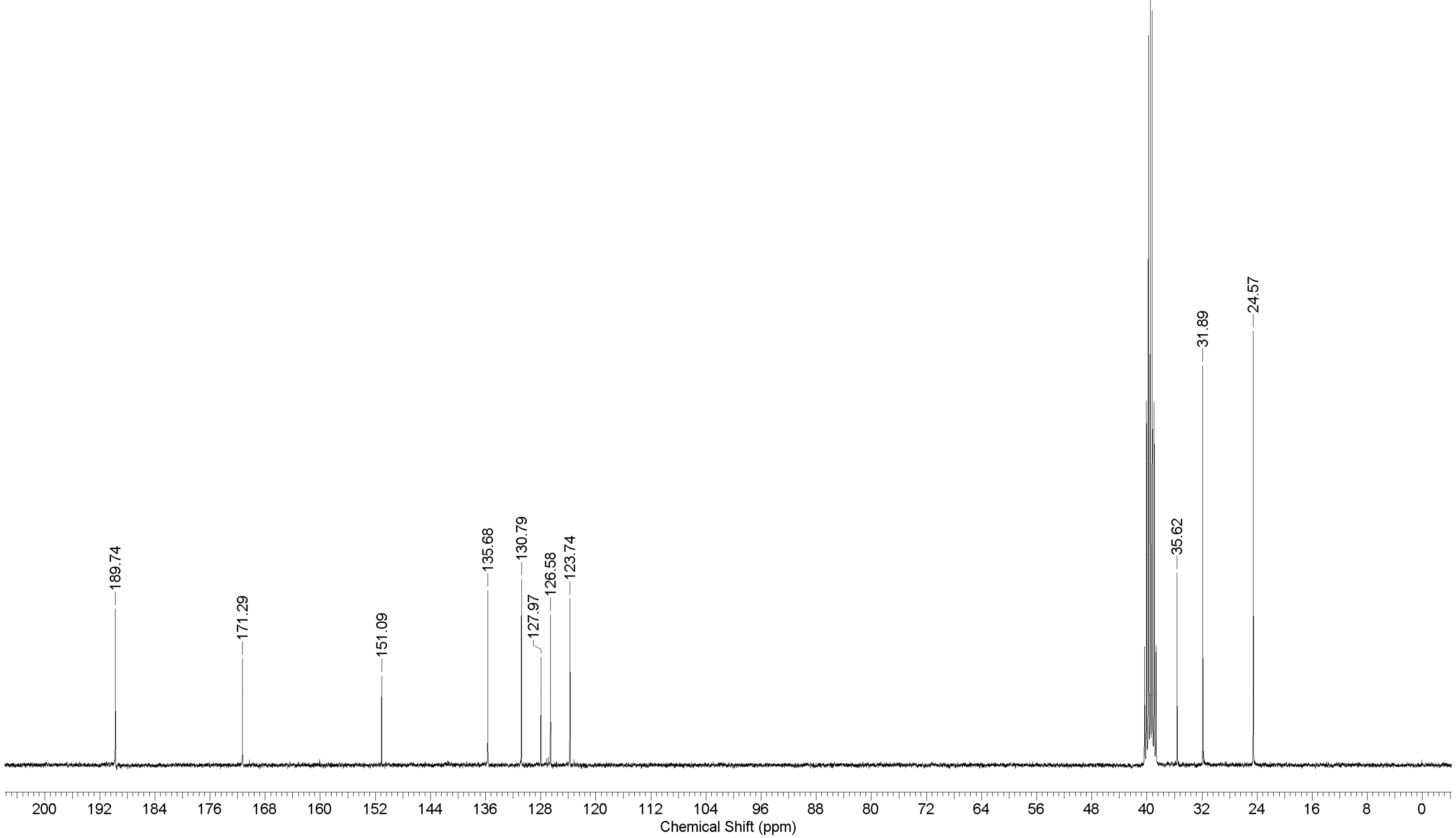
DMSO
189.74
171.29
151.09
135.68
130.79
127.97
126.58
123.74
35.62
31.89
24.57
200
192
184
176
168
160
152
144
136
128
120
112
104
96
88
80
72
64
56
48
40
32
24
16
8
0
Chemical Shift (ppm)

3j

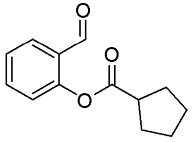

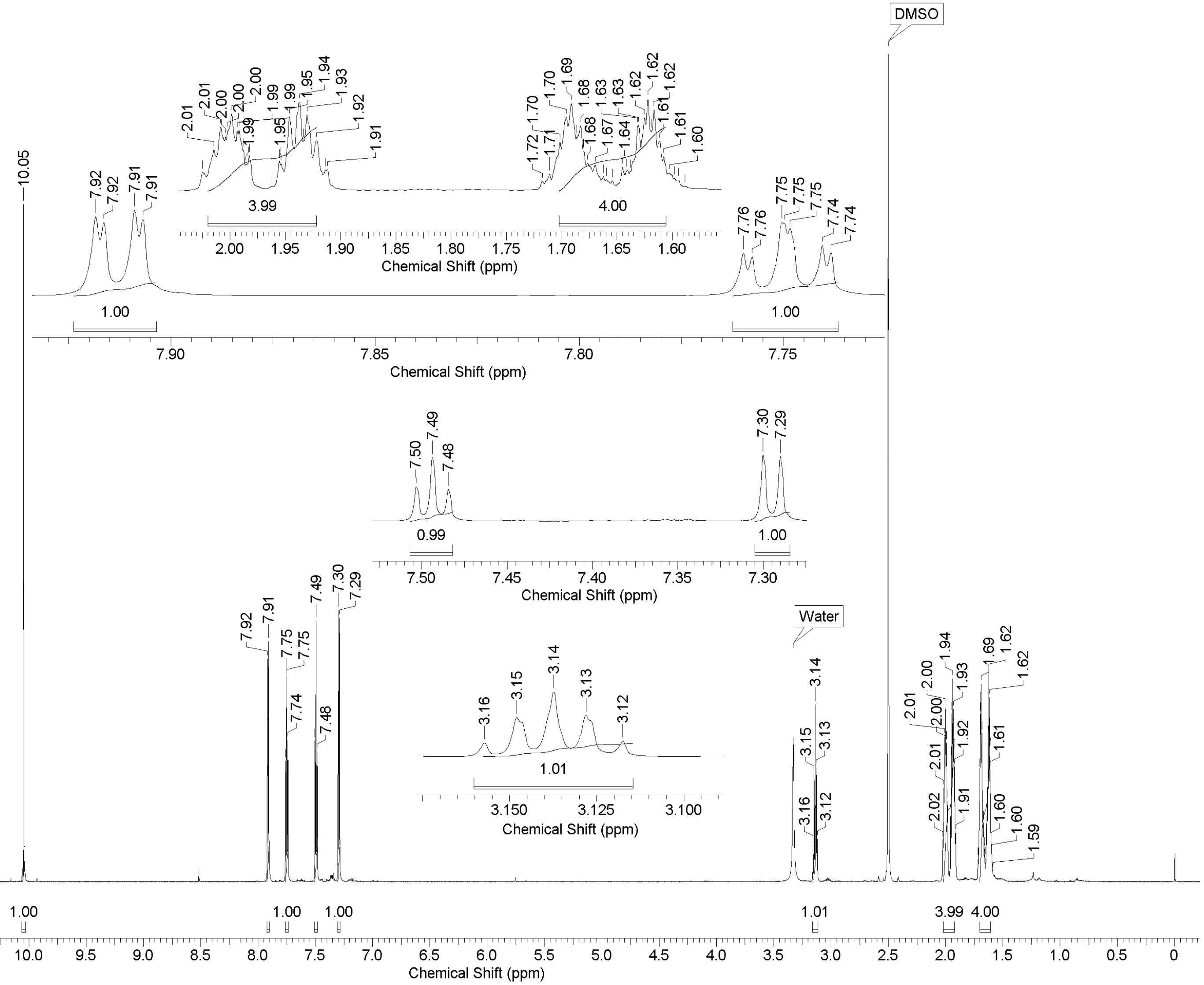

3k

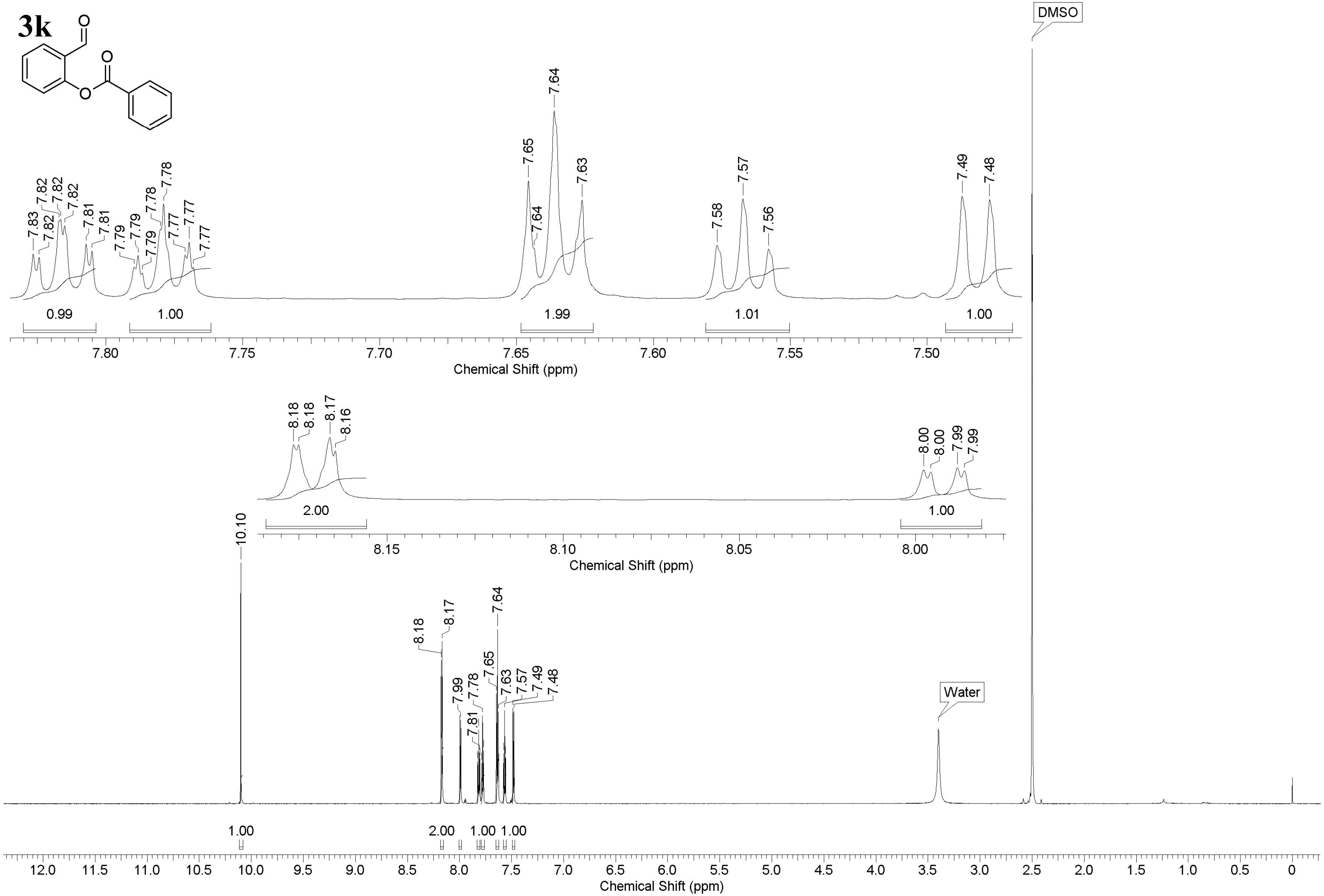
DMSO
Water
Chemical Shift (ppm)
Chemical Shift (ppm)
Chemical Shift (ppm)

3l

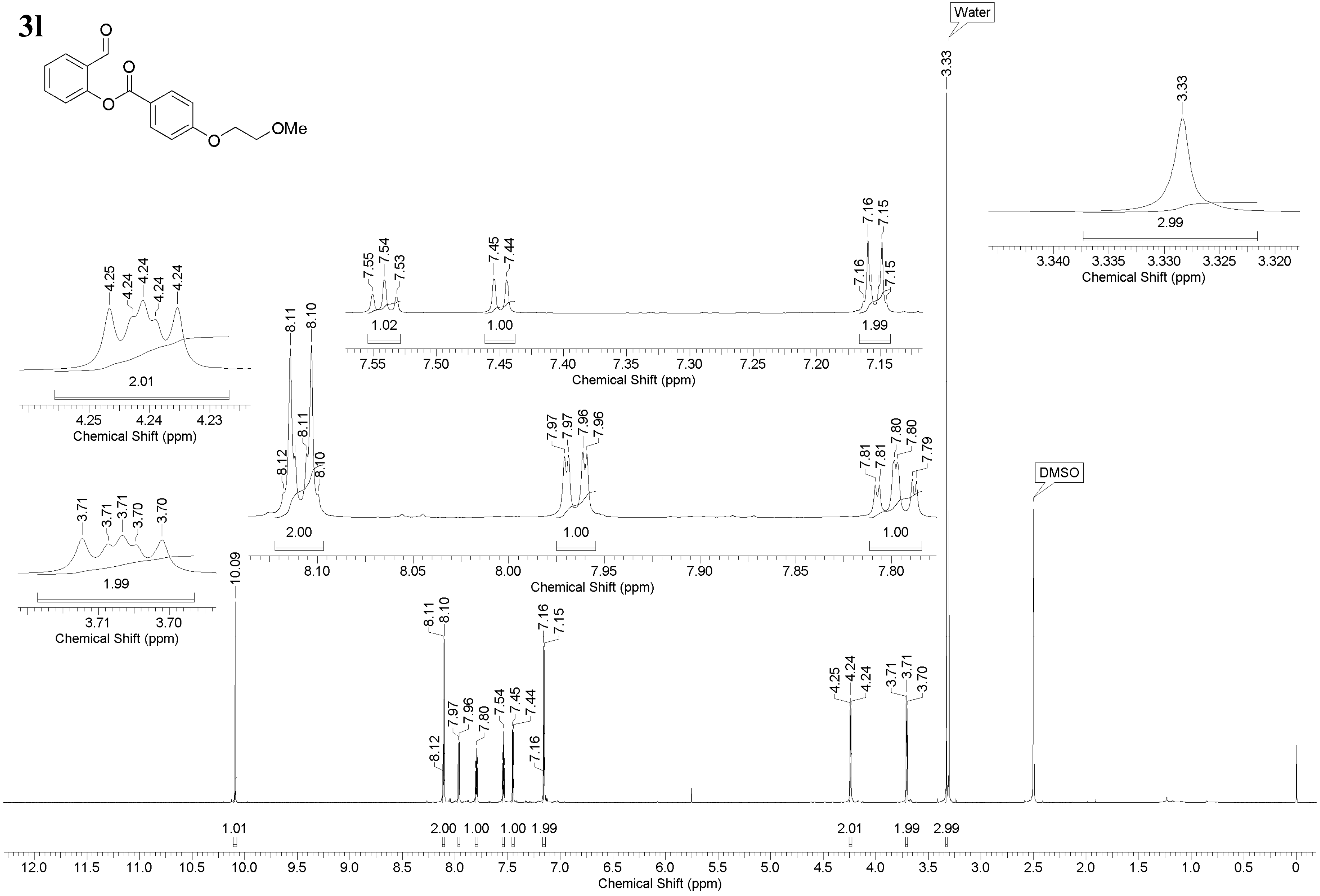

3l

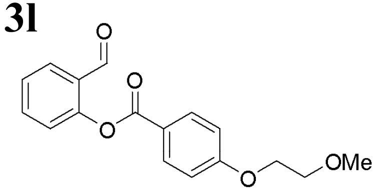
OMe

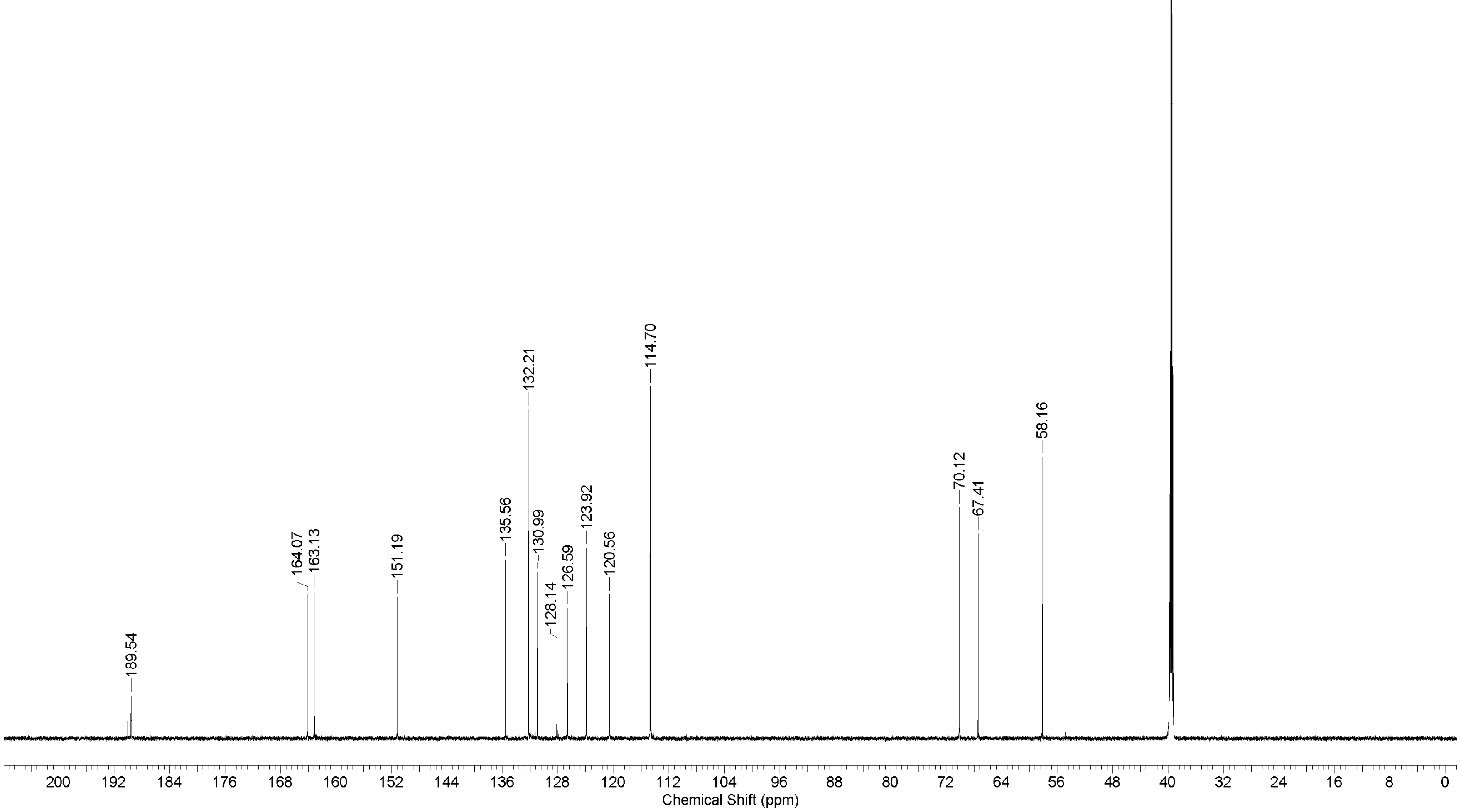
DMSO
189.54
164.07
163.13
151.19
135.56
132.21
130.99
128.14
126.59
123.92
120.56
114.70
70.12
67.41
58.16
200
192
184
176
168
160
152
144
136
128
120
112
104
96
88
80
72
64
56
48
40
32
24
16
8
0
Chemical Shift (ppm)

3p

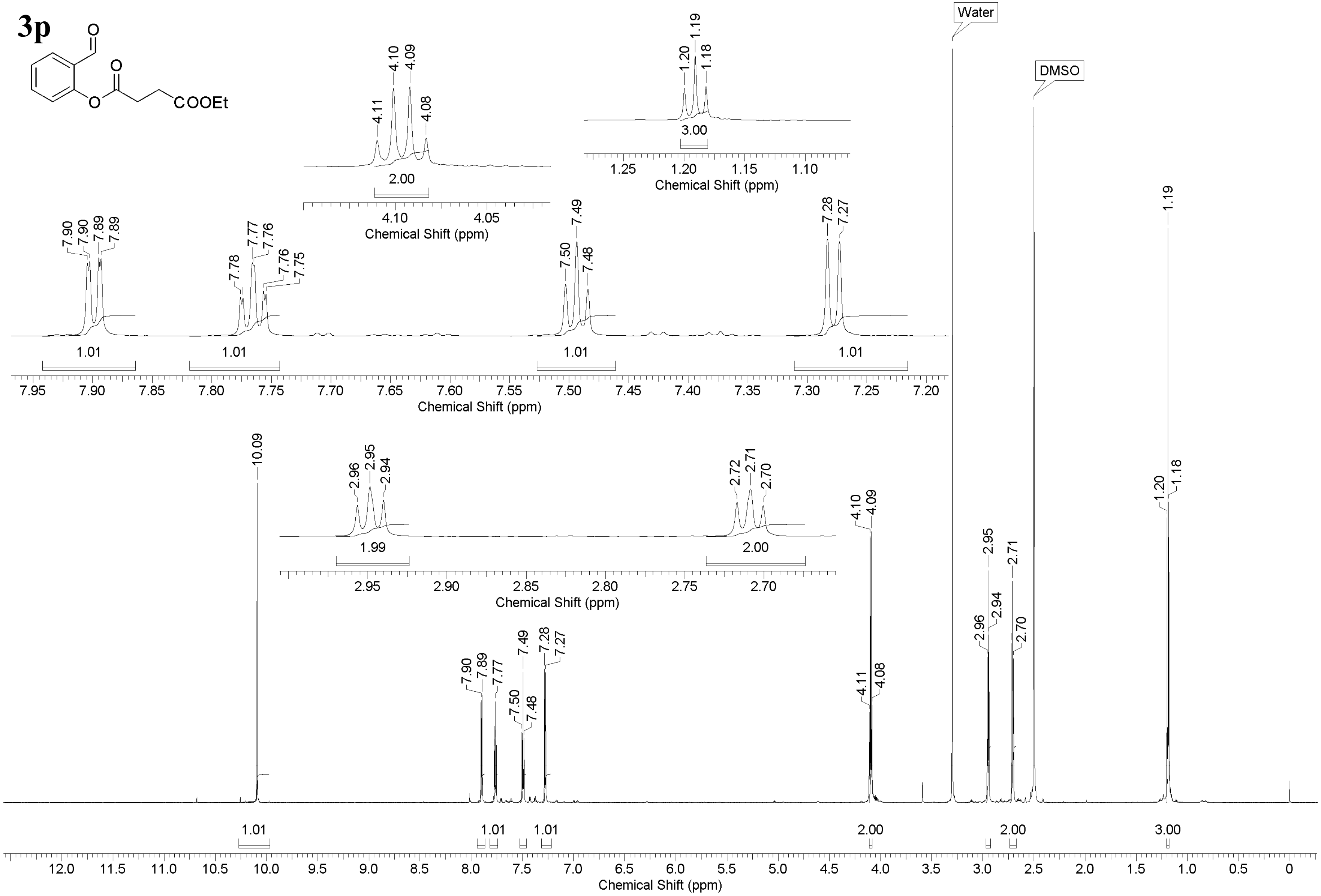

COOEt
Water
DMSO
10.09
7.90
7.89
7.77
7.50
7.49
7.48
7.28
7.27
4.11
4.10
4.09
4.08
2.96
2.95
2.94
2.71
2.70
1.20
1.19
1.18
1.01
2.00
3.00
1.99
Chemical Shift (ppm)

3e

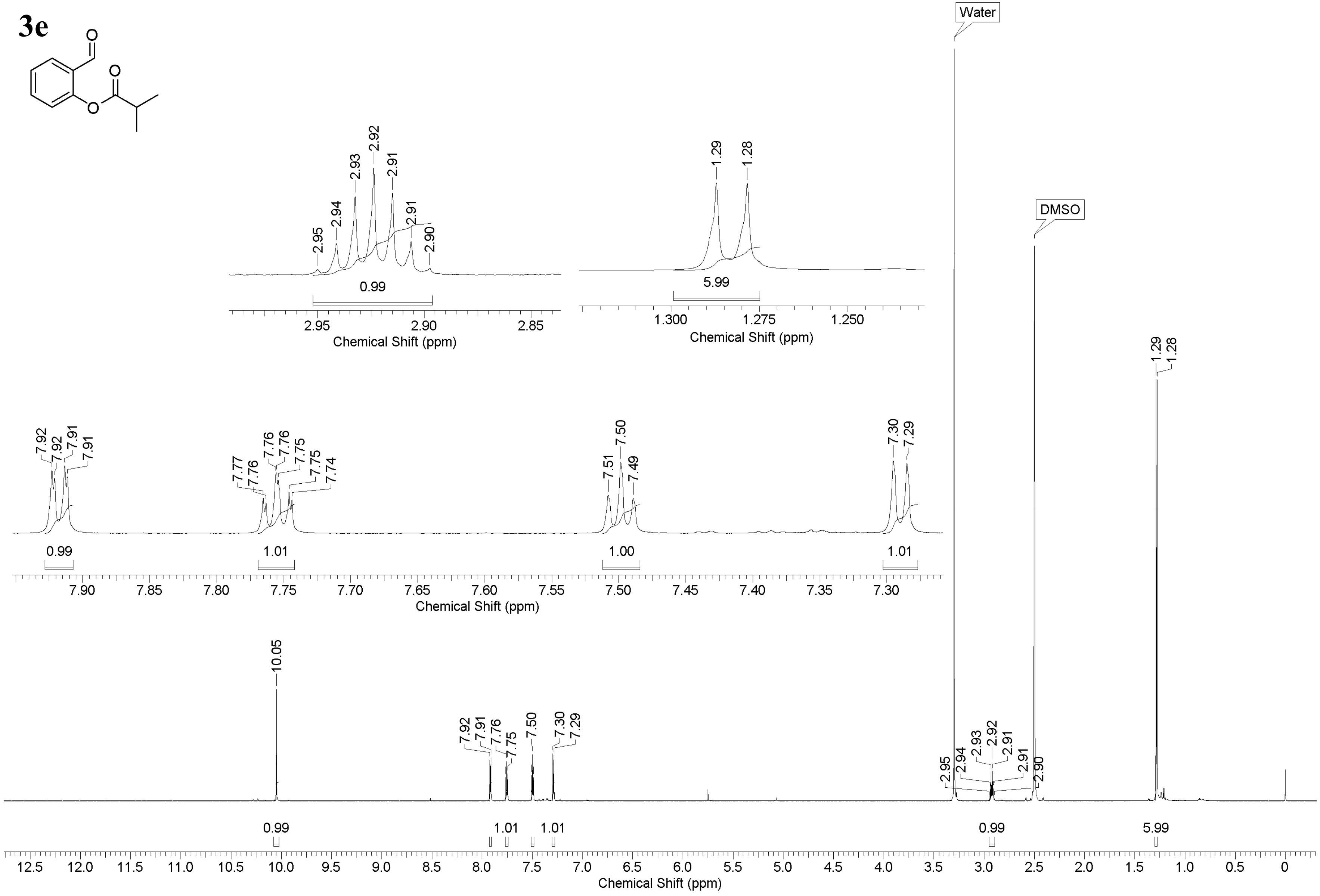

3f

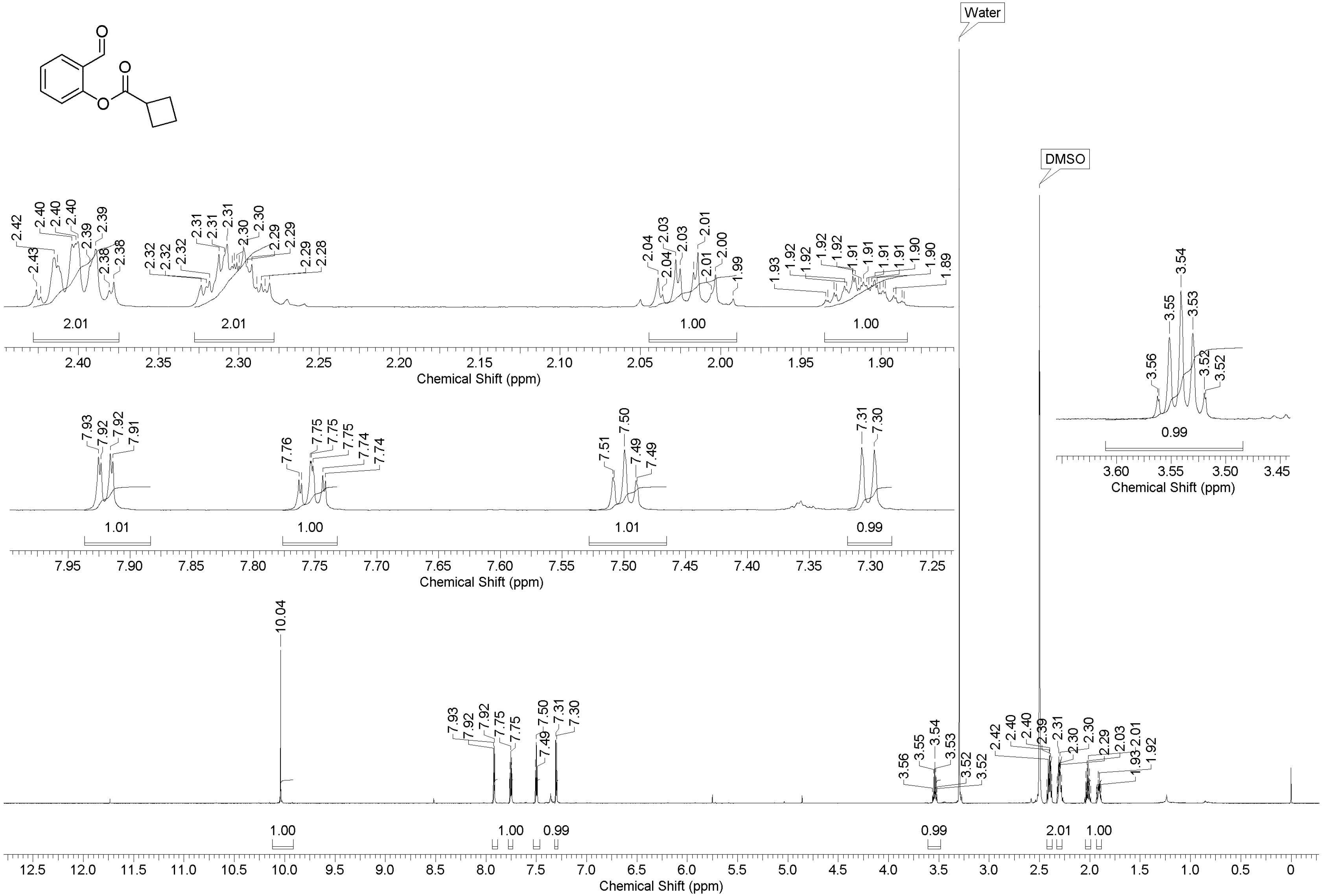

3f

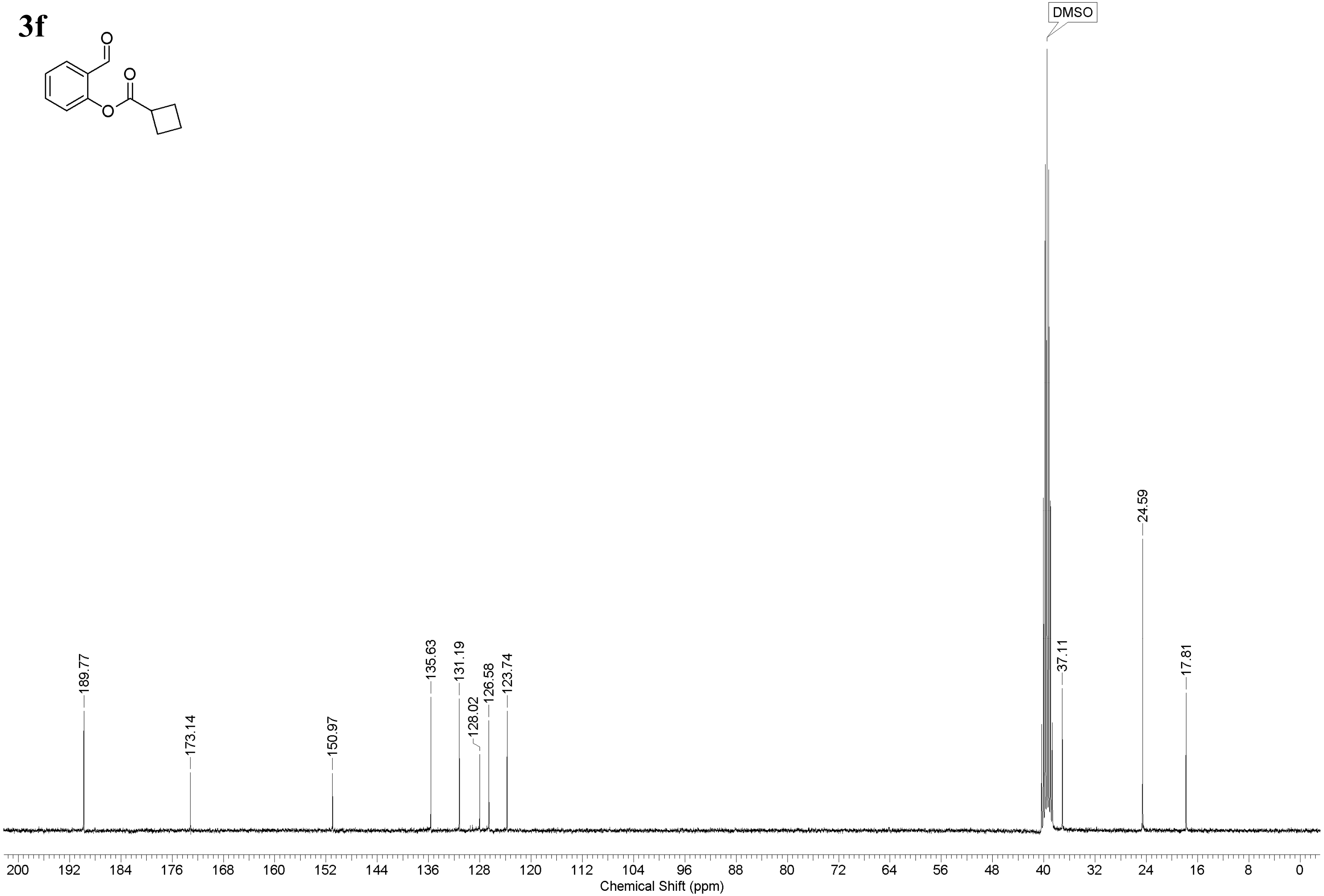
DMSO
189.77
173.14
150.97
135.63
131.19
128.02
126.58
123.74
37.11
24.59
17.81
200
192
184
176
168
160
152
144
136
128
120
112
104
96
88
80
72
64
56
48
40
32
24
16
8
0
Chemical Shift (ppm)

3g

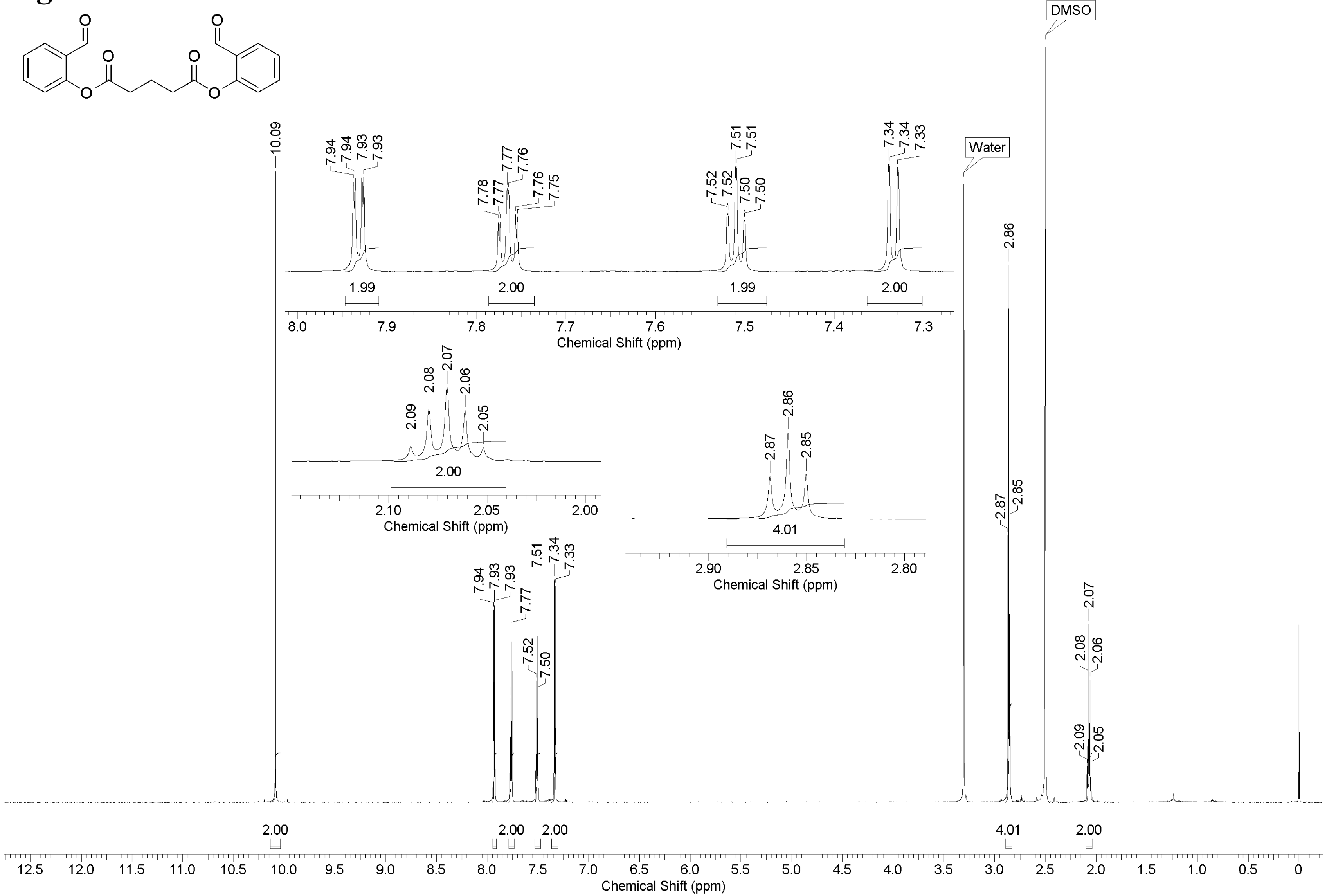
DMSO
Water
10.09
7.94
7.93
7.93
7.77
7.52
7.51
7.50
7.34
7.33
2.87
2.86
2.85
2.09
2.08
2.07
2.06
2.05
1.99
2.00
1.99
2.00
4.01
2.00
Chemical Shift (ppm)

3n

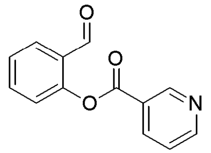

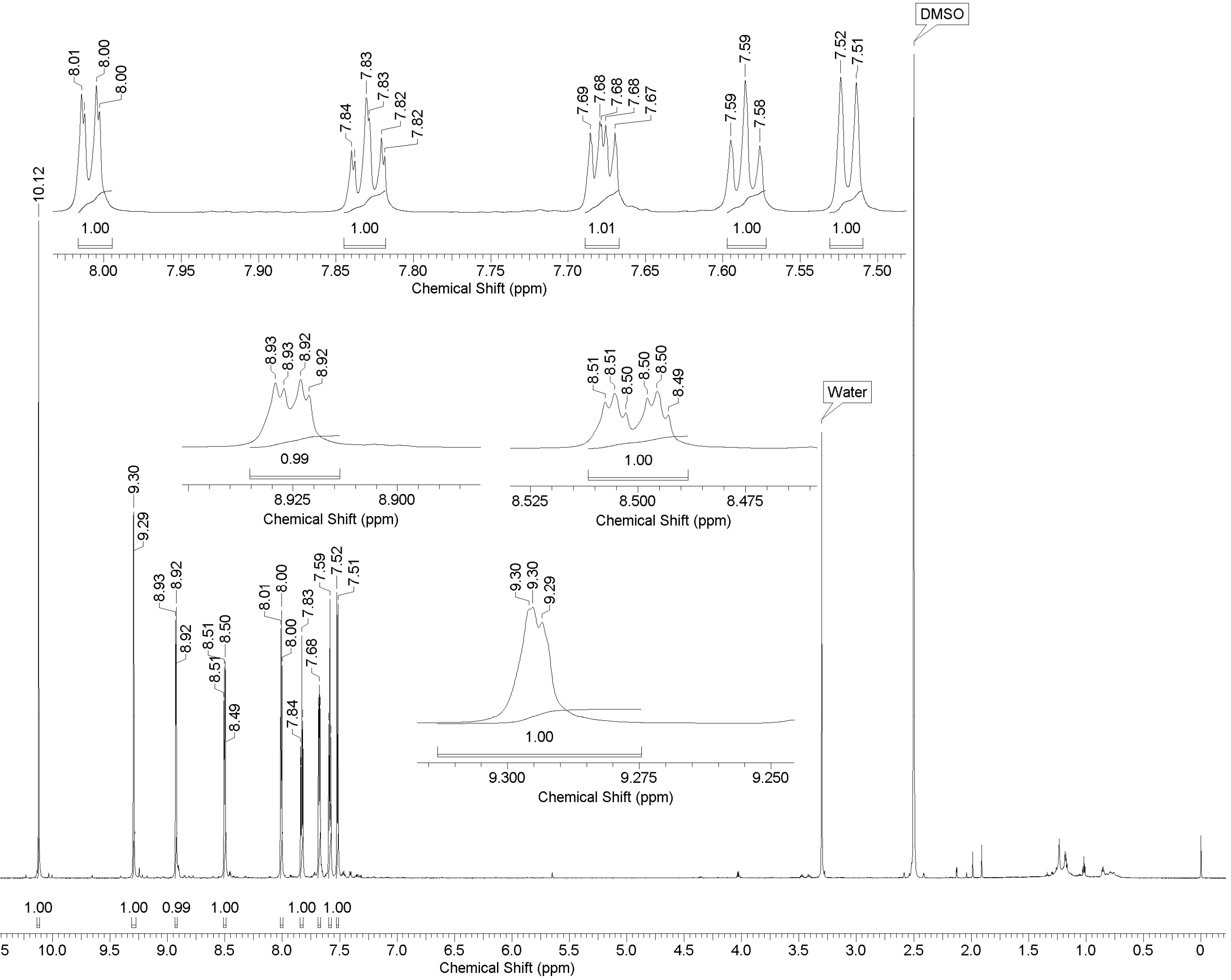

DMSO
Water
10.12
9.30
9.29
8.93
8.92
8.51
8.50
8.49
8.01
8.00
7.84
7.83
7.82
7.69
7.68
7.67
7.59
7.58
7.52
7.51
1.00
1.01
0.99
Chemical Shift (ppm)

3n

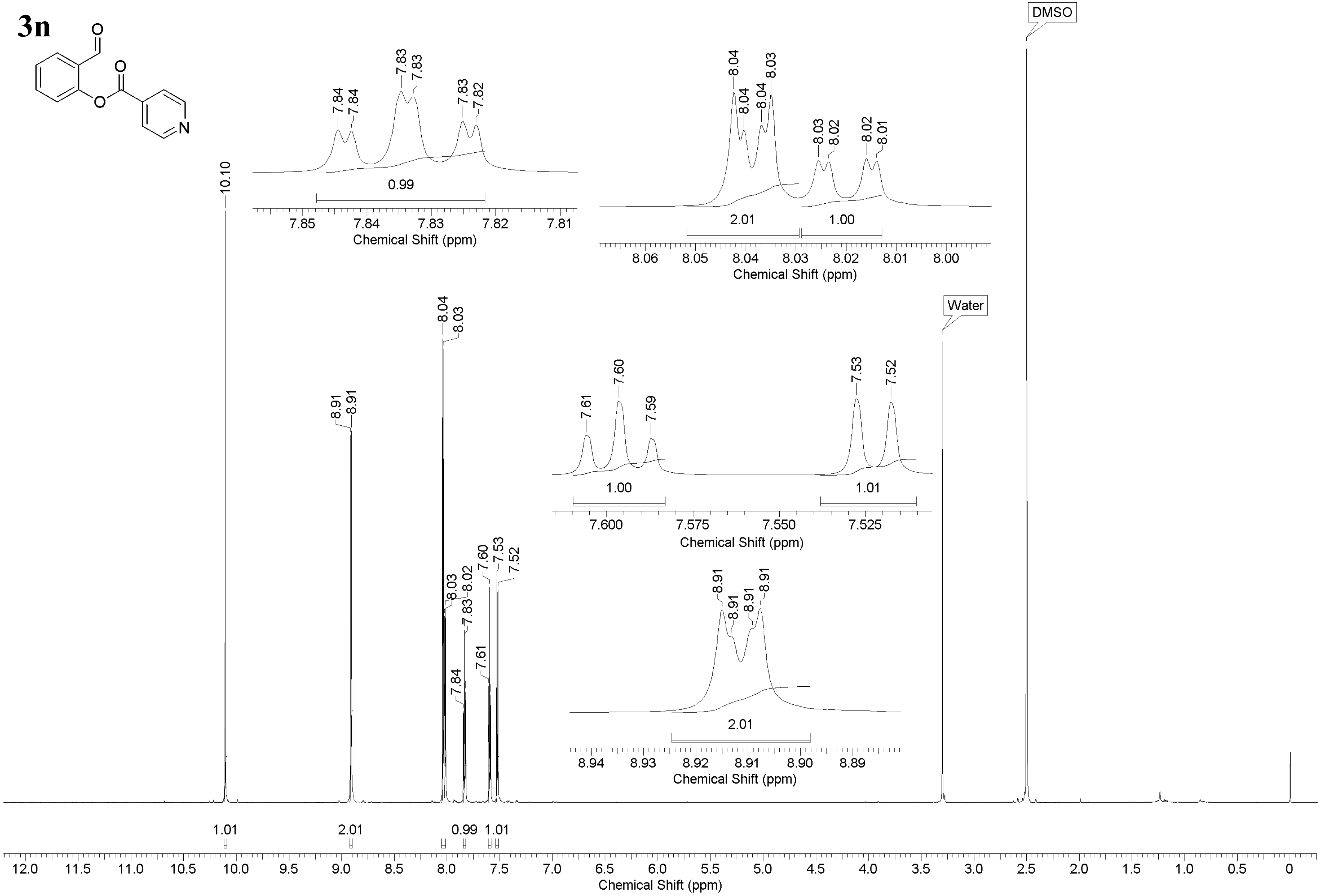
DMSO
Water
10.10
8.91
8.91
8.04
8.03
8.03
8.02
7.84
7.83
7.61
7.60
7.53
7.52
1.01
2.01
0.99
1.01
Chemical Shift (ppm)
12.0 11.5 11.0 10.5 10.0 9.5 9.0 8.5 8.0 7.5 7.0 6.5 6.0 5.5 5.0 4.5 4.0 3.5 3.0 2.5 2.0 1.5 1.0 0.5 0

3o

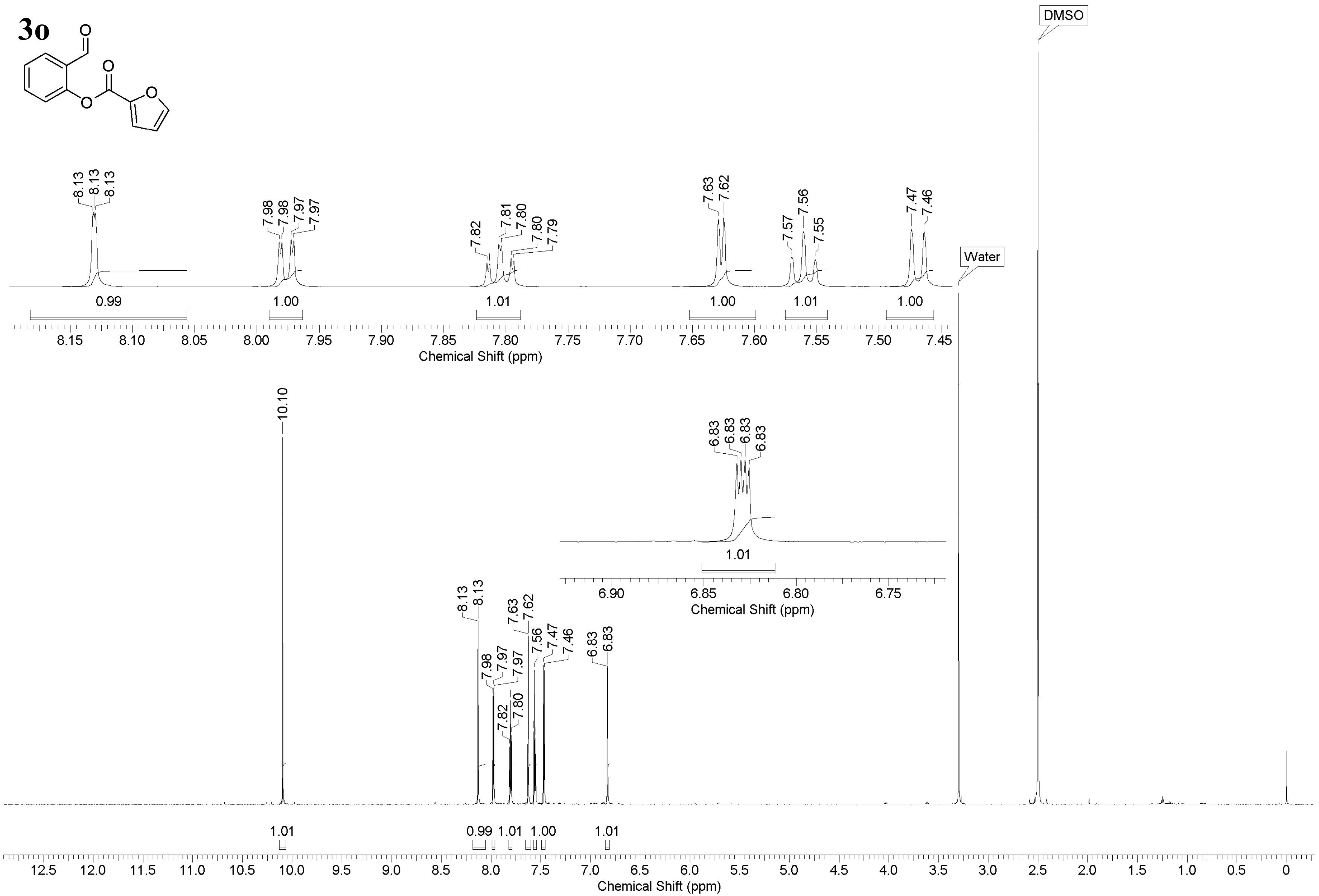
DMSO
Water
Chemical Shift (ppm)
Chemical Shift (ppm)
Chemical Shift (ppm)

4a

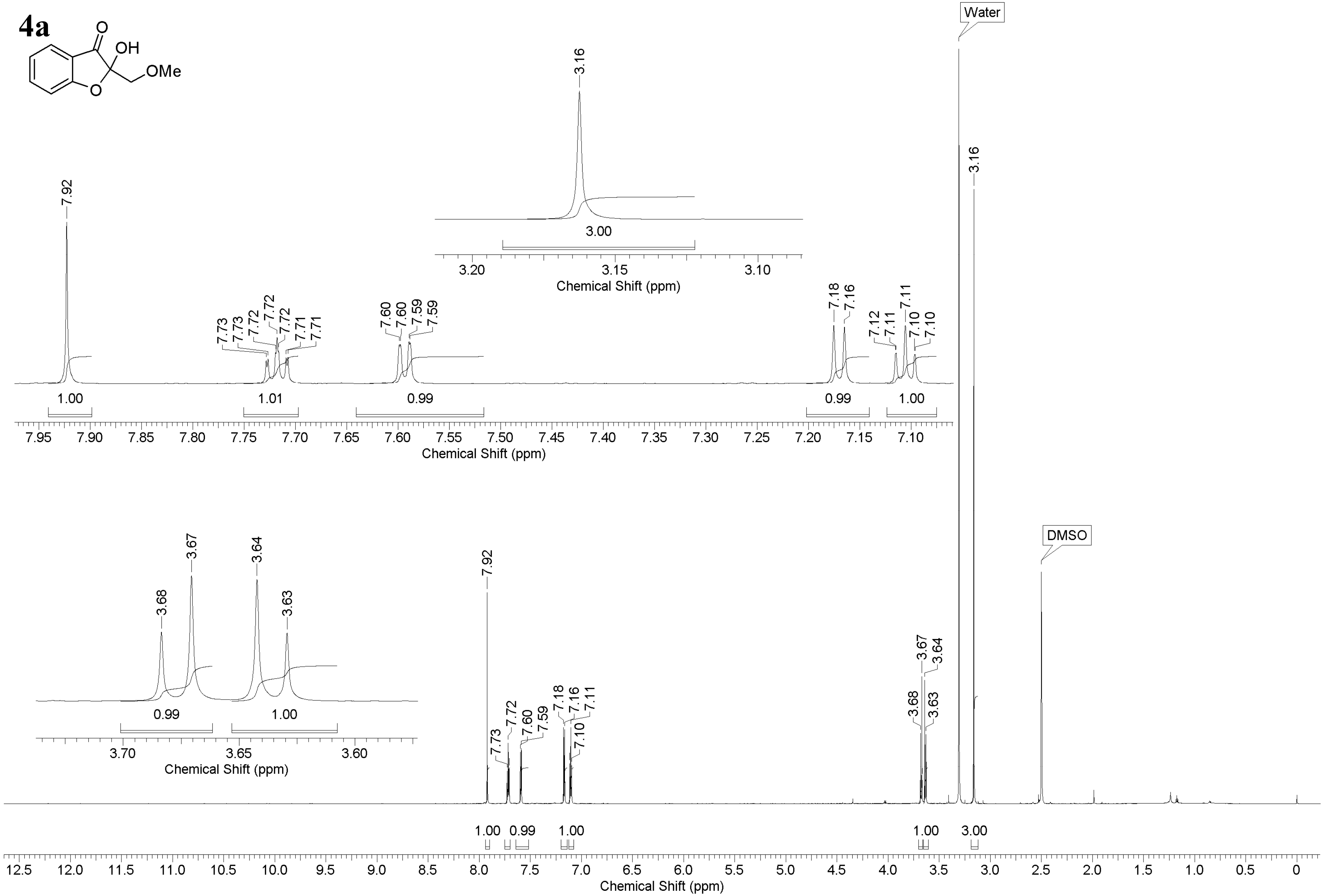

4a

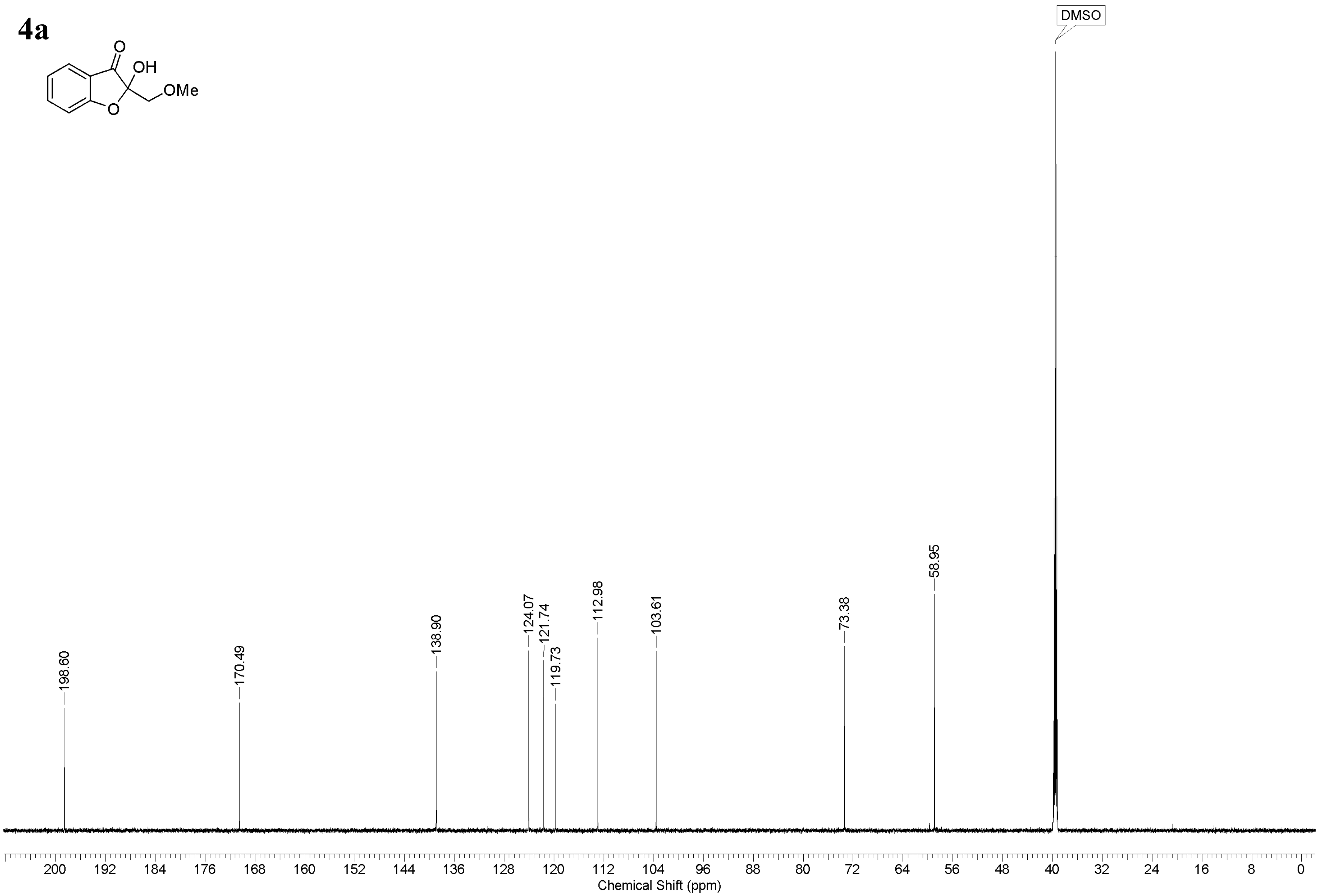
OH
OMe
O
O
DMSO
198.60
170.49
138.90
124.07
121.74
119.73
112.98
103.61
73.38
58.95
200 192 184 176 168 160 152 144 136 128 120 112 104 96 88 80 72 64 56 48 40 32 24 16 8 0
Chemical Shift (ppm)

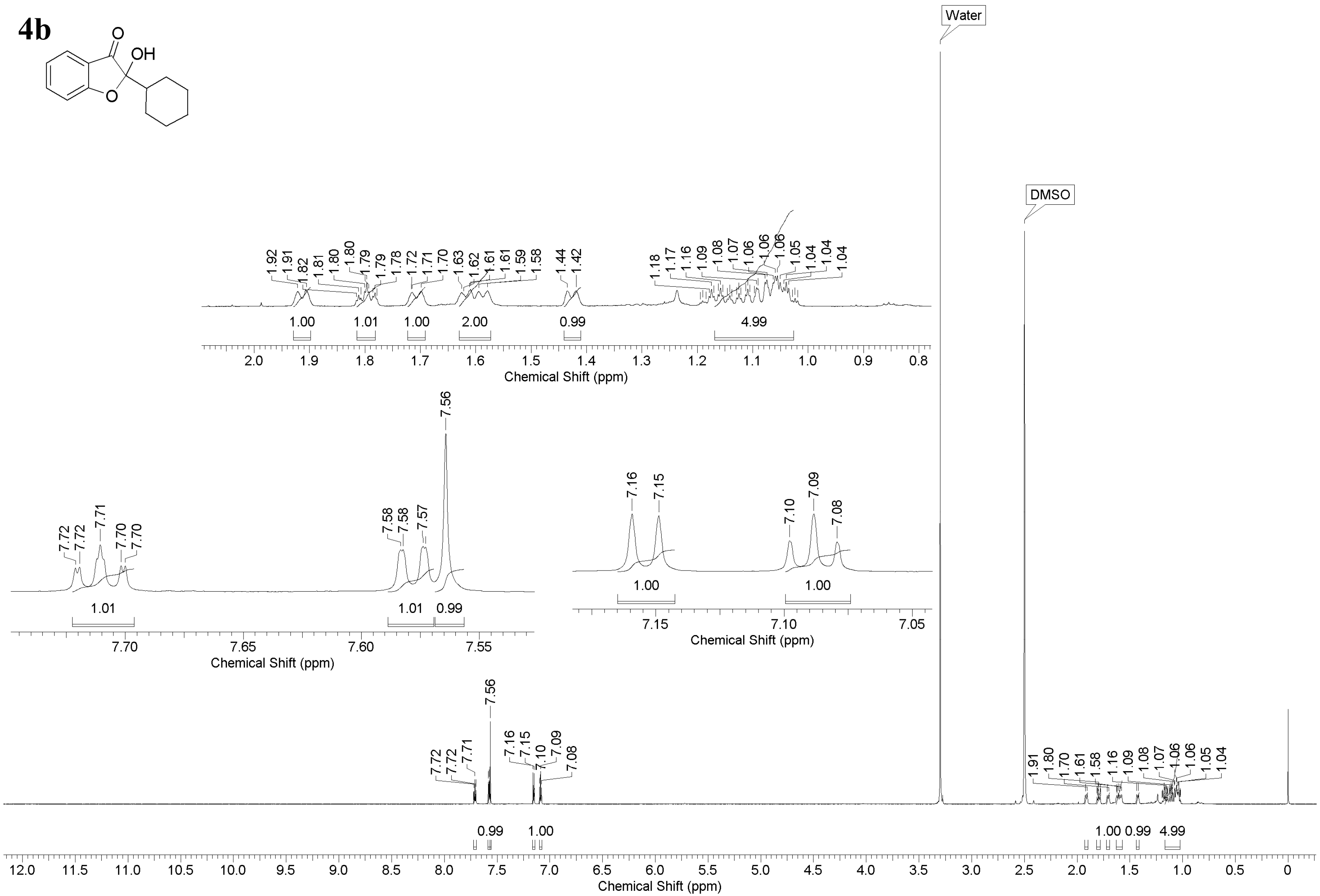

4b
O
OH
O
Water
DMSO
Chemical Shift (ppm)

4b

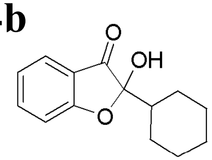

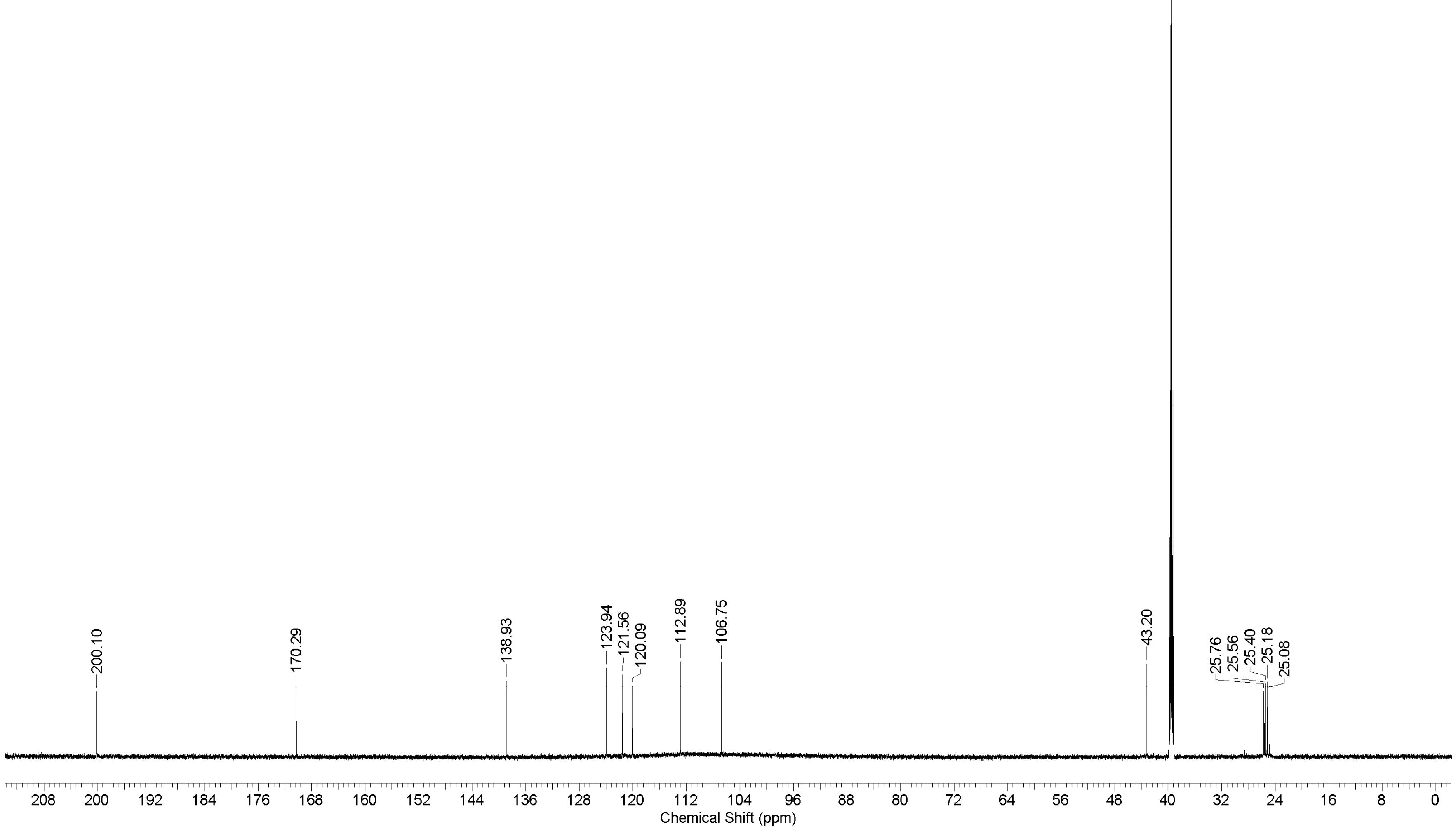

DMSO
200.10
170.29
138.93
123.94
121.56
120.09
112.89
106.75
43.20
25.76
25.56
25.40
25.18
25.08
208
200
192
184
176
168
160
152
144
136
128
120
112
104
96
88
80
72
64
56
48
40
32
24
16
8
0
Chemical Shift (ppm)

4c

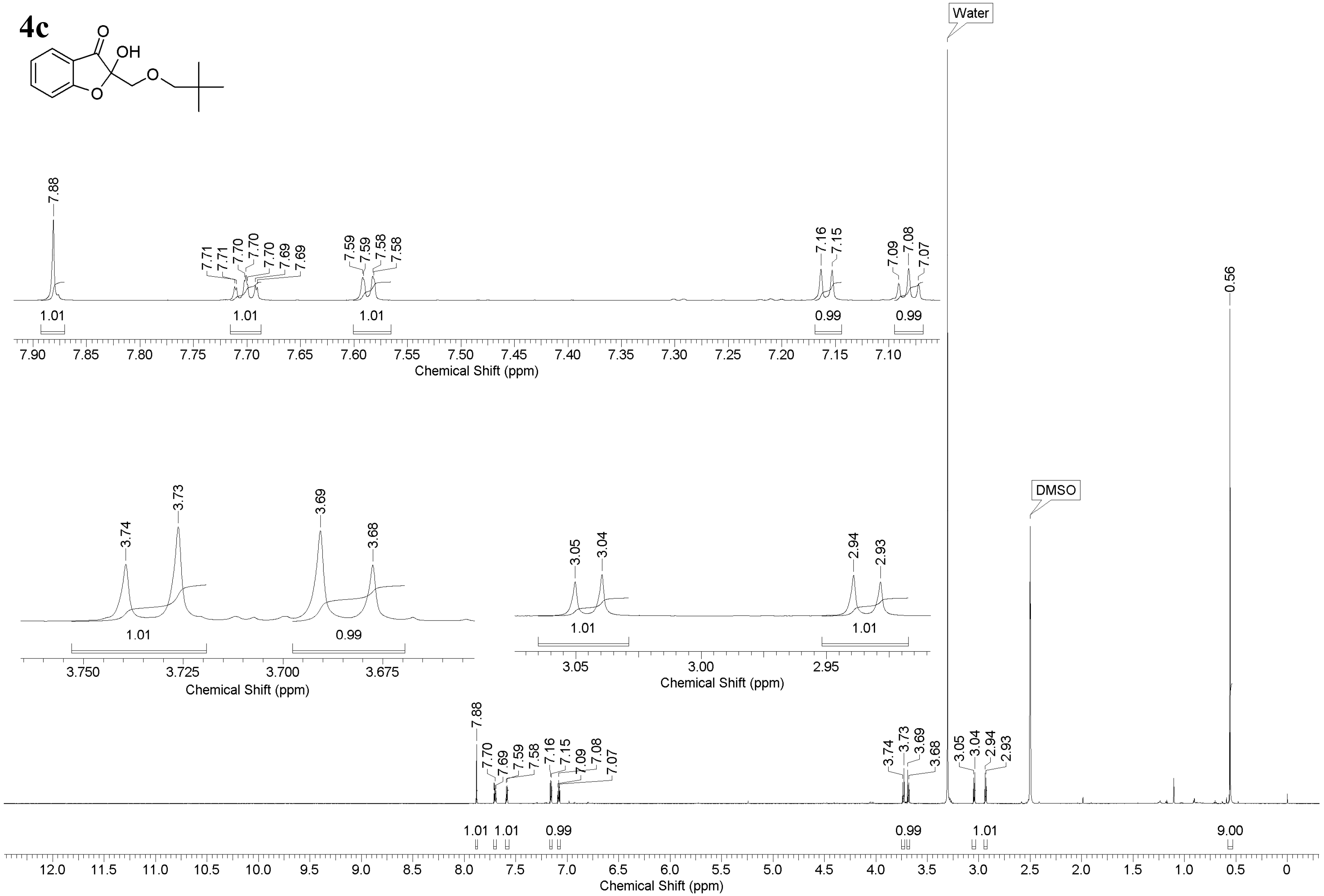
O
OH
O
O
Water
DMSO
7.88
7.71
7.71
7.70
7.70
7.70
7.69
7.69
7.59
7.59
7.58
7.58
7.16
7.15
7.09
7.08
7.07
0.56
1.01
1.01
1.01
0.99
0.99
Chemical Shift (ppm)
3.74
3.73
3.69
3.68
1.01
0.99
Chemical Shift (ppm)
3.05
3.04
2.94
2.93
1.01
1.01
Chemical Shift (ppm)
1.01 1.01
0.99
0.99
1.01
9.00
Chemical Shift (ppm)

4c

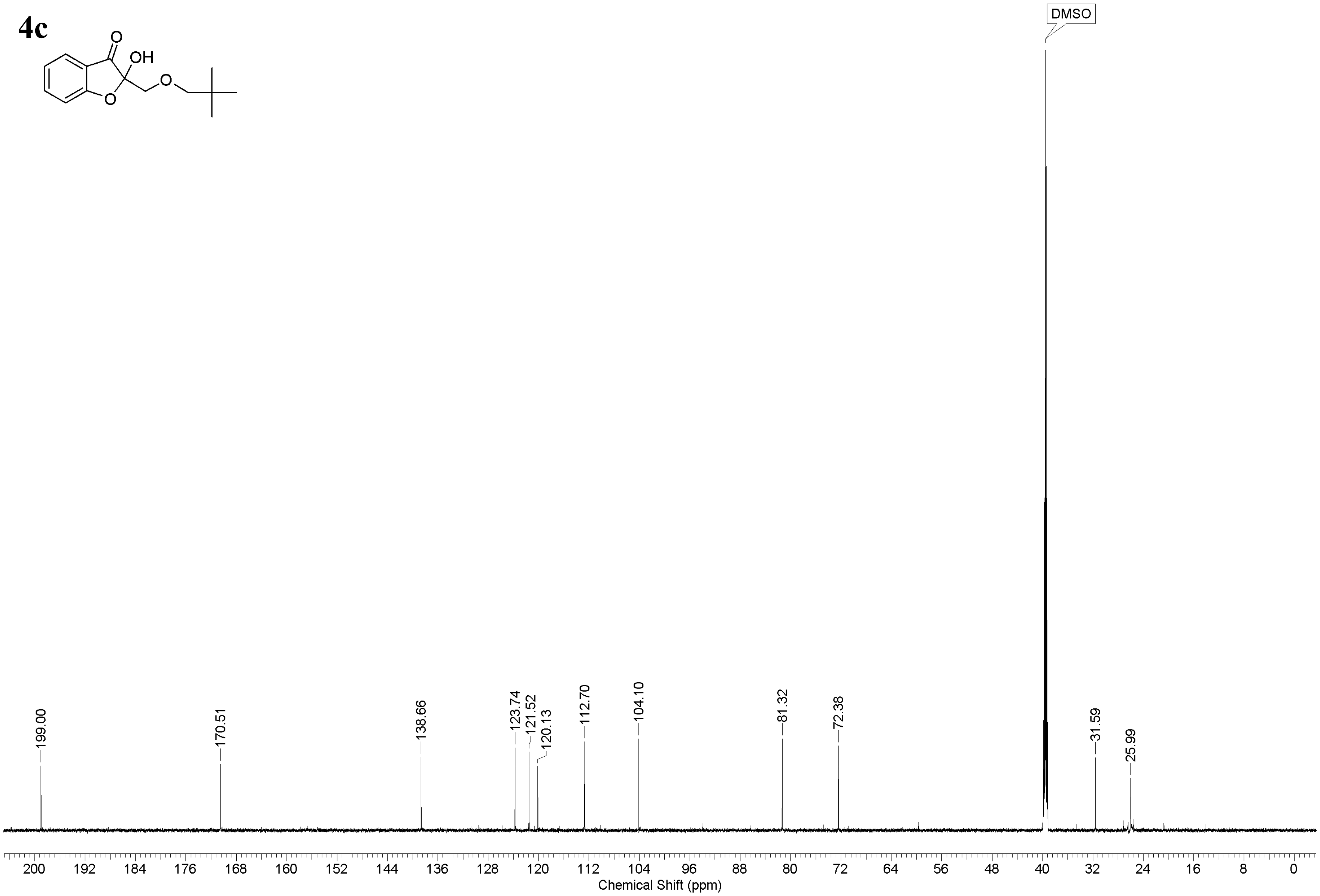
O
OH
O
O
DMSO
199.00
170.51
138.66
123.74
121.52
120.13
112.70
104.10
81.32
72.38
31.59
25.99
200 192 184 176 168 160 152 144 136 128 120 112 104 96 88 80 72 64 56 48 40 32 24 16 8 0
Chemical Shift (ppm)

4d

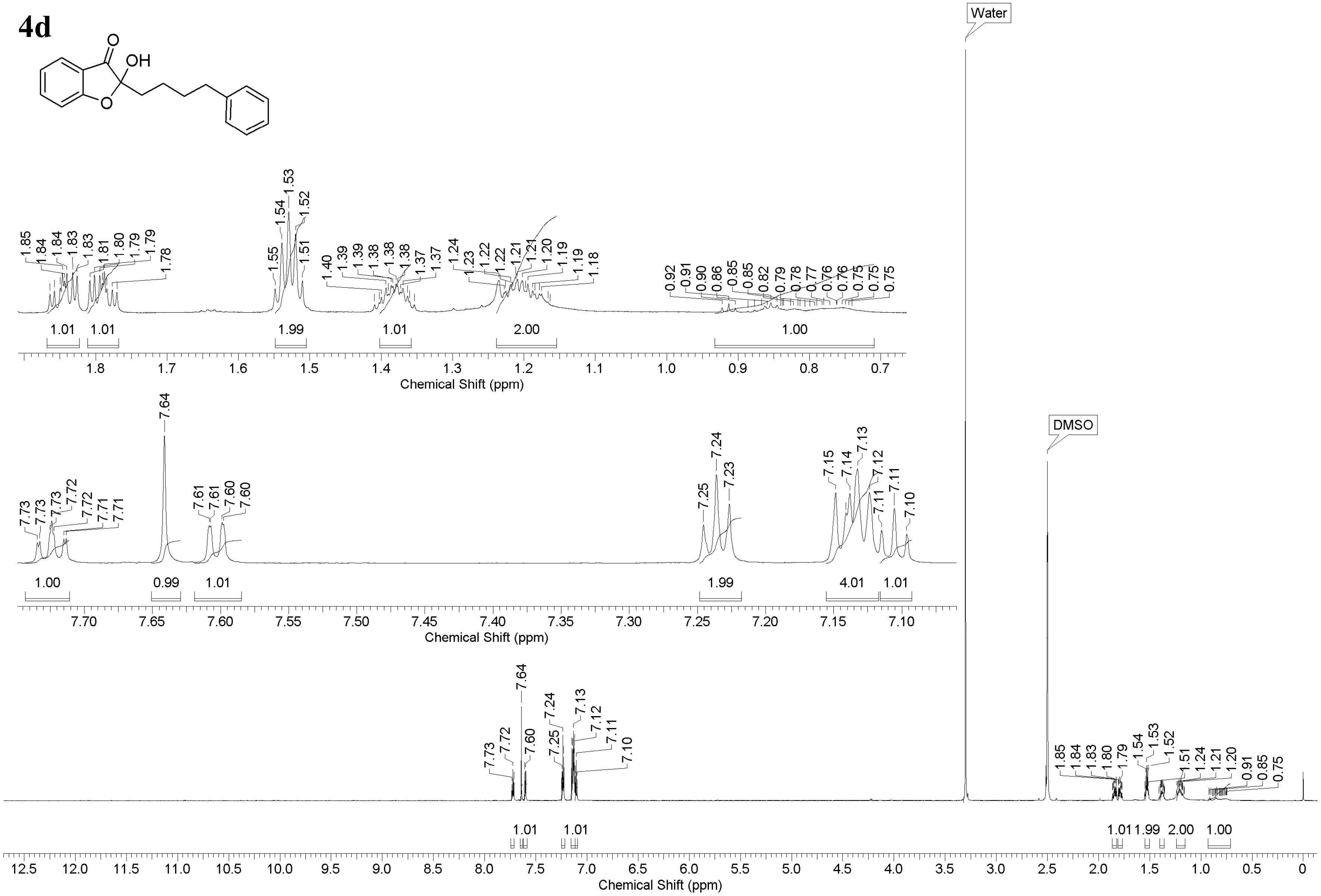

O
OH
O
Water
DMSO
Chemical Shift (ppm)
Chemical Shift (ppm)
Chemical Shift (ppm)

4d

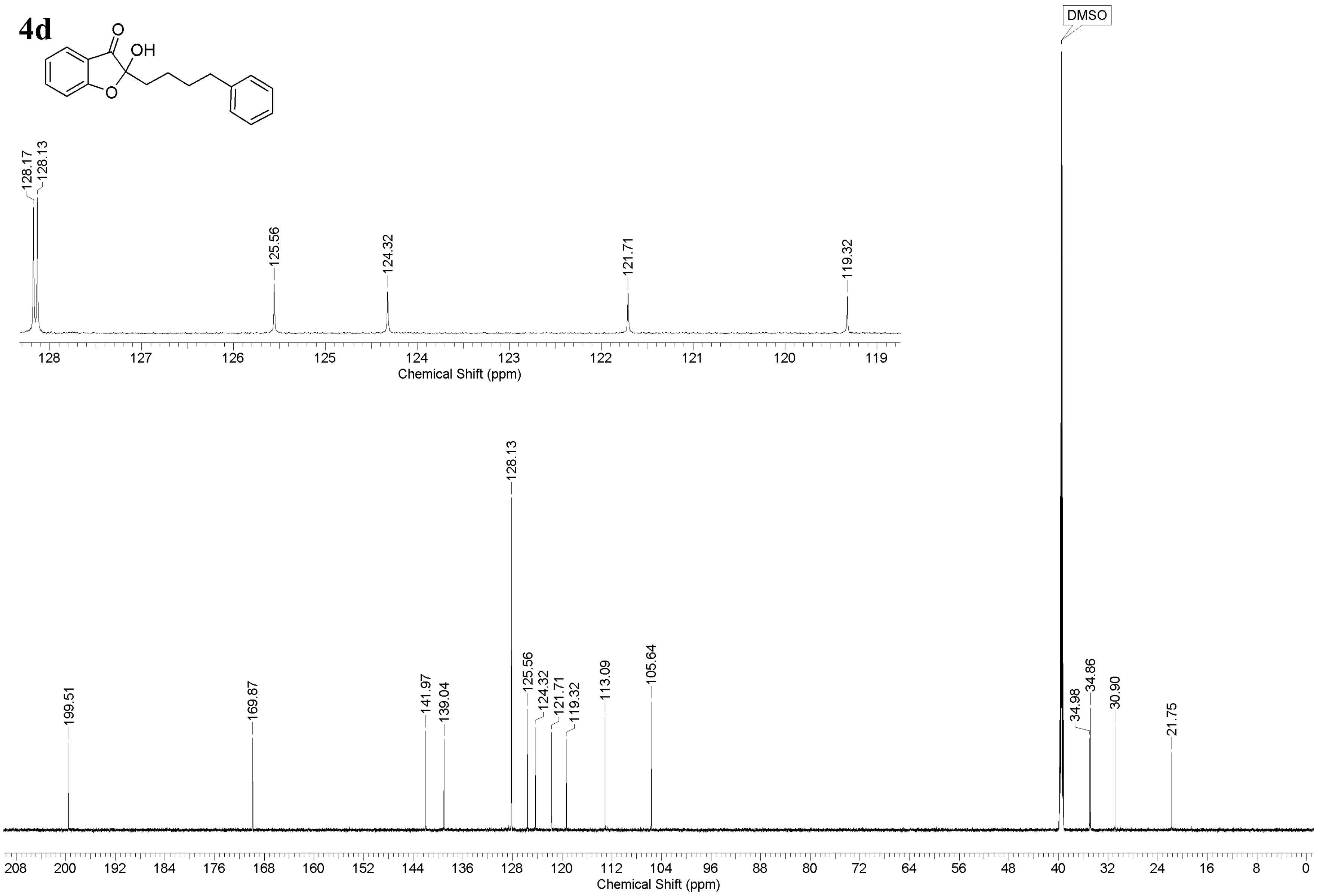
OH
O
O
128.17
128.13
125.56
124.32
121.71
119.32
Chemical Shift (ppm)
DMSO
199.51
169.87
141.97
139.04
128.13
125.56
124.32
121.71
119.32
113.09
105.64
34.98
34.86
30.90
21.75
Chemical Shift (ppm)

4e

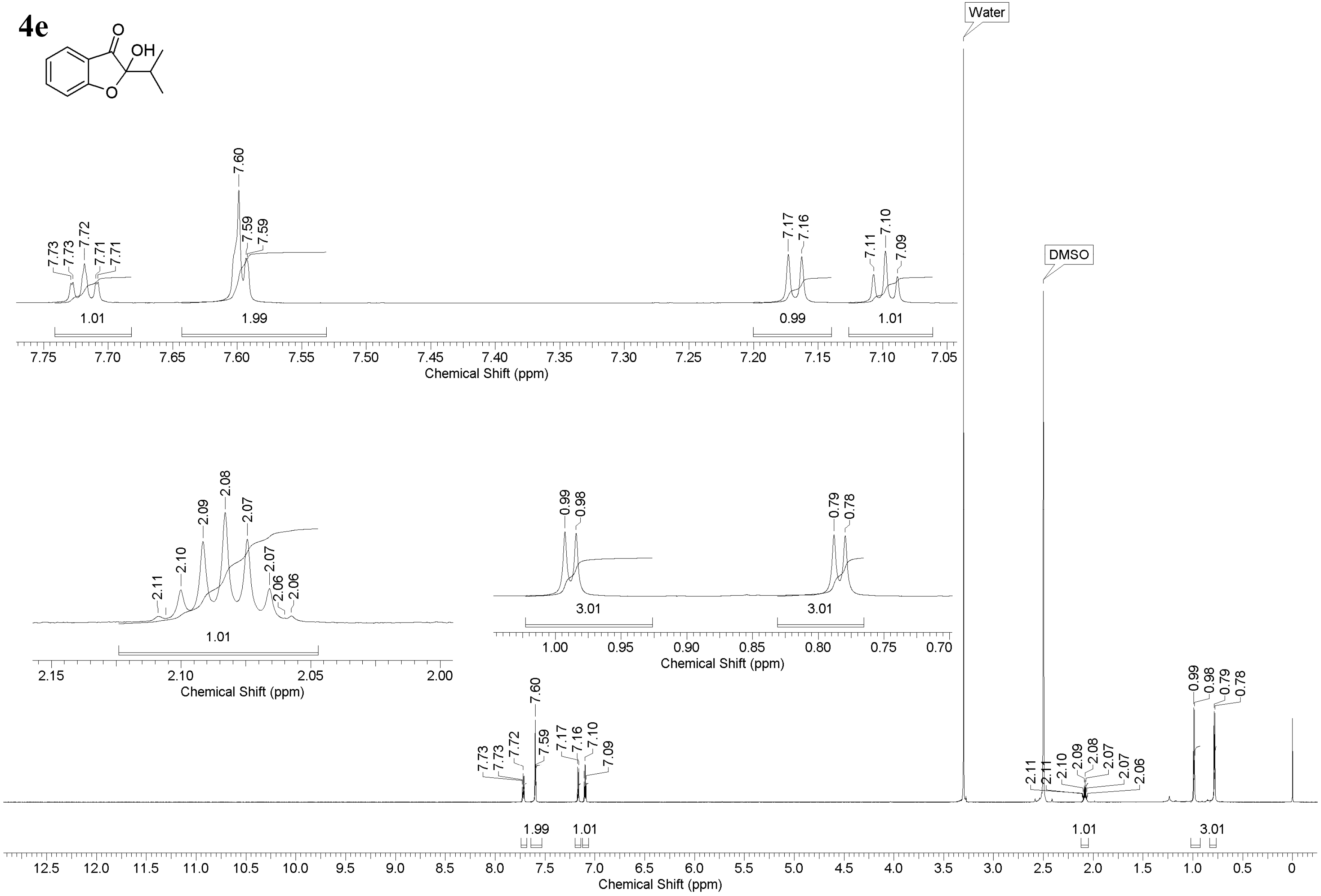

4e

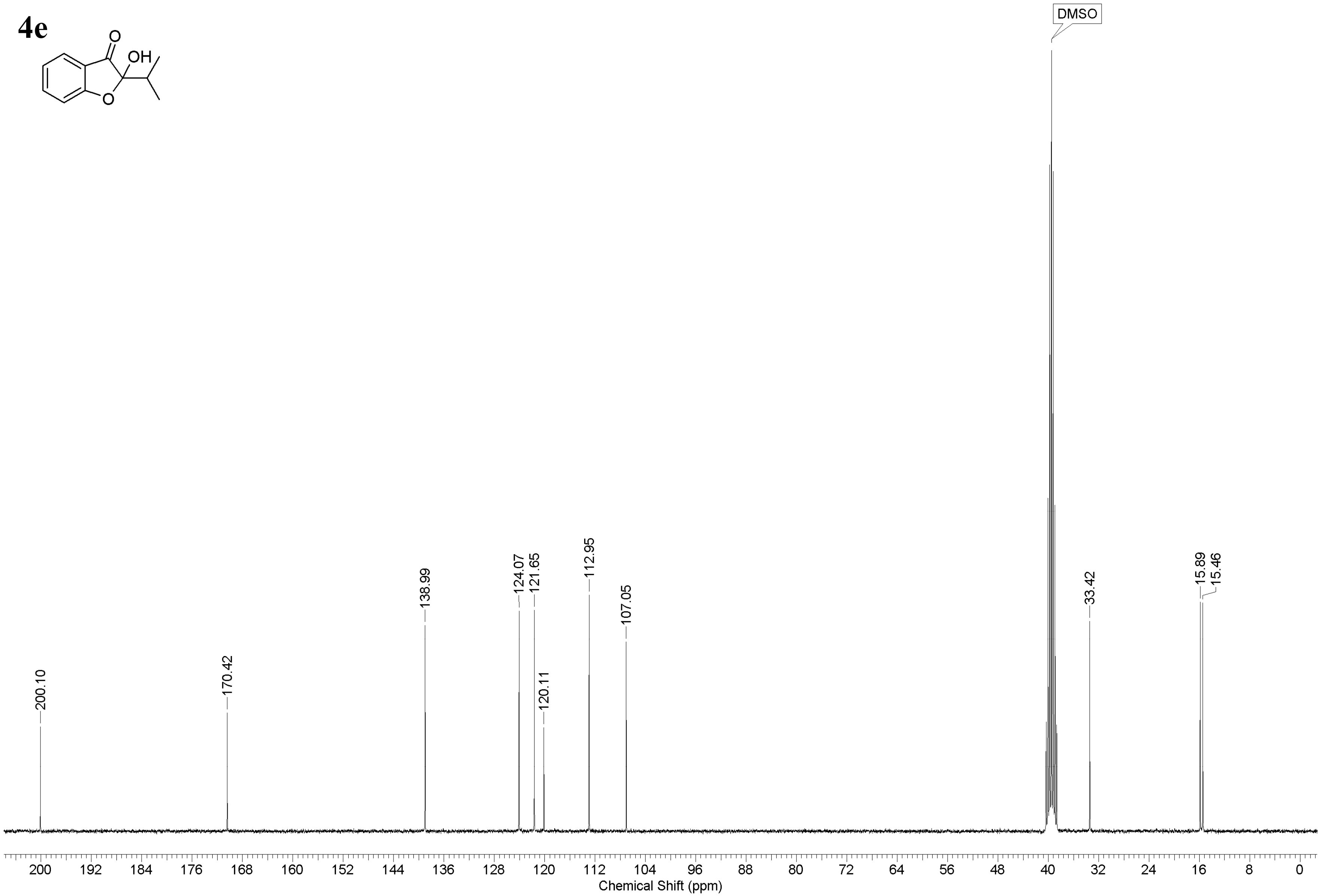
O
OH
O
DMSO
200.10
170.42
138.99
124.07
121.65
120.11
112.95
107.05
33.42
15.89
15.46
200
192
184
176
168
160
152
144
136
128
120
112
104
96
88
80
72
64
56
48
40
32
24
16
8
0
Chemical Shift (ppm)

4f

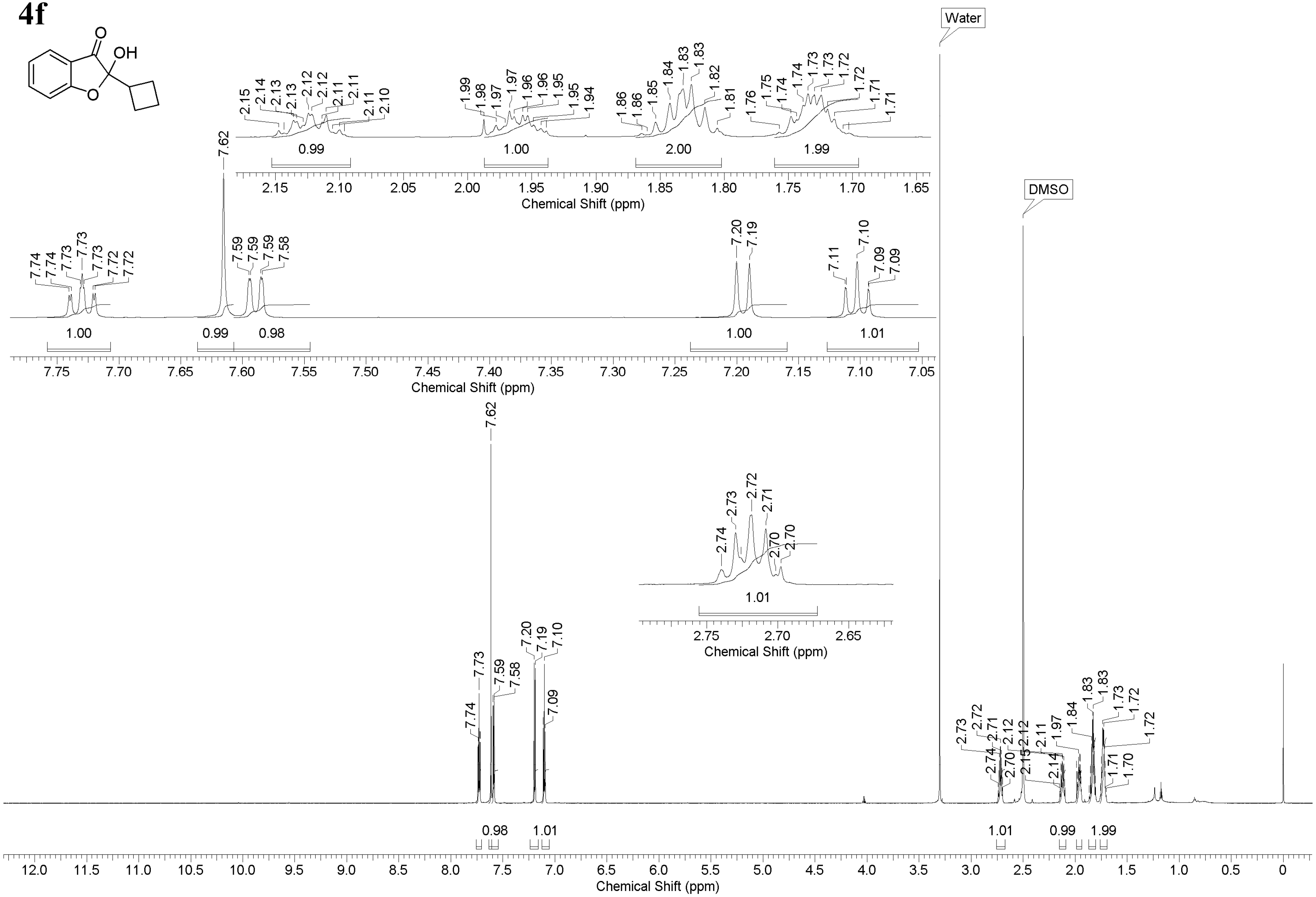

4f

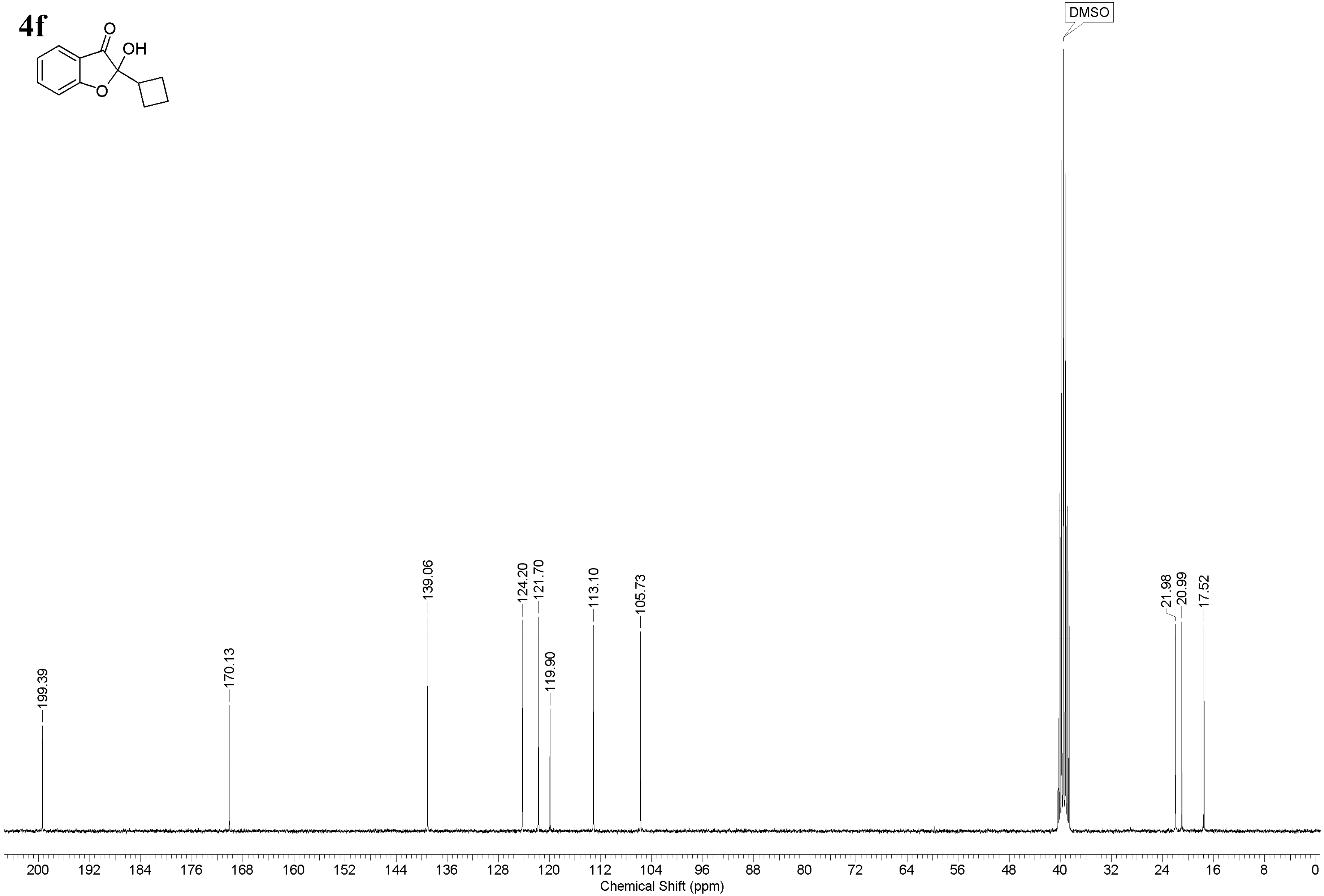
O
OH
O
DMSO
199.39
170.13
139.06
124.20
121.70
119.90
113.10
105.73
21.98
20.99
17.52
200 192 184 176 168 160 152 144 136 128 120 112 104 96 88 80 72 64 56 48 40 32 24 16 8 0
Chemical Shift (ppm)

4g

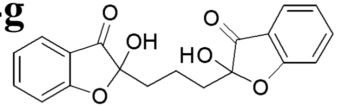

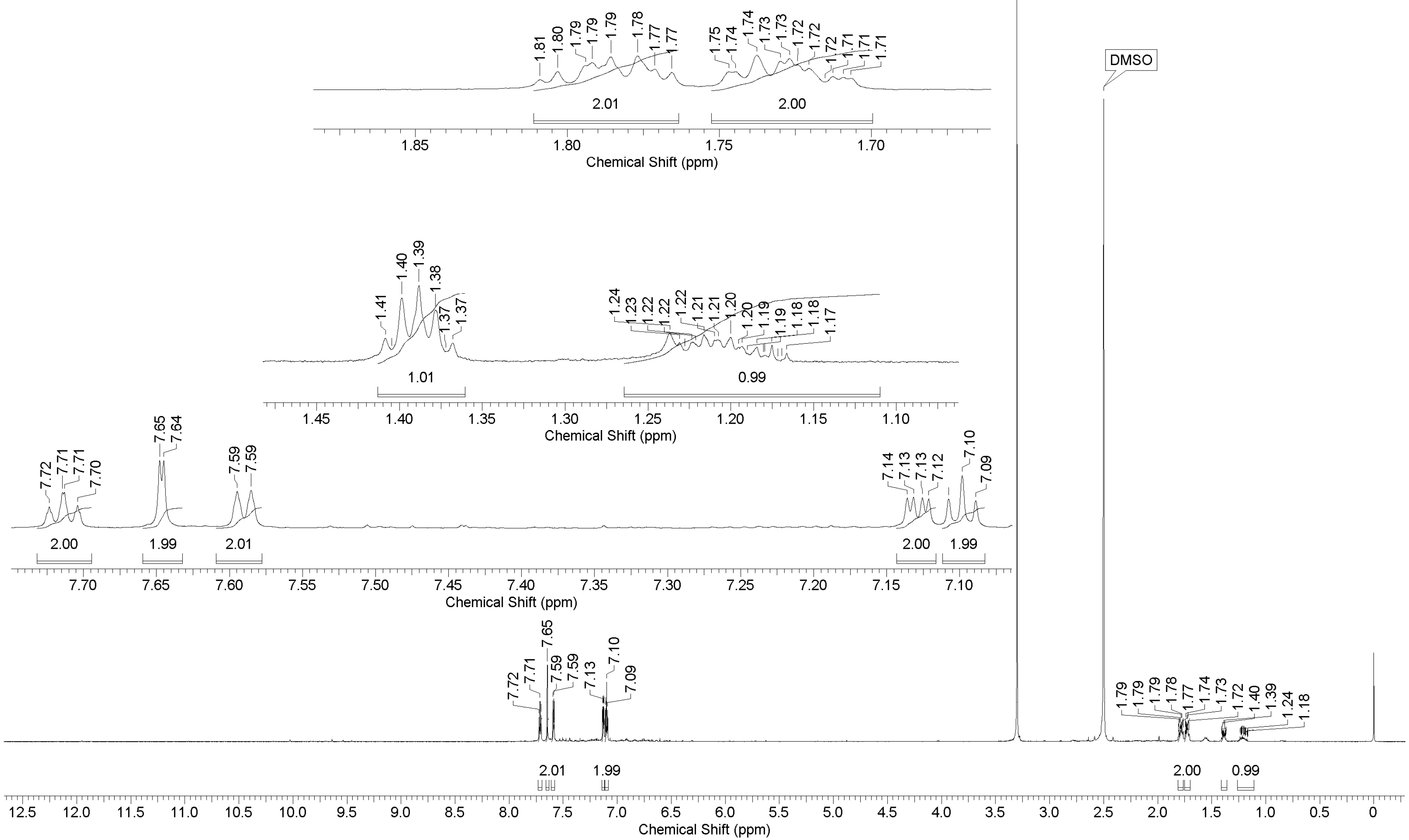

4g

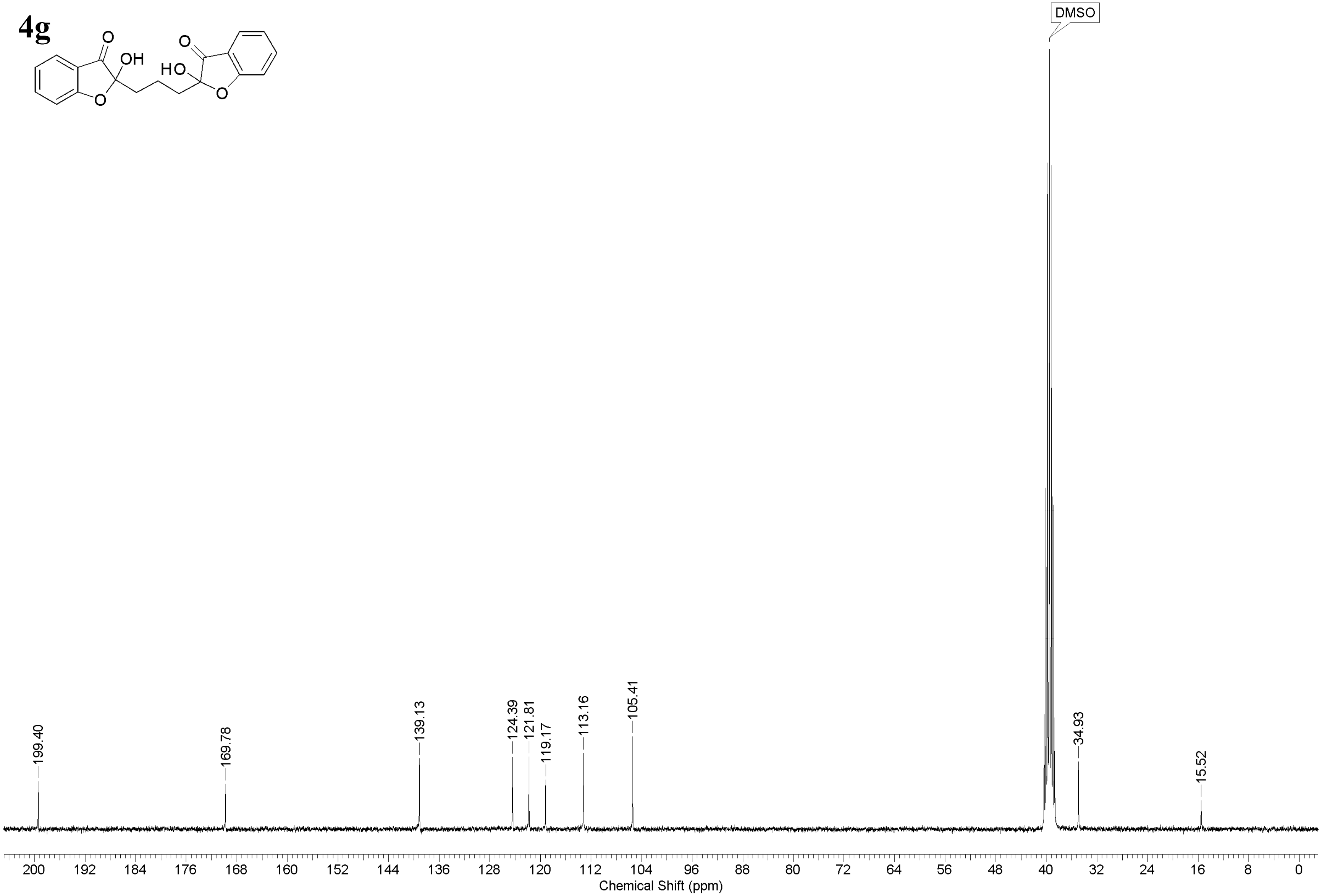
O
OH
O
HO
O
O
DMSO
199.40
169.78
139.13
124.39
121.81
119.17
113.16
105.41
34.93
15.52
200
192
184
176
168
160
152
144
136
128
120
112
104
96
88
80
72
64
56
48
40
32
24
16
8
0
Chemical Shift (ppm)

4h

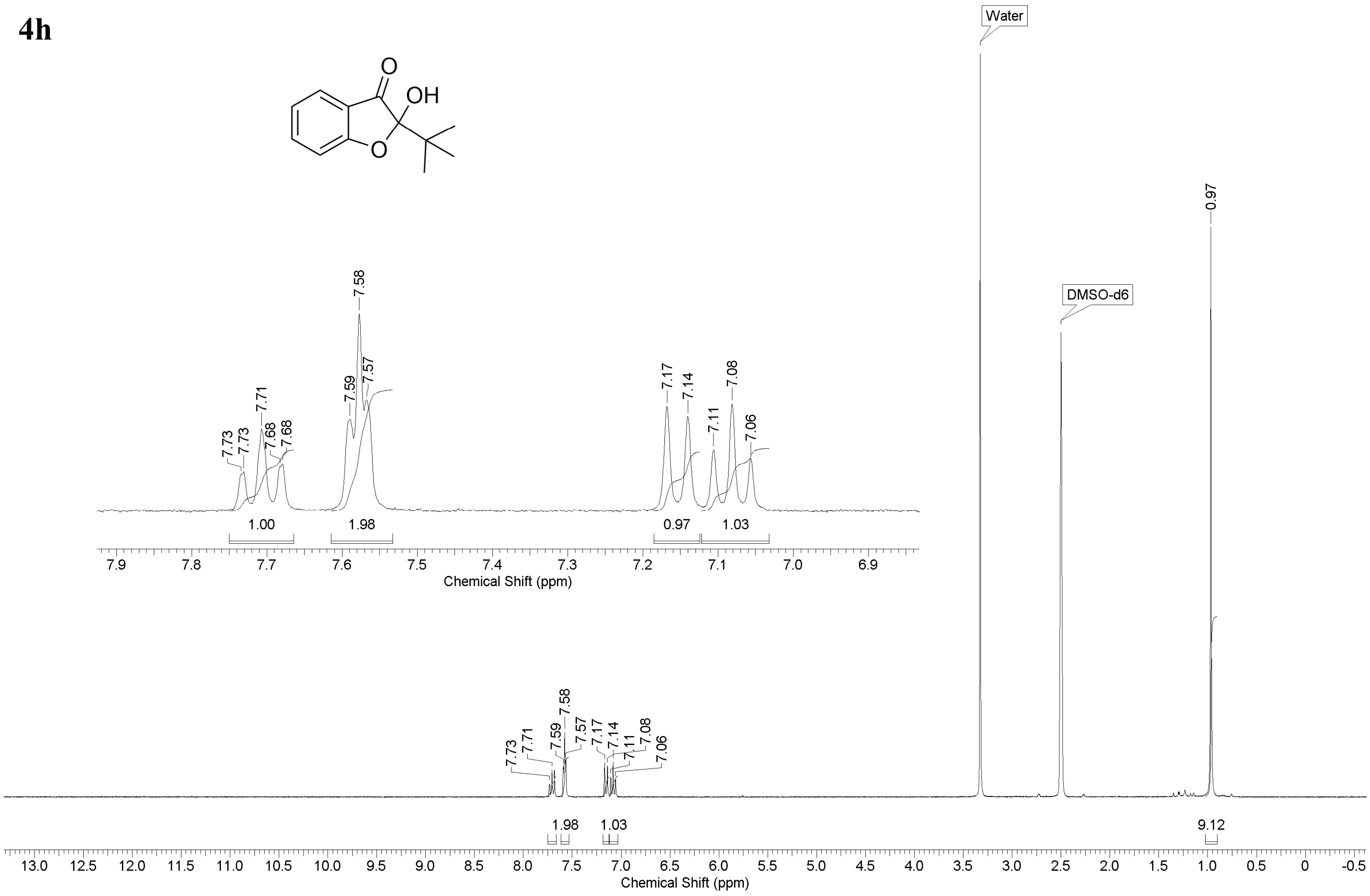
O
OH
O
Water
DMSO-d6
0.97
7.58
7.59
7.57
7.73
7.73
7.71
7.68
7.68
7.17
7.14
7.11
7.08
7.06
1.00
1.98
0.97
1.03
7.9
7.8
7.7
7.6
7.5
7.4
7.3
7.2
7.1
7.0
6.9
Chemical Shift (ppm)
7.73
7.71
7.59
7.58
7.57
7.17
7.14
7.11
7.08
7.06
1.98
1.03
9.12
13.0
12.5
12.0
11.5
11.0
10.5
10.0
9.5
9.0
8.5
8.0
7.5
7.0
6.5
6.0
5.5
5.0
4.5
4.0
3.5
3.0
2.5
2.0
1.5
1.0
0.5
0
-0.5
Chemical Shift (ppm)

4h

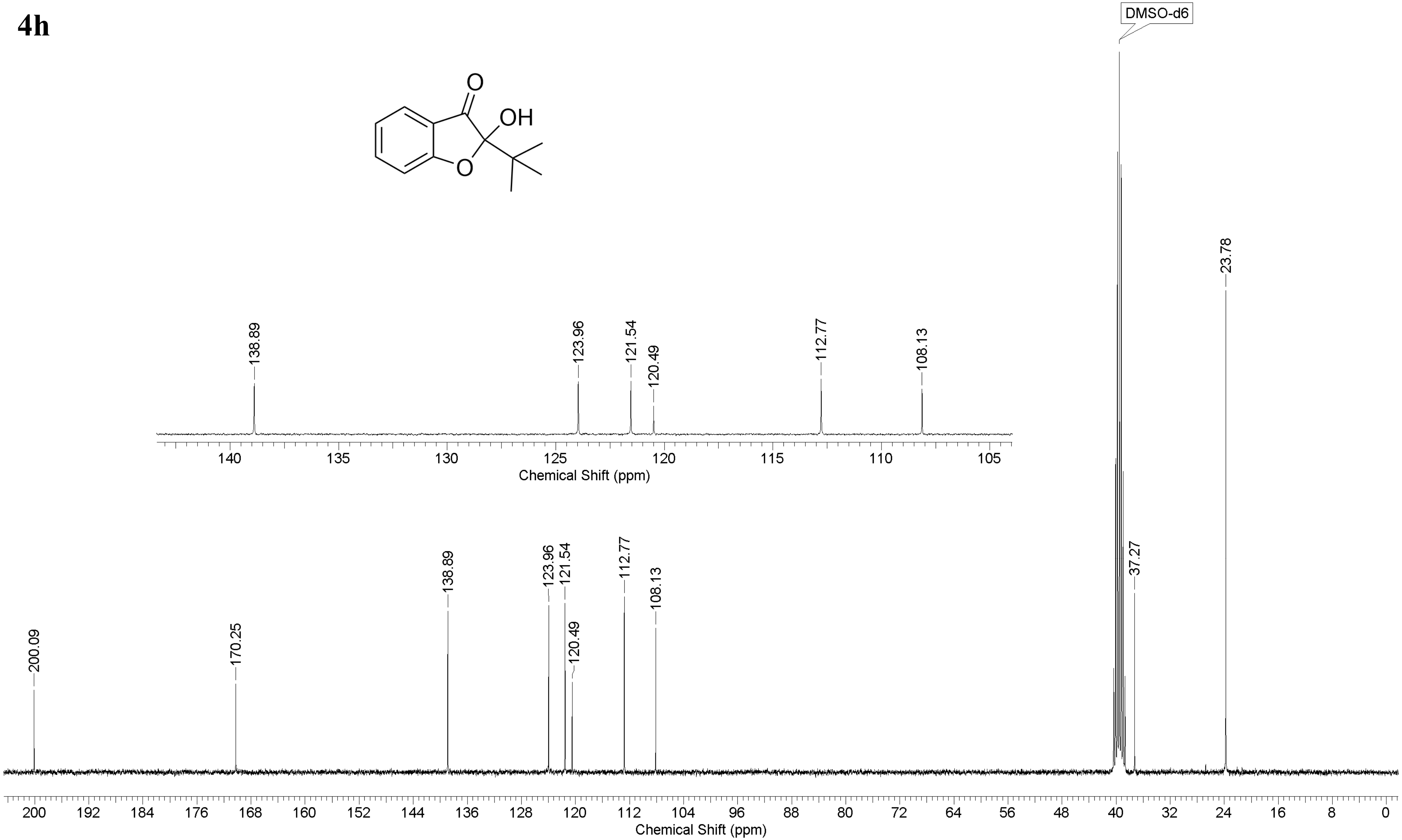
O
OH
O
DMSO-d6
23.78
138.89
123.96
121.54
120.49
112.77
108.13
140
135
130
125
120
115
110
105
Chemical Shift (ppm)
200.09
170.25
138.89
123.96
121.54
120.49
112.77
108.13
37.27
200
192
184
176
168
160
152
144
136
128
120
112
104
96
88
80
72
64
56
48
40
32
24
16
8
0
Chemical Shift (ppm)

5b

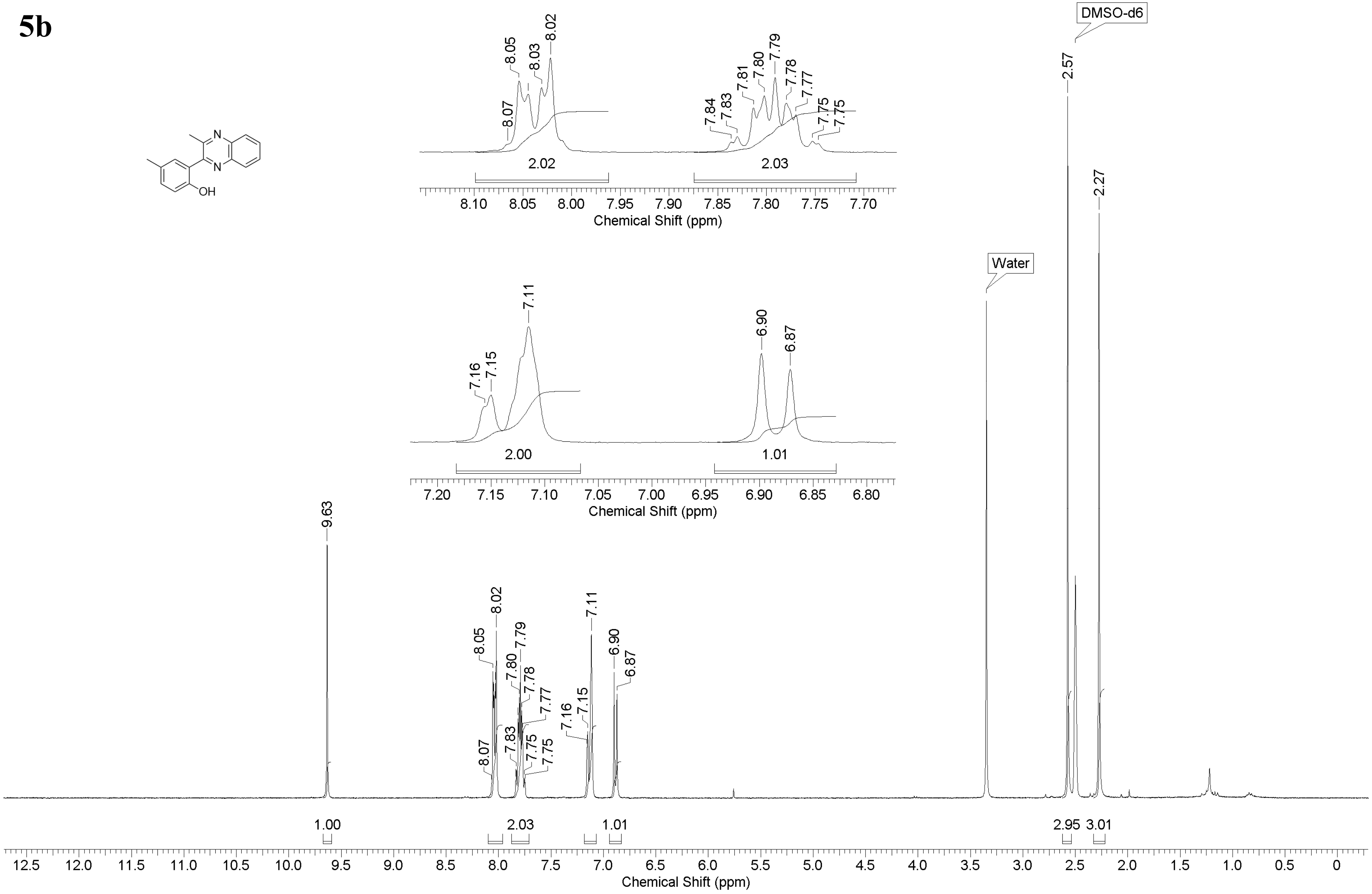
DMSO-d6
Water
9.63
8.05
8.02
7.79
7.11
6.90
6.87
1.00
2.03
1.01
2.95
3.01
2.57
2.27
2.02
2.00
Chemical Shift (ppm)

5b

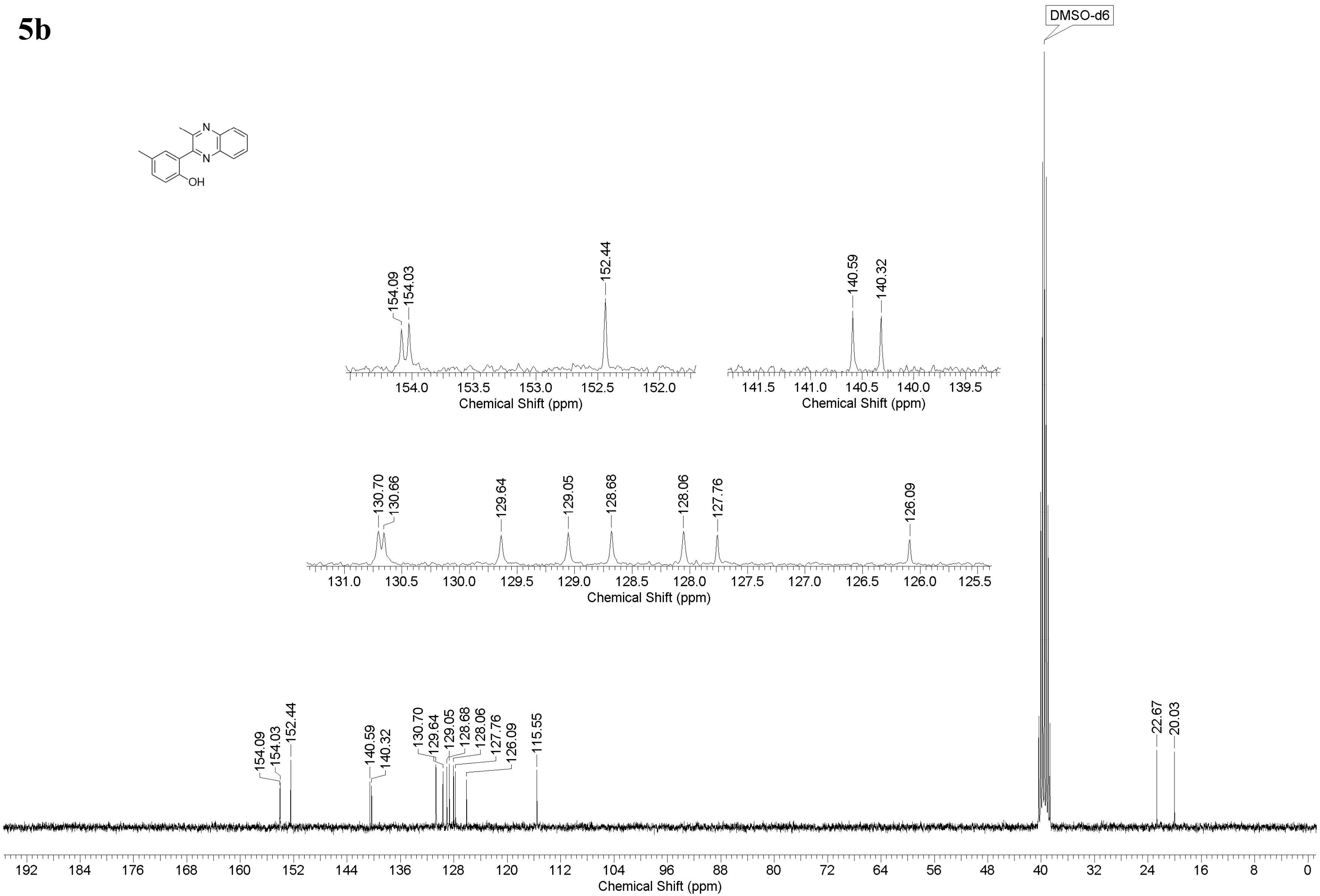

OH
N
N
DMSO-d6
154.09
154.03
152.44
140.59
140.32
130.70
130.66
129.64
129.05
128.68
128.06
127.76
126.09
115.55
22.67
20.03
Chemical Shift (ppm)

5d

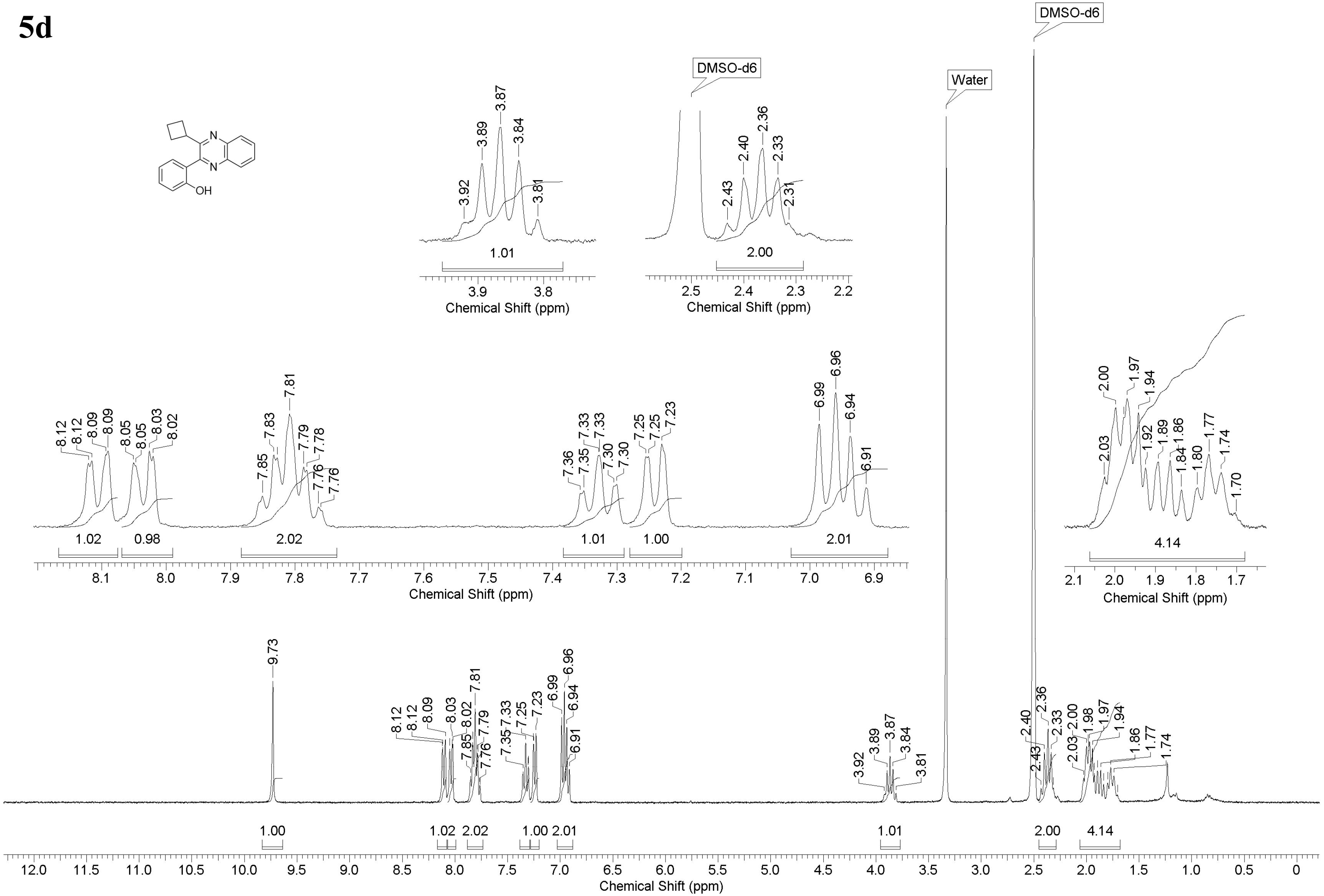

5d

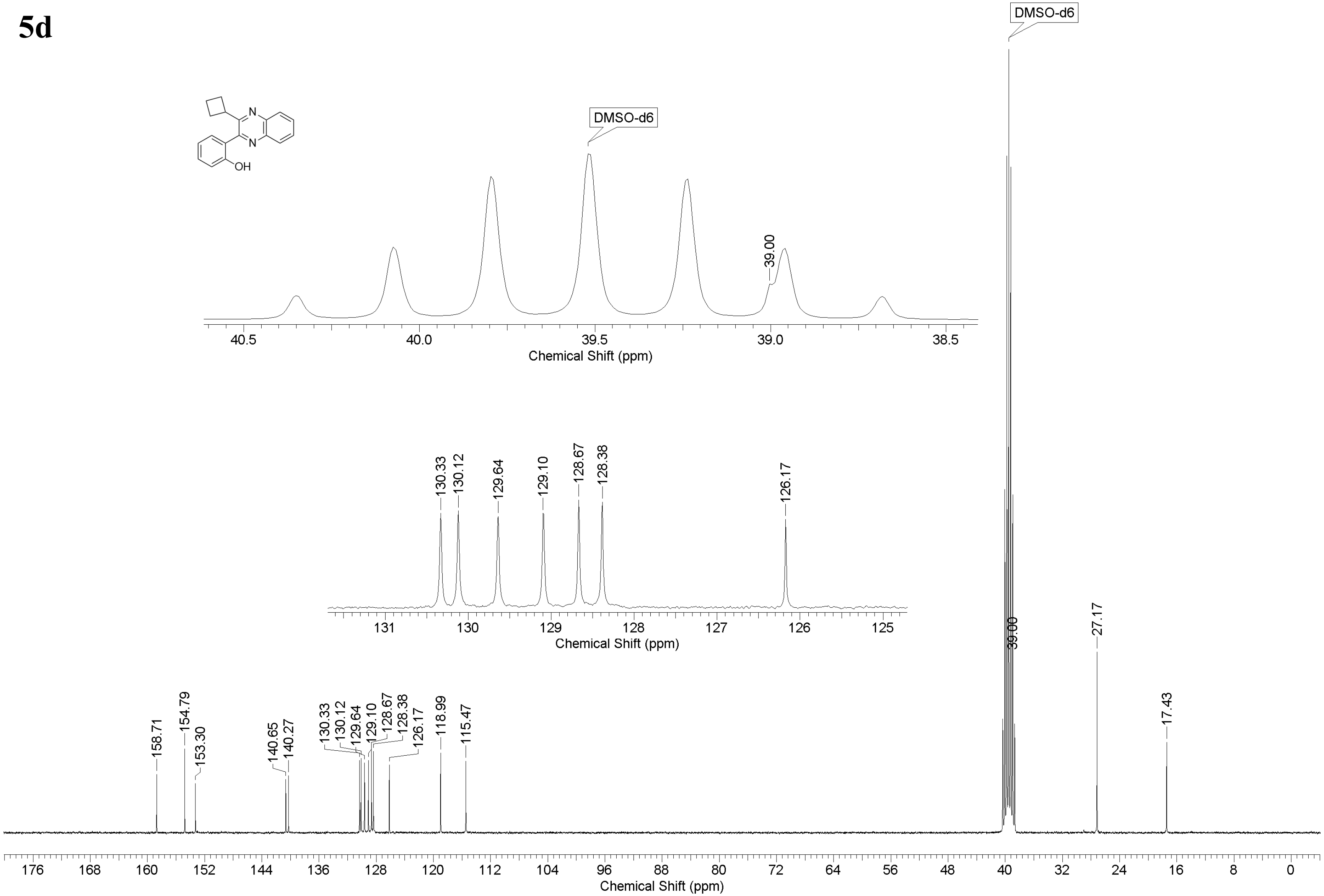

OH
N
DMSO-d6
39.00
40.5
40.0
39.5
39.0
38.5
Chemical Shift (ppm)
130.33
130.12
129.64
129.10
128.67
128.38
126.17
131
130
129
128
127
126
125
Chemical Shift (ppm)
158.71
154.79
153.30
140.65
140.27
118.99
115.47
27.17
17.43
176
168
160
152
144
136
128
120
112
104
96
88
80
72
64
56
48
40
32
24
16
8
0
Chemical Shift (ppm)

5f

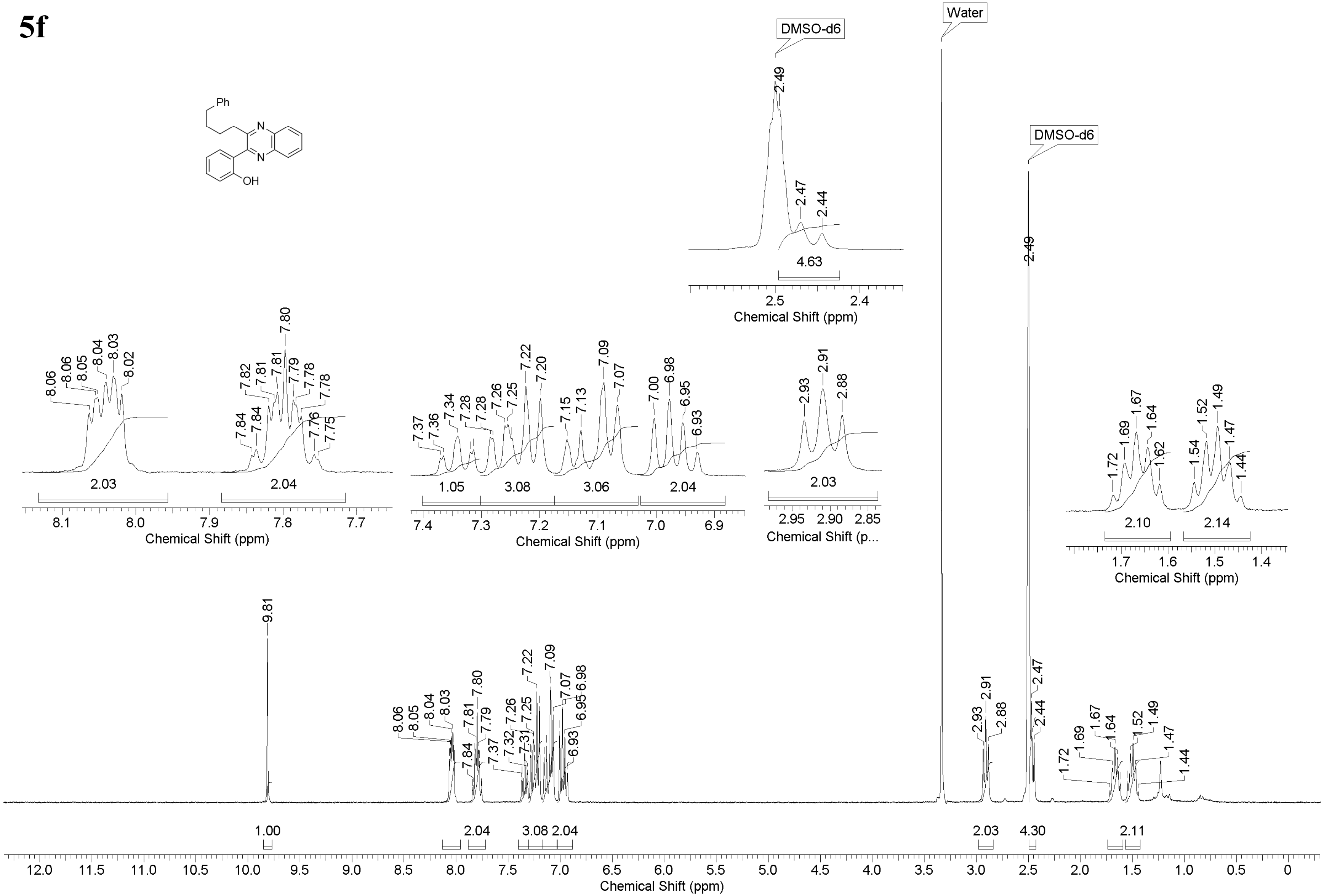
DMSO-d6
Water
DMSO-d6
4.63
2.5 2.4
Chemical Shift (ppm)
2.03 2.04 1.05 3.08 3.06 2.04 2.03 2.10 2.14
Chemical Shift (ppm)
1.00 2.04 3.08 2.04 2.03 4.30 2.11
12.0 11.5 11.0 10.5 10.0 9.5 9.0 8.5 8.0 7.5 7.0 6.5 6.0 5.5 5.0 4.5 4.0 3.5 3.0 2.5 2.0 1.5 1.0 0.5 0
Chemical Shift (ppm)

5f

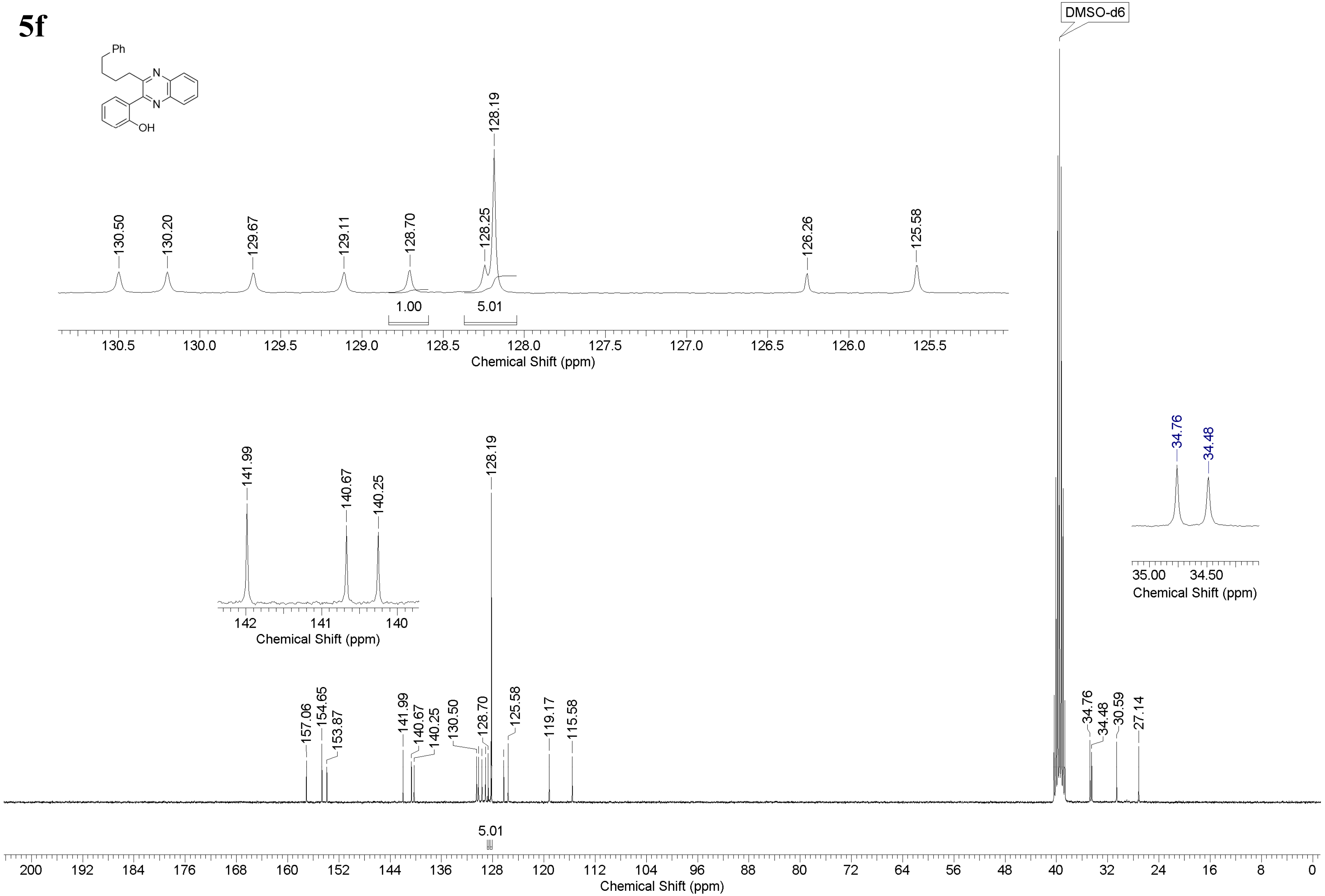
DMSO-d6
130.50
130.20
129.67
129.11
128.70
128.25
128.19
126.26
125.58
1.00
5.01
Chemical Shift (ppm)
141.99
140.67
140.25
34.76
34.48
157.06
154.65
153.87
119.17
115.58
30.59
27.14

6b

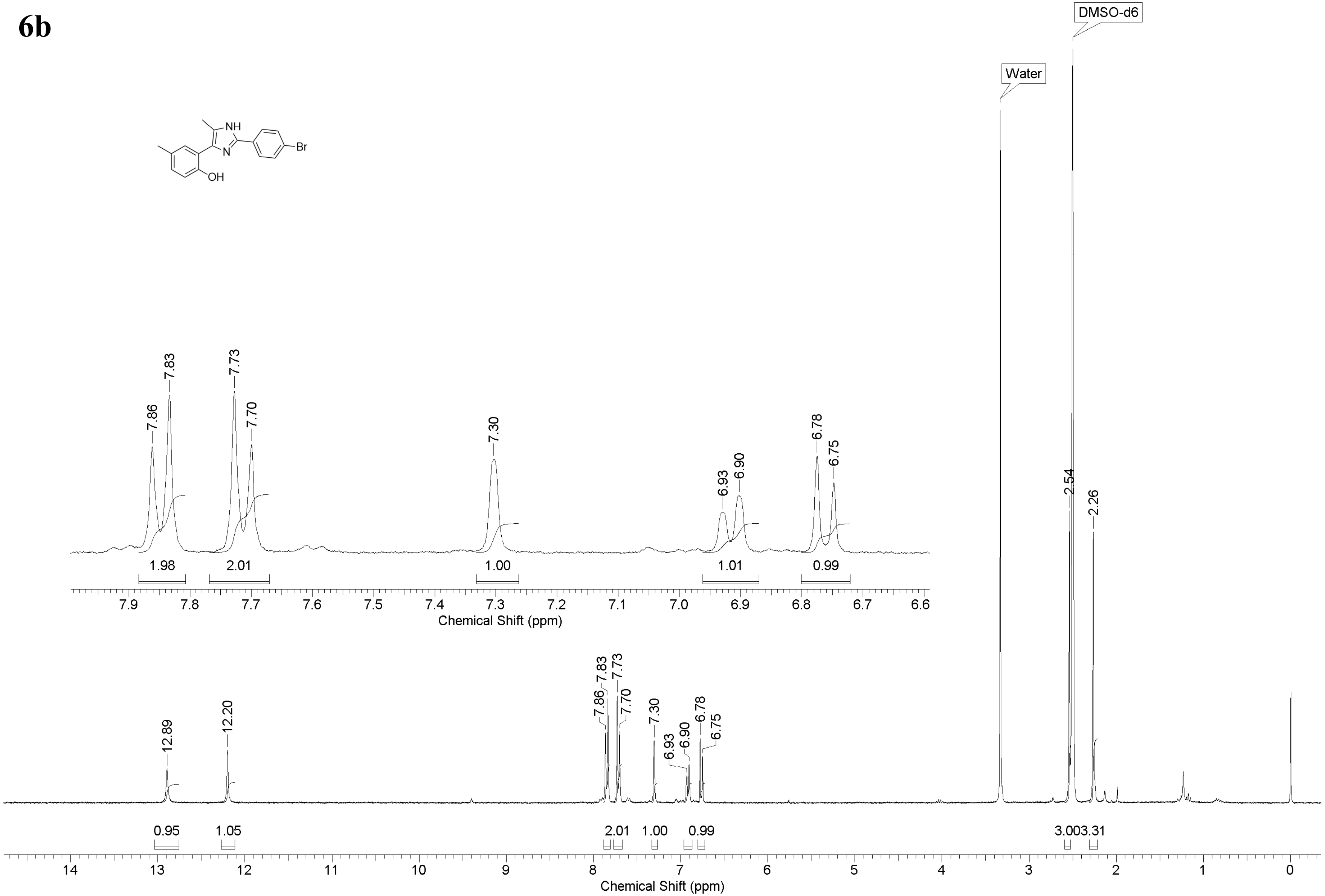

NH
N
Br
OH
DMSO-d6
Water
7.86
7.83
7.73
7.70
7.30
6.93
6.90
6.78
6.75
1.98
2.01
1.00
1.01
0.99
Chemical Shift (ppm)
12.89
12.20
2.54
2.26
0.95
1.05
3.00
3.31
Chemical Shift (ppm)

6b

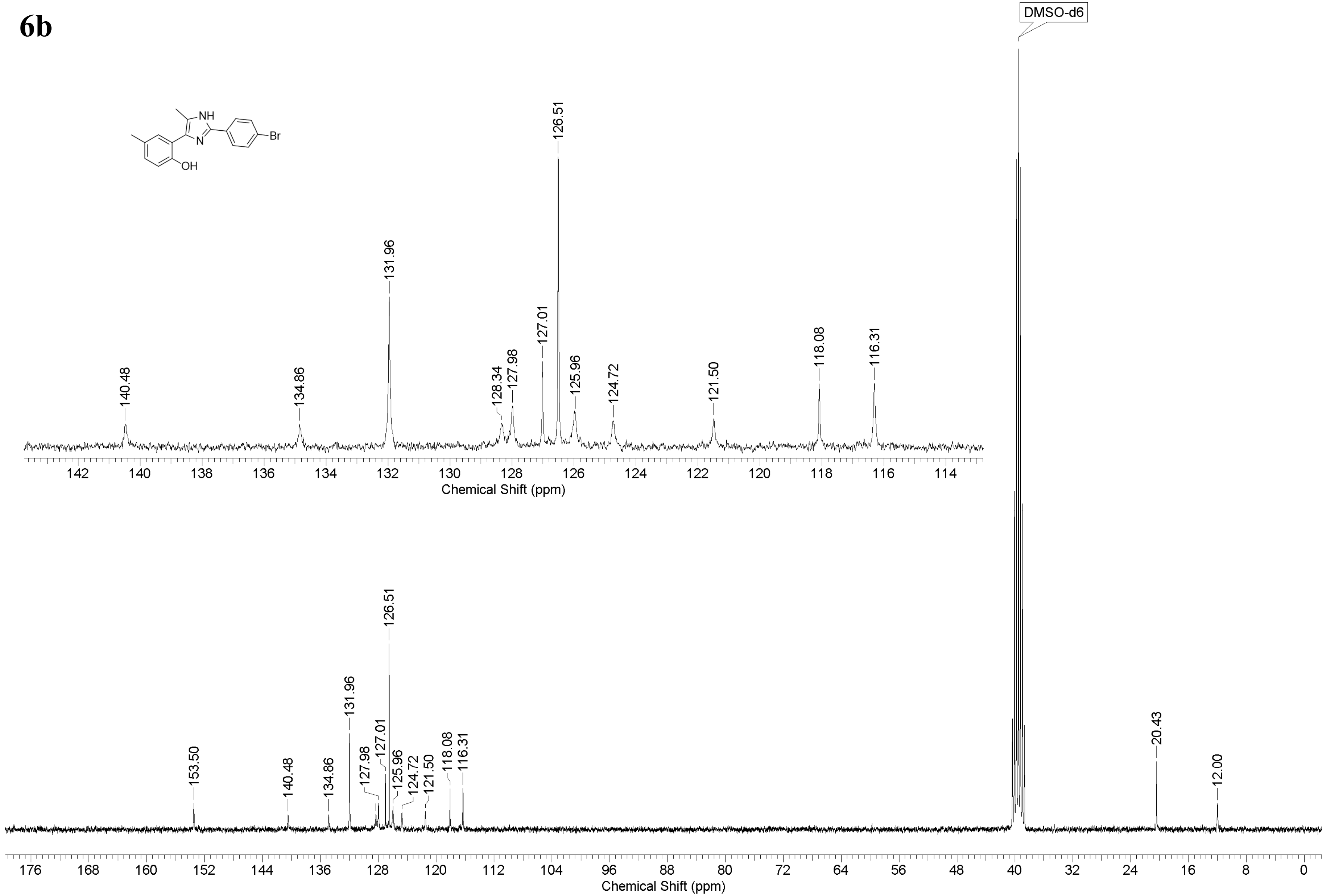
NH
N
Br
OH
DMSO-d6
140.48
134.86
131.96
128.34
127.98
127.01
126.51
125.96
124.72
121.50
118.08
116.31
142 140 138 136 134 132 130 128 126 124 122 120 118 116 114
Chemical Shift (ppm)
153.50
140.48
134.86
131.96
127.98
127.01
126.51
125.96
124.72
121.50
118.08
116.31
20.43
12.00
176 168 160 152 144 136 128 120 112 104 96 88 80 72 64 56 48 40 32 24 16 8 0
Chemical Shift (ppm)
